# Association of metabolic syndrome components and their combinations with functional disability among older adults in a longevity-associated ethnic minority region of Southwest China

**DOI:** 10.3389/fpubh.2025.1635390

**Published:** 2025-07-11

**Authors:** Haiyan Lu, Wenjie Liang, Hongyuan Huang, Kaiyong Huang, Lirong Zeng, Li Yang

**Affiliations:** ^1^Department of Occupational and Environmental Health, School of Public Health, Guangxi Medical University, Nanning, China; ^2^Department of Social Medicine, School of Public Health, Guangxi Medical University, Nanning, China; ^3^Department of Youth League Committee, School of Information and Management, Guangxi Medical University, Nanning, China

**Keywords:** metabolic syndrome, ADL disability, IADL disability, comorbidity, aging

## Abstract

**Purpose:**

Metabolic syndrome (MetS) is associated with functional disability; however, the associations between combinations of MetS components and functional disabilities remain largely unexplored.

**Methods:**

This cross-sectional study was conducted among adults aged ≥60 years in Donglan County. Basic activities of daily living (ADL) disability and instrumental activities of daily living (IADL) disability were identified using physical self-maintenance and IADL scales. Modified Poisson regression and restricted cubic splines were used to evaluate the associations of MetS, the number of MetS components, and combinations of MetS components with functional disability.

**Results:**

A total of 4,450 participants were enrolled in this study. Abdominal obesity was associated with a 1.03-fold (95% CI: 1.01–1.05) higher ADL disability risk. Lower HDL cholesterol remained associated with a 4% reduced risk of IADL disability (PR = 0.96, 95% CI: 0.93–0.99). The combination of abdominal obesity, elevated blood pressure, and elevated fasting glucose was correlated with a 1.08-fold (95% CI: 1.01–1.14) higher risk of ADL disability and a 1.12-fold (95% CI: 1.05–1.19) higher risk of IADL disability.

**Conclusion:**

Lower HDL cholesterol levels may serve as a protective factor against IADL disability. The combination of abdominal obesity, elevated blood pressure, and elevated fasting glucose appears to represent the highest-risk combination for both ADL disability and IADL disability in the older adult population.

## 1 Introduction

Functional independence refers to the ability to perform basic activities of daily living (ADL) and instrumental activities of daily living (IADL) (1), where ADL measures the ability to perform essential activities of functional living, and IADL assesses the ability to live independently in a community (1). According to the latest China Health and Retirement Longitudinal Study, the proportion of adults aged ≥60 years requiring ADL assistance declined from 11.7% (2011) to 8.1% (2020). When defining care needs using ADL or IADL criteria, this proportion decreased from 24.5 to 17.8% during the same period (2). However, the absolute number of older adults requiring care increased due to population aging and growth (1). Potential influencing factors of disability include age, living in an aged care house or with spouse/children, low monthly income, without health insurance, tight family expenses, having stroke or malignant tumor, irregular eating habit, smoking, sedentary lifestyle, lack of physical exercise, sleeping difficulty, lack of family support, and atrial fibrillation with diabetes mellitus type 2 (3–5). Disability is strongly associated with elevated mortality risk (6, 7), reduced health-related quality of life (8, 9), and cognitive decline (10).

Metabolic syndrome (MetS), a cluster of central obesity, raised blood pressure, raised triglycerides (TG), low high-density lipoprotein cholesterol (HDL-C), and elevated fasting plasma glucose (FPG), is recognized as an interesting potential risk factor for functional disability because of its potential reversibility with adequate pharmacological and health education interventions (11–13). The global prevalence of MetS is challenging to estimate because of the different criteria for its diagnosis; however, the prevalence in different countries showed an upward trend (14–16). The relationship between MetS and functional disability remains controversial; while some longitudinal studies found no association between MetS and ADL/IADL disability progression (17–19), others reported increased risks (20–23). The exact reasons for these inconsistent findings are unclear, but might depend on the diagnostic criteria of MetS, the definition of functional disability, lifestyle factors, and population characteristics. Therefore, a study on this topic in Guangxi is extremely urgent, especially in the remote ethnic minority longevity region of Southwest China.

Previous studied indicated that diabetes (24), hypertension (24, 25), low HDL-C (26, 27), TG (27), and central obesity/waist circumference (28, 29) were association with ADL disability or IADL disability. More importantly, MetS diagnosis requires ≥3 components, yet individuals may exhibit varying numbers (3–5) and distinct combinations of MetS components. Existing research has primarily focused on MetS presence or component count, with limited attention to combinations of MetS components. To date, only one study has analyzed ADL disability risks across pairs of MetS components, revealing striking disparities: individuals with hypertension combined with hyperglycemia had an almost 3-fold higher risk (hazard ratio = 3.51, 95% CI: 1.66–7.43) compared to those with dyslipidemia combined with hyperglycemia (hazard ratio = 1.32, 95% CI: 1.07–1.64) (30). No studies have explored the risks associated with different combinations of three and four MetS components with functional disability.

To address this gap, we conducted an analysis of the association between functional disability and MetS using modified Poisson regression, restricted cubic splines, subgroup analyses, and sensitive analyses based on data from Donglan County, which is one of the five counties of the Hongshuihe basin of Guangxi in China, a famous longevity area with Bama County as the center, to examine the associations of MetS status and component count with functional disability and identify high-risk combinations of MetS components affecting functional independence among adults aged ≥60 years.

## 2 Materials and methods

### 2.1 Study design and population

This cross-sectional survey was conducted between August 2016 and July 2018 in Donglan County, Hongshuihe Basin, Guangxi Zhuang Autonomous Region, Southwest China. The eligibility criteria were (a) age ≥60 years at enrollment and (b) ≥ 10 years of residency in the study townships. The exclusion criteria were (a) diagnosis of neurological/psychiatric disorders (e.g., Alzheimer’s disease, dementia, cognitive impairment), (b) severe sensory impairments (blindness, deafness, or mutism), and (c) active malignancy. Participants were invited to designated local clinics for structured interviews and health examinations, with site selection based on medical resource availability, village distribution, and logistical feasibility. Data collection included (a) demographic/socioeconomic characteristics and lifestyle factors via face-to-face questionnaires; (b) clinical measurements (blood pressure, anthropometrics); and (c) fasting blood samples analyzed at the laboratory of the School of Public Health, Guangxi Medical University for TG, HDL-C, and FPG. The study protocol was approved by the Ethics Committee of Guangxi Medical University (approval no.: 201503010–2). All participants provided written informed consent or fingerprint signatures.

The minimum sample size was estimated using the formula (31): $\;n=\frac{{\left(z_{1-\alpha }\right)}^{2}\times p\times q}{d^{2}}$ , *p* = expected prevalence rate; *q* = 1–*p*; *d* = acceptable margin of error (set as 0.1 × *p*); Z_1–*α*/2_ = 1.96 (*α* = 0.05). Prevalence estimates were derived from prior studies in Western China (2, 32): ADL disability = 12.1%, Comorbid ADL-IADL disability = 26.9% (2), and IADL disability = 24.2% (assumed ≥2 × ADL disability prevalence). Minimum required sample size:


$$\mathrm{ADL}\;\text{disability}:n=1.96^{2}\times 0.121\times 0.879/0.0121^{2}=2,791;$$

$$\text{IADL disability}:n=1.96^{2}\times 0.242\times 0.758/0.0242^{2}=1,204;$$

$$\begin{array}{l} \text{Comorbid}\;\mathrm{ADL}­\text{IADL disability}:n=1.96^{2}\times 0.269\\ \times 0.731/0.0269^{2}=1,044 \end{array}$$

**Figure 1:**
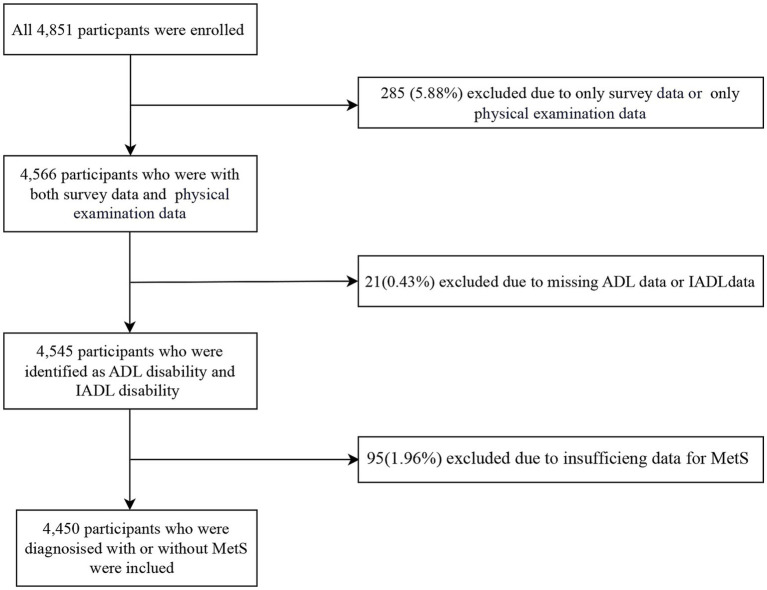
The selection process of population in the present population. MetS, metabolic syndrome; ADL, activities of daily living; IADL, instrumental activities of daily living.

Among the 4,851 initially enrolled participants, 401 were excluded because of incomplete MetS/ADL/IADL data, yielding a ‌final analytic sample of 4,450 (Figure 1).

### 2.2 Outcome variables

Functional ability was assessed through self-reported measures using Chinese-adapted versions of the physical self-maintenance and IADL scales (33, 34). The ADL disability evaluation employed the Physical Self-Maintenance Scale, a six-item instrument that measures independence in the following basic tasks: walking, feeding, dressing, grooming, bathing, and toileting. Concurrently, the IADL disability assessment utilized an eight-item scale to evaluate competence in more complex physical and cognitive tasks, such as transportation use, food preparation, housekeeping, medication management, shopping, laundry, telephone operation, and financial management.

Both scales employed a hierarchical four-point scoring system: (1) “Independent without difficulty,” (2) “Independent with some difficulty,” (3) “Requires partial assistance,” and (4) “Completely dependent.” A score ≥2 on any item indicated functional limitation (34). Operational definitions of functional disabilities were established as follows: ADL disability denoted limitations in ≥1 Physical Self-Maintenance Scale item; IADL disability reflected limitations in ≥1 IADL scale item; and Comorbid ADL-IADL disability was defined as concurrent limitations in ≥1 Physical Self-Maintenance Scale item and ≥1 IADL scale item. This dichotomization categorized outcomes as either “no limitation” or “disability”.

Psychometric analysis revealed strong internal consistency, with Cronbach’s alpha coefficients of 0.907 for the Physical Self-Maintenance Scale and 0.857 for the IADL scale, confirming measurement reliability.

### 2.3 Main explanatory variables

#### 2.3.1 MetS definition

MetS was diagnosed according to the 2009 Joint Interim Statement consensus criteria, requiring ≥3 of the following components (35): abdominal obesity, waist circumference (WC) ≥ 85 cm (men)/≥ 80 cm (women); Elevated TG, TG ≥ 150 mg/dL or lipid-lowering therapy; Reduced HDL-C: HDL-C < 40 mg/dL (men)/<50 mg/dL or lipid-regulating therapy; elevated blood pressure, Systolic BP ≥ 130 mmHg and/or diastolic BP ≥ 85 mmHg or antihypertensive treatment; and Elevated FPG: FPG ≥ 100 mg/dL or antidiabetic therapy.

#### 2.3.2 Variable operationalization

MetS status was dichotomized (presence/absence).

The component count was categorized as follows: 0, 1, 2, 3, and ≥4.

#### 2.3.3 Component combination analysis

To ensure the comparability of group sizes and to better fit Modified Poisson Regression Model, combinations with fewer than 50 participants were categorized into “other pairs,” “other triplets,” and “other quadruplets” groups. Pairwise combinations (10 total divided into 5 groups): elevated blood pressure + abdominal obesity, elevated blood pressure + elevated fasting glucose, elevated blood pressure + elevated triglycerides, elevated blood pressure + reduced HDL-C, and other pairs. Triplet combinations (10 total divided into 5 groups): elevated blood pressure + abdominal obesity + elevated TG, elevated blood pressure + abdominal obesity + elevated FPG, elevated blood pressure + abdominal obesity + reduced HDL-C, elevated blood pressure + elevated FPG + elevated TG, and other triplets. Quadruplet combinations (5 total divided into 3 groups): elevated blood pressure + abdominal obesity + elevated TG + elevated FPG, elevated blood pressure + abdominal obesity + elevated TG + reduced HDL-C, and other quadruplets. All combination frequencies and proportions are detailed in Supplementary Table S1.

### 2.4 Covariates

This study considered the following variables as potential confounders: sociodemographic variables (age, sex, ethnicity, marital status, educational attainment, occupation, and annual income), lifestyle factors (smoking status and alcohol consumption), chronic diseases (cerebrovascular disease, rheumatism, and osteoarthropathy), and physical examination indicators [handgrip strength, hemoglobin (Hb), total cholesterol (TC), low-density lipoprotein cholesterol (LDL-C), aspartate aminotransferase (AST), alanine transaminase (ALT), serum creatinine (Scr), and uric acid]. Further details of these covariates, including the measurement methods and categorization criteria, are provided in Supplementary Table S2.

### 2.5 Statistical analysis

The Kolmogorov–Smirnov test were used to determine whether the variables followed a normal distribution. Continuous variables with a normal distribution are presented as mean ± standard deviation (SD), and those with a skewed distribution are reported as median and interquartile range (IQR). Categorical variables were described as frequencies and percentages. Independent sample t-tests (normal distribution) or nonparametric tests (skewed distribution) for continuous variables, with difference and 95% CI. Chi-square tests for categorical variables, with difference and 95% CI; because of potentially inflated type I error due to multiple comparisons, *post hoc* comparisons were corrected using Bonferroni method for multiple categorical variables. Additionally, we evaluated the trends in disability outcomes across the number of MetS components. Moreover, we conducted chi-square tests to assess the variation in disability outcomes among individual MetS components and dual-component, triple-component, and ≥4-component combinations.

Modified Poisson regression models with a robust (sandwich) estimation of variance were appropriate for cross-sectional surveys to calculate the prevalence ratio (PR) and 95% CI when the rare disease assumption was violated (36). This method was used to assess the association of MetS status, the number of MetS components, and combinations of MetS components with functional disabilities. In addition, trends in functional disability severity across increasing numbers of MetS components were analyzed using modified Poisson regression models. For model diagnostics, we utilized the likelihood ratio chi-square test and the ratio of deviance statistic to degrees of freedom. If the *p*-value of the likelihood ratio chi-square test is less than 0.05, this indicates that the modified Poisson regression model demonstrates superiority over the null model in data fitting, with statistically significant. The ratio of deviance statistic to degrees of freedom closer to 1 suggests better model fit.

Restricted cubic spline (RCS) regression models were used to visualize the potential non-linear relationships between continuous SBP, DBP, FPG, TG, HDL-C, and WC with functional disability.

To identify potential impact factors in the associations of MetS status, number of MetS components, and individual MetS components with functional disability, we performed subgroup analyses stratified by all covariates and multiplicative/additive interaction analyses using modified Poisson regression models. The PR with a 95% confidence interval of the product term was used to measure the multiplicative interaction. The relative excess risk due to interaction (RERI), attributable proportion due to interaction (AP), and synergy index (SI) were used to evaluate the additive interaction, calculated using the coefficients and corresponding standard errors of the product term as well as the covariance matrix (37).

Several sensitivity analyses were performed. First, to assess the robustness of the results, we consistently adjusted for covariates in the different models. Second, to test the robustness and consistency of our findings, we repeated all analyses using binary logistic regression. Third, to determine the robustness of the associations between combinations of MetS components and functional disability, we repeated the analyses in the Zhuang population, farmer population, and non-drinkers.

Missingness for all covariates was <5% (Supplementary Table S3). Missing values were coded as “not reported/unknown” in the regression models without imputation. The tests were two-sided, and statistical significance was set at *p* < 0.05. All analyses were performed using IBM SPSS software (version 26.0; IBM Corp., Armonk, NY, United States).

## 3 Results

### 3.1 Basic baseline characteristics of the study participants

The study included 4,450 participants with a mean age of 70.06 ± 7.37 years. The prevalence of disability was 8.56, 31.01, and 7.75% for ADL, IADL, and comorbid ADL-IADL, respectively. The majority of the participants were female (59.69%), of Zhuang ethnicity (85.43%), married (69.35%), farmers (91.40%), had less than a primary school education (46.20%), reported an annual household income of ≥30,000 renminbi (35.56%), were non-smokers (84.34%), non-drinkers (76.68%), did not have cerebrovascular disease (95.44%), did not have rheumatism (88.76%), did not have osteoarthropathy (84.92%), had low handgrip strength (57.56%), and were not anemic (63.96%). Statistically significant differences were observed in age, ethnicity, marital status, educational attainment, occupation, annual income, alcohol consumption, handgrip strength, anemia, ALT, and Scr across ADL disability, IADL disability, and comorbid ADL-IADL disability groups (all *p* < 0.05). The complete baseline characteristics are presented in Table 1.

**Table 1:** General characteristics of the study participants according to functional disability.

Variables	Total	ADL disability				IADL disability				Comorbid ADL-IADL disability			
		No	Yes	RD (95% CI)	*p* value	No	Yes	RD (95%CI)	*p* value	No	Yes	RD (95%CI)	*p* value
Total [n (%)]	4,450 (100.00)	4,069 (91.44)	381 (8.56)			3,070 (68.99)	1,380 (31.01)			4,105 (92.25)	345(7.75)		
Gender [n (%)]				−0.001 (−0.018, 0.015)	0.878			**0.193 (0.166, 0.219)**	**<0.001**			0.004 (−0.012, 0.020)	0.641
Male	1794 (40.31)	1,639 (91.36)	155 (8.64)			1,444 (80.49)	350 (19.51)			1,659 (92.47)	135 (7.53)		
Female	2,656 (59.69)	2,430 (91.49)	226 (8.51)			1,626 (61.22)	1,030 (38.78)			2,446 (92.09)	210 (7.91)		
Age group [n (%)]				**0.086 (0.069, 0.102)**	<**0.001**			**0.208 (0.181, 0.235)**	<**0.001**			**0.083 (0.067, 0.099)**	**<0.001**
60–69 years	2,357 (52.97)	2,250 (95.46)	107 (4.54)			1857 (78.79)	500 (21.21)			2,266 (96.14)	91 (3.86)		
≥ 70 years	2093 (47.03)	1819 (86.91)	274 (13.09)			1,213 (57.96)	880 (42.04)			1839 (87.86)	254 (12.14)		
Ethnic [n (%)]				**−0.035 (−0.063, −0.010)**	**0.003**			**−0.053 (−0.093, −0.014)**	**0.007**			**−0.032 (−0.058, −0.008)**	**0.004**
Non-zhuang	648 (14.57)	573 (88.43)	75 (11.57)			418 (64.51)	230 (35.49)			580 (89.51)	68 (10.49)		
Zhuang	3,799 (85.43)	3,494 (91.97)	305 (8.03)			2,651 (69.78)	1,148 (30.22)			3,523 (92.73)	276 (7.27)		
Marital status [n (%)]				**0.049 (0.030, 0.069)**	<**0.001**			**0.160 (0.130, 0.191)**	**<0.001**			**0.050 (0.032, 0.069)**	**<0.001**
Partnered	3,081 (69.35)	2,864 (92.96)	217 (7.04)			2,277 (73.90)	804 (26.10)			2,890 (93.80)	191 (6.20)		
Single	1,362 (30.65)	1,199 (88.03)	163 (11.97)			788 (57.86)	574 (42.14)			1,209 (88.77)	153 (11.23)		
Educational attainment [n (%)]					**0.011** ^**▲**^				<**0.001**^**●**^				<**0.001**^**●**^
Less than primary school	2051 (46.20)	1856 (90.49)	195 (9.51)			1,176 (57.34)	875 (42.66)	***−0.162 (−0.193, −0.130)*** ^***a***^	***<0.001*** ^***a***^	1865 (90.93)	186 (9.07)		
Primary school	1,480 (33.33)	1,351 (91.28)	129 (8.72)			1,088 (73.51)	392 (26.49)	***−0.145 (−0.175, −0.114)*** ^***b***^	***<0.001*** ^***b***^	1,364 (92.16)	116 (7.84)	***−0.032 (−0.051, −0.012)c***	***0.006c***
High school and above	909 (20.47)	853 (93.84)	56 (6.16)	***−0.033 (−0.053, −0.013)d***	***0.008*** ^***d***^	800 (88.01)	109 (11.99)	***0.307 (−0.336, −0.276)*** ^***e***^	***<0.001*** ^***e***^	867 (95.38)	42 (4.62)	***−0.044 (−0.062, −0.025)*** ^***f***^	***<0.001*** ^***f***^
Occupation [n (%)]				0.031 (0.003, 0.054)	**0.041**			**0.211 (0.173, 0.244)**	<**0.001**			**0.030 (0.004–0.052)**	**0.034**
Non-farmer	380 (8.60)	358 (94.21)	22 (5.79)			335 (88.16)	45 (11.84)			361 (95.00)	19 (5.00)		
Farmer	4,040 (91.40)	3,682 (91.14)	358 (8.86)			2,711 (67.10)	1,329 (32.90)			3,715 (91.96)	325 (8.04)		
Annual income [n (%)]					<**0.001**^**●**^				<**0.001**^**●**^				<**0.001**^**●**^
<10,000 RMB	1,680 (38.12)	1,498 (89.17)	182 (10.83)			1,018 (60.60)	662 (39.40)	***−0.066 (−0.100, −0.033)*** ^***g***^	***<0.001*** ^***g***^	1,512 (90.00)	168 (10.00)		
10,000–29,999 RMB	1,160 (26.32)	1,077 (92.84)	83 (7.16)	***−0.037 (−0.058, −0.015)*** ^***h***^	***0.003*** ^***h***^	816 (70.34)	344 (29.66)	***−0.097 (−0.132, −0.062)*** ^***i***^	***<0.001*** ^***i***^	1,085 (93.53)	75 (6.47)	***−0.035 (−0.055, −0.015)*** ^***j***^	***0.003*** ^***j***^
≥ 30,000 RMB	1,567 (35.56)	1,454 (92.79)	113 (7.21)	**−0.036 (−0.056, −0.016)** ^**k**^	***0.001*** ^***k***^	1,206 (76.96)	361 (23.04)	**−0.164 (−0.195, −0.132)** ^**l**^	***<0.001*** ^***l***^	1,468 (93.68)	99 (6.32)	***−0.037 (−0.056, −0.018)*** ^***m***^	***<0.001*** ^***m***^
Cerebrovascular disease [n (%)]				0.008 (−0.029, 0.054)	0.678			−0.026 (−0.087, 0.040)	0.443			0.012 (−0.025, 0.056)	0.543
No	4,247 (95.44)	3,885 (91.48)	362 (8.52)			2,925 (68.87)	1,322 (31.13)			3,920 (92.30)	327 (7.70)		
Yes	203 (4.56)	184 (90.64)	19 (9.36)			145 (71.43)	58 (28.57)			185 (91.13)	18 (8.87)		
Rheumatism [n (%)]				−0.004 (−0.028, 0.023)	0.755			−0.005 (−0.047, 0.039)	0.852			0.001 (−0.023, 0.027)	0.97
No	3,947 (88.76)	3,607 (91.39)	340 (8.61)			2,720 (68.91)	1,227 (31.09)			3,641 (92.25)	306 (7.75)		
Yes	500 (11.24)	459 (91.80)	41 (8.20)			347 (69.40)	153 (30.60)			461 (92.20)	39 (7.80)		
Osteoarthropathy [n (%)]				−0.004 (−0.026, 0.019)	0.712			−0.039 (−0.075, −0.001)	**0.045**			−0.004 (−0.024, 0.019)	0.75
No	3,778 (84.92)	3,452 (91.37)	326 (8.63)			2,584 (68.40)	1,194 (31.60)			3,483 (92.19)	295 (7.81)		
Yes	671 (15.08)	616 (91.80)	55 (8.20)			485 (72.28)	186 (27.72)			621 (92.55)	50 (7.45)		
Smoking status [n (%)]				−0.016 (−0.037, 0.006)	0.154			−0.164 (−0.195, −0.131)	<**0.001**			−0.017 (−0.036, 0.004)	0.122
No	3,749 (84.34)	3,418 (91.17)	331 (8.83)			2,489 (66.39)	1,260 (33.61)			3,448 (91.97)	301 (8.03)		
Yes	696 (15.66)	646 (92.82)	50 (7.18)			576 (82.76)	120 (17.24)			652 (93.68)	44 (6.32)		
Alcohol consumption [n (%)]				−0.026 (−0.043, −0.007)	**0.009**			−0.159 (−0.188, −0.130)	<**0.001**			−0.029 (−0.046, −0.012)	**0.002**
No	3,394 (76.68)	3,082 (90.81)	312 (9.19)			2,215 (65.26)	1,179 (34.74)			3,107 (91.54)	287 (8.46)		
Yes	1,032 (23.32)	964 (93.41)	68 (6.59)			838 (81.20)	194 (18.80)			975 (94.48)	57 (5.52)		
Low handgrip strength [n (%)]				0.058 (0.043, 0.074)	<**0.001**			0.153 (0.126, 0.179)	<**0.001**			0.056 (0.041, 0.071)	<**0.001**
No	1882 (42.44)	1787 (94.95)	95 (5.05)			1,466 (77.90)	416 (22.10)			1800 (95.64)	82 (4.36)		
Yes	2,552 (57.56)	2,274 (89.11)	278 (10.89)			1,598 (62.62)	954 (37.38)			2,297 (90.01)	255 (9.99)		
Anemia [n (%)]				0.035 (0.017, 0.053)	<**0.001**			0.096 (0.067, 0.125)	<**0.001**			0.037 (0.019, 0.054)	<**0.001**
No	2,838 (63.96)	2,631 (92.71)	207 (7.29)			2057 (72.48)	781 (27.52)			2,656 (93.59)	182 (6.41)		
Yes	1,599 (36.04)	1,427 (89.24)	172 (10.76)			1,005 (62.85)	594 (37.15)			1,438 (89.93)	161 (10.07)		
LDL-C [mmol/L, median(IQR)]	4,289 (96.38)	3.20 (1.20)	3.20 (1.20)	0.10 (0.01, 0.20)	0.225	3.20 (1.20)	3.15 (1.20)	**0.10 (0.01, 0.10)**	**0.021**	3.20 (1.20)	3.20 (1.20)	0.10 (0.01, 0.20)	0.256
TC [mmol/L, median(IQR)]	4,450 (100.00)	5.34 (1.41)	5.29 (1.37)	0.08 (−0.04, 0.19)	0.178	5.35 (1.40)	5.30 (1.45)	0.05 (−0.02, 0.12)	0.152	5.34 (1.41)	5.29 (1.40)	0.08 (−0.04, 0.20)	0.166
AST [U/L, median(IQR)]	4,428 (99.51)	28.00 (10.00)	28.00 (11.00)	**1.00 (0.001, 2.00)**	**0.027**	28.00 (10.00)	28.00 (11.00)	0.01 (0.001, 1.00)	0.227	28.00 (10.00)	27.00 (11.00)	**1.00 (0.01, 2.00)**	**0.01**
ALT [U/L, median(IQR)]	4,428 (99.51)	18.00 (11.00)	16.00 (12.00)	**2.00 (1.00, 3.00)**	<**0.001**	18.50 (11.00)	16.00 (10.00)	**2.00 (2.00, 3.00)**	<**0.001**	18.00 (11.00)	16.00 (12.00)	**2.00 (1.00, 3.00)**	<**0.001**
Serum creatinine [μmol/L, median(IQR)]	4,428 (99.51)	62.00 (26.00)	66.00 (32.00)	**−3.00 (−5.00, −0.90)**	**0.015**	63.00 (25.00)	60.00 (27.00)	**3.70 (2.00, 5.00)**	<**0.001**	62.00 (26.00)	66.00 (33.00)	**−3.00 (−6.00, −0.50)**	**0.018**
Uric acid [μmol/L, median(IQR)]	4,450 (100.00)	308.00 (149.00)	329.00 (164.00)	**−14.00 (−26.00, −2.00)**	**0.024**	312.00 (150.00)	307.00 (148.75)	5.00 (−2.00, 12.00)	0.132	308.00 (149.00)	332.00 (169.50)	**−14.00 (−27.00, −1.00)**	**0.034**
SBP [mmHg, median(IQR)]	4,449 (99.98)	136.00 (28.00)	141.00 (31.00)	**−4.00 (−7.00, −2.00)**	<**0.001**	135.00 (28.00)	139.00 (30.00)	**−2.00 (−3.00, −1.00)**	**0.003**	136.00 (29.00)	141.00 (31.00)	**−4.00 (−7.00, −2.00)**	<**0.001**
DBP [mmHg, median(IQR)]	4,449 (99.98)	79.00 (15.00)	79.00 (19.00)	−1.00 (−2.00, 1.00)	0.279	79.00 (15.00)	78.00 (16.00)	1.00 (0.01, 1.00)	0.097	79.00 (15.00)	79.00 (19.00)	−1.00 (−2.00, 1.00)	0.416
FBG [mmol/L, median(IQR)]	4,450 (100.00)	4.74 (0.94)	4.70 (1.15)	−0.01 (−0.10, 0.08)	0.842	4.73 (0.96)	4.75 (0.96)	−0.04 (−0.09, 0.01)	0.138	4.74 (0.94)	4.69 (1.17)	0.00 (−0.09, 0.10)	0.948
TG [mmol/L, median(IQR)]	4,450 (100.00)	1.05 (0.67)	1.12 (0.73)	**−0.06 (−0.11, −0.01)**	**0.019**	1.06 (0.69)	1.04 (0.63)	**0.03 (0.10, 0.06)**	**0.026**	1.05 (0.67)	1.10 (0.72)	**−0.05 (−0.10, 0.00)**	**0.047**
HDL-C [mmol/L, median(IQR)]	4,450 (100.00)	1.54 (0.43)	1.52 (0.46)	**0.04 (0.01, 0.08)**	**0.017**	1.52 (0.43)	1.57 (0.42)	**−0.03 (−0.05, −0.01)**	**0.003**	1.54 (0.43)	1.52 (0.46)	**0.05 (0.01, 0.08)**	**0.013**
WC [cm, median(IQR)]	4,450 (100.00)	77.00 (12.00)	79.00 (11.50)	**−2.00 (−3.00, −2.00)**	<**0.001**	77.00 (12.00)	77.00 (12.00)	0.01 (0.001, 1.00)	0.701	77.00 (12.00)	79.00 (12.00)	**−2.00 (−3.00, −1.00)**	<**0.001**
MetS [n (%)]				**0.048 (0.024, 0.074)**	<**0.001**			0.006 (−0.030, 0.043)	0.739			**0.040 (0.017, 0.065)**	<**0.001**
No	3,711 (83.39)	3,423 (92.24)	288 (7.76)			2,564 (69.09)	1,147 (30.91)			3,448 (92.91)	263 (7.09)		
Yes	739 (16.61)	646 (87.42)	93 (12.58)			506 (68.47)	233 (31.53)			657 (88.90)	82 (11.10)		
MetS components [n (%)]					<**0.001**^**●**^				**0.049** ^**★**^				<**0.001**^**●**^
0	784 (17.62)	738 (94.13)	46 (5.87)			572 (72.96)	212 (27.04)			744 (94.90)	40 (5.10)		
1	1780 (40)	1,648 (92.58)	132 (7.42)	***0.055 (0.025, 0.087)*** ^***n***^	***0.001*** ^***n***^	1,230 (69.10)	550 (30.90)			1,656 (93.03)	124 (6.97)	***0.045 (0.016, 0.075)*** ^***o***^	***0.008*** ^***o***^
2	1,147 (25.78)	1,037 (90.41)	110 (9.59)	***0.037 (0.013, 0.061)*** ^***p***^	***0.032*** ^***p***^	762 (66.43)	385 (33.57)	***0.065 (0.024, 0.106)*** ^***q***^	***0.023*** ^***q***^	1,048 (91.37)	99 (8.63)	***0.035 (0.012, 0.057)*** ^***r***^	***0.032*** ^***r***^
3	542 (12.18)	472 (87.08)	70 (12.92)	***0.070 (0.038, 0.104)*** ^***s***^	***<0.001*** ^***s***^	369 (68.08)	173 (31.92)			480 (88.56)	62 (11.44)	***0.063 (0.033, 0.095)*** ^***t***^	***<0.001*** ^***t***^
≧ 4	197 (4.42)	174 (88.32)	23 (11.68)	***0.058 (0.013, 0.109)*** ^***u***^	***0.044*** ^***u***^	137 (69.54)	60 (30.46)			177 (89.85)	20 (10.15)		

MetS, metabolic syndrome; ADL, activities of daily living; IADL, instrumental activities of daily living; SD, standard deviation; IQR, interquartile range; LDL-C, low density lipoprotein cholesterol; TC, total cholesterol; AST, aspartate aminotransferase; ALT, alanine aminotransferase; SBP, systolic blood pressure; DBP, diastolic blood pressure; FBG, fasting blood glucose; TG, triglycerides; HDL-C, high density lipoprotein cholesterol; WC, waist circumference. Independent sample *t*-tests (normal distribution) or nonparametric tests (skewed distribution) for continuous variables, with difference and 95% CI. Chi-square tests for categorical variables, with difference and 95% CI; because of potentially inflated type I error due to multiple comparisons, post hoc comparisons were corrected using Bonferroni method for multiple categorical variables. The bold values indicate statistically significant differences. ^▲^*p* for trend = 0.004; ^●^*p* for trend <0.001. ^★^*p* for trend = 0.039. ^a^Less than primary school VS. High school and above for IADL disability; ^b^Less than primary school VS. Primary school for IADL disability; ^c^Primary school VS. High school and above for comorbid ADL-IADL disability; ^d^Less than primary school VS. High school and above for ADL disability; ^e^Less than primary school VS. High school and above for IADL disability; ^f^Less than primary school VS. High school and above for comorbid ADL-IADL disability. ^g^10,000–29,999 RMB VS. ≥ 30,000 RMB for IADL disability; ^h^<10,000 RMB VS. 10,000–29,999 RMB for ADL disability; ^i^<10,000 RMB VS. 10,000–29,999 RMB for IADL disability; ^j^<10,000 RMB VS. 10,000–29,999 RMB for comorbid ADL-IADL disability; ^k^<10,000 RMB VS. ≥ 30,000 RMB for ADL disability; ^l^<10,000 RMB VS. ≥ 30,000 RMB for IADL disability; ^m^<10,000 RMB VS. ≥ 30,000 RMB for comorbid ADL-IADL disability. ^n^1 VS. 3 for ADL disability; ^o^1 VS. 3 for comorbid ADL-IADL disability; ^p^0 VS. 2 for ADL disability; ^q^0 VS. 2 for IADL disability; ^r^0 VS. 2 for comorbid ADL-IADL disability; ^s^0 VS. 3 for ADL disability; ^t^0 VS. 3 for comorbid ADL-IADL disability; ^u^0 VS. ≥4 for comorbid ADL-IADL disability.

### 3.2 Functional disability in older adults with MetS and its components/combinations

As shown in Table 1, 16.61% (*n* = 739) of the participants had MetS. Older adults with MetS demonstrated significantly higher rates of both ADL disability (12.58% vs. 7.76%; *p* < 0.001) and comorbid ADL-IADL disability (11.10% vs. 7.09%; *p* < 0.001) than those without MetS.

Notably, the prevalence of ADL disability, IADL disability, and comorbid ADL-IADL disability among older adults with varying numbers of MetS components was significantly different (all group comparisons, *p* < 0.05). The prevalence of ADL disability, IADL disability, and comorbid ADL-IADL disability increased as the number of MetS components increased (*p* for trends < 0.001, 0.039, and < 0.001, respectively).

Notably, IADL disability differed among the combinations of the three MetS components (*p* < 0.001). No significant differences were observed for other multicomponent combinations across all disability categories. Additional data are presented in Supplementary Table S4.

### 3.3 Association of MetS, number of MetS components, and combinations of MetS components with functional disability

As demonstrated in Table 2, participants with MetS had a 1.04-fold higher risk of ADL disability (95% CI: 1.02–1.07) and a 1.53-fold elevated risk of comorbid ADL-IADL disability (95% CI: 1.18–1.97) compared to individuals without MetS in fully adjusted models (Model 3).

**Table 2:** Associations of MetS with functional disability in older adults population.

Models	ADL disability [PR (95% CI)]		IADL disability [PR (95% CI)]		Comorbid ADL-IADL disability [PR (95% CI)]	
	Without MetS	With MetS	Without MetS	With MetS	Without MetS	With MetS
Model 1	1.00 (Ref)	1.05 (1.02,1.07)***	1.00 (Ref)	1.01 (0.98,1.03)	1.00 (Ref)	1.57 (1.24,1.98)***
Model 2	1.00 (Ref)	1.04 (1.02,1.07)***	1.00 (Ref)	0.99 (0.97,1.02)	1.00 (Ref)	1.54 (1.22,1.94)***
Model 3	1.00 (Ref)	1.04 (1.02,1.07)***	1.00 (Ref)	0.99 (0.96,1.01)	1.00 (Ref)	1.53 (1.18,1.97)**

MetS, metabolic syndrome; ADL, activities of daily living; IADL, instrumental activities of daily living; PR, prevalence ratio; CI, confidence interval. The bold values indicated statistically significant differences.**, *p*<0.01; ***, *p*<0.001. Modified Poisson regression models were calculated associations of MetS with functional disability. Model 1 was unadjusted model; All *P*-value of the likelihood ratio chi-square test of models of ADL disability, IADL disability, and comorbid ADL-IADL disability <0.001; The ratio of deviance statistic to degrees of freedom of models of ADL disability, IADL disability, and comorbid ADL-IADL disability were 0.592, 0.127, and 0.343, respectively. Model 2 adjusted for sex, age, ethnic, marital status, educational attainment, occupation, annual income, cerebrovascular disease, rheumatism, osteoarthropathy, smoking status, and alcohol consumption; All *p*-value of the likelihood ratio chi-square test of models of ADL disability, IADL disability, and comorbid ADL-IADL disability <0.001; The ratio of deviance statistic to degrees of freedom of models of ADL disability, IADL disability, and comorbid ADL-IADL disability were 0.553, 0.128, and 0.343, respectively. Model 3 further adjusted hand grip strength, anemia, total cholesterol, low density lipoprotein cholesterol, aspartate aminotransferase, alanine aminotransferase, serum creatinine, and uric acid; *p*-value of the likelihood ratio chi-square test of models of ADL disability, IADL disability, and comorbid ADL-IADL disability were <0.001, 0.008, and 0.001,respectively; The ratio of deviance statistic to degrees of freedom of models of ADL disability, IADL disability, and comorbid ADL-IADL disability were 0.554, 0.152, 0.343, and 0.394,respectively.

**Figure 2:**
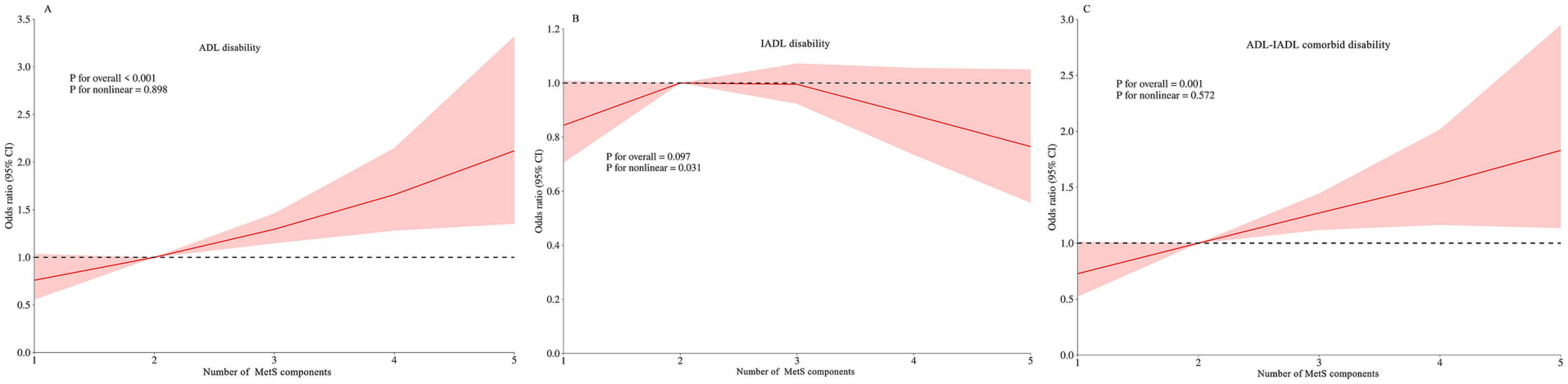
Restricted cubic spline of the linear trends between number of MetS component and functional disability. **(A)** the ADL disability risk with number of MetS component; **(B)** the IADL disability risk with number of MetS component; **(C)** the comorbid ADL-IADL disability risk with number of MetS component. Spline analyses were adjusted for sex, age, ethnic, marital status, educational attainment, occupation, annual income, cerebrovascular disease, rheumatism, osteoarthropathy, smoking status, alcohol consumption, hand grip strength, anemia, total cholesterol, low density lipoprotein cholesterol, aspartate aminotransferase, alanine aminotransferase, serum creatinine, and uric acid. MetS, metabolic syndrome; ADL, activities of daily living; IADL, instrumental activities of daily living.

Table 3 reveals significant dose–response relationships between the number of MetS components and functional disability. Each additional MetS component was associated with a 2% increased risk of ADL disability (PR for trend = 1.02, 95% CI: 1.01–1.03) and a 22% higher risk of comorbid ADL-IADL disability (PR for trend = 1.22, 95% CI: 1.10–1.35) in Model 3. These linear associations were corroborated by RCS analyses for all disability outcomes (Figure 2).

**Table 3:** Associations between number of MetS components and functional disability in older adults population.

Functional disability/Models	The number of MetS components [PR (95% CI)]					PR (95% CI) for trend
	0	1	2	3	≥4	
ADL disability						
Model 1	1.00 (Ref)	1.06 (1.01,1.10)*	1.07 (1.04,1.10)***	1.04 (1.01,1.06)**	1.02 (1.00,1.03)	1.02 (1.01,1.03)***
Model 2	1.00 (Ref)	1.04 (0.99,1.08)	1.06 (1.03,1.09)***	1.02 (1.00,1.05)*	1.00 (0.98,1.02)	1.02 (1.01,1.02)***
Model 3	1.00 (Ref)	1.04 (0.99,1.08)	1.07 (1.03,1.10)***	1.03 (1.01,1.05)**	1.00 (0.99,1.02)	1.02 (1.01,1.03)***
IADL disability						
Model 1	1.00 (Ref)	1.03 (0.97,1.09)	1.04 (1.00,1.08)	1.05 (1.02,1.09)*	1.03 (1.00,1.06)	1.01 (1.00,1.02)
Model 2	1.00 (Ref)	0.98 (0.93,1.03)	1.02 (0.98,1.05)	1.03 (1.00,1.06)	1.01 (0.98,1.04)	1.00 (0.99,1.01)
Model 3	1.00 (Ref)	0.96 (0.91,1.02)	1.01 (0.98,1.05)	1.03 (1.00,1.06)	1.01 (0.98,1.04)	1.00 (0.99,1.01)
Comorbid ADL-IADL disability						
Model 1	1.00 (Ref)	1.99 (1.19,3.33)**	2.24 (1.53,3.29)***	1.69 (1.19,2.42)**	1.37 (0.97,1.93)	1.23 (1.13,1.35)***
Model 2	1.00 (Ref)	1.58 (0.94,2.64)	1.96 (1.33,2.90)**	1.42 (1.00,2.03)	1.13 (0.80,1.61)	1.20 (1.10,1.32)***
Model 3	1.00 (Ref)	1.54 (0.85,2.78)	2.15 (1.43,3.25)***	1.57 (1.09,2.26)	1.22 (0.85,1.74)	1.22 (1.10,1.35)***

MetS, metabolic syndrome; ADL, activities of daily living; IADL, instrumental activities of daily living; PR, prevalence ratio; CI, confidence interval. Modified Poisson regression models were calculated associations between number of MetS components and functional disability. The bold values indicate statistically significant differences. *, *p*<0.05; **, *p*<0.01; ***, *p*<0.001. Model 1 was unadjusted model; All P-value of the likelihood ratio chi-square test of models of ADL disability, IADL disability, and comorbid ADL-IADL disability <0.001; The ratio of deviance statistic to degrees of freedom of models of ADL disability, IADL disability, and comorbid ADL-IADL disability were 0.595, 0.127, and 0.392, respectively. Model 2 adjusted for sex, age, ethnic, marital status, educational attainment, occupation, annual income, cerebrovascular disease, rheumatism, osteoarthropathy, smoking status, and alcohol consumption; All p-value of the likelihood ratio chi-square test of models of ADL disability, IADL disability, and comorbid ADL-IADL disability <0.001; The ratio of deviance statistic to degrees of freedom of models of ADL disability, IADL disability, and comorbid ADL-IADL disability were 0.546, 0.128, and 0.342, respectively. Model 3 further adjusted hand grip strength, anemia, total cholesterol, low density lipoprotein cholesterol, aspartate aminotransferase, alanine aminotransferase, serum creatinine, and uric acid; p-value of the likelihood ratio chi-square test of models of ADL disability, IADL disability, and comorbid ADL-IADL disability were <0.001, 0.001 and <0.001, respectively; The ratio of deviance statistic to degrees of freedom of models of ADL disability, IADL disability, and comorbid ADL-IADL disability were 0.558, 0.152, and 0.342, respectively.

Table 4 showed that abdominal obesity independently predicted a 3% increased ADL disability risk (PR = 1.03, 95% CI: 1.01–1.05) and a 36% higher comorbid ADL-IADL disability risk (PR = 1.36, 95% CI: 1.08–1.71). Conversely, reduced HDL-C levels were associated with a 4% lower IADL disability risk (PR = 0.96, 95% CI: 0.93–0.99) compared to normal HDL-C levels in fully adjusted models.

**Table 4:** Associations between individual components of MetS and functional disability in older adults population.

Functional disability/Models	Elevated blood pressure [PR (95% CI)]		Elevated fasting glucose [PR (95% CI)]		Elevated triglycerides [PR (95% CI)]		Reduced HDL cholesterol [PR (95% CI)]		Abdominal obesity [PR (95% CI)]	
	No	Yes	No	Yes	No	Yes	No	Yes	No	Yes
ADL disability										
Model1	1.00 (Ref)	1.02 (1.01,1.04)**	1.00 (Ref)	1.02 (1.00,1.04)	1.00 (Ref)	1.00 (0.98,1.02)	1.00 (Ref)	1.03 (1.00,1.06)*	1.00 (Ref)	1.02 (1.01,1.04)*
Model2	1.00 (Ref)	1.01 (0.99,1.02)	1.00 (Ref)	1.01 (0.99,1.03)	1.00 (Ref)	1.01 (0.99,1.03)	1.00 (Ref)	1.03 (1.00,1.05)*	1.00 (Ref)	1.03 (1.01,1.05)**
Model3	1.00 (Ref)	1.01 (0.99,1.03)	1.00 (Ref)	1.01 (0.99,1.03)	1.00 (Ref)	1.01 (0.98,1.03)	1.00 (Ref)	1.02 (1.00,1.05)	1.00 (Ref)	1.03 (1.01,1.05)**
IADL disability										
Model1	1.00 (Ref)	1.03 (1.00,1.05)*	1.00 (Ref)	1.01 (0.98,1.04)	1.00 (Ref)	0.96 (0.94,0.99)**	1.00 (Ref)	1.02 (0.99,1.06)	1.00 (Ref)	1.03 (1.00,1.05)*
Model2	1.00 (Ref)	1.01 (0.99,1.04)	1.00 (Ref)	1.01 (0.98,1.04)	1.00 (Ref)	0.99 (0.97,1.02)	1.00 (Ref)	0.97 (0.94,0.99)*	1.00 (Ref)	1.01 (0.99,1.03)
Model3	1.00 (Ref)	1.02 (0.99,1.04)	1.00 (Ref)	1.01 (0.98,1.04)	1.00 (Ref)	0.98 (0.95,1.02)	1.00 (Ref)	0.96 (0.93,0.99)*	1.00 (Ref)	1.01 (0.99,1.03)
Comorbid ADL-IADL disability										
Model1	1.00 (Ref)	1.33 (1.04,1.69)*	1.00 (Ref)	1.25 (0.97,1.62)	1.00 (Ref)	0.98 (0.75,1.27)	1.00 (Ref)	1.43 (1.09,1.88)*	1.00 (Ref)	1.27 (1.03,1.58)*
Model2	1.00 (Ref)	1.14 (0.91,1.44)	1.00 (Ref)	1.14 (0.89,1.45)	1.00 (Ref)	1.06 (0.83,1.36)	1.00 (Ref)	1.30 (1.01,1.69)*	1.00 (Ref)	1.34 (1.08,1.66)**
Model3	1.00 (Ref)	1.20 (0.94,1.53)	1.00 (Ref)	1.13 (0.87,1.46)	1.00 (Ref)	1.06 (0.79,1.43)	1.00 (Ref)	1.27 (0.94,1.71)	1.00 (Ref)	1.36 (1.08,1.71)**

MetS, metabolic syndrome; ADL, activities of daily living; IADL, instrumental activities of daily living; PR, prevalence ratio; CI, confidence interval. Modified Poisson regression models were calculated associations between individual components of MetS and functional disability. The bold values indicate statistically significant differences. *p<0.05; **p<0.01. Model 1 was unadjusted model; P-value of the likelihood ratio chi-square test of models of ADL disability, IADL disability, and comorbid ADL-IADL disability were <0.001, 0.371 and <0.001, respecyively; The ratio of deviance statistic to degrees of freedom of models of ADL disability, IADL disability, and comorbid ADL-IADL disability were 0.582, 0.151, and 0.388, respectively. Model 2 adjusted for sex, age, ethnic, marital status, educational attainment, occupation, annual income, cerebrovascular disease, rheumatism, osteoarthropathy, smoking status, and alcohol consumption; All p-value of the likelihood ratio chi-square test of models of ADL disability, IADL disability, and comorbid ADL-IADL disability <0.001; The ratio of deviance statistic to degrees of freedom of models of ADL disability, IADL disability, and comorbid ADL-IADL disability were 0.546, 0.127, and 0.312, respectively. Model 3 further adjusted hand grip strength, anemia, total cholesterol, low density lipoprotein cholesterol, aspartate aminotransferase, alanine aminotransferase, serum creatinine, and uric acid; All p-value of the likelihood ratio chi-square test of models of ADL disability, IADL disability, and comorbid ADL-IADL disability <0.001; The ratio of deviance statistic to degrees of freedom of models of ADL disability, IADL disability, and comorbid ADL-IADL disability were 0.545, 0.126, and 0.341, respectively.

### 3.4 Association of the combinations of MetS components with functional disability

As shown in Table 5, distinct risk patterns emerged for specific MetS component combinations in the fully adjusted model (Model 3). Compared to 0 MetS components, reduced HDL-C showed a 10% lower IADL disability risk (PR = 0.90, 95% CI: 0.84–0.96), elevated blood pressure with reduced HDL cholesterol demonstrated a 1.82-fold increased risk of comorbid ADL-IADL disability (95% CI: 1.06–3.14).

**Table 5:** Associations of different combinations of MetS components with functional disability in older adults population.

Components/combinations	ADL disability [PR (95% CI)]			IADL disability [PR (95% CI)]			Comorbid ADL-IADL disability [PR (95% CI)]		
	Model 1	Model 2	Model 3	Model 1	Model 2	Model 3	Model 1	Model 2	Model 3
One MetS component									
None	1.00 (Ref)	1.00 (Ref)	1.00 (Ref)	1.00 (Ref)	1.00 (Ref)	1.00 (Ref)	1.00 (Ref)	1.00 (Ref)	1.00 (Ref)
Elevated blood pressure	1.02 (1.00, 1.04)	1.00 (0.98, 1.02)	1.00 (0.98, 1.02)	1.03 (1.00, 1.07)*	1.02 (0.99, 1.05)	1.02 (0.99, 1.05)	1.40 (0.98, 2.00)	1.11 (0.77, 1.60)	1.05 (0.72, 1.52)
Elevated fasting glucose	1.01 (0.96, 1.06)	1.00 (0.95, 1.05)	1.00 (0.95, 1.05)	1.01 (0.93, 1.09)	1.01 (0.94, 1.09)	1.01 (0.94, 1.09)	1.09 (0.44, 2.69)	1.03 (0.43, 2.49)	0.92 (0.38, 2.28)
Elevated triglycerides	1.00 (0.95, 1.06)	0.99 (0.95, 1.04)	0.99 (0.95, 1.04)	0.98 (0.90, 1.06)	1.00 (0.93, 1.09)	1.00 (0.93, 1.09)	1.17 (0.43, 3.17)	0.94 (0.34, 2.58)	0.84 (0.32, 2.26)
Reduced HDL cholesterol	1.02 (0.96, 1.07)	1.00 (0.96, 1.05)	1.01 (0.96, 1.05)	0.96 (0.90, 1.04)	0.89 (0.84, 0.95)**	0.90 (0.84, 0.96)**	1.46 (0.67, 3.17)	1.16 (0.61, 2.22)	1.04 (0.53, 2.03)
Abdominal obesity	1.01 (0.97, 1.05)	1.01 (0.98, 1.05)	1.01 (0.97, 1.05)	1.07 (1.01, 1.14)*	1.05 (0.99, 1.10)	1.04 (0.98, 1.10)	1.28 (0.67, 2.45)	1.29 (0.69, 2.38)	1.08 (0.55, 2.14)
Two MetS components									
None	1.00 (Ref)	1.00 (Ref)	1.00 (Ref)	1.00 (Ref)	1.00 (Ref)	1.00 (Ref)	1.00 (Ref)	1.00 (Ref)	1.00 (Ref)
Elevated blood pressure + Reduced HDL cholesterol	1.07 (1.01, 1.13)*	1.05 (0.99, 1.11)	1.05 (0.99, 1.12)	1.11 (1.04, 1.19)**	1.03 (0.97, 1.10)	1.03 (0.96, 1.11)	2.50 (1.40, 4.43)**	1.83 (1.06, 3.16)*	1.82 (1.06, 3.14)*
Elevated blood pressure + Elevated triglycerides	1.01 (0.97, 1.05)	1.00 (0.96, 1.03)	1.01 (0.97, 1.05)	1.02 (0.97, 1.08)	1.04 (0.98, 1.09)	1.04 (0.98, 1.09)	1.13 (0.60, 2.12)	0.99 (0.55, 1.78)	1.04 (0.58, 1.88)
Elevated blood pressure + Elevated fasting glucose	1.04 (1.00, 1.08)	1.01 (0.97, 1.05)	1.02 (0.97, 1.07)	1.03 (0.97, 1.09)	1.02 (0.97, 1.07)	1.02 (0.96, 1.08)	1.69 (0.99, 2.88)	1.25 (0.75, 2.10)	1.30 (0.76, 2.20)
Abdominal obesity + Elevated blood pressure	1.04 (1.01, 1.07)**	1.03 (1.00, 1.06)	1.03 (1.00, 1.06)	1.06 (1.02, 1.10)**	1.02 (0.98, 1.06)	1.02 (0.98, 1.06)	1.71 (1.13, 2.59)*	1.44 (0.94, 2.23)	1.41 (0.91, 2.19)
Other combinations	1.03 (0.99, 1.08)	1.03 (0.99, 1.07)	1.03 (0.99, 1.07)	1.07 (1.00, 1.14)*	1.02 (0.96, 1.08)	1.02 (0.96, 1.08)	1.85 (1.01, 3.36)*	1.42 (0.84, 2.40)	1.45 (0.86, 2.46)
Three MetS components									
None	1.00 (Ref)	1.00 (Ref)	1.00 (Ref)	1.00 (Ref)	1.00 (Ref)	1.00 (Ref)	1.00 (Ref)	1.00 (Ref)	1.00 (Ref)
Elevated blood pressure + Elevated triglycerides + Elevated fasting glucose	1.04 (0.97, 1.12)	1.04 (0.96, 1.12)	1.05 (0.97, 1.13)	0.96 (0.88, 1.06)	0.98 (0.90, 1.07)	0.99 (0.91, 1.08)	1.35 (0.50, 3.65)	1.37 (0.51, 3.70)	1.65 (0.60, 4.54)
Abdominal obesity + Elevated blood pressure + Reduced HDL cholesterol	1.07 (1.00, 1.14)*	1.05 (0.98, 1.13)	1.05 (0.98, 1.13)	1.08 (1.00, 1.17)	1.03 (0.96, 1.10)	1.02 (0.95, 1.10)	1.87 (0.90, 3.85)	1.50 (0.69, 3.23)	1.56 (0.71, 3.40)
Abdominal obesity + Elevated blood pressure + Elevated fasting glucose	1.10 (1.03, 1.17)*	1.07 (1.01, 1.14)*	1.08 (1.01, 1.14)*	1.16 (1.09, 1.24)***	1.12 (1.05, 1.19)***	1.12 (1.05, 1.19)***	3.00 (1.76, 5.11)***	2.25 (1.30, 3.90)**	2.42 (1.37, 4.27)**
Abdominal obesity + Elevated blood pressure + Elevated triglycerides	1.04 (1.00, 1.09)*	1.04 (1.00, 1.09)	1.05 (1.00, 1.09)*	0.96 (0.91, 1.01)	0.97 (0.92, 1.02)	0.97 (0.92, 1.02)	1.87 (1.11, 3.16)*	1.75 (1.05, 2.92)*	1.91 (1.11, 3.27)*
Other combinations	1.09 (1.02, 1.17)*	1.06 (1.00, 1.13)	1.07 (1.00, 1.14)*	1.07 (0.99, 1.15)	1.00 (0.93, 1.07)	1.00 (0.93, 1.07)	3.05 (1.73, 5.38)***	2.22 (1.30, 3.79)**	2.39 (1.36, 4.23)**
Four MetS components									
None	1.00 (Ref)	1.00 (Ref)	1.00 (Ref)	1.00 (Ref)	1.00 (Ref)	1.00 (Ref)	1.00 (Ref)	1.00 (Ref)	1.00 (Ref)
Abdominal obesity + Elevated blood pressure + Elevated triglycerides + Reduced HDL cholesterol	1.07 (0.99, 1.17)	1.05 (0.97, 1.13)	1.05 (0.97,1.13)	1.05 (0.95, 1.16)	0.97 (0.88, 1.06)	0.96 (0.88,1.05)	2.31 (1.03, 5.18)*	1.56 (0.74, 3.27)	1.48 (0.69,3.19)
Abdominal obesity + Elevated blood pressure + Elevated triglycerides + Elevated fasting glucose	1.05 (0.99, 1.11)	1.02 (0.96, 1.08)	1.02 (0.96,1.08)	1.02 (0.94, 1.10)	1.01 (0.94, 1.08)	1.00 (0.93,1.08)	1.90 (0.95, 3.78)	1.17 (0.56, 2.43)	1.22 (0.60,2.50)
Other combinations	1.05 (0.96, 1.15)	1.04 (0.95, 1.13)	1.03 (0.94,1.13)	1.01 (0.90, 1.13)	0.97 (0.87, 1.09)	0.97 (0.86,1.08)	1.63 (0.53, 5.03)	1.38 (0.44, 4.30)	1.29 (0.40,4.10)

MetS, metabolic syndrome; ADL, activities of daily living; IADL, instrumental activities of daily living; PR, prevalence ratio; CI, confidence interval. Modified Poisson regression models were calculated associations between different combinations of MetS components of MetS and functional disability. The bold values indicate statistically significant differences. *, *p*<0.05; **p<0.01, ***, *p*<0.001. Model 1 was unadjusted model. In one MetS component, *P*-value of the likelihood ratio chi-square test of models of ADL disability, IADL disability, and comorbid ADL-IADL disability were <0.001, <0.001, and 0.001, respectively. In two MetS component, *P*-value of the likelihood ratio chi-square test of models of ADL disability, IADL disability, and comorbid ADL-IADL disability were <0.001, 0.796, and 0.027, respectively. In three MetS component, *p*-value of the likelihood ratio chi-square test of models of ADL disability, IADL disability, and comorbid ADL-IADL disability <0.001, 0.446, and 0.001, respectively. In four MetS component, *p*-value of the likelihood ratio chi-square test of models of ADL disability, IADL disability, and comorbid ADL-IADL disability were <0.001, 0.083, and <0.001, respectively. Model 2 adjusted for sex, age, ethnic, marital status, educational attainment, occupation, annual income. In one MetS component, all *p*-value of the likelihood ratio chi-square test of models of ADL disability, IADL disability, and comorbid ADL-IADL disability were <0.001. In two MetS component, all *p*-value of the likelihood ratio chi-square test of models of ADL disability, IADL disability, and comorbid ADL-IADL disability were <0.001. In three MetS component, *p*-value of the likelihood ratio chi-square test of models of ADL disability, IADL disability, and comorbid ADL-IADL disability were <0.001, 0.446, and 0.001, respectively. In four MetS component, *p*-value of the likelihood ratio chi-square test of models of ADL disability, IADL disability, and comorbid ADL-IADL disability were <0.001, 0.032, and <0.001, respectively. Model 3 further adjusted cerebrovascular disease, rheumatism, osteoarthropathy, smoking status, and alcohol consumption. In one MetS component, *p*-value of the likelihood ratio chi-square test of models of ADL disability, IADL disability, and comorbid ADL-IADL disability were <0.001, 0.005, and <0.001, respectively. In two MetS component, *p*-value of the likelihood ratio chi-square test of models of ADL disability, IADL disability, and comorbid ADL-IADL disability were <0.001, 0.001, and <0.001, respectively. In three MetS component, *p*-value of the likelihood ratio chi-square test of models of ADL disability, IADL disability, and comorbid ADL-IADL disability were <0.001, 0.008, and 0.001, respectively. In four MetS component, *p*-value of the likelihood ratio chi-square test of models of ADL disability, IADL disability, and comorbid ADL-IADL disability were <0.001, 0.016, and 0.001, respectively.

The combination of abdominal obesity, elevated blood pressure, and elevated FPG was associated with a 10% higher ADL disability risk (PR = 1.10, 95% CI: 1.01–1.14), 12% elevated IADL disability risk (PR = 1.12, 95% CI: 1.05–1.19), and 2.42-fold increased comorbid ADL-IADL disability risk (95% CI: 1.37–4.27). Abdominal obesity combined with elevated blood pressure and elevated TG exhibited a 1.91-fold greater ADL disability risk (95% CI: 1.11–3.27).

### 3.5 Non-linear trends of individual MetS components with functional disability

**Figure 3:**
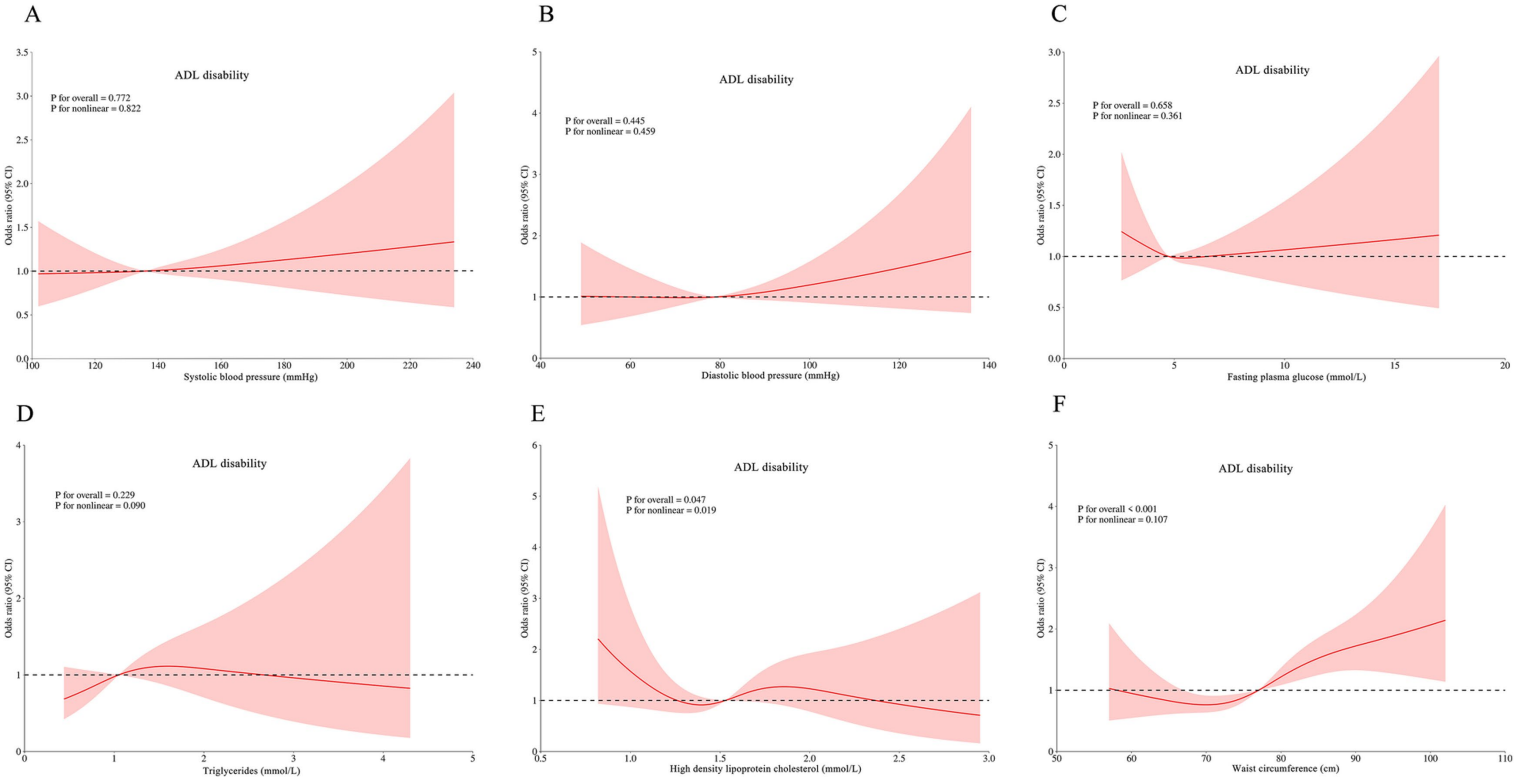
Restricted cubic spline of the linear trends between individual MetS component and ADL disability. **(A)** the ADL disability risk with systolic blood pressure; **(B)** the ADL disability risk with diastolic blood pressure; **(C)** the ADL disability risk with fasting blood glucose; **(D)** the ADL disability risk with triglycerides; **(E)** the ADL disability risk with high density lipoprotein cholesterol; **(F)** the ADL disability risk with waist circumference. Spline analyses were adjusted for sex, age, ethnic, marital status, educational attainment, occupation, annual income, cerebrovascular disease, rheumatism, osteoarthropathy, smoking status, alcohol consumption, hand grip strength, anemia, total cholesterol, low density lipoprotein cholesterol, aspartate aminotransferase, alanine aminotransferase, serum creatinine, and uric acid. MetS, metabolic syndrome; ADL, activities of daily living.

**Figure 4:**
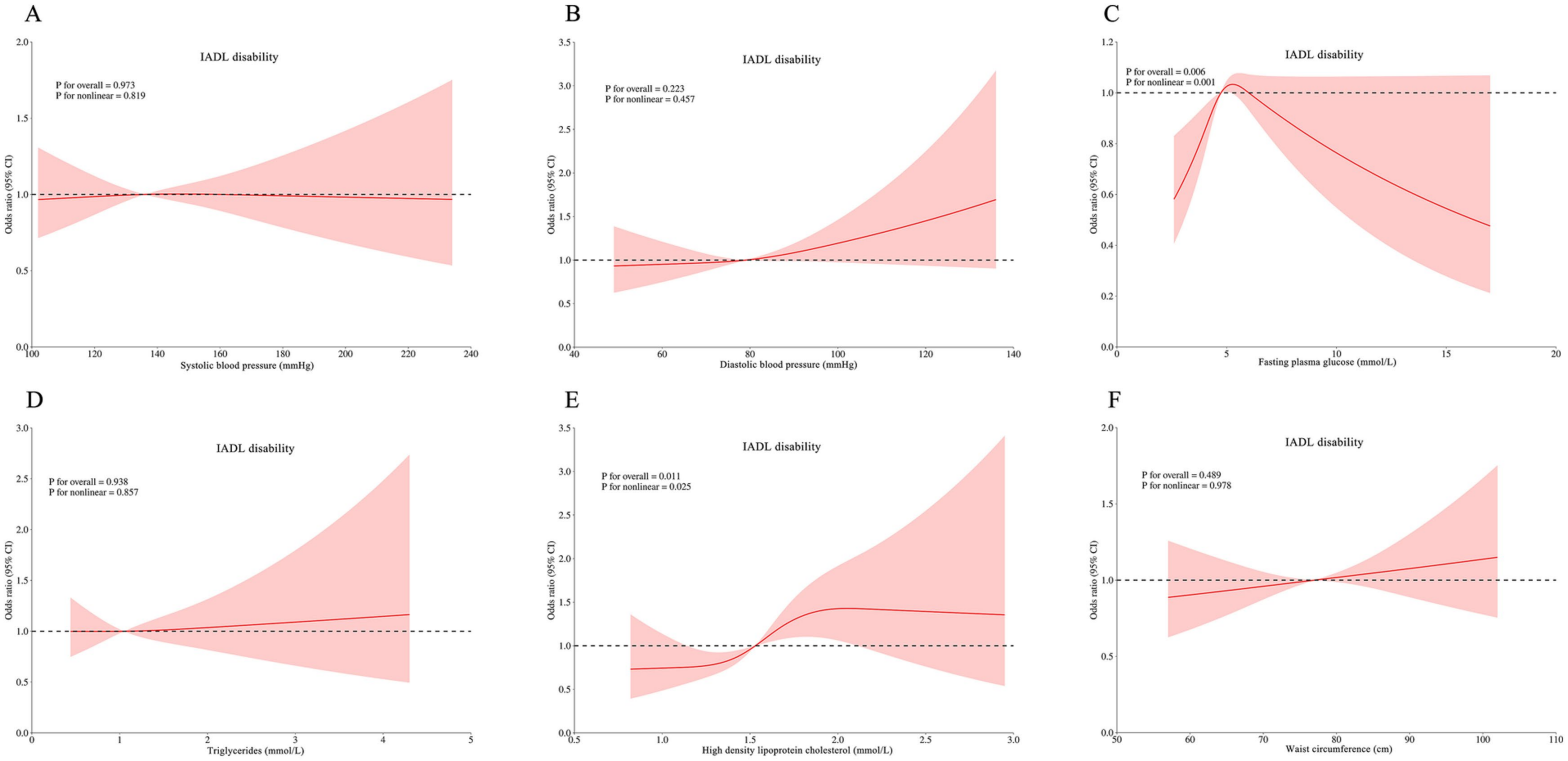
Restricted cubic spline of the linear trends between individual MetS component and IADL disability. **(A)** the IADL disability risk with systolic blood pressure; **(B)** the IADL disability risk with diastolic blood pressure; **(C)** the IADL disability risk with fasting blood glucose; **(D)** the IADL disability risk with triglycerides; **(E)** the IADL disability risk with high density lipoprotein cholesterol; **(F)** the IADL disability risk with waist circumference. Spline analyses were adjusted for sex, age, ethnic, marital status, educational attainment, occupation, annual income, cerebrovascular disease, rheumatism, osteoarthropathy, smoking status, alcohol consumption, hand grip strength, anemia, total cholesterol, low density lipoprotein cholesterol, aspartate aminotransferase, alanine aminotransferase, serum creatinine, and uric acid. MetS, metabolic syndrome; instrumental activities of daily living. MetS, metabolic syndrome; IADL, instrumental activities of daily living.

**Figure 5:**
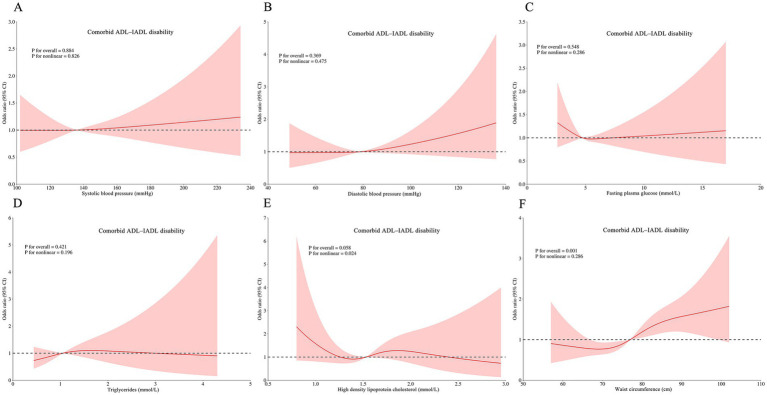
Restricted cubic spline of the linear trends between individual MetS component and comorbid ADLIADL disability. **(A)** the comorbid ADL-IADL disability risk with systolic blood pressure; **(B)** the comorbid ADL-IADL disability risk with diastolic blood pressure; **(C)** the comorbid ADL-IADL disability risk with fasting blood glucose; **(D)** the comorbid ADL-IADL disability risk with triglycerides; **(E)** the comorbid ADL-IADL disability risk with high density lipoprotein cholesterol; **(F)** the comorbid ADL-IADL disability risk with waist circumference. Spline analyses were adjusted for sex, age, ethnic, marital status, educational attainment, occupation, annual income, cerebrovascular disease, rheumatism, osteoarthropathy, smoking status, alcohol consumption, hand grip strength, anemia, total cholesterol, low density lipoprotein cholesterol, aspartate aminotransferase, alanine aminotransferase, serum creatinine, and uric acid. MetS, metabolic syndrome; ADL, activities of daily living; IADL, instrumental activities of daily living.

RCS analyses revealed complex dose–response relationships between continuous metabolic parameters and disability outcomes after multivariable adjustment. Three key patterns emerged: HDL-C was associated with ADL disability (*p* for non-linear = 0.019; Figure 3E), IADL disability (*P* for non-linear = 0.025; Figure 4E), and comorbid ADL-IADL disability (*p* for non-linear = 0.024; Figure 5E); A non-linear trend was observed in the relationship between FPG and IADL disability (*p* for non-linear = 0.001; Figure 4C); Additionally, WC exhibited linear relationships with ADL disability (*p* for overall<0.001; Figure 3F) and comorbid ADL-IADL disability (*p* for overall = 0.001; Figure 5F). Additionally, SBP, DBP, FBG, TG, and WC did not show non-linear or linear trend with ADL disability (all *p* for overall and *p* for non-linear >0.05; Figures 3A–D); SBP, DBP, TG, and WC did not display on-linear or linear trend with IADL disability (all *p* for overall and *p* for non-linear >0.05; Figures 4A,B,D,F); SBP, DBP, FBG, and TG did not show non-linear or linear trend with comorbid ADL-IADL disability (all *p* for overall and *p* for non-linear >0.05; Figures 5A–D).

### 3.6 Stratified and sensitivity analyses

The stratified analyses demonstrated several key findings (Supplementary Tables S5–13): (1) Across most subgroups of older adults, robust associations between MetS and both ADL disability and comorbid ADL-IADL disability were observed; ADL and comorbid ADL-IADL disability risks progressively increased with increasing number of MetS components, abdominal obesity had a stronger association with ADL disability and comorbid ADL-IADL disability, and reduced HDL-C had a robust association with IADL disability; (2) no significant association emerged between MetS and IADL disability in any subgroup analysis; and (3) IADL disability risk did not increase with increasing number of MetS components in any subgroup analysis.

Interaction analyses revealed several important findings (Supplementary Tables S14–22): (1) the effect of MetS on both ADL disability and comorbid ADL-IADL disability was stronger in older adults with anemia; and (2) in adults aged ≥70 years, the effects of MetS on IADL disability, reduced HDL-C on ADL/IADL/ADL-IADL disabilities, and abdominal obesity on IADL disability were stronger.

The correlations of MetS, number of MetS components, individual MetS components, and combinations of MetS components with ADL disability, IADL disability, and comorbid ADL-IADL disability remained similar when analyzed using binary logistic regression models (Supplementary Tables S23–26). The association of combinations of MetS components with ADL disability, IADL disability, and comorbid ADL-IADL disability was still robust in the Zhuang ethnicity population (Supplementary Table S27), farmers (Supplementary Table S28), and non-smokers (Supplementary Table S29).

## 4 Discussion

The current study found that MetS was significantly associated with ADL disability risk rather than IADL disability risk. The risk of ADL disability showed a progressive increase with an increasing number of MetS components. Further analysis revealed several significant findings: (1) abdominal obesity increased the risk of ADL disability, (2) reduced HDL cholesterol decreased the risk of IADL disability, and (3) the combination of abdominal obesity, elevated blood pressure, and elevated fasting glucose increased both ADL and IADL disability risks. Our findings have brought a nuanced perspective to the fore on how individual MetS components and combinations of MetS components influence functional disability, offering important implications for public health strategies.

In present study, our results suggested that MetS was significantly associated with ADL disability but not IADL disability. This finding was in accordance with previous longitudinal and cross-sectional studies (17, 19, 38, 39). Inversely, two other studies suggested that it was significantly associated with IADL disability (22, 23). In this study, ADL disability increased with an increase in the number of MetS components, rather than IADL disability. This finding is inconsistent with those of other studies. A 7-year multicenter longitudinal study found that IADL disability increased with the number of MetS components, rather than with ADL disability (21). Another study suggested that the number of MetS components is not associated with ADL or IADL disability (23). Collectively, these differences can be explained in several ways. First, there were different definitions of MetS (definition from IDF 2005, definition from ATP III 2005, definition from JIS 2009, and so on). Second, tools of evaluating physical function were different, such as some studies adopted complete scale (Barthel index, Katz index, and physical sell-maintenance scale). Finally, the approaches to scoring for the same scale may be different (the primary approach and the modified approach). The mechanism of MetS with ADL disability might be atherogenesis induced by intravascular endothelial injury and a reduction in muscle mass. The pathogenesis of MetS involves multiple genetic and acquired entities that fall under the umbrella of chronic low-grade inflammation and insulin resistance (40). Individuals with MetS exhibit elevated levels of pro-inflammatory mediators, including adiponectin, leptin, high-sensitivity C-reactive protein (CRP), interleukin (IL)-1, IL-6, IL-8, fibrinogen, monocytic toll-like receptors 2 and 4, and tumor necrosis factor-alpha (TNF-*α*), compared to those without MetS (41, 42). Chronic inflammation involves a coordinated interaction between the vascular endothelium and circulating immune cells, which promotes arterial stiffening and thickening (39). These vascular changes drive atherogenesis, which may contribute to mobility impairments (e.g., walking disability) (43, 44). Furthermore, insulin and insulin-like growth factor 1 maintain muscle mass by stimulating protein synthesis and suppressing proteolysis (45, 46). Insulin resistance exacerbates muscle degradation via dysregulation of insulin and insulin-like growth factor 1 signaling. Additionally, insulin resistance activates inflammatory cascades that synergistically promote atherogenesis (47), leading to ADL disability. We also confirmed that ADL disability risk was positively associated with the number of MetS components. This association can be explained by the synergistic and additive effects of MetS components on ADL disability. Further studies are needed to understand the complex relationship between MetS components and ADL disabilities.

Our study demonstrated an inverse association between reduced HDL-C levels and IADL disability after comprehensive adjustment for potential confounders. This observed relationship may be mechanistically explained through the concept of inflammaging, a chronic low-grade inflammatory state recognized as one of the 12 hallmarks of aging (48). Inflammaging manifests through both systemic biological changes and localized pathological phenotypes, potentially mediating functional decline (49). Emerging evidence supports the role of the inflammatory pathway in functional disabilities. Elevated CRP levels have consistently been associated with IADL impairment (50). Notably, a cross-sectional study identified significant associations between inflammatory biomarkers (erythrocyte sedimentation rate, CRP, and IL-6) and IADL limitations, specifically in individuals with low HDL cholesterol concentration (51). The dualistic nature of HDL inflammatory modulation suggests a possible biological mechanism. Current research indicates that HDL particles exhibit context-dependent anti-inflammatory or pro-inflammatory properties mediated by structural modifications (including apolipoprotein composition and lipid cargo), cellular cholesterol trafficking dynamics, and signaling pathway interactions (52). Under physiological conditions, HDL demonstrates anti-inflammatory and antioxidant capacities via multiple pathways (53, 54).

However, chronic inflammatory states induce HDL dysfunction through a proposed threshold mechanism that transforms these lipoprotein complexes into pro-inflammatory mediators (52). This phenotypic conversion appears to be driven by excessive cellular cholesterol depletion, which activates the inositol-requiring enzyme 1α/apoptosis signal-regulating kinase 1/p38 mitogen-activated protein kinase signaling cascade (53). The subsequent induction of endoplasmic reticulum stress responses creates a pro-inflammatory feedback loop. Our findings indicate that age-related inflammation may surpass the critical threshold required to subvert the HDL-C protective function, thereby potentiating inflammatory cascades through multiple mechanisms. This pathophysiological shift could explain the paradoxical association between HDL cholesterol levels and the functional decline observed in aging populations. Additionally, the inverse relationship between reduced HDL-C and IADL disability may be due to confounding or reverse causation from cross-sectional design. Therefore, conducting longitudinal research on this topic is essential in the future.

In the current study, abdominal obesity and a combination of abdominal obesity, elevated blood pressure, and elevated fasting glucose levels were associated with ADL disability. To the best of our knowledge, this study is the first to examine the association between combinations of the three MetS components and functional disability. Our findings indicate that MetS components have pronounced synergistic or additive effects on ADL impairment. However, the exact biological mechanism remains unclear. A longitudinal study from 2011 to 2018 demonstrated that compared with middle-aged and older adults without MetS, the risk for ADL impairments was 3.51 times higher (95%CI: 1.66–7.43) for those with hypertension complicated with diabetes (30). To the best of our knowledge, this investigation represents the first systematic examination of ADL impairment risks associated with combinations of ≥3 MetS components. The longitudinal association between abdominal obesity and ADL disability incidence aligns with previous cohort studies (55, 56), whereas cross-sectional evidence further substantiates WC as a robust predictor of mobility limitations (28). The pathophysiological mechanisms linking abdominal obesity to functional decline appear to be multifactorial. Age-related changes in body composition, characterized by sarcopenic obesity and the co-occurrence of muscle mass depletion and visceral fat accumulation, create a biological substrate for functional impairment (57). Key possible mechanisms include: (1) Myosteatosis: Intramuscular lipid infiltration alters muscle architecture and contractile function (58, 59); (2) pro-inflammatory signaling: adipose tissue-driven overexpression of circulating cytokines (such as TNF-*α*, TNF-*β*, and IL-6) promotes muscle catabolism and inhibits insulin-mediated anabolic processes (60–63); (3) neuromuscular dysregulation: adipose-induced overexpression of circulating cytokines hinders the insulin-mediated repair mechanisms of motor neurons (62, 63). Longitudinal and cross-sectional analyses have demonstrated that individuals with hypertension experience greater ADL limitations than those without hypertension (24, 64–66). Moreover, a systematic review suggested that antihypertensive therapy is associated with a lower risk of ADL impairment than control therapy (67). The vascular-inflammatory axis appears central to this relationship, involving the following: (1) complement system activation and inflammasome-mediated immune cell phenotypic changes (68, 69), and (2) target organ damage via overt (stroke, myocardial infarction) and subclinical (covert stroke, sarcopenia) vascular events (70, 71). Numerous studies have shown that people with diabetes or impaired glucose tolerance have an increased risk of ADL disability compared to those without diabetes or with normal glucose tolerance (24, 70, 72, 73). An earlier meta-analysis estimating the magnitude of the association between diabetes and disability found that diabetes was associated with increased odds of difficulties with ADLs compared to no diabetes (74). The mechanisms underlying diabetes-related disabilities may be linked to skeletal muscle strength, muscle quality, and complications of diabetes. Diabetes mellitus and impaired glucose homeostasis confer a substantial risk of ADL disability through multiple pathways: (1) Systemic inflammation-induced muscle quality deterioration: Chronic hyperglycemia induces systemic inflammatory responses, promoting the development of age-related sarcopenia through sustained pro-catabolic signaling, ultimately leading to progressive mobility impairment and functional disability (75–81); (2) Imbalance of muscle protein degradation and synthesis resulting in decreased muscle mass: Insulin resistance disrupts IGF-1-mediated protein synthesis and catabolism, as insulin and insulin-like growth factor 1 are responsible not only for glucose uptake but also for maintaining muscle mass via the stimulation of muscle protein synthesis and inhibition of muscle protein breakdown (70, 82); (3) Neurological complications: The progression of diabetic neuropathy causes muscle weakness and sensorimotor deficits, which are closely associated with limitations in performing daily activities (83, 84). Taken together, healthcare providers should pay more attention to individuals with a combination of abdominal obesity, hypertension, and hyperglycemia in the MetS population, because these components may represent modifiable targets for ADL disability risk reduction.

In the present study, we found that a significant combination of abdominal obesity, elevated blood pressure, and elevated fasting glucose levels was associated with IADL disability. To the best of our knowledge, this is the first study to assess the association between this combination and IADL impairment. Our results demonstrate that the components of MetS have a significant combined impact on IADL impairment. However, the mechanisms underlying this collaboration remain unclear. Prior evidence indicates that individuals with diabetes not only suffer from IADL disability earlier than those without diabetes, but also show a higher incidence of IADL disability (72, 85, 86), a finding corroborated by meta-analytic data showing that diabetes confers 1.69-fold increased odds of IADL difficulties (74). Additionally, an earlier meta-analysis estimating the magnitude of the association between diabetes and disability showed that diabetes was associated with increased odds of difficulties with IADL compared to no diabetes. Similarly, the Atherosclerosis Risk in Communities study further substantiated this relationship, demonstrating a 1.82-fold elevated risk of IADL disability in diabetic populations (87). Mechanistically, chronic hyperglycemia may drive IADL disability through skeletal muscle degradation, as elevated glucose levels promote protein catabolism and proteolysis (79), leading to diminished muscle mass and strength, both of which are established predictors of IADL dependence (88, 89). Notably, abdominal obesity exacerbates functional decline via distinct pathways. Longitudinal analyses of 3,374 participants from the English Longitudinal Study of Aging and 1,040 subjects from the Brazilian Health Study revealed that isolated abdominal obesity accelerates IADL disability progression by 37% compared to non-abdominally obese/non-dynapenic individuals (90). Similarly, other studies have demonstrated that higher WC is associated with greater IADL disability (20, 91). This association appears to be mediated through three synergistic mechanisms: (1) visceral adipose-triggered systemic inflammation activating ubiquitin-proteasome pathways (62, 92) and (2) fatty infiltration of muscles reducing muscle strength via inflammatory and endocrine mechanisms (93, 94). Hypertension further compounds IADL limitations, as demonstrated in a cohort of 3,046 older Mexican Americans, where hypertensive individuals exhibited a 23% faster annual progression of IADL restrictions after multivariate adjustment (64). This relationship may involve mechanisms of inflammation. It is generally accepted that systemic inflammation is involved in the pathogenesis of hypertension (39). Hypertension correlates with IADL disability through the effects of inflammatory biomarkers on muscle strength and mass (50, 51). Taken together, the public health implications of this finding emphasize the importance of a comprehensive approach to IADL disability prevention. Abdominal obesity, elevated blood pressure, and elevated fasting glucose levels are modifiable risk factors, and a considerable proportion of functional disabilities may be prevented if one or two MetS components are reduced in older adults via adequate pharmacological and health education interventions.

Despite the key insights uncovered, we acknowledge the limitations of this study. First, our study was not able to establish causalities of MetS status, number of MetS components, and combinations of MetS components with functional disability, as residual confounding factors cannot be completely ruled out. Second, although the ADL/IADL information was captured using validated scales, it is still subjective and known to lead to possible measurement bias. Third, the observed interconnectedness between chronic noncommunicable diseases may not be fully considered, and the temporal order of noncommunicable disease incidence may yield a residual bias. The participants in our study resided in a remote county in the mountainous area of Guangxi, which resulted in poor access to medical and health services. Therefore, some older adults may not be aware of chronic diseases, even if they have them. Fourth, because the study sample was of more than 85% Zhuang ethnicity, whether the findings can be generalized to other ethnic groups requires further investigation. Fifth, the sample size did not allow us to conduct subgroup and interaction analyses of the associations between combinations of MetS components and functional disability. Nevertheless, despite the limitations mentioned above, our results highlight the importance of steady associations of MetS, the number of MetS components, and combinations of MetS components with ADL disability, IADL disability, and comorbid ADL-IADL disability after adjusting for different confounders and using different analysis methods, subgroup analysis, and interaction analysis.

## 5 Conclusion

In summary, MetS and the number of MetS components were positively correlated with ADL disability risk and comorbid ADL-IADL disability risk. Reduced HDL cholesterol levels showed a strong negative association with the risk of IADL disability. The combination of abdominal obesity, elevated blood pressure, and elevated fasting glucose levels was strongly associated with ADL disability risk, IADL disability risk, and comorbid ADL-IADL disability risk. Our findings provide potential paths for preventing or at least delaying ADL and IADL disability processes in the management of the MetS population to promote independent living for a longer and better quality of life. Further longitudinal cohort studies and interventions are needed to investigate causality and mechanisms.

## Data Availability

The raw data supporting the conclusions of this article will be made available by the authors, without undue reservation.
