# Supplementary material for: Association of metabolic syndrome components and their combinations with functional disability among older adults in a longevity-associated ethnic minority region of Southwest China
Source: Front Public Health. 2025 Jul 11;13:1635390. doi: 10.3389/fpubh.2025.1635390 (PMC12289597; doi:10.3389/fpubh.2025.1635390)
Supplement: Supplementary file 1 [file Table_1.docx]

Supplementary Material

# Supplementary Supplementary Tables

**Supplementary Table S1.** The combinations of MetS components

**Supplementary Table S2.** The groups and definitions of covariates

**Supplementary Table S3.** The missing data of all variables

**Supplementary Table S4.** The functional disability of different combinations of MetS components

**Supplementary Table S5.** Subgroup analysis of associations of MetS with ADL disability

**Supplementary Table S6.** Subgroup analysis of associations of MetS with IADL disability

**Supplementary Table S7.** Subgroup analysis of associations of MetS with comorbid ADL–IADL disability

**Supplementary Table S8.** Subgroup analysis of associations between the number of MetS components with ADL disability

**Supplementary Table S9.** Subgroup analysis of associations between the number of MetS components with IADL disability

**Supplementary Table S10.** Subgroup analysis of associations between the number of MetS components with comorbid ADL–IADL disability

**Supplementary Table S11.** Subgroup analysis of associations between individual MetS components and ADL disability

**Supplementary Table S12.** Subgroup analysis of associations between individual MetS components and IADL disability

**Supplementary Table S13.** Subgroup analysis of associations between individual MetS components and comorbid ADL–IADL disability

**Supplementary Table S14.** Interaction effects of covariates and MetS on functional disability

**Supplementary Table S15.** Interaction effects of covariates and number of MetS components on ADL disability

**Supplementary Table S16.** Interaction effects of covariates and number of MetS components on IADL disability

**Supplementary Table S17.** Interaction effects of covariates and number of MetS components on comorbid ADL­IADL disability

**Supplementary Table S18.** Interaction effects of covariates and elevated blood pressure on functional disability

**Supplementary Table S19.** Interaction effects of covariates and elevated blood glucose on functional disability

**Supplementary Table S20.** Interaction effects of covariates and elevated triglycerides on functional disability

**Supplementary Table S21.** Interaction effects of covariates and reduced high density lipoprotein cholesterol on functional disability

**Supplementary Table S22.** Interaction effects of covariates and reduced waist circumference on functional disability

**Supplementary Table S23.** Associations between metabolic syndrome and functional disability analyzed using binary logistic regression analysis

**Supplementary Table S24.** Associations between the number of MetS components and functional disability analyzed using binary logistic regression analysis

**Supplementary Table S25.** Associations between individual MetS components and functional disability analyzed using binary logistic regression analysis

**Supplementary Table S26.** Associations between various combinations of MetS components and functional disability analyzed using binary logistic regression analysis

**Supplementary Table S27.** Associations between various combinations of MetS components and functional disability in Zhuang ethnic population

**Supplementary Table S28.** Associations between various combinations of MetS components and functional disability in farmer

**Supplementary Table S29.** Associations between various combinations of MetS components and functional disability in non–drinking population

Abbreviation: MetS, metabolic syndrome; ADL, Activities of daily living; IADL, instrumental activities of daily living.

**Supplementary Table S1.** The combinations of MetS components

| Components/combinations of metabolic syndrome | Frequency | Percentage (%) |
| --- | --- | --- |
| **One metabolic syndrome component (n=1780)** |  |  |
| Elevated blood pressure | 1361 | 76.46 |
| Abdominal obesity | 168 | 9.44 |
| Elevated fasting glucose | 90 | 5.06 |
| Reduced HDL cholesterol | 94 | 5.28 |
| Elevated triglycerides | 67 | 3.76 |
| **Combinations of two metabolic syndrome components (n=1147)** |  |  |
| Abdominal obesity + Elevated blood pressure | 482 | 42.02 |
| Elevated blood pressure + Elevated fasting glucose | 209 | 18.22 |
| Elevated blood pressure + Elevated triglycerides | 208 | 18.13 |
| Elevated blood pressure + Reduced HDL cholesterol | 110 | 9.59 |
| Other combinations |  |  |
| Abdominal obesity + Reduced HDL cholesterol | 42 | 3.66 |
| Abdominal obesity + Elevated triglycerides | 32 | 2.79 |
| Abdominal obesity + Elevated fasting glucose | 29 | 2.53 |
| Elevated triglycerides + Elevated fasting glucose | 13 | 1.13 |
| Elevated triglycerides + Reduced HDL cholesterol | 12 | 1.05 |
| Elevated fasting glucose + Reduced HDL cholesterol | 10 | 0.87 |
| **Combinations of three metabolic syndrome components (n=542)** |  |  |
| Abdominal obesity + Elevated blood pressure + Elevated triglycerides | 199 | 36.72 |
| Abdominal obesity + Elevated blood pressure + Elevated fasting glucose | 111 | 20.48 |
| Abdominal obesity + Elevated blood pressure + Reduced HDL cholesterol | 84 | 15.50 |
| Elevated blood pressure + Elevated triglycerides + Elevated fasting glucose | 58 | 10.70 |
| Other combinations |  |  |
| Elevated blood pressure + Elevated triglycerides + Reduced HDL cholesterol | 30 | 5.54 |
| Elevated blood pressure + Elevated fasting glucose + Reduced HDL cholesterol | 22 | 4.06 |
| Abdominal obesity + Elevated triglycerides + Reduced HDL cholesterol | 14 | 2.58 |
| Abdominal obesity + Elevated triglycerides + Elevated fasting glucose | 12 | 2.21 |
| Abdominal obesity + Elevated fasting glucose + Reduced HDL cholesterol | 9 | 1.66 |
| Elevated triglycerides + Elevated fasting glucose + Reduced HDL cholesterol | 3 | 0.55 |
| **Combinations of four metabolic syndrome components (n=180)** |  |  |
| Abdominal obesity + Elevated blood pressure + Elevated fasting glucose + Elevated triglycerides | 93 | 51.67 |
| Abdominal obesity + Elevated blood pressure + Elevated triglycerides + Reduced HDL cholesterol | 51 | 28.33 |
| Other combinations |  |  |
| Abdominal obesity + Elevated blood pressure + Elevated fasting glucose + Reduced HDL cholesterol | 29 | 16.11 |
| Elevated blood pressure + Elevated triglycerides + Elevated fasting glucose + Reduced HDL cholesterol | 5 | 2.78 |
| Abdominal obesity + Elevated triglycerides + Elevated fasting glucose + Reduced HDL cholesterol | 2 | 1.11 |
| **Combination of five metabolic syndrome components (n=17)** |  |  |
| Abdominal obesity + Elevated blood pressure + Elevated triglycerides + Elevated fasting glucose + Reduced HDL cholesterol | 17 | 100.00 |

**Abbreviations:** HDL, high–density lipoprotein.

**Supplementary Table S2.** The groups and definitions of covariates

| Covariates | Groups | Definition |
| --- | --- | --- |
| **Sociodemographic variables** | | |
| Age | 60–69 years | – |
|  | ≥70 years | – |
| Gender | male | – |
|  | female | – |
| Ethnicity | Zhuang | – |
|  | Non–Zhuang | including ethnicity of Han, Yao, Miao and so on. |
| Marital status | single | including divorced, widowed, single |
|  | partnered | married and partnered |
| Educational attainment | less than primary school | – |
|  | primary school | – |
|  | high school and above | including high school, undergraduate, graduate, and so on |
| Occupation | farmer | – |
|  | non–farmer | Other occupations other than farmers |
| Annual income | ＜10000 renminbi | – |
|  | 10000–29999 renminbi | – |
|  | ≥30000 renminbi | – |
| **Lifestyle factors** |  | Participants who had always smoked ≥1 cigarette per day over the past 6 months were defined as current smokers |
| Smoking status | no | ex–smokers and nonsmokers (participants who had quit smoking at the time of the interview were defined as ex–smokers;  those who never smoked in lifetime were defined as nonsmokers) |
|  | yes | current smokers (participants who had always smoked ≥1 cigarette per day over the past 6 months were defined as current smokers) |
| Alcohol consumption | no | nondrinkers and ex–drinkers (participants who had quit drinking at the time of the survey were defined as ex–drinkers;  those who never drank alcohol in their lifetime were defined as nondrinkers) |
|  | yes | current drinkers (participants who had always smoked ≥1 cigarette per day over the past 6 months were defined as current smokers) |
| **Chronic diseases** |  | the history of chronic diseases was obtained by asking participants if |
| Cerebrovascular disease | no | participants who had not been diagnosed by a doctor with cerebrovascular disease |
|  | yes | participants who had been diagnosed by a doctor with cerebrovascular disease |
| Rheumatism | no | participants who had not been diagnosed by a doctor with rheumatism |
|  | yes | participants who had been diagnosed by a doctor with rheumatism |
| Osteoarthropathy | no | participants who had not been diagnosed by a doctor with osteoarthropathy |
|  | yes | participants who had been diagnosed by a doctor with osteoarthropathy |
| **Physical examination indicators** | | |
| Hand grip strength | low | male:﹤28kg, female:＜18kg |
|  | normal | male:≥28kg, female:≥18kg |
| Anemia | no | male: hemoglobin＜120 g/L, female: hemoglobin＜110 g/L |
|  | yes | male: hemoglobin≥120 g/L, female: hemoglobin≥110 g/L |
| Total cholesterol | normal | ＜5.2mmol/L |
|  | high | ≥5.2mmol/L |
| low density lipoprotein cholesterol | normal | ＜3.4mmol/L |
|  | high | ≥3.4mmol/L |
| Aspartate aminotransferase | normal | ≤35U/L |
|  | high | ＞35U/L |
| Alanine aminotransferase | normal | ≤40U/L |
|  | high | ＞40U/L |
| Serum creatinine | normal | male:53–133μmol/L, female: 44–133μmol/L |
|  | low | male:＜53μmol/L, female:＜44μmol/L |
| Uric acid | normal | ≤ 420 μmol/L |
|  | high | ＞420 μmol/L |

**Supplementary Table S3.** The missing data of all variables

| Variables | Valid [n (%)] | Missing [n (%)] |
| --- | --- | --- |
| Age | 4450 (100.00) | 0 |
| Gender | 4450 (100.00) | 0 |
| Cerebrovascular disease | 4450 (100.00) | 0 |
| Total cholesterol | 4450 (100.00) | 0 |
| Uric acid | 4450 (100.00) | 0 |
| Fasting blood glucose | 4450 (100.00) | 0 |
| Triglyceride | 4450 (100.00) | 0 |
| High density lipoprotein cholesterol | 4450 (100.00) | 0 |
| Waist circumference | 4450 (100.00) | 0 |
| Basic activities of daily living | 4450 (100.00) | 0 |
| Instrumental activities of daily living | 4450 (100.00) | 0 |
| Systolic blood pressure | 4449 (99.98) | 1 (0.02) |
| Diastolic blood pressure | 4449 (99.98) | 1 (0.02) |
| Osteoarthropathy | 4449 (99.98) | 1 (0.02) |
| Ethnic | 4447 (99.93) | 3 (0.07) |
| Rheumatism | 4447 (99.93) | 3 (0.07) |
| Smoking status | 4445 (99.89) | 5 (0.11) |
| Marital status | 4443 (99.84) | 7 (0.16) |
| Education attainment | 4440 (99.78) | 10 (0.22) |
| Anemia | 4437 (99.71) | 13 (0.29) |
| Hang grip stength | 4434 (99.64) | 16 (0.36) |
| Aspartate aminotransferasee | 4428 (99.51) | 22 (0.49) |
| Alanine aminotransferasee | 4428 (99.51) | 22 (0.49) |
| Serum creatinine | 4428 (99.51) | 22 (0.49) |
| Alcohol consumption | 4426 (99.46) | 24 (0.54) |
| Occupation | 4420 (99.33) | 30 (0.67) |
| Annual income | 4407 (99.03) | 43 (0.97) |
| Low density lipoprotein cholesterol | 4289 (96.38) | 161 (3.62) |

**Supplementary Table S4**. The functional disability according to different combinations of MetS components

| Components/combinations | Total | ADL | | IADL | | Comorbid ADL­IADL | |
| --- | --- | --- | --- | --- | --- | --- | --- |
|  |  | Disability [N (%)] | *P* value | Disability [N (%)] | *P* value | Disability [N (%)] | *P* value |
| **One MetS Component (n = 1780)** |  |  | 0.986 |  | 0.102 |  | 0.970 |
| Abdominal obesity | 168 | 12 (7.14) |  | 61 (36.31) |  | 11 (6.55) |  |
| Elevated blood pressure | 1361 | 103 (7.57) |  | 427 (31.37) |  | 97 (7.13) |  |
| Elevated triglycerides | 67 | 4 (5.97) |  | 16 (23.88) |  | 4 (5.97) |  |
| Elevated fasting glucose | 90 | 6 (6.67) |  | 25 (27.78) |  | 5 (5.56) |  |
| Reduced HDL cholesterol | 94 | 7 (7.45) |  | 21 (22.34) |  | 7 (7.45) |  |
| **Two MetS Components (n = 1147)** |  |  | 0.493 |  | 0.275 |  | 0.329 |
| Abdominal obesity  + Elevated blood pressure | 482 | 49 (10.17) |  | 165 (34.23) |  | 42 (8.71) |  |
| Elevated blood pressure + Elevated fasting glucose | 209 | 20 (9.57) |  | 64 (30.62) |  | 18 (8.61) |  |
| Elevated blood pressure + Elevated triglycerides | 208 | 14 (6.73) |  | 62 (29.81) |  | 12 (5.77) |  |
| Elevated blood pressure + Reduced HDL cholesterol | 110 | 14 (12.73) |  | 45 (40.91) |  | 14 (12.73) |  |
| Other combinations | 138 | 13 (9.42) |  | 49 (35.51) |  | 13 (9.42) |  |
| **Three MetS Components (n = 542)** |  |  | 0.546 |  | **＜0.001^a^** |  | 0.254 |
| Abdominal obesity  + Elevated blood pressure + Elevated triglycerides | 199 | 21 (10.55) |  | **44 (22.11)** |  | 19 (9.55) |  |
| Abdominal obesity  + Elevated blood pressure + Elevated fasting glucose | 111 | 18 (16.22) |  | **53 (47.75)** |  | 17 (15.32) |  |
| Abdominal obesity  + Elevated blood pressure + Reduced HDL cholesterol | 84 | 11 (13.10) |  | 31 (36.90) |  | 8 (9.52) |  |
| Elevated blood pressure + Elevated triglycerides + Elevated fasting glucose | 58 | 6 (10.34) |  | **13 (22.41)** |  | 4 (6.90) |  |
| Other combinations | 90 | 14 (15.56) |  | 32 (35.56) |  | 14 (15.56) |  |
| **Four MetS Components (n = 180)** |  |  | 0.866 |  | 0.820 |  | 0.862 |
| Abdominal obesity  + Elevated blood pressure + Elevated triglycerides + Elevated fasting glucose | 93 | 10 (10.75) |  | 27 (29.03) |  | 9 (9.68) |  |
| Abdominal obesity  + Elevated blood pressure + Elevated triglycerides + Reduced HDL cholesterol | 51 | 7 (13.73) |  | 17 (33.33) |  | 6 (11.76) |  |
| Other combinations | 36 | 4 (11.11) |  | 10 (27.78) |  | 3 (8.33) |  |

**Notes:** ^a^, Post hoc comparisons among combinations of three MetS components: Abdominal obesity + Elevated blood pressure + Elevated triglycerides vs. Abdominal obesity + Elevated blood pressure + Elevated fasting glucose, *P*＜0.001; Abdominal obesity + Elevated blood pressure + Elevated fasting glucose vs. Elevated blood pressure + Elevated triglycerides+ Elevated fasting glucose, *P* = 0.014.

**Abbreviations:** MetS, metabolic syndrome.

**Supplementary Table S5**. Subgroup analysis of associations of MetS with ADL disability

| Variables | ADL disability [PR (95% CI)] | | | |
| --- | --- | --- | --- | --- |
|  | Without MetS (Ref) | With MetS (model 1) | With MetS (model 2) | With MetS (model 3) |
| Gender |  |  |  |  |
| Male | 1.00 (Ref) | 1.02 (0.99,1.06) | **1.04 (1.01,1.08)*** | 1.04 (1.00,1.08) |
| Female | 1.00 (Ref) | **1.06 (1.03,1.09)^***^** | **1.05 (1.02,1.08)**** | **1.05 (1.02,1.08)**** |
| Age group |  |  |  |  |
| 60–69 years | 1.00 (Ref) | **1.03 (1.00,1.06)^*^** | **1.03 (1.00,1.06)*** | **1.03 (1.00,1.06)*** |
| ≥ 70 years | 1.00 (Ref) | **1.06 (1.02,1.10)^**^** | **1.07 (1.03,1.11)**** | **1.07 (1.03,1.11)**** |
| Ethnic |  |  |  |  |
| Non–zhuang | 1.00 (Ref) | 1.05 (1.00,1.11) | **1.06 (1.00,1.12)*** | 1.05 (0.99,1.11) |
| Zhuang | 1.00 (Ref) | **1.04 (1.01,1.07)^**^** | **1.04 (1.01,1.06)**** | **1.04 (1.01,1.07)**** |
| Marital status |  |  |  |  |
| Partnered | 1.00 (Ref) | **1.03 (1.01,1.06)*** | **1.04 (1.01,1.06)**** | **1.04 (1.01,1.07)**** |
| Single | 1.00 (Ref) | **1.07 (1.02,1.12)**** | **1.06 (1.01,1.10)*** | **1.06 (1.01,1.11)*** |
| Educational attainment |  |  |  |  |
| Less than primary school | 1.00 (Ref) | **1.05 (1.02,1.09)**** | **1.05 (1.01,1.08)**** | **1.05 (1.01,1.09)**** |
| Primary school | 1.00 (Ref) | **1.04 (1.00,1.09)*** | **1.05 (1.01,1.09)*** | 1.04 (1.00,1.09) |
| High school and above | 1.00 (Ref) | 1.03 (0.98,1.07) | 1.03 (0.99,1.08) | 1.02 (0.97,1.06) |
| Occupation |  |  |  |  |
| Non–farmer | 1.00 (Ref) | 1.02 (0.97,1.08) | 1.05 (0.99,1.12) | 1.04 (0.99,1.10) |
| Farmer | 1.00 (Ref) | **1.05 (1.02,1.08)***** | **1.04 (1.02,1.07)**** | **1.04 (1.02,1.07)***** |
| Annual income |  |  |  |  |
| ＜10000 renminbi | 1.00 (Ref) | **1.06 (1.01,1.10)**** | **1.05 (1.01,1.09)*** | **1.05 (1.00,1.09)*** |
| 10000–29999 renminbi | 1.00 (Ref) | 1.04 (1.00,1.09) | 1.04 (0.99,1.08) | 1.04 (0.99,1.08) |
| ≥ 30000 renminbi | 1.00 (Ref) | **1.04 (1.00,1.08)*** | **1.04 (1.00,1.07)*** | **1.04 (1.01,1.08)*** |
| Cerebrovascular disease |  |  |  |  |
| No | 1.00 (Ref) | **1.04 (1.02,1.07)***** | **1.04 (1.02,1.07)***** | **1.04 (1.02,1.07)**** |
| Yes | 1.00 (Ref) | 1.08 (0.95,1.24) | 1.07 (0.94,1.21) | 1.10 (0.95,1.26) |
| Rheumatism |  |  |  |  |
| No | 1.00 (Ref) | **1.04 (1.02,1.07)***** | **1.04 (1.02,1.07)***** | **1.04 (1.02,1.07)**** |
| Yes | 1.00 (Ref) | 1.07 (0.98,1.17) | 1.07 (0.98,1.16) | 1.08 (0.98,1.18) |
| Osteoarthropathy |  |  |  |  |
| No | 1.00 (Ref) | **1.04 (1.02,1.07)**** | **1.04 (1.02,1.07)**** | **1.04 (1.01,1.06)**** |
| Yes | 1.00 (Ref) | 1.07 (0.98,1.17) | 1.07 (0.98,1.16) | **1.11 (1.01,1.21)*** |
| Smoking status |  |  |  |  |
| No | 1.00 (Ref) | **1.04 (1.02,1.07)**** | **1.04 (1.02,1.07)**** | **1.04 (1.02,1.07)**** |
| Yes | 1.00 (Ref) | 1.05 (0.99,1.12) | **1.06 (1.00,1.12)*** | 1.06 (1.00,1.13) |
| Alcohol consumption |  |  |  |  |
| No | 1.00 (Ref) | **1.06 (1.03,1.08)***** | **1.05 (1.02,1.08)***** | **1.05 (1.02,1.08)***** |
| Yes | 1.00 (Ref) | 1.01 (0.97,1.05) | 1.02 (0.98,1.07) | 1.02 (0.97,1.06) |
| Low grip stength |  |  |  |  |
| No | 1.00 (Ref) | 1.02 (1.00,1.05) | 1.02 (0.99,1.05) | 1.02 (0.99,1.05) |
| Yes | 1.00 (Ref) | **1.07 (1.03,1.10)***** | **1.06 (1.03,1.10)**** | **1.06 (1.02,1.10)**** |
| Anemia |  |  |  |  |
| No | 1.00 (Ref) | **1.03 (1.00,1.05)*** | **1.03 (1.00,1.05)*** | **1.03 (1.00,1.06)*** |
| Yes | 1.00 (Ref) | **1.10 (1.05,1.15)***** | **1.08 (1.03,1.14)**** | **1.08 (1.03,1.13)**** |
| low density lipoprotein cholesterol |  |  |  |  |
| Normal | 1.00 (Ref) | **1.06 (1.03,1.10)**** | **1.06 (1.02,1.09)**** | **1.06 (1.02,1.09)**** |
| High | 1.00 (Ref) | 1.03 (1.00,1.07) | 1.03 (0.99,1.06) | 1.03 (1.00,1.07) |
| Total cholesterol |  |  |  |  |
| Normal | 1.00 (Ref) | **1.05 (1.01,1.09)**** | **1.05 (1.01,1.09)*** | **1.05 (1.01,1.09)*** |
| High | 1.00 (Ref) | **1.04 (1.01,1.07)**** | **1.04 (1.01,1.07)**** | **1.04 (1.01,1.07)*** |
| Serum creatinine |  |  |  |  |
| Normal | 1.00 (Ref) | **1.04 (1.02,1.07)**** | **1.04 (1.02,1.07)**** | **1.05 (1.02,1.07)**** |
| Low | 1.00 (Ref) | 1.05 (1.00,1.11) | 1.04 (0.99,1.10) | 1.03 (0.98,1.09) |
| Uric acid |  |  |  |  |
| Normal | 1.00 (Ref) | **1.04 (1.01,1.07)**** | **1.04 (1.01,1.07)**** | **1.04 (1.01,1.07)**** |
| High | 1.00 (Ref) | 1.04 (1.00,1.09) | 1.04 (1.00,1.09) | 1.04 (0.99,1.10) |
| Aspartate aminotransferase |  |  |  |  |
| Normal | 1.00 (Ref) | **1.04 (1.01,1.07)**** | **1.04 (1.01,1.06)**** | **1.04 (1.01,1.06)*** |
| High | 1.00 (Ref) | **1.07 (1.02,1.12)**** | **1.07 (1.02,1.12)**** | **1.07 (1.01,1.13)*** |
| Alanine aminotransferasee |  |  |  |  |
| Normal | 1.00 (Ref) | **1.05 (1.02,1.07)***** | **1.04 (1.02,1.07)***** | **1.05 (1.02,1.07)**** |
| High | 1.00 (Ref) | 1.04 (0.97,1.12) | 1.04 (0.97,1.11) | 1.03 (0.96,1.10) |

**Notes:** *, *P*<0.05; **, *P*<0.01; ***, *P*<0.001.Model 1 was unadjusted model; Model 2 adjusted for sex, age, ethnic, marital status, educational attainment, occupation, annual income, cerebrovascular disease, rheumatism, osteoarthropathy, smoking status, and alcohol consumption; Model 3 further adjusted hand grip strength, anemia, total cholesterol, low density lipoprotein cholesterol, aspartate aminotransferase, alanine aminotransferasee, serum creatinine and uric acid.

**Abbreviations:** MetS, metabolic syndrome; ADL, activities of daily living; PR, prevalence ratio; CI, confidence interval.

**Supplementary Table S6.** Subgroup analysis of associations of MetS with IADL disability

| Variables | IADL disability [PR (95% CI)] | | | |
| --- | --- | --- | --- | --- |
|  | Without MetS (Ref) | With MetS (model 1) | With MetS (model 2) | With MetS (model 3) |
| Gender |  |  |  |  |
| Male | 1.00 (Ref) | 0.99 (0.95,1.04) | 1.03 (0.99,1.07) | 1.02 (0.97,1.07) |
| Female | 1.00 (Ref) | 0.99 (0.96,1.03) | 0.98 (0.95,1.01) | 0.97 (0.94,1.01) |
| Age group |  |  |  |  |
| 60–69 years | 1.00 (Ref) | **0.96 (0.93,1.00)*** | 0.97 (0.94,1.01) | **0.96 (0.92,1.00)*** |
| ≥ 70 years | 1.00 (Ref) | **1.04 (1.00,1.08)*** | 1.02 (0.98,1.06) | 1.01 (0.97,1.06) |
| Ethnic |  |  |  |  |
| Non–zhuang | 1.00 (Ref) | 1.01 (0.94,1.07) | 1.00 (0.95,1.07) | 1.00 (0.94,1.06) |
| Zhuang | 1.00 (Ref) | 1.00 (0.97,1.03) | 0.99 (0.96,1.02) | 0.98 (0.95,1.01) |
| Marital status |  |  |  |  |
| Partnered | 1.00 (Ref) | 1.00 (0.97,1.03) | 0.99 (0.96,1.02) | 0.99 (0.95,1.02) |
| Single | 1.00 (Ref) | 1.01 (0.97,1.06) | 0.99 (0.95,1.04) | 0.98 (0.94,1.03) |
| Educational attainment |  |  |  |  |
| Less than primary school | 1.00 (Ref) | 1.00 (0.96,1.04) | 0.98 (0.94,1.01) | 0.97 (0.93,1.01) |
| Primary school | 1.00 (Ref) | 1.01 (0.96,1.06) | 1.01 (0.96,1.06) | 1.00 (0.95,1.05) |
| High school and above | 1.00 (Ref) | 1.01 (0.96,1.06) | 1.01 (0.96,1.07) | 1.00 (0.94,1.06) |
| Occupation |  |  |  |  |
| Non–farmer | 1.00 (Ref) | 0.98 (0.91,1.04) | 1.00 (0.94,1.07) | 1.01 (0.94,1.08) |
| Farmer | 1.00 (Ref) | 1.02 (0.99,1.05) | 0.99 (0.96,1.02) | 0.98 (0.95,1.01) |
| Annual income |  |  |  |  |
| ＜10000 renminbi | 1.00 (Ref) | 1.04 (0.99,1.09) | 1.02 (0.97,1.06) | 1.00 (0.95,1.04) |
| 10000–29999 renminbi | 1.00 (Ref) | 0.99 (0.94,1.05) | 0.97 (0.93,1.02) | 0.96 (0.91,1.01) |
| ≥ 30000 renminbi | 1.00 (Ref) | 0.99 (0.95,1.03) | 0.98 (0.94,1.02) | 0.99 (0.95,1.04) |
| Cerebrovascular disease |  |  |  |  |
| No | 1.00 (Ref) | 1.00 (0.98,1.03) | 0.99 (0.97,1.02) | 0.99 (0.96,1.01) |
| Yes | 1.00 (Ref) | 1.06 (0.91,1.23) | 0.98 (0.87,1.12) | 0.96 (0.83,1.12) |
| Rheumatism |  |  |  |  |
| No | 1.00 (Ref) | 1.01 (0.98,1.04) | 1.00 (0.97,1.02) | 0.99 (0.96,1.02) |
| Yes | 1.00 (Ref) | 0.98 (0.88,1.08) | 0.97 (0.89,1.06) | 0.96 (0.88,1.04) |
| Osteoarthropathy |  |  |  |  |
| No | 1.00 (Ref) | 1.00 (0.97,1.03) | 0.99 (0.96,1.01) | 0.98 (0.95,1.01) |
| Yes | 1.00 (Ref) | 1.04 (0.94,1.15) | 1.06 (0.96,1.17) | 1.05 (0.95,1.18) |
| Smoking status |  |  |  |  |
| No | 1.00 (Ref) | 1.00 (0.97,1.03) | 0.99 (0.96,1.01) | 0.97 (0.95,1.00) |
| Yes | 1.00 (Ref) | 1.03 (0.96,1.11) | 1.05 (0.98,1.11) | 1.07 (1.00,1.14) |
| Alcohol consumption |  |  |  |  |
| No | 1.00 (Ref) | 1.01 (0.98,1.05) | 0.99 (0.97,1.02) | 0.99 (0.96,1.02) |
| Yes | 1.00 (Ref) | 0.97 (0.92,1.02) | 0.99 (0.94,1.04) | 0.98 (0.92,1.04) |
| Low grip strength |  |  |  |  |
| No | 1.00 (Ref) | 0.98 (0.95,1.02) | 0.98 (0.94,1.02) | 0.96 (0.92,1.00) |
| Yes | 1.00 (Ref) | 1.04 (1.00,1.08) | 1.01 (0.98,1.05) | 1.00 (0.96,1.04) |
| Anemia |  |  |  |  |
| No | 1.00 (Ref) | 0.99 (0.96,1.03) | 0.99 (0.96,1.02) | 0.97 (0.94,1.00) |
| Yes | 1.00 (Ref) | **1.06 (1.00,1.11)*** | 1.02 (0.97,1.07) | 1.02 (0.97,1.07) |
| Low density lipoprotein cholesterol |  |  |  |  |
| Normal | 1.00 (Ref) | 1.00 (0.96,1.04) | 0.98 (0.94,1.01) | 0.98 (0.94,1.01) |
| High | 1.00 (Ref) | 1.02 (0.97,1.06) | 1.00 (0.96,1.05) | 1.00 (0.96,1.05) |
| Total cholesterol |  |  |  |  |
| Normal | 1.00 (Ref) | 1.01 (0.96,1.05) | 0.98 (0.94,1.02) | 0.98 (0.94,1.02) |
| High | 1.00 (Ref) | 1.00 (0.97,1.04) | 1.00 (0.97,1.03) | 0.99 (0.95,1.02) |
| Serum creatinine |  |  |  |  |
| Normal | 1.00 (Ref) | 1.00 (0.97,1.03) | 0.99 (0.96,1.02) | 0.99 (0.96,1.02) |
| Low | 1.00 (Ref) | 1.02 (0.96,1.09) | 1.00 (0.94,1.06) | 0.98 (0.92,1.04) |
| Uric acid |  |  |  |  |
| Normal | 1.00 (Ref) | 0.99 (0.96,1.03) | 0.97 (0.94,1.00) | 0.97 (0.94,1.00) |
| High | 1.00 (Ref) | 1.04 (0.99,1.10) | 1.03 (0.98,1.08) | 1.02 (0.97,1.08) |
| Aspartate aminotransferase |  |  |  |  |
| Normal | 1.00 (Ref) | 1.00 (0.97,1.04) | 0.98 (0.95,1.01) | 0.97 (0.94,1.00) |
| High | 1.00 (Ref) | 1.02 (0.96,1.08) | 1.02 (0.96,1.08) | 1.02 (0.96,1.09) |
| Alanine aminotransferasee |  |  |  |  |
| Normal | 1.00 (Ref) | 1.00 (0.97,1.03) | 0.99 (0.96,1.01) | 0.98 (0.95,1.01) |
| High | 1.00 (Ref) | 1.08 (0.99,1.19) | 1.08 (0.99,1.18) | 1.06 (0.96,1.17) |

**Notes:** *, *P*<0.05. Model 1 was unadjusted model;Model 2 adjusted for sex, age, ethnic, marital status, educational attainment, occupation, annual income, cerebrovascular disease, rheumatism, osteoarthropathy, smoking status, and alcohol consumption; Model 3 further adjusted hand grip strength, anemia, total cholesterol, low density lipoprotein cholesterol, aspartate aminotransferase,

alanine aminotransferasee, serum creatinine, and uric acid.

**Abbreviations:** MetS, metabolic syndrome; IADL, instrumental activities of daily living; PR, prevalence ratio; CI, confidence interval.

**Supplementary Table S7**. Subgroup analysis of associations of MetS with comorbid ADL­IADL disability

| Variables | Comorbid ADL­IADL disability [PR(95% CI)] | | | |
| --- | --- | --- | --- | --- |
|  | Without MetS (Ref) | With MetS (model 1) | With MetS (model 2) | With MetS (model 3) |
| Gender |  |  |  |  |
| Male | 1.00 (Ref) | 1.18 (0.75,1.84) | **1.52 (1.00,2.31)*** | 1.40 (0.87,2.26) |
| Female | 1.00 (Ref) | **1.78 (1.35,2.36)***** | **1.56 (1.18,2.07)**** | **1.60 (1.17,2.17)**** |
| Age group |  |  |  |  |
| 60–69 years | 1.00 (Ref) | 1.55 (0.96,2.49) | 1.60 (0.99,2.58) | 1.47 (0.88,2.46) |
| ≥ 70 years | 1.00 (Ref) | **1.54 (1.18,2.01)**** | **1.60 (1.23,2.10)**** | **1.66 (1.23,2.24)**** |
| Ethnic |  |  |  |  |
| Non–zhuang | 1.00 (Ref) | 1.38 (0.85,2.26) | 1.53 (0.94,2.48) | 1.25 (0.75,2.10) |
| Zhuang | 1.00 (Ref) | **1.55 (1.18,2.03)**** | **1.52 (1.17,1.98)**** | **1.53 (1.14,2.05)**** |
| Marital status |  |  |  |  |
| Partnered | 1.00 (Ref) | 1.37 (0.98,1.91) | **1.53 (1.10,2.12)*** | **1.50 (1.05,2.15)*** |
| Single | 1.00 (Ref) | **1.75 (1.26,2.43)**** | **1.63 (1.18,2.27)**** | **1.61 (1.11,2.32)*** |
| Educational attainment |  |  |  |  |
| Less than primary school | 1.00 (Ref) | **1.62 (1.18,2.20)**** | **1.52 (1.11,2.08)*** | **1.56 (1.12,2.17)**** |
| Primary school | 1.00 (Ref) | 1.49 (0.98,2.27) | 1.56 (1.04,2.35) | 1.50 (0.95,2.38) |
| High school and above | 1.00 (Ref) | 1.54 (0.78,3.07) | 1.69 (0.84,3.37) | 1.54 (0.63,3.81) |
| Occupation |  |  |  |  |
| Non–farmer | 1.00 (Ref) | 1.42 (0.56,3.64) | 2.34 (0.87,6.29) | **2.58 (1.02,6.51)*** |
| Farmer | 1.00 (Ref) | **1.61 (1.27,2.06)***** | **1.50 (1.18,1.91)**** | **1.48 (1.13,1.94)***** |
| Annual income |  |  |  |  |
| ＜10000 renminbi | 1.00 (Ref) | **1.53 (1.09,2.14)*** | **1.50 (1.07,2.09)*** | 1.45 (0.98,2.16) |
| 10000–29999 renminbi | 1.00 (Ref) | **1.82 (1.11,2.99)*** | **1.67 (1.03,2.72)*** | 1.67 (0.98,2.84) |
| ≥ 30000 renminbi | 1.00 (Ref) | 1.54 (1.00,2.38) | **1.55 (1.02,2.36)*** | **1.68 (1.06,2.64)*** |
| Cerebrovascular disease |  |  |  |  |
| No | 1.00 (Ref) | **1.55 (1.21,1.97)***** | **1.52 (1.20,1.93)**** | **1.50 (1.15,1.95)**** |
| Yes | 1.00 (Ref) | 2.24 (0.80,6.22) | 2.33 (0.73,7.47) | 2.89 (0.75,11.20) |
| Rheumatism |  |  |  |  |
| No | 1.00 (Ref) | **1.55 (1.21,1.99)***** | **1.55 (1.21,1.98)***** | **1.53 (1.17,2.00)**** |
| Yes | 1.00 (Ref) | 1.81 (0.84,3.89) | 1.57 (0.65,3.80) | 1.56 (0.66,3.71) |
| Osteoarthropathy |  |  |  |  |
| No | 1.00 (Ref) | **1.51 (1.18,1.94)**** | **1.47 (1.15,1.88)**** | **1.41 (1.09,1.83)**** |
| Yes | 1.00 (Ref) | **2.22 (1.10,4.48)*** | **2.44 (1.22,4.89)*** | **3.99 (1.94,8.21)***** |
| Smoking status |  |  |  |  |
| No | 1.00 (Ref) | **1.54 (1.20,1.97)**** | **1.52 (1.18,1.94)**** | **1.50 (1.13,1.98)**** |
| Yes | 1.00 (Ref) | 1.69 (0.84,3.40) | 1.95 (1.00,3.79) | **2.14 (1.24,3.71)**** |
| Alcohol consumption |  |  |  |  |
| No | 1.00 (Ref) | **1.74 (1.36,2.23)***** | **1.69 (1.31,2.16)***** | **1.66 (1.26,2.18)***** |
| Yes | 1.00 (Ref) | 0.76 (0.35,1.64) | 0.85 (0.39,1.82) | 0.77 (0.30,1.94) |
| Low grip strength |  |  |  |  |
| No | 1.00 (Ref) | 1.46 (0.90,2.36) | 1.38 (0.86,2.21) | 1.33 (0.79,2.26) |
| Yes | 1.00 (Ref) | **1.68 (1.28,2.20)***** | **1.58 (1.20,2.08)**** | **1.57 (1.17,2.10)**** |
| Anemia |  |  |  |  |
| No | 1.00 (Ref) | **1.40 (1.01,1.94)*** | **1.40 (1.01,1.94)*** | 1.41 (0.99,2.00) |
| Yes | 1.00 (Ref) | **2.03 (1.44,2.85)***** | **1.78 (1.25,2.55)**** | **1.63 (1.11,2.40)*** |
| Low density lipoprotein cholesterol |  |  |  |  |
| Normal | 1.00 (Ref) | **1.66 (1.21,2.27)**** | **1.60 (1.17,2.17)**** | **1.60 (1.15,2.21)**** |
| High | 1.00 (Ref) | **1.48 (1.01,2.15)*** | 1.42 (0.97,2.09) | 1.41 (0.92,2.15) |
| Total cholesterol |  |  |  |  |
| Normal | 1.00 (Ref) | **1.52 (1.06,2.18)*** | **1.46 (1.03,2.08)*** | **1.50 (1.02,2.20)*** |
| High | 1.00 (Ref) | **1.62 (1.19,2.22)**** | **1.60 (1.16,2.19)**** | **1.50 (1.05,2.16)*** |
| Serum creatinine |  |  |  |  |
| Normal | 1.00 (Ref) | **1.59 (1.22,2.09)**** | **1.60 (1.22,2.08)**** | **1.62 (1.21,2.18)**** |
| Low | 1.00 (Ref) | 1.51 (0.91,2.50) | 1.34 (0.79,2.29) | 1.24 (0.70,2.22) |
| Uric acid |  |  |  |  |
| Normal | 1.00 (Ref) | **1.53 (1.15,2.04)**** | **1.53 (1.15,2.04)**** | **1.51 (1.11,2.05)**** |
| High | 1.00 (Ref) | 1.43 (0.94,2.19) | 1.33 (0.87,2.01) | 1.27 (0.79,2.06) |
| Aspartate aminotransferase |  |  |  |  |
| Normal | 1.00 (Ref) | **1.47 (1.12,1.92)**** | **1.43 (1.09,1.87)**** | **1.40 (1.04,1.87)*** |
| High | 1.00 (Ref) | **1.99 (1.23,3.21)**** | **1.94 (1.20,3.15)**** | **2.26 (1.32,3.88)**** |
| Alanine aminotransferasee |  |  |  |  |
| Normal | 1.00 (Ref) | **1.57 (1.23,2.01)***** | **1.52 (1.18,1.95)**** | **1.51 (1.15,1.97)**** |
| High | 1.00 (Ref) | 1.82 (0.75,4.41) | 1.72 (0.69,4.30) | 1.41 (0.42,4.72) |

**Notes:** *, *P*<0.05; **, *P*<0.01; ***, *P*<0.001. Model 1 was unadjusted model; Model 2 adjusted for sex, age, ethnic, marital status, educational attainment, occupation, annual income, cerebrovascular disease, rheumatism, osteoarthropathy, smoking status, and alcohol consumption; Model 3 further adjusted hand grip strength, anemia, total cholesterol, low density lipoprotein cholesterol, aspartate aminotransferase, alanine aminotransferasee, serum creatinine, and uric acid.

**Abbreviations:** MetS, metabolic syndrome; ADL, activities of daily living; IADL, instrumental activities of daily living; PR, prevalence ratio; CI, confidence interval.

**Supplementary Table S8.** Subgroup analysis of associations between the number of MetS components and ADL disability

| Variables | Models | The number of MetS components [PR(95% CI)] | | | | | PR (95% CI) for trend |
| --- | --- | --- | --- | --- | --- | --- | --- |
|  |  | 0 | 1 | 2 | 3 | ≥4 |  |
| Gender |  |  |  |  |  |  |  |
| Male | Model 1 | 1.00 (Ref) | 1.02 (0.99,1.05) | **1.05 (1.01,1.09)*** | 1.05 (1.00,1.10) | 1.04 (0.97,1.12) | **1.02 (1.00,1.03)**** |
|  | Model 2 | 1.00 (Ref) | 1.01 (0.98,1.04) | 1.05 (1.02,1.09) | 1.07 (1.02,1.12) | 1.07 (0.99,1.15) | **1.02 (1.01,1.04)***** |
|  | Model 3 | 1.00 (Ref) | 1.01 (0.98,1.04) | **1.05 (1.02,1.09)**** | **1.06 (1.01,1.12)*** | 1.04 (0.96,1.13) | **1.02 (1.01,1.04)**** |
| Female | Model 1 | 1.00 (Ref) | 1.01 (0.99,1.04) | 1.03 (1.00,1.05) | **1.08 (1.04,1.12)***** | **1.06 (1.01,1.12)*** | **1.02 (1.01,1.03)***** |
|  | Model 2 | 1.00 (Ref) | 1.00 (0.97,1.02) | 1.01 (0.98,1.04) | **1.06 (1.02,1.10)**** | 1.02 (0.97,1.08) | **1.01 (1.00,1.02)**** |
|  | Model 3 | 1.00 (Ref) | 1.01 (0.98,1.03) | 1.02 (0.99,1.05) | **1.07 (1.03,1.11)**** | 1.04 (0.98,1.09) | **1.02 (1.01,1.03)**** |
| Age group |  |  |  |  |  |  |  |
| 60–69 years | Model 1 | 1.00 (Ref) | 0.99 (0.97,1.02) | 1.00 (0.98,1.03) | **1.03 (1.00,1.07)*** | 1.01 (0.96,1.05) | 1.01 (1.00,1.02) |
|  | Model 2 | 1.00 (Ref) | 1.00 (0.97,1.02) | 1.00 (0.98,1.03) | **1.04 (1.00,1.07)*** | 1.01 (0.96,1.05) | 1.01 (1.00,1.02) |
|  | Model 3 | 1.00 (Ref) | 0.99 (0.97,1.02) | 1.00 (0.98,1.03) | 1.04 (1.00,1.08) | 1.00 (0.95,1.05) | 1.01 (1.00,1.02) |
| ≥ 70 years | Model 1 | 1.00 (Ref) | 1.03 (0.99,1.06) | **1.06 (1.02,1.10)**** | **1.09 (1.04,1.15)***** | **1.09 (1.02,1.17)*** | **1.03 (1.01,1.04)***** |
|  | Model 2 | 1.00 (Ref) | 1.03 (1.00,1.07) | **1.07 (1.03,1.11)**** | **1.11 (1.05,1.17)***** | **1.10 (1.02,1.18)*** | **1.03 (1.02,1.04)***** |
|  | Model 3 | 1.00 (Ref) | **1.04 (1.00,1.08)*** | **1.08 (1.04,1.13)***** | **1.12 (1.07,1.18)***** | **1.10 (1.02,1.19)*** | **1.03 (1.02,1.05)***** |
| Ethnic |  |  |  |  |  |  |  |
| Non–zhuang | Model 1 | 1.00 (Ref) | 0.98 (0.92,1.04) | 1.03 (0.96,1.11) | 1.08 (0.99,1.18) | 0.99 (0.90,1.08) | 1.02 (1.00,1.04) |
|  | Model 2 | 1.00 (Ref) | 0.98 (0.92,1.04) | 1.03 (0.97,1.11) | **1.09 (1.00,1.18)*** | 1.00 (0.91,1.10) | **1.02 (1.00,1.04)**** |
|  | Model 3 | 1.00 (Ref) | 0.99 (0.93,1.05) | 1.06 (0.99,1.14) | **1.09 (1.00,1.18)*** | 1.03 (0.93,1.15) | **1.02 (1.00,1.05)*** |
| Zhuang | Model 1 | 1.00 (Ref) | **1.02 (1.00,1.04)*** | **1.03 (1.01,1.06)**** | **1.06 (1.03,1.09)***** | **1.06 (1.01,1.12)*** | **1.02 (1.01,1.03)***** |
|  | Model 2 | 1.00 (Ref) | 1.00 (0.98,1.02) | 1.02 (1.00,1.04) | **1.05 (1.02,1.08)**** | 1.05 (1.00,1.10) | **1.01 (1.01,1.02)***** |
|  | Model 3 | 1.00 (Ref) | 1.01 (0.99,1.03) | **1.03 (1.00,1.05)*** | **1.06 (1.02,1.09)**** | 1.04 (0.99,1.09) | **1.02 (1.01,1.02)***** |
| Marital status |  |  |  |  |  |  |  |
| Partnered | Model 1 | 1.00 (Ref) | 1.01 (0.99,1.03) | **1.03 (1.01,1.06)**** | **1.06 (1.03,1.10)***** | 1.01 (0.97,1.05) | **1.01 (1.01,1.02)**** |
|  | Model 2 | 1.00 (Ref) | 1.01 (0.99,1.03) | **1.04 (1.01,1.06)**** | **1.06 (1.03,1.10)***** | 1.01 (0.97,1.05) | **1.01 (1.01,1.02)***** |
|  | Model 3 | 1.00 (Ref) | 1.01 (0.99,1.03) | **1.03 (1.01,1.06)*** | **1.07 (1.03,1.10)**** | 1.01 (0.97,1.06) | **1.02 (1.01,1.02)***** |
| Single | Model 1 | 1.00 (Ref) | 1.01 (0.97,1.06) | 1.03 (0.98,1.07) | **1.07 (1.01,1.14)*** | **1.11 (1.02,1.21)*** | **1.03 (1.01,1.04)**** |
|  | Model 2 | 1.00 (Ref) | 1.01 (0.97,1.06) | 1.03 (0.98,1.08) | **1.08 (1.01,1.15)*** | **1.12 (1.03,1.23)*** | **1.02 (1.00,1.03)*** |
|  | Model 3 | 1.00 (Ref) | 1.00 (0.96,1.04) | 1.02 (0.97,1.07) | 1.06 (1.00,1.13) | 1.07 (0.98,1.17) | **1.02 (1.00,1.04)**** |
| Educational attainment |  |  |  |  |  |  |  |
| Less than primary school | Model 1 | 1.00 (Ref) | 1.02 (0.99,1.05) | **1.05 (1.01,1.08)**** | **1.08 (1.04,1.13)***** | **1.07 (1.00,1.14)*** | **1.02 (1.01,1.04)***** |
|  | Model 2 | 1.00 (Ref) | 1.00 (0.97,1.03) | 1.02 (0.99,1.05) | **1.06 (1.01,1.11)*** | 1.03 (0.97,1.10) | **1.02 (1.01,1.03)**** |
|  | Model 3 | 1.00 (Ref) | 1.01 (0.98,1.04) | 1.03 (1.00,1.07) | **1.08 (1.03,1.13)*** | 1.04 (0.98,1.11) | **1.02 (1.01,1.03)**** |
| Primary school | Model 1 | 1.00 (Ref) | 1.02 (0.99,1.06) | 1.04 (1.00,1.08) | **1.08 (1.03,1.14)**** | 1.02 (0.96,1.10) | **1.02 (1.00,1.03)*** |
|  | Model 2 | 1.00 (Ref) | 1.02 (0.99,1.05) | **1.04 (1.00,1.08)*** | **1.08 (1.03,1.14)**** | 1.03 (0.97,1.10) | **1.02 (1.01,1.03)**** |
|  | Model 3 | 1.00 (Ref) | 1.02 (0.99,1.06) | **1.04 (1.00,1.08)*** | **1.09 (1.03,1.15)**** | 1.02 (0.95,1.10) | **1.02 (1.00,1.03)**** |
| High school and above | Model 1 | 1.00 (Ref) | 0.99 (0.95,1.03) | 1.00 (0.96,1.05) | 1.01 (0.95,1.07) | 1.07 (0.96,1.18) | 1.01 (0.99,1.03) |
|  | Model 2 | 1.00 (Ref) | 0.98 (0.94,1.02) | 1.00 (0.96,1.05) | 1.02 (0.96,1.08) | 1.04 (0.95,1.15) | 1.01 (1.00,1.03) |
|  | Model 3 | 1.00 (Ref) | 0.98 (0.94,1.03) | 1.01 (0.96,1.06) | 1.00 (0.95,1.07) | 1.04 (0.94,1.16) | 1.01 (0.99,1.03) |
| Occupation |  |  |  |  |  |  |  |
| Non–farmer | Model 1 | 1.00 (Ref) | 0.96 (0.90,1.03) | 1.01 (0.94,1.10) | 1.02 (0.93,1.11) | 0.99 (0.88,1.10) | 1.01 (0.99,1.03) |
|  | Model 2 | 1.00 (Ref) | 0.96 (0.89,1.03) | 1.01 (0.93,1.09) | 1.03 (0.95,1.13) | 1.00 (0.90,1.12) | 1.02 (0.99,1.04) |
|  | Model 3 | 1.00 (Ref) | 0.96 (0.89,1.03) | 1.01 (0.93,1.09) | 1.04 (0.95,1.15) | 1.02 (0.90,1.15) | 1.02 (1.00,1.04) |
| Farmer | Model 1 | 1.00 (Ref) | 1.02 (1.00,1.04) | **1.04 (1.02,1.06)**** | **1.08 (1.04,1.11)***** | **1.06 (1.02,1.11)**** | **1.02 (1.01,1.03)***** |
|  | Model 2 | 1.00 (Ref) | 1.00 (0.98,1.02) | 1.02 (1.00,1.05) | **1.06 (1.03,1.09)***** | 1.04 (0.99,1.09) | **1.02 (1.01,1.02)***** |
|  | Model 3 | 1.00 (Ref) | 1.01 (0.99,1.03) | **1.03 (1.01,1.06)*** | **1.07 (1.03,1.10)***** | 1.04 (0.99,1.09) | **1.02 (1.01,1.03)***** |
| Annual income |  |  |  |  |  |  |  |
| ＜10000 renminbi | Model 1 | 1.00 (Ref) | 1.02 (0.99,1.06) | **1.06 (1.02,1.11)**** | **1.09 (1.04,1.15)**** | 1.07 (1.00,1.16) | **1.03 (1.01,1.04)***** |
|  | Model 2 | 1.00 (Ref) | 1.01 (0.98,1.04) | **1.04 (1.00,1.08)*** | **1.08 (1.03,1.14)**** | 1.04 (0.97,1.12) | **1.02 (1.01,1.04)**** |
|  | Model 3 | 1.00 (Ref) | 1.01 (0.98,1.05) | **1.05 (1.01,1.09)*** | **1.09 (1.03,1.15)**** | 1.02 (0.95,1.11) | **1.02 (1.01,1.04)**** |
| 10000–29999 renminbi | Model 1 | 1.00 (Ref) | 0.99 (0.96,1.03) | 1.03 (0.98,1.07) | 1.04 (0.98,1.10) | 1.07 (0.98,1.16) | **1.02 (1.00,1.03)**** |
|  | Model 2 | 1.00 (Ref) | 0.99 (0.95,1.02) | 1.02 (0.98,1.06) | 1.04 (0.98,1.10) | 1.04 (0.96,1.12) | **1.01 (1.00,1.03)*** |
|  | Model 3 | 1.00 (Ref) | 0.99 (0.96,1.03) | 1.03 (0.99,1.08) | 1.05 (0.99,1.11) | 1.04 (0.96,1.13) | **1.02 (1.00,1.03)*** |
| ≥ 30000 renminbi | Model 1 | 1.00 (Ref) | 1.02 (0.99,1.05) | 1.01 (0.98,1.05) | **1.06 (1.01,1.11)*** | 1.02 (0.96,1.09) | **1.01 (1.00,1.02)*** |
|  | Model 2 | 1.00 (Ref) | 1.00 (0.97,1.04) | 1.00 (0.97,1.04) | 1.05 (1.00,1.10) | 1.02 (0.95,1.09) | 1.01 (1.00,1.02) |
|  | Model 3 | 1.00 (Ref) | 1.00 (0.97,1.04) | 1.01 (0.97,1.05) | **1.05 (1.00,1.11)*** | 1.04 (0.96,1.12) | **1.01 (1.00,1.03)*** |
| Cerebrovascular disease |  |  |  |  |  |  |  |
| No | Model 1 | 1.00 (Ref) | 1.01 (0.99,1.03) | **1.03 (1.01,1.06)**** | **1.07 (1.03,1.10)***** | **1.05 (1.00,1.09)*** | **1.02 (1.01,1.03)***** |
|  | Model 2 | 1.00 (Ref) | 1.00 (0.98,1.02) | 1.02 (1.00,1.04) | **1.06 (1.02,1.09)***** | 1.03 (0.99,1.07) | **1.02 (1.01,1.02)***** |
|  | Model 3 | 1.00 (Ref) | 1.00 (0.98,1.02) | **1.03 (1.00,1.05)*** | **1.06 (1.03,1.10)***** | 1.03 (0.98,1.07) | **1.02 (1.01,1.02)***** |
| Yes | Model 1 | 1.00 (Ref) | 1.06 (0.99,1.15) | 1.07 (0.98,1.18) | 1.09 (0.94,1.28) | 1.25 (0.96,1.63) | 1.04 (1.00,1.08) |
|  | Model 2 | 1.00 (Ref) | 1.05 (0.97,1.15) | 1.07 (0.98,1.18) | 1.11 (0.95,1.30) | 1.13 (0.88,1.43) | 1.03 (0.99,1.07) |
|  | Model 3 | 1.00 (Ref) | 1.06 (0.98,1.16) | 1.10 (1.00,1.21) | 1.17 (0.99,1.39) | 1.14 (0.92,1.42) | **1.04 (1.00,1.08)*** |
| Rheumatism |  |  |  |  |  |  |  |
| No | Model 1 | 1.00 (Ref) | 1.01 (0.99,1.04) | **1.03 (1.01,1.06)**** | **1.06 (1.03,1.10)***** | **1.06 (1.01,1.11)*** | **1.02 (1.01,1.03)***** |
|  | Model 2 | 1.00 (Ref) | 1.00 (0.98,1.02) | 1.02 (1.00,1.05) | **1.05 (1.02,1.09)**** | 1.04 (1.00,1.09) | **1.02 (1.01,1.02)***** |
|  | Model 3 | 1.00 (Ref) | 1.00 (0.98,1.03) | **1.03 (1.00,1.05)*** | **1.06 (1.03,1.09)**** | 1.04 (0.99,1.09) | **1.02 (1.01,1.03)***** |
| Yes | Model 1 | 1.00 (Ref) | 1.02 (0.97,1.08) | 1.06 (0.99,1.14) | **1.13 (1.00,1.27)*** | 1.05 (0.93,1.18) | **1.03 (1.00,1.05)*** |
|  | Model 2 | 1.00 (Ref) | 1.01 (0.96,1.05) | 1.03 (0.96,1.10) | **1.14 (1.01,1.29)*** | 0.97 (0.88,1.07) | 1.02 (0.99,1.04) |
|  | Model 3 | 1.00 (Ref) | 1.01 (0.97,1.06) | 1.05 (0.98,1.13) | **1.15 (1.02,1.30)*** | 0.98 (0.88,1.10) | 1.03 (1.00,1.05) |
| Osteoarthropathy |  |  |  |  |  |  |  |
| No | Model 1 | 1.00 (Ref) | 1.01 (0.99,1.04) | **1.03 (1.01,1.06)*** | **1.06 (1.03,1.10)***** | **1.06 (1.01,1.11)*** | **1.02 (1.01,1.03)***** |
|  | Model 2 | 1.00 (Ref) | 1.00 (0.98,1.02) | 1.02 (0.99,1.04) | **1.05 (1.02,1.08)**** | 1.03 (0.99,1.08) | **1.01 (1.01,1.02)***** |
|  | Model 3 | 1.00 (Ref) | 1.00 (0.98,1.02) | 1.02 (1.00,1.05) | **1.05 (1.02,1.09)**** | 1.03 (0.98,1.08) | **1.01 (1.01,1.02)**** |
| Yes | Model 1 | 1.00 (Ref) | 1.02 (0.97,1.06) | 1.05 (1.00,1.10) | **1.12 (1.01,1.25)*** | 1.02 (0.89,1.16) | **1.02 (1.00,1.05)*** |
|  | Model 2 | 1.00 (Ref) | 1.00 (0.96,1.05) | 1.04 (0.99,1.09) | **1.12 (1.01,1.23)*** | 0.99 (0.86,1.14) | **1.02 (1.00,1.04)*** |
|  | Model 3 | 1.00 (Ref) | 1.01 (0.96,1.05) | **1.06 (1.00,1.12)*** | **1.17 (1.04,1.30)**** | 1.04 (0.89,1.21) | **1.03 (1.01,1.06)**** |
| Smoking status |  |  |  |  |  |  |  |
| No | Model 1 | 1.00 (Ref) | 1.01 (0.99,1.04) | **1.04 (1.01,1.06)**** | **1.06 (1.03,1.09)***** | **1.06 (1.01,1.11)*** | **1.02 (1.01,1.03)***** |
|  | Model 2 | 1.00 (Ref) | 1.00 (0.97,1.02) | 1.02 (0.99,1.04) | **1.05 (1.02,1.08)*** | 1.04 (0.99,1.09) | **1.01 (1.01,1.02)***** |
|  | Model 3 | 1.00 (Ref) | 1.00 (0.98,1.02) | **1.03 (1.00,1.05)*** | **1.06 (1.02,1.09)**** | 1.04 (0.99,1.09) | **1.02 (1.01,1.03)***** |
| Yes | Model 1 | 1.00 (Ref) | 1.02 (0.98,1.06) | 1.03 (0.98,1.09) | **1.10 (1.02,1.19)*** | **0.96 (0.93,0.99)*** | 1.02 (1.00,1.03) |
|  | Model 2 | 1.00 (Ref) | 1.02 (0.98,1.06) | 1.04 (0.99,1.09) | **1.11 (1.03,1.20)*** | 0.98 (0.94,1.03) | **1.02 (1.00,1.04)*** |
|  | Model 3 | 1.00 (Ref) | 1.02 (0.98,1.06) | 1.05 (1.00,1.11) | **1.12 (1.03,1.21)**** | 0.98 (0.92,1.04) | **1.03 (1.01,1.05)*** |
| Alcohol consumption |  |  |  |  |  |  |  |
| No | Model 1 | 1.00 (Ref) | 1.02 (0.99,1.04) | **1.03 (1.01,1.06)*** | **1.08 (1.04,1.12)***** | **1.06 (1.01,1.12)*** | **1.02 (1.01,1.03)***** |
|  | Model 2 | 1.00 (Ref) | 0.99 (0.97,1.02) | 1.02 (0.99,1.04) | **1.06 (1.02,1.10)**** | 1.03 (0.98,1.08) | **1.02 (1.01,1.02)***** |
|  | Model 3 | 1.00 (Ref) | 1.00 (0.98,1.02) | 1.02 (1.00,1.05) | **1.07 (1.03,1.11)***** | 1.03 (0.98,1.08) | **1.02 (1.01,1.03)***** |
| Yes | Model 1 | 1.00 (Ref) | 1.02 (0.98,1.06) | **1.04 (1.00,1.09)*** | 1.03 (0.98,1.09) | 1.03 (0.95,1.12) | 1.01 (1.00,1.03) |
|  | Model 2 | 1.00 (Ref) | 1.02 (0.98,1.06) | **1.05 (1.01,1.10)*** | 1.05 (1.00,1.11) | 1.04 (0.97,1.13) | **1.02 (1.00,1.03)*** |
|  | Model 3 | 1.00 (Ref) | 1.02 (0.99,1.06) | **1.06 (1.01,1.11)*** | 1.05 (0.99,1.11) | 1.05 (0.95,1.16) | **1.02 (1.00,1.03)*** |
| Low grip strength |  |  |  |  |  |  |  |
| No | Model 1 | 1.00 (Ref) | 0.99 (0.97,1.02) | 1.01 (0.98,1.04) | 1.02 (0.99,1.06) | 1.02 (0.97,1.07) | 1.01 (1.00,1.02) |
|  | Model 2 | 1.00 (Ref) | 0.99 (0.96,1.02) | 1.01 (0.98,1.04) | 1.02 (0.98,1.06) | 1.01 (0.96,1.06) | 1.01 (1.00,1.02) |
|  | Model 3 | 1.00 (Ref) | 0.99 (0.96,1.02) | 1.01 (0.98,1.04) | 1.02 (0.98,1.07) | 1.01 (0.96,1.07) | 1.01 (1.00,1.02) |
| Yes | Model 1 | 1.00 (Ref) | **1.03 (1.00,1.06)*** | **1.05 (1.02,1.09)**** | **1.10 (1.06,1.15)***** | **1.09 (1.02,1.16)*** | **1.03 (1.02,1.04)***** |
|  | Model 2 | 1.00 (Ref) | 1.01 (0.98,1.04) | **1.04 (1.01,1.07)*** | **1.09 (1.04,1.14)***** | 1.06 (0.99,1.13) | **1.02 (1.01,1.04)***** |
|  | Model 3 | 1.00 (Ref) | 1.01 (0.99,1.04) | **1.05 (1.01,1.08)**** | **1.10 (1.05,1.15)***** | 1.05 (0.98,1.12) | **1.02 (1.01,1.04)***** |
| Anemia |  |  |  |  |  |  |  |
| No | Model 1 | 1.00 (Ref) | **1.03 (1.00,1.05)*** | **1.04 (1.01,1.06)**** | **1.06 (1.02,1.09)**** | 1.04 (1.00,1.09) | **1.01 (1.01,1.02)**** |
|  | Model 2 | 1.00 (Ref) | 1.01 (0.99,1.04) | **1.03 (1.00,1.05)*** | **1.05 (1.01,1.08)**** | 1.03 (0.98,1.07) | **1.01 (1.00,1.02)**** |
|  | Model 3 | 1.00 (Ref) | 1.02 (0.99,1.04) | **1.03 (1.00,1.06)*** | **1.06 (1.02,1.10)**** | 1.02 (0.98,1.07) | **1.01 (1.00,1.02)**** |
| Yes | Model 1 | 1.00 (Ref) | 1.01 (0.97,1.04) | **1.05 (1.01,1.09)*** | **1.11 (1.05,1.18)***** | **1.12 (1.02,1.24)*** | **1.03 (1.02,1.05)***** |
|  | Model 2 | 1.00 (Ref) | 0.98 (0.95,1.01) | 1.03 (0.99,1.07) | **1.08 (1.02,1.15)**** | 1.08 (0.99,1.19) | **1.03 (1.01,1.04)***** |
|  | Model 3 | 1.00 (Ref) | 0.98 (0.95,1.02) | 1.03 (0.99,1.08) | **1.08 (1.01,1.15)*** | 1.09 (0.98,1.20) | **1.03 (1.01,1.04)**** |
| Low density lipoprotein cholesterol | |  |  |  |  |  |  |
| Normal | Model 1 | 1.00 (Ref) | 1.01 (0.98,1.03) | **1.04 (1.01,1.08)**** | **1.08 (1.04,1.13)***** | 1.05 (0.99,1.12) | **1.02 (1.01,1.03)***** |
|  | Model 2 | 1.00 (Ref) | 0.99 (0.96,1.01) | 1.03 (0.99,1.06) | **1.07 (1.03,1.12)**** | 1.03 (0.97,1.10) | **1.02 (1.01,1.03)***** |
|  | Model 3 | 1.00 (Ref) | 0.99 (0.97,1.02) | **1.03 (1.00,1.07)*** | **1.07 (1.03,1.12)**** | 1.04 (0.98,1.10) | **1.02 (1.01,1.03)***** |
| High | Model 1 | 1.00 (Ref) | 1.03 (0.99,1.06) | 1.03 (1.00,1.07) | **1.06 (1.01,1.10)*** | 1.04 (0.98,1.11) | **1.01 (1.00,1.02)*** |
|  | Model 2 | 1.00 (Ref) | 1.02 (0.98,1.05) | 1.02 (0.99,1.06) | **1.05 (1.00,1.10)*** | 1.02 (0.96,1.09) | 1.01 (1.00,1.02) |
|  | Model 3 | 1.00 (Ref) | 1.02 (0.99,1.05) | 1.03 (1.00,1.06) | **1.06 (1.01,1.11)*** | 1.03 (0.97,1.10) | **1.01 (1.00,1.03)*** |
| Total cholesterol |  |  |  |  |  |  |  |
| Normal | Model 1 | 1.00 (Ref) | 1.00 (0.97,1.03) | **1.04 (1.01,1.08)*** | **1.07 (1.02,1.12)**** | 1.04 (0.97,1.11) | **1.02 (1.01,1.03)***** |
|  | Model 2 | 1.00 (Ref) | 0.98 (0.95,1.00) | 1.03 (0.99,1.06) | **1.05 (1.01,1.11)*** | 1.01 (0.95,1.08) | **1.02 (1.01,1.03)**** |
|  | Model 3 | 1.00 (Ref) | 0.98 (0.95,1.01) | 1.03 (1.00,1.07) | **1.06 (1.01,1.12)*** | 1.03 (0.96,1.10) | **1.02 (1.01,1.03)**** |
| High | Model 1 | 1.00 (Ref) | **1.03 (1.00,1.06)*** | **1.03 (1.00,1.06)*** | **1.07 (1.03,1.11)**** | **1.07 (1.01,1.13)*** | **1.02 (1.01,1.03)**** |
|  | Model 2 | 1.00 (Ref) | 1.02 (0.99,1.05) | 1.02 (1.00,1.05) | **1.06 (1.02,1.10)*** | 1.05 (1.00,1.11) | **1.02 (1.01,1.03)**** |
|  | Model 3 | 1.00 (Ref) | 1.02 (0.99,1.05) | 1.02 (0.99,1.05) | **1.06 (1.02,1.11)*** | 1.03 (0.97,1.09) | **1.01 (1.00,1.03)**** |
| Serum creatinine |  |  |  |  |  |  |  |
| Normal | Model 1 | 1.00 (Ref) | 1.01 (0.99,1.03) | **1.04 (1.01,1.06)**** | **1.06 (1.03,1.10)***** | **1.06 (1.01,1.11)*** | **1.02 (1.01,1.03)***** |
|  | Model 2 | 1.00 (Ref) | 0.99 (0.97,1.02) | 1.02 (1.00,1.05) | **1.05 (1.02,1.09)**** | 1.04 (0.99,1.09) | **1.02 (1.01,1.02)***** |
|  | Model 3 | 1.00 (Ref) | 1.00 (0.98,1.02) | **1.03 (1.00,1.06)*** | **1.06 (1.02,1.10)**** | 1.05 (1.00,1.10) | **1.02 (1.01,1.03)***** |
| Low | Model 1 | 1.00 (Ref) | 1.01 (0.97,1.06) | 1.02 (0.97,1.08) | **1.07 (1.00,1.15)*** | 1.05 (0.94,1.16) | **1.02 (1.00,1.04)*** |
|  | Model 2 | 1.00 (Ref) | 1.01 (0.97,1.05) | 1.02 (0.97,1.08) | 1.06 (1.00,1.14) | 1.03 (0.94,1.12) | 1.02 (1.00,1.03) |
|  | Model 3 | 1.00 (Ref) | 1.02 (0.97,1.06) | 1.03 (0.98,1.08) | 1.07 (1.00,1.15) | 0.98 (0.91,1.06) | 1.01 (0.99,1.03) |
| Uric acid |  |  |  |  |  |  |  |
| Normal | Model 1 | 1.00 (Ref) | 1.02 (1.00,1.04) | **1.03 (1.00,1.05)*** | **1.06 (1.03,1.10)***** | 1.04 (0.99,1.10) | **1.02 (1.01,1.02)***** |
|  | Model 2 | 1.00 (Ref) | 1.00 (0.98,1.02) | 1.01 (0.99,1.04) | **1.05 (1.02,1.09)**** | 1.03 (0.98,1.08) | **1.01 (1.00,1.02)**** |
|  | Model 3 | 1.00 (Ref) | 1.01 (0.99,1.03) | 1.02 (1.00,1.05) | **1.06 (1.02,1.10)**** | 1.02 (0.97,1.07) | **1.01 (1.00,1.02)**** |
| High | Model 1 | 1.00 (Ref) | 0.99 (0.93,1.06) | 1.06 (0.99,1.13) | 1.06 (0.99,1.15) | 1.06 (0.97,1.16) | **1.02 (1.01,1.04)**** |
|  | Model 2 | 1.00 (Ref) | 0.99 (0.93,1.05) | 1.06 (0.99,1.13) | 1.07 (1.00,1.15) | 1.05 (0.96,1.15) | **1.03 (1.01,1.04)**** |
|  | Model 3 | 1.00 (Ref) | 0.98 (0.92,1.05) | **1.07 (1.00,1.15)*** | 1.08 (1.00,1.16) | 1.06 (0.96,1.17) | **1.03 (1.01,1.05)**** |
| Aspartate aminotransferase |  |  |  |  |  |  |  |
| Normal | Model 1 | 1.00 (Ref) | 1.00 (0.98,1.03) | **1.03 (1.00,1.06)*** | **1.05 (1.02,1.09)**** | 1.04 (0.99,1.09) | **1.02 (1.01,1.03)***** |
|  | Model 2 | 1.00 (Ref) | 0.99 (0.97,1.01) | 1.02 (0.99,1.04) | **1.04 (1.01,1.08)*** | 1.02 (0.97,1.07) | **1.01 (1.00,1.02)**** |
|  | Model 3 | 1.00 (Ref) | 1.00 (0.97,1.02) | **1.03 (1.00,1.05)*** | **1.05 (1.01,1.09)**** | 1.02 (0.97,1.07) | **1.01 (1.01,1.02)**** |
| High | Model 1 | 1.00 (Ref) | **1.05 (1.02,1.09)**** | **1.05 (1.01,1.09)*** | **1.11 (1.05,1.18)***** | **1.11 (1.01,1.21)*** | **1.03 (1.01,1.04)**** |
|  | Model 2 | 1.00 (Ref) | **1.04 (1.00,1.08)*** | 1.04 (1.00,1.08) | **1.10 (1.04,1.17)**** | 1.09 (1.00,1.19) | **1.02 (1.01,1.04)**** |
|  | Model 3 | 1.00 (Ref) | **1.04 (1.01,1.09)*** | **1.05 (1.01,1.10)*** | **1.12 (1.05,1.19)**** | 1.10 (0.99,1.22) | **1.03 (1.01,1.05)**** |
| Alanine aminotransferasee |  |  |  |  |  |  |  |
| Normal | Model 1 | 1.00 (Ref) | 1.01 (0.99,1.03) | **1.04 (1.01,1.06)**** | **1.07 (1.04,1.10)***** | **1.05 (1.00,1.10)*** | **1.02 (1.01,1.03)***** |
|  | Model 2 | 1.00 (Ref) | 1.00 (0.98,1.02) | **1.02 (1.00,1.05)*** | **1.06 (1.02,1.09)**** | 1.03 (0.99,1.08) | **1.02 (1.01,1.02)***** |
|  | Model 3 | 1.00 (Ref) | 1.00 (0.98,1.02) | **1.03 (1.01,1.05)*** | **1.07 (1.03,1.10)***** | 1.03 (0.99,1.08) | **1.02 (1.01,1.03)***** |
| High | Model 1 | 1.00 (Ref) | 1.06 (0.97,1.17) | 1.03 (0.95,1.11) | 1.07 (0.98,1.17) | 1.11 (0.96,1.28) | 1.02 (0.99,1.05) |
|  | Model 2 | 1.00 (Ref) | 1.08 (0.97,1.20) | 1.03 (0.94,1.12) | 1.08 (0.98,1.19) | 1.09 (0.95,1.26) | 1.01 (0.99,1.04) |
|  | Model 3 | 1.00 (Ref) | 1.11 (0.98,1.25) | 1.06 (0.95,1.17) | 1.09 (0.97,1.23) | 1.10 (0.92,1.31) | 1.01 (0.98,1.05) |

**Notes:** *, *P*<0.05; **, *P*<0.01; ***, *P*<0.001. Model 1 was unadjusted model; Model 2 adjusted for sex, age, ethnic, marital status, educational attainment, occupation, annual income, cerebrovascular disease, rheumatism, osteoarthropathy, smoking status, and alcohol consumption; Model 3 further adjusted hand grip strength, anemia, total cholesterol, low density lipoprotein cholesterol, aspartate aminotransferase, alanine aminotransferasee, serum creatinine, and uric acid.

**Abbreviations:** MetS, metabolic syndrome; ADL, activities of daily living; PR, prevalence ratio; CI, confidence interval.

**Supplementary Table S9.** Subgroup analysis of associations between the number of MetS components and IADL disability

| Variables | Models | The number of MetS components [PR(95% CI)] | | | | | PR (95% CI) for trend |
| --- | --- | --- | --- | --- | --- | --- | --- |
|  |  | 0 | 1 | 2 | 3 | ≥4 |  |
| Gender |  |  |  |  |  |  |  |
| Male | Model 1 | 1.00 (Ref) | 1.03 (0.99,1.08) | 1.04 (0.99,1.09) | 1.03 (0.97,1.10) | 0.99 (0.91,1.08) | 1.01 (0.99,1.02) |
|  | Model 2 | 1.00 (Ref) | 1.01 (0.97,1.05) | 1.03 (0.99,1.08) | 1.05 (1.00,1.12) | 1.02 (0.94,1.11) | 1.01 (1.00,1.03) |
|  | Model 3 | 1.00 (Ref) | 1.01 (0.97,1.05) | 1.03 (0.99,1.08) | 1.05 (0.99,1.12) | 0.96 (0.88,1.06) | 1.01 (0.99,1.03) |
| Female | Model 1 | 1.00 (Ref) | 1.04 (1.00,1.08) | **1.06 (1.02,1.10)**** | 1.03 (0.98,1.08) | 1.02 (0.96,1.09) | 1.01 (1.00,1.02) |
|  | Model 2 | 1.00 (Ref) | 1.01 (0.98,1.05) | 1.03 (0.99,1.07) | 1.00 (0.96,1.05) | 0.97 (0.91,1.03) | 1.00 (0.99,1.01) |
|  | Model 3 | 1.00 (Ref) | 1.02 (0.98,1.06) | 1.03 (0.99,1.07) | 1.00 (0.95,1.05) | 0.97 (0.90,1.03) | 1.00 (0.98,1.01) |
| Age group |  |  |  |  |  |  |  |
| 60–69 years | Model 1 | 1.00 (Ref) | 0.99 (0.96,1.03) | 1.00 (0.96,1.04) | 0.96 (0.91,1.00) | 0.97 (0.90,1.04) | 0.99 (0.98,1.00) |
|  | Model 2 | 1.00 (Ref) | 1.00 (0.96,1.04) | 1.01 (0.97,1.05) | 0.97 (0.93,1.02) | 0.97 (0.90,1.04) | 0.99 (0.98,1.01) |
|  | Model 3 | 1.00 (Ref) | 1.00 (0.97,1.04) | 1.01 (0.97,1.05) | 0.97 (0.92,1.02) | 0.95 (0.88,1.02) | 0.99 (0.98,1.01) |
| ≥ 70 years | Model 1 | 1.00 (Ref) | 1.04 (1.00,1.09) | **1.08 (1.03,1.14)**** | **1.10 (1.04,1.17)**** | 1.06 (0.98,1.15) | **1.02 (1.01,1.04)**** |
|  | Model 2 | 1.00 (Ref) | 1.04 (1.00,1.09) | **1.06 (1.02,1.12)**** | **1.08 (1.02,1.14)**** | 1.01 (0.94,1.09) | 1.01 (1.00,1.03) |
|  | Model 3 | 1.00 (Ref) | 1.04 (1.00,1.09) | **1.07 (1.02,1.12)*** | **1.08 (1.02,1.14)*** | 1.00 (0.92,1.09) | 1.01 (1.00,1.03) |
| Ethnic |  |  |  |  |  |  |  |
| Non–zhuang | Model 1 | 1.00 (Ref) | 1.00 (0.92,1.08) | 1.01 (0.92,1.10) | 1.03 (0.93,1.13) | 0.97 (0.85,1.09) | 1.00 (0.98,1.03) |
|  | Model 2 | 1.00 (Ref) | 1.00 (0.93,1.08) | 1.01 (0.93,1.09) | 1.03 (0.94,1.12) | 0.96 (0.86,1.07) | 1.00 (0.98,1.02) |
|  | Model 3 | 1.00 (Ref) | 1.00 (0.92,1.08) | 1.02 (0.94,1.11) | 1.02 (0.93,1.11) | 0.98 (0.87,1.11) | 1.00 (0.98,1.03) |
| Zhuang | Model 1 | 1.00 (Ref) | **1.04 (1.00,1.07)*** | **1.06 (1.02,1.09)**** | 1.04 (0.99,1.08) | 1.03 (0.97,1.10) | **1.01 (1.00,1.02)*** |
|  | Model 2 | 1.00 (Ref) | 1.01 (0.98,1.04) | 1.03 (0.99,1.06) | 1.01 (0.97,1.05) | 0.98 (0.93,1.04) | 1.00 (0.99,1.01) |
|  | Model 3 | 1.00 (Ref) | 1.01 (0.98,1.04) | 1.03 (0.99,1.06) | 1.01 (0.97,1.05) | 0.95 (0.90,1.02) | 1.00 (0.99,1.01) |
| Marital status |  |  |  |  |  |  |  |
| Partnered | Model 1 | 1.00 (Ref) | 1.02 (0.98,1.05) | 1.03 (0.99,1.07) | 1.02 (0.98,1.07) | 0.99 (0.92,1.05) | 1.00 (0.99,1.01) |
|  | Model 2 | 1.00 (Ref) | 1.02 (0.99,1.05) | 1.02 (0.98,1.06) | 1.02 (0.98,1.07) | 0.98 (0.92,1.04) | 1.00 (0.99,1.01) |
|  | Model 3 | 1.00 (Ref) | 1.01 (0.98,1.05) | 1.02 (0.98,1.06) | 1.01 (0.97,1.06) | 0.96 (0.90,1.03) | 1.00 (0.99,1.01) |
| Single | Model 1 | 1.00 (Ref) | 1.04 (0.98,1.10) | **1.07 (1.01,1.14)*** | 1.05 (0.98,1.13) | 1.06 (0.96,1.17) | 1.02 (1.00,1.03) |
|  | Model 2 | 1.00 (Ref) | 1.04 (0.98,1.10) | **1.08 (1.01,1.14)*** | 1.06 (0.99,1.15) | 1.07 (0.97,1.18) | 1.01 (0.99,1.02) |
|  | Model 3 | 1.00 (Ref) | 1.02 (0.96,1.08) | 1.05 (0.99,1.11) | 1.02 (0.95,1.09) | 0.98 (0.89,1.08) | 1.00 (0.99,1.02) |
| Educational attainment |  |  |  |  |  |  |  |
| Less than primary school | Model 1 | 1.00 (Ref) | 1.03 (0.99,1.08) | **1.06 (1.01,1.11)*** | 1.04 (0.98,1.09) | 1.01 (0.94,1.10) | 1.01 (1.00,1.02) |
|  | Model 2 | 1.00 (Ref) | 1.00 (0.96,1.04) | 1.01 (0.97,1.06) | 0.99 (0.94,1.05) | 0.95 (0.88,1.02) | 0.99 (0.98,1.01) |
|  | Model 3 | 1.00 (Ref) | 1.01 (0.96,1.05) | 1.01 (0.97,1.06) | 0.99 (0.94,1.05) | 0.93 (0.86,1.01) | 0.99 (0.98,1.01) |
| Primary school | Model 1 | 1.00 (Ref) | 1.02 (0.98,1.08) | 1.04 (0.98,1.09) | 1.05 (0.98,1.12) | 0.99 (0.90,1.09) | 1.01 (0.99,1.02) |
|  | Model 2 | 1.00 (Ref) | 1.02 (0.98,1.07) | 1.04 (0.99,1.10) | 1.05 (0.98,1.12) | 0.99 (0.91,1.08) | 1.01 (0.99,1.02) |
|  | Model 3 | 1.00 (Ref) | 1.02 (0.97,1.07) | 1.04 (0.99,1.10) | 1.04 (0.97,1.11) | 0.97 (0.88,1.07) | 1.01 (0.99,1.02) |
| High school and above | Model 1 | 1.00 (Ref) | 1.04 (0.99,1.09) | 1.04 (0.98,1.09) | 1.02 (0.95,1.08) | 1.09 (0.98,1.22) | 1.01 (0.99,1.03) |
|  | Model 2 | 1.00 (Ref) | 1.02 (0.97,1.07) | 1.03 (0.98,1.09) | 1.02 (0.96,1.09) | 1.06 (0.95,1.18) | 1.01 (0.99,1.03) |
|  | Model 3 | 1.00 (Ref) | 1.03 (0.98,1.08) | 1.05 (0.99,1.11) | 1.02 (0.95,1.10) | 1.03 (0.92,1.17) | 1.01 (0.99,1.03) |
| Occupation |  |  |  |  |  |  |  |
| Non–farmer | Model 1 | 1.00 (Ref) | 1.05 (0.97,1.14) | **1.10 (1.01,1.20)*** | 1.05 (0.95,1.15) | 0.99 (0.88,1.10) | 1.01 (0.98,1.03) |
|  | Model 2 | 1.00 (Ref) | 1.03 (0.96,1.11) | **1.09 (1.00,1.18)*** | 1.07 (0.98,1.16) | 0.99 (0.88,1.12) | 1.01 (0.99,1.04) |
|  | Model 3 | 1.00 (Ref) | 1.04 (0.96,1.12) | **1.09 (1.00,1.19)*** | 1.07 (0.98,1.17) | 1.00 (0.88,1.15) | 1.02 (0.99,1.04) |
| Farmer | Model 1 | 1.00 (Ref) | **1.03 (1.00,1.06)*** | **1.06 (1.02,1.09)**** | **1.05 (1.01,1.10)*** | 1.04 (0.98,1.10) | **1.01 (1.00,1.02)**** |
|  | Model 2 | 1.00 (Ref) | 1.01 (0.98,1.04) | 1.02 (0.99,1.05) | 1.01 (0.97,1.05) | 0.98 (0.93,1.03) | 1.00 (0.99,1.01) |
|  | Model 3 | 1.00 (Ref) | 1.01 (0.98,1.04) | 1.02 (0.99,1.05) | 1.01 (0.97,1.05) | 0.96 (0.90,1.02) | 1.00 (0.99,1.01) |
| Annual income |  |  |  |  |  |  |  |
| ＜10000 renminbi | Model 1 | 1.00 (Ref) | 1.00 (0.95,1.05) | 1.04 (0.99,1.10) | 1.04 (0.98,1.11) | 1.08 (0.99,1.17) | **1.02 (1.00,1.04)*** |
|  | Model 2 | 1.00 (Ref) | 1.00 (0.96,1.05) | 1.02 (0.97,1.07) | 1.03 (0.97,1.09) | 1.02 (0.94,1.11) | 1.01 (0.99,1.02) |
|  | Model 3 | 1.00 (Ref) | 1.00 (0.96,1.05) | 1.02 (0.97,1.08) | 1.02 (0.95,1.08) | 0.98 (0.89,1.08) | 1.00 (0.99,1.02) |
| 10000–29999 renminbi | Model 1 | 1.00 (Ref) | 1.01 (0.96,1.07) | 1.04 (0.97,1.10) | 1.00 (0.93,1.08) | 1.02 (0.92,1.13) | 1.01 (0.99,1.02) |
|  | Model 2 | 1.00 (Ref) | 0.99 (0.94,1.04) | 1.00 (0.95,1.06) | 0.97 (0.90,1.04) | 0.97 (0.88,1.05) | 0.99 (0.98,1.01) |
|  | Model 3 | 1.00 (Ref) | 0.99 (0.93,1.04) | 1.01 (0.95,1.07) | 0.95 (0.88,1.03) | 0.95 (0.86,1.05) | 0.99 (0.97,1.01) |
| ≥ 30000 renminbi | Model 1 | 1.00 (Ref) | **1.07 (1.02,1.12)**** | **1.07 (1.02,1.12)*** | **1.07 (1.01,1.14)*** | 0.96 (0.89,1.04) | 1.01 (0.99,1.02) |
|  | Model 2 | 1.00 (Ref) | **1.05 (1.00,1.10)*** | **1.06 (1.00,1.11)*** | 1.04 (0.99,1.11) | 0.94 (0.87,1.02) | 1.00 (0.99,1.01) |
|  | Model 3 | 1.00 (Ref) | **1.06 (1.01,1.11)*** | **1.06 (1.01,1.11)*** | **1.07 (1.00,1.13)*** | 0.96 (0.88,1.04) | 1.01 (0.99,1.02) |
| Cerebrovascular disease |  |  |  |  |  |  |  |
| No | Model 1 | 1.00 (Ref) | 1.03 (1.00,1.07) | 1.05 (1.02,1.09) | 1.04 (1.00,1.08) | 1.02 (0.96,1.08) | 1.01 (1.00,1.02) |
|  | Model 2 | 1.00 (Ref) | 1.01 (0.99,1.04) | 1.02 (0.99,1.06) | 1.02 (0.98,1.05) | 0.98 (0.93,1.03) | 1.00 (0.99,1.01) |
|  | Model 3 | 1.00 (Ref) | 1.01 (0.99,1.04) | 1.02 (0.99,1.06) | 1.01 (0.98,1.05) | 0.96 (0.91,1.01) | 1.00 (0.99,1.01) |
| Yes | Model 1 | 1.00 (Ref) | 0.96 (0.84,1.10) | 1.05 (0.90,1.23) | 0.97 (0.79,1.19) | 1.22 (0.94,1.59) | 1.03 (0.98,1.09) |
|  | Model 2 | 1.00 (Ref) | 0.96 (0.85,1.08) | 1.08 (0.94,1.24) | 0.96 (0.80,1.15) | 1.02 (0.81,1.29) | 1.02 (0.97,1.06) |
|  | Model 3 | 1.00 (Ref) | 0.96 (0.84,1.09) | 1.10 (0.96,1.26) | 0.96 (0.79,1.18) | 0.99 (0.75,1.31) | 1.02 (0.97,1.07) |
| Rheumatism |  |  |  |  |  |  |  |
| No | Model 1 | 1.00 (Ref) | **1.04 (1.00,1.07)*** | **1.05 (1.01,1.08)**** | **1.04 (1.00,1.09)*** | 1.03 (0.97,1.09) | 1.01 (1.00,1.02) |
|  | Model 2 | 1.00 (Ref) | 1.02 (0.99,1.05) | 1.03 (0.99,1.06) | 1.02 (0.98,1.06) | 0.99 (0.94,1.04) | 1.00 (0.99,1.01) |
|  | Model 3 | 1.00 (Ref) | 1.02 (0.99,1.05) | 1.03 (0.99,1.06) | 1.02 (0.98,1.06) | 0.97 (0.91,1.03) | 1.00 (0.99,1.01) |
| Yes | Model 1 | 1.00 (Ref) | 0.99 (0.91,1.08) | 1.10 (1.00,1.21) | 0.99 (0.86,1.14) | 1.00 (0.85,1.18) | 1.02 (0.98,1.05) |
|  | Model 2 | 1.00 (Ref) | 0.97 (0.90,1.04) | 1.04 (0.95,1.13) | 1.00 (0.88,1.12) | 0.91 (0.80,1.04) | 1.00 (0.97,1.03) |
|  | Model 3 | 1.00 (Ref) | 0.97 (0.90,1.05) | 1.05 (0.95,1.15) | 0.99 (0.88,1.11) | 0.89 (0.77,1.03) | 1.00 (0.97,1.03) |
| Osteoarthropathy |  |  |  |  |  |  |  |
| No | Model 1 | 1.00 (Ref) | 1.03 (0.99,1.06) | **1.04 (1.00,1.08)*** | 1.03 (0.98,1.07) | 1.02 (0.96,1.08) | 1.01 (1.00,1.02) |
|  | Model 2 | 1.00 (Ref) | 1.01 (0.97,1.04) | 1.01 (0.98,1.05) | 1.00 (0.96,1.04) | 0.97 (0.92,1.02) | 1.00 (0.99,1.01) |
|  | Model 3 | 1.00 (Ref) | 1.01 (0.98,1.04) | 1.01 (0.98,1.05) | 1.00 (0.96,1.04) | 0.96 (0.90,1.01) | 1.00 (0.99,1.01) |
| Yes | Model 1 | 1.00 (Ref) | 1.03 (0.97,1.10) | **1.10 (1.02,1.18)*** | 1.09 (0.97,1.23) | 1.05 (0.87,1.28) | **1.03 (1.01,1.06)*** |
|  | Model 2 | 1.00 (Ref) | 1.03 (0.96,1.09) | **1.09 (1.02,1.16)*** | 1.12 (0.99,1.25) | 1.05 (0.86,1.29) | **1.03 (1.01,1.06)*** |
|  | Model 3 | 1.00 (Ref) | 1.03 (0.96,1.09) | **1.09 (1.02,1.17)*** | 1.12 (0.98,1.27) | 1.04 (0.85,1.29) | **1.04 (1.01,1.07)*** |
| Smoking status |  |  |  |  |  |  |  |
| No | Model 1 | 1.00 (Ref) | **1.04 (1.01,1.07)*** | **1.06 (1.02,1.10)**** | 1.03 (0.99,1.08) | 1.03 (0.97,1.09) | 1.01 (1.00,1.02) |
|  | Model 2 | 1.00 (Ref) | 1.02 (0.99,1.05) | 1.03 (1.00,1.06) | 1.01 (0.97,1.05) | 0.99 (0.94,1.04) | 1.00 (0.99,1.01) |
|  | Model 3 | 1.00 (Ref) | 1.02 (0.99,1.05) | 1.03 (0.99,1.06) | 1.00 (0.96,1.04) | 0.96 (0.91,1.02) | 1.00 (0.99,1.01) |
| Yes | Model 1 | 1.00 (Ref) | 0.98 (0.92,1.05) | 1.00 (0.93,1.08) | 1.06 (0.96,1.17) | **0.89 (0.80,1.00)*** | 1.01 (0.98,1.03) |
|  | Model 2 | 1.00 (Ref) | 0.98 (0.92,1.04) | 1.00 (0.94,1.07) | 1.06 (0.98,1.15) | 0.92 (0.82,1.04) | 1.01 (0.99,1.03) |
|  | Model 3 | 1.00 (Ref) | 0.99 (0.93,1.05) | 1.02 (0.95,1.09) | **1.09 (1.00,1.19)*** | 0.94 (0.80,1.11) | 1.02 (0.99,1.04) |
| Alcohol consumption |  |  |  |  |  |  |  |
| No | Model 1 | 1.00 (Ref) | 1.05 (1.01,1.08) | **1.07 (1.03,1.10)**** | **1.06 (1.01,1.11)*** | 1.04 (0.98,1.11) | **1.01 (1.00,1.03)*** |
|  | Model 2 | 1.00 (Ref) | 1.01 (0.98,1.04) | 1.02 (0.99,1.06) | 1.02 (0.97,1.06) | 0.98 (0.93,1.04) | 1.00 (0.99,1.01) |
|  | Model 3 | 1.00 (Ref) | 1.02 (0.98,1.05) | 1.02 (0.99,1.06) | 1.01 (0.97,1.06) | 0.96 (0.90,1.02) | 1.00 (0.99,1.01) |
| Yes | Model 1 | 1.00 (Ref) | 1.01 (0.95,1.07) | 1.03 (0.96,1.10) | 0.99 (0.92,1.07) | 0.95 (0.86,1.06) | 1.00 (0.98,1.02) |
|  | Model 2 | 1.00 (Ref) | 1.01 (0.96,1.07) | 1.03 (0.97,1.10) | 1.02 (0.94,1.10) | 0.96 (0.88,1.04) | 1.00 (0.98,1.02) |
|  | Model 3 | 1.00 (Ref) | 1.01 (0.95,1.07) | 1.03 (0.97,1.10) | 1.00 (0.92,1.09) | 0.94 (0.85,1.04) | 1.00 (0.98,1.02) |
| Low grip strength |  |  |  |  |  |  |  |
| No | Model 1 | 1.00 (Ref) | 1.02 (0.98,1.07) | 1.04 (0.99,1.09) | 0.99 (0.94,1.04) | 1.05 (0.97,1.13) | 1.00 (0.99,1.02) |
|  | Model 2 | 1.00 (Ref) | 1.03 (0.99,1.07) | 1.04 (1.00,1.09) | 0.99 (0.94,1.05) | 1.04 (0.96,1.11) | 1.00 (0.99,1.02) |
|  | Model 3 | 1.00 (Ref) | 1.03 (0.98,1.07) | 1.04 (0.99,1.09) | 0.98 (0.92,1.03) | 1.01 (0.93,1.09) | 1.00 (0.98,1.01) |
| Yes | Model 1 | 1.00 (Ref) | 1.03 (0.99,1.07) | **1.06 (1.02,1.10)**** | **1.09 (1.03,1.14)**** | 1.02 (0.94,1.10) | **1.02 (1.01,1.03)**** |
|  | Model 2 | 1.00 (Ref) | 1.00 (0.97,1.04) | 1.02 (0.98,1.06) | 1.04 (0.99,1.09) | 0.95 (0.88,1.02) | 1.00 (0.99,1.02) |
|  | Model 3 | 1.00 (Ref) | 1.00 (0.97,1.04) | 1.02 (0.98,1.06) | 1.04 (0.99,1.09) | **0.91 (0.85,0.98)*** | 1.00 (0.99,1.01) |
| Anemia |  |  |  |  |  |  |  |
| No | Model 1 | 1.00 (Ref) | 1.03 (0.99,1.07) | **1.06 (1.02,1.11)*** | 1.03 (0.99,1.08) | 1.01 (0.95,1.08) | 1.01 (1.00,1.02) |
|  | Model 2 | 1.00 (Ref) | 1.01 (0.97,1.05) | 1.04 (1.00,1.08) | 1.01 (0.97,1.06) | 0.98 (0.92,1.04) | 1.00 (0.99,1.01) |
|  | Model 3 | 1.00 (Ref) | 1.01 (0.97,1.05) | 1.03 (0.99,1.07) | 1.00 (0.95,1.05) | **0.94 (0.88,1.00)*** | 1.00 (0.98,1.01) |
| Yes | Model 1 | 1.00 (Ref) | **1.05 (1.00,1.10)*** | 1.05 (1.00,1.11) | **1.09 (1.02,1.16)*** | **1.13 (1.02,1.25)*** | **1.02 (1.01,1.04)**** |
|  | Model 2 | 1.00 (Ref) | 1.02 (0.98,1.06) | 1.01 (0.97,1.07) | 1.03 (0.97,1.10) | 1.02 (0.93,1.12) | 1.01 (0.99,1.02) |
|  | Model 3 | 1.00 (Ref) | 1.02 (0.97,1.06) | 1.02 (0.97,1.07) | 1.03 (0.97,1.10) | 1.03 (0.93,1.14) | 1.01 (0.99,1.03) |
| Low density lipoprotein cholesterol | |  |  |  |  |  |  |
| Normal | Model 1 | 1.00 (Ref) | 1.01 (0.97,1.05) | **1.06 (1.02,1.11)**** | 1.04 (0.99,1.09) | 0.97 (0.90,1.05) | 1.01 (1.00,1.02) |
|  | Model 2 | 1.00 (Ref) | 0.99 (0.96,1.03) | 1.04 (1.00,1.08) | 1.01 (0.96,1.06) | **0.91 (0.85,0.98)*** | 1.00 (0.99,1.01) |
|  | Model 3 | 1.00 (Ref) | 0.99 (0.96,1.03) | 1.04 (1.00,1.08) | 1.01 (0.96,1.06) | **0.91 (0.85,0.98)*** | 1.00 (0.99,1.01) |
| High | Model 1 | 1.00 (Ref) | **1.06 (1.01,1.11)*** | **1.06 (1.01,1.11)*** | 1.05 (0.99,1.12) | **1.09 (1.00,1.19)*** | 1.01 (1.00,1.03) |
|  | Model 2 | 1.00 (Ref) | 1.03 (0.99,1.08) | 1.02 (0.97,1.07) | 1.02 (0.97,1.08) | 1.04 (0.96,1.13) | 1.00 (0.99,1.02) |
|  | Model 3 | 1.00 (Ref) | 1.04 (0.99,1.08) | 1.02 (0.97,1.07) | 1.02 (0.96,1.08) | 1.03 (0.95,1.12) | 1.00 (0.99,1.02) |
| Total cholesterol |  |  |  |  |  |  |  |
| Normal | Model 1 | 1.00 (Ref) | 1.02 (0.97,1.06) | **1.05 (1.00,1.10)*** | 1.05 (0.99,1.12) | 0.97 (0.90,1.06) | 1.01 (1.00,1.03) |
|  | Model 2 | 1.00 (Ref) | 0.98 (0.94,1.02) | 1.03 (0.98,1.07) | 1.01 (0.96,1.07) | **0.90 (0.84,0.97)**** | 1.00 (0.98,1.01) |
|  | Model 3 | 1.00 (Ref) | 0.98 (0.95,1.02) | 1.03 (0.98,1.07) | 1.01 (0.96,1.07) | **0.89 (0.83,0.96)**** | 1.00 (0.99,1.01) |
| High | Model 1 | 1.00 (Ref) | **1.05 (1.00,1.09)*** | **1.05 (1.01,1.10)*** | 1.04 (0.98,1.09) | 1.07 (0.99,1.15) | 1.01 (1.00,1.02) |
|  | Model 2 | 1.00 (Ref) | 1.04 (1.00,1.08) | 1.03 (0.99,1.07) | 1.02 (0.97,1.07) | 1.04 (0.97,1.12) | 1.00 (0.99,1.02) |
|  | Model 3 | 1.00 (Ref) | 1.03 (0.99,1.07) | 1.02 (0.98,1.06) | 1.00 (0.95,1.05) | 0.99 (0.92,1.07) | 1.00 (0.99,1.01) |
| Serum creatinine |  |  |  |  |  |  |  |
| Normal | Model 1 | 1.00 (Ref) | **1.04 (1.00,1.07)*** | **1.06 (1.03,1.10)**** | 1.04 (0.99,1.08) | 1.04 (0.98,1.11) | **1.01 (1.00,1.02)*** |
|  | Model 2 | 1.00 (Ref) | 1.01 (0.98,1.05) | **1.03 (1.00,1.07)*** | 1.01 (0.97,1.06) | 0.99 (0.94,1.05) | 1.00 (0.99,1.01) |
|  | Model 3 | 1.00 (Ref) | 1.01 (0.98,1.05) | 1.03 (1.00,1.07) | 1.01 (0.97,1.06) | 0.98 (0.92,1.04) | 1.00 (0.99,1.01) |
| Low | Model 1 | 1.00 (Ref) | 1.01 (0.94,1.07) | 1.01 (0.94,1.08) | 1.04 (0.96,1.13) | 0.99 (0.87,1.13) | 1.01 (0.98,1.03) |
|  | Model 2 | 1.00 (Ref) | 1.00 (0.94,1.06) | 0.99 (0.93,1.06) | 1.01 (0.94,1.10) | 0.94 (0.83,1.07) | 1.00 (0.97,1.02) |
|  | Model 3 | 1.00 (Ref) | 1.01 (0.95,1.07) | 0.99 (0.92,1.06) | 1.00 (0.92,1.09) | 0.88 (0.76,1.01) | 0.99 (0.97,1.01) |
| Uric acid |  |  |  |  |  |  |  |
| Normal | Model 1 | 1.00 (Ref) | 1.03 (0.99,1.06) | **1.05 (1.01,1.08)*** | 1.02 (0.98,1.07) | 1.00 (0.94,1.07) | 1.01 (1.00,1.02) |
|  | Model 2 | 1.00 (Ref) | 1.00 (0.97,1.03) | 1.01 (0.98,1.05) | 0.99 (0.95,1.03) | 0.94 (0.89,1.00) | 0.99 (0.98,1.00) |
|  | Model 3 | 1.00 (Ref) | 1.01 (0.98,1.04) | 1.02 (0.99,1.06) | 1.00 (0.96,1.04) | **0.93 (0.87,0.99)*** | 1.00 (0.99,1.01) |
| High | Model 1 | 1.00 (Ref) | **1.09 (1.00,1.19)*** | **1.13 (1.03,1.23)**** | **1.15 (1.04,1.26)**** | **1.14 (1.02,1.28)*** | **1.03 (1.01,1.05)*** |
|  | Model 2 | 1.00 (Ref) | 1.08 (0.99,1.17) | **1.10 (1.01,1.19)*** | **1.12 (1.03,1.22)*** | 1.07 (0.97,1.19) | 1.02 (1.00,1.04) |
|  | Model 3 | 1.00 (Ref) | 1.08 (0.99,1.17) | **1.10 (1.01,1.19)*** | **1.11 (1.01,1.21)*** | 1.09 (0.98,1.21) | 1.02 (1.00,1.04) |
| Aspartate aminotransferase |  |  |  |  |  |  |  |
| Normal | Model 1 | 1.00 (Ref) | **1.04 (1.00,1.07)*** | **1.07 (1.03,1.10)**** | **1.05 (1.00,1.10)*** | 1.02 (0.96,1.09) | **1.01 (1.00,1.02)*** |
|  | Model 2 | 1.00 (Ref) | 1.02 (0.99,1.05) | **1.03 (1.00,1.07)*** | 1.01 (0.97,1.06) | 0.97 (0.91,1.03) | 1.00 (0.99,1.01) |
|  | Model 3 | 1.00 (Ref) | 1.02 (0.99,1.05) | **1.04 (1.00,1.07)*** | 1.01 (0.97,1.05) | 0.95 (0.89,1.01) | 1.00 (0.99,1.01) |
| High | Model 1 | 1.00 (Ref) | 1.00 (0.94,1.07) | 1.00 (0.94,1.08) | 1.01 (0.93,1.09) | 1.05 (0.94,1.18) | 1.01 (0.99,1.03) |
|  | Model 2 | 1.00 (Ref) | 0.99 (0.93,1.06) | 0.99 (0.92,1.06) | 1.01 (0.93,1.09) | 1.01 (0.91,1.12) | 1.00 (0.98,1.02) |
|  | Model 3 | 1.00 (Ref) | 1.00 (0.93,1.06) | 1.00 (0.93,1.07) | 1.02 (0.94,1.11) | 1.03 (0.91,1.16) | 1.01 (0.98,1.03) |
| Alanine aminotransferase |  |  |  |  |  |  |  |
| Normal | Model 1 | 1.00 (Ref) | **1.03 (1.00,1.06)*** | **1.06 (1.03,1.09)**** | **1.04 (1.00,1.09)*** | 1.02 (0.96,1.08) | **1.01 (1.00,1.02)*** |
|  | Model 2 | 1.00 (Ref) | 1.01 (0.98,1.04) | 1.03 (1.00,1.06) | 1.01 (0.97,1.05) | 0.97 (0.92,1.03) | 1.00 (0.99,1.01) |
|  | Model 3 | 1.00 (Ref) | 1.01 (0.99,1.04) | 1.03 (1.00,1.06) | 1.01 (0.97,1.05) | 0.95 (0.90,1.01) | 1.00 (0.99,1.01) |
| High | Model 1 | 1.00 (Ref) | 0.96 (0.83,1.12) | 0.94 (0.81,1.08) | 1.02 (0.88,1.18) | 1.10 (0.90,1.33) | 1.02 (0.98,1.06) |
|  | Model 2 | 1.00 (Ref) | 0.95 (0.82,1.11) | 0.95 (0.82,1.09) | 1.03 (0.89,1.20) | 1.03 (0.86,1.23) | 1.02 (0.98,1.06) |
|  | Model 3 | 1.00 (Ref) | 0.96 (0.82,1.13) | 0.93 (0.80,1.09) | 1.01 (0.86,1.19) | 1.01 (0.81,1.25) | 1.01 (0.97,1.05) |

**Notes:** *, *P*<0.05; **, *P*<0.01.Model 1 was unadjusted model; Model 2 adjusted for sex, age, ethnic, marital status, educational attainment, occupation, annual income, cerebrovascular disease, rheumatism, osteoarthropathy, smoking status, and alcohol consumption; Model 3 further adjusted hand grip strength, anemia, total cholesterol, low density lipoprotein cholesterol, aspartate aminotransferase, alanine aminotransferasee, serum creatinine, and uric acid.

**Abbreviations:** MetS, metabolic syndrome; IADL, instrumental activities of daily living; PR, prevalence ratio; CI, confidence interval.

**Supplementary Table S10.** Subgroup analysis of associations between the number of MetS components and comorbid ADL­IADL disability

| Variables | Models | The number of MetS components [PR(95% CI)] | | | | | PR (95% CI) for trend |
| --- | --- | --- | --- | --- | --- | --- | --- |
|  |  | 0 | 1 | 2 | 3 | ≥4 |  |
| Gender |  |  |  |  |  |  |  |
| Male | Model 1 | 1.00 (Ref) | 1.49 (0.86,2.60) | **1.93 (1.09,3.41)*** | 1.80 (0.91,3.56) | 1.76 (0.66,4.65) | **1.18 (1.02,1.36)*** |
|  | Model 2 | 1.00 (Ref) | 1.31 (0.76,2.26) | **1.83 (1.06,3.18)*** | **2.07 (1.08,3.99)*** | 2.43 (0.94,6.28) | **1.28 (1.10,1.49)**** |
|  | Model 3 | 1.00 (Ref) | 1.23 (0.71,2.11) | **1.78 (1.02,3.10)*** | **2.09 (1.06,4.10)*** | 1.28 (0.29,5.60) | **1.25 (1.05,1.49)*** |
| Female | Model 1 | 1.00 (Ref) | 1.28 (0.82,2.00) | 1.55 (0.98,2.44) | **2.43 (1.52,3.88)***** | **2.06 (1.12,3.79)*** | **1.26 (1.13,1.41)***** |
|  | Model 2 | 1.00 (Ref) | 1.06 (0.67,1.67) | 1.24 (0.78,1.97) | **1.94 (1.19,3.16)**** | 1.30 (0.70,2.40) | **1.17 (1.05,1.31)**** |
|  | Model 3 | 1.00 (Ref) | 1.27 (0.79,2.05) | 1.46 (0.89,2.39) | **2.28 (1.34,3.87)**** | 1.55 (0.77,3.10) | **1.20 (1.07,1.36)**** |
| Age group |  |  |  |  |  |  |  |
| 60–69 years | Model 1 | 1.00 (Ref) | 0.92 (0.52,1.63) | 0.94 (0.50,1.74) | 1.60 (0.84,3.05) | 1.08 (0.37,3.11) | 1.11 (0.92,1.34) |
|  | Model 2 | 1.00 (Ref) | 0.95 (0.53,1.69) | 0.91 (0.49,1.70) | 1.69 (0.89,3.23) | 1.05 (0.36,3.07) | 1.11 (0.92,1.34) |
|  | Model 3 | 1.00 (Ref) | 0.95 (0.53,1.68) | 0.90 (0.48,1.68) | 1.56 (0.80,3.05) | 0.85 (0.25,2.87) | 1.07 (0.88,1.30) |
| ≥ 70 years | Model 1 | 1.00 (Ref) | 1.45 (0.93,2.27) | **1.96 (1.25,3.08)**** | **2.41 (1.49,3.90)***** | **2.26 (1.24,4.12)**** | **1.26 (1.14,1.39)***** |
|  | Model 2 | 1.00 (Ref) | 1.52 (0.97,2.38) | **2.12 (1.34,3.34)**** | **2.71 (1.66,4.42)***** | **2.36 (1.26,4.40)**** | **1.29 (1.16,1.42)***** |
|  | Model 3 | 1.00 (Ref) | **1.69 (1.05,2.73)*** | **2.45 (1.51,3.98)***** | **3.25 (1.92,5.51)***** | **2.58 (1.26,5.30)*** | **1.34 (1.20,1.50)***** |
| Ethnic |  |  |  |  |  |  |  |
| Non–zhuang | Model 1 | 1.00 (Ref) | 0.75 (0.36,1.56) | 1.15 (0.56,2.36) | 1.59 (0.76,3.29) | 0.63 (0.18,2.19) | 1.11 (0.92,1.34) |
|  | Model 2 | 1.00 (Ref) | 0.72 (0.35,1.50) | 1.02 (0.49,2.13) | 1.62 (0.75,3.50) | 0.69 (0.18,2.66) | 1.14 (0.93,1.39) |
|  | Model 3 | 1.00 (Ref) | 0.89 (0.36,2.16) | 1.32 (0.56,3.15) | 1.55 (0.62,3.90) | 0.82 (0.18,3.68) | 1.12 (0.90,1.39) |
| Zhuang | Model 1 | 1.00 (Ref) | 1**.57 (1.06,2.33)*** | **1.85 (1.23,2.79)**** | **2.35 (1.51,3.68)***** | **2.44 (1.37,4.36)**** | **1.25 (1.13,1.38)***** |
|  | Model 2 | 1.00 (Ref) | 1.26 (0.85,1.87) | **1.51 (1.01,2.26)*** | **2.00 (1.28,3.12)**** | **1.92 (1.11,3.33)*** | **1.21 (1.09,1.34)***** |
|  | Model 3 | 1.00 (Ref) | 1.28 (0.87,1.89) | **1.57 (1.05,2.35)*** | **2.17 (1.37,3.43)**** | 1.70 (0.89,3.24) | **1.22 (1.09,1.36)***** |
| Marital status |  |  |  |  |  |  |  |
| Partnered | Model 1 | 1.00 (Ref) | 1.36 (0.87,2.12) | **1.68 (1.06,2.67)*** | **2.19 (1.34,3.60)**** | 0.93 (0.36,2.38) | **1.16 (1.04,1.31)**** |
|  | Model 2 | 1.00 (Ref) | 1.34 (0.86,2.09) | **1.69 (1.06,2.68)*** | **2.21 (1.34,3.63)**** | 0.92 (0.36,2.36) | **1.19 (1.05,1.35)**** |
|  | Model 3 | 1.00 (Ref) | 1.26 (0.81,1.95) | **1.59 (1.00,2.51)*** | **2.30 (1.37,3.87)**** | 0.95 (0.35,2.61) | **1.20 (1.05,1.37)**** |
| Single | Model 1 | 1.00 (Ref) | 1.27 (0.73,2.21) | 1.50 (0.86,2.63) | **2.14 (1.18,3.88)*** | **2.63 (1.34,5.16)**** | **1.28 (1.12,1.46)***** |
|  | Model 2 | 1.00 (Ref) | 1.32 (0.75,2.34) | 1.62 (0.91,2.88) | **2.30 (1.25,4.24)**** | **2.82 (1.42,5.61)**** | **1.23 (1.07,1.41)**** |
|  | Model 3 | 1.00 (Ref) | 1.19 (0.65,2.16) | 1.48 (0.80,2.73) | **1.96 (1.00,3.85)*** | 2.29 (1.00,5.26) | **1.25 (1.07,1.46)**** |
| Educational attainment |  |  |  |  |  |  |  |
| Less than primary school | Model 1 | 1.00 (Ref) | 1.29 (0.80,2.08) | **1.77 (1.09,2.87)*** | **2.29 (1.36,3.83)**** | **2.09 (1.06,4.09)*** | **1.26 (1.12,1.42)***** |
|  | Model 2 | 1.00 (Ref) | 1.03 (0.63,1.67) | 1.26 (0.77,2.05) | **1.79 (1.04,3.06)*** | 1.46 (0.75,2.85) | **1.18 (1.04,1.34)**** |
|  | Model 3 | 1.00 (Ref) | 1.13 (0.69,1.84) | 1.40 (0.85,2.29) | **2.01 (1.16,3.50)*** | 1.59 (0.76,3.29) | **1.21 (1.06,1.38)**** |
| Primary school | Model 1 | 1.00 (Ref) | 1.68 (0.90,3.11) | **2.00 (1.05,3.79)*** | **2.79 (1.41,5.52)**** | 1.44 (0.48,4.32) | **1.22 (1.05,1.41)**** |
|  | Model 2 | 1.00 (Ref) | 1.55 (0.84,2.84) | **2.03 (1.08,3.80)*** | **2.72 (1.39,5.34)**** | 1.58 (0.52,4.78) | **1.25 (1.08,1.45)**** |
|  | Model 3 | 1.00 (Ref) | 1.72 (0.89,3.34) | **2.23 (1.13,4.41)*** | **3.04 (1.43,6.45)**** | 1.42 (0.40,5.10) | **1.25 (1.06,1.48)**** |
| High school and above | Model 1 | 1.00 (Ref) | 1.02 (0.43,2.44) | 0.90 (0.34,2.36) | 1.21 (0.42,3.50) | 2.44 (0.75,7.90) | 1.15 (0.86,1.55) |
|  | Model 2 | 1.00 (Ref) | 0.85 (0.35,2.06) | 0.86 (0.32,2.32) | 1.43 (0.49,4.15) | 1.59 (0.45,5.67) | 1.15 (0.85,1.55) |
|  | Model 3 | 1.00 (Ref) | 0.90 (0.37,2.22) | 0.99 (0.37,2.65) | 1.42 (0.43,4.63) | 1.80 (0.24,13.79) | 1.13 (0.79,1.63) |
| Occupation |  |  |  |  |  |  |  |
| Non–farmer | Model 1 | 1.00 (Ref) | 0.58 (0.10,3.37) | 1.67 (0.37,7.58) | 1.67 (0.34,8.24) | 1.14 (0.11,11.92) | 1.26 (0.87,1.84) |
|  | Model 2 | 1.00 (Ref) | 0.67 (0.11,4.10) | 1.48 (0.30,7.19) | 2.69 (0.43,16.67) | 2.04 (0.17,24.86) | 1.48 (0.90,2.43) |
|  | Model 3 | 1.00 (Ref) | 0.66 (0.13,3.28) | 1.47 (0.34,6.39) | 2.83 (0.56,14.33) | 2.86 (0.28,28.86) | 1.57 (0.99,2.50) |
| Farmer | Model 1 | 1.00 (Ref) | **1.46 (1.02,2.09)*** | **1.76 (1.21,2.54)**** | **2.41 (1.62,3.59)***** | **2.14 (1.26,3.62)**** | **1.25 (1.14,1.36)***** |
|  | Model 2 | 1.00 (Ref) | 1.18 (0.82,1.68) | 1.40 (0.97,2.02) | **1.93 (1.29,2.88)**** | 1.59 (0.94,2.68) | **1.19 (1.08,1.30)***** |
|  | Model 3 | 1.00 (Ref) | 1.27 (0.87,1.83) | **1.55 (1.06,2.26)*** | **2.12 (1.38,3.26)**** | 1.50 (0.82,2.78) | **1.20 (1.08,1.33)**** |
| Annual income |  |  |  |  |  |  |  |
| ＜10000 renminbi | Model 1 | 1.00 (Ref) | 1.48 (0.88,2.49) | **2.06 (1.22,3.48)**** | **2.45 (1.38,4.34)**** | **2.23 (1.06,4.67)*** | 1.26 (1.12,1.43) |
|  | Model 2 | 1.00 (Ref) | 1.25 (0.75,2.10) | **1.70 (1.01,2.85)*** | **2.17 (1.22,3.86)**** | 1.70 (0.81,3.56) | **1.22 (1.07,1.38)**** |
|  | Model 3 | 1.00 (Ref) | 1.40 (0.82,2.39) | **1.83 (1.05,3.17)*** | **2.43 (1.30,4.54)**** | 1.47 (0.59,3.69) | **1.22 (1.06,1.41)**** |
| 10000–29999 renminbi | Model 1 | 1.00 (Ref) | 0.75 (0.38,1.48) | 1.22 (0.63,2.37) | 1.55 (0.73,3.28) | 2.19 (0.92,5.23) | **1.27 (1.04,1.56)*** |
|  | Model 2 | 1.00 (Ref) | 0.74 (0.37,1.48) | 1.10 (0.55,2.18) | 1.49 (0.69,3.21) | 1.60 (0.67,3.85) | 1.21 (0.99,1.48) |
|  | Model 3 | 1.00 (Ref) | 0.80 (0.38,1.69) | 1.39 (0.67,2.91) | 1.80 (0.78,4.19) | 1.64 (0.59,4.55) | **1.25 (1.01,1.55)*** |
| ≥ 30000 renminbi | Model 1 | 1.00 (Ref) | 1.79 (0.91,3.53) | 1.65 (0.80,3.39) | 2.79 (1.35,5.77) | 1.25 (0.35,4.40) | **1.18 (1.01,1.39)*** |
|  | Model 2 | 1.00 (Ref) | 1.43 (0.73,2.81) | 1.34 (0.65,2.77) | **2.24 (1.06,4.70)*** | 1.27 (0.35,4.59) | **1.17 (0.98,1.40)** |
|  | Model 3 | 1.00 (Ref) | 1.43 (0.73,2.82) | 1.55 (0.75,3.17) | **2.51 (1.16,5.46)*** | 1.70 (0.48,6.09) | **1.24 (1.03,1.49)*** |
| Cerebrovascular disease |  |  |  |  |  |  |  |
| No | Model 1 | 1.00 (Ref) | 1.32 (0.93,1.88) | **1.64 (1.14,2.36)**** | **2.19 (1.49,3.23)***** | **1.82 (1.07,3.11)*** | **1.22 (1.12,1.34)***** |
|  | Model 2 | 1.00 (Ref) | 1.11 (0.77,1.58) | 1.38 (0.96,1.98) | **1.91 (1.28,2.84)**** | 1.48 (0.86,2.53) | **1.19 (1.08,1.31)***** |
|  | Model 3 | 1.00 (Ref) | 1.19 (0.82,1.72) | **1.51 (1.04,2.19)*** | **2.07 (1.36,3.15)**** | 1.40 (0.74,2.64) | **1.20 (1.08,1.33)***** |
| Yes | Model 1 | 1.00 (Ref) | 2.89 (0.37,22.26) | 3.65 (0.45,29.84) | 4.37 (0.43,44.81) | **10.00 (1.04,95.75)*** | **1.52 (1.05,2.21)*** |
|  | Model 2 | 1.00 (Ref) | 2.16 (0.21,22.17) | 3.54 (0.32,38.95) | 5.88 (0.39,88.64) | 4.68 (0.42,52.15) | 1.46 (0.98,2.17) |
|  | Model 3 | 1.00 (Ref) | 3.39 (0.37,31.35) | 5.40 (0.48,61.31) | **20.92 (1.37,320.48)*** | 3.97 (0.43,36.65) | **1.56 (1.02,2.39)*** |
| Rheumatism |  |  |  |  |  |  |  |
| No | Model 1 | 1.00 (Ref) | 1.35 (0.94,1.95) | **1.64 (1.12,2.38)*** | **2.17 (1.46,3.25)***** | **1.98 (1.15,3.40)*** | **1.23 (1.12,1.35)***** |
|  | Model 2 | 1.00 (Ref) | 1.16 (0.80,1.68) | 1.44 (0.99,2.09) | **1.94 (1.29,2.92)**** | **1.78 (1.05,3.04)*** | **1.21 (1.10,1.34)***** |
|  | Model 3 | 1.00 (Ref) | 1.22 (0.84,1.79) | **1.54 (1.05,2.25)*** | **2.08 (1.36,3.20)**** | 1.69 (0.91,3.14) | **1.22 (1.10,1.36)***** |
| Yes | Model 1 | 1.00 (Ref) | 1.49 (0.52,4.31) | 2.26 (0.75,6.86) | 3.33 (0.95,11.66) | 2.10 (0.41,10.69) | **1.32 (1.02,1.70)*** |
|  | Model 2 | 1.00 (Ref) | 1.06 (0.43,2.61) | 1.23 (0.44,3.46) | **3.82 (1.19,12.25)*** | 0.39 (0.05,2.76) | 1.10 (0.83,1.45) |
|  | Model 3 | 1.00 (Ref) | 1.21 (0.40,3.69) | 1.48 (0.40,5.43) | 3.54 (0.79,15.74) | 0.53 (0.09,3.10) | 1.14 (0.84,1.55) |
| Osteoarthropathy |  |  |  |  |  |  |  |
| No | Model 1 | 1.00 (Ref) | 1.35 (0.92,1.99) | **1.65 (1.11,2.46)*** | **2.10 (1.38,3.20)**** | **2.00 (1.16,3.44)*** | **1.22 (1.11,1.34)***** |
|  | Model 2 | 1.00 (Ref) | 1.09 (0.74,1.60) | 1.34 (0.90,1.99) | **1.76 (1.15,2.70)**** | 1.56 (0.91,2.67) | **1.18 (1.07,1.30)**** |
|  | Model 3 | 1.00 (Ref) | 1.18 (0.79,1.75) | 1.47 (0.97,2.21) | **1.89 (1.20,2.97)**** | 1.44 (0.77,2.69) | **1.18 (1.06,1.31)**** |
| Yes | Model 1 | 1.00 (Ref) | 1.38 (0.61,3.13) | 1.86 (0.82,4.20) | **3.77 (1.45,9.77)**** | 1.50 (0.20,11.15) | **1.36 (1.06,1.75)*** |
|  | Model 2 | 1.00 (Ref) | 1.20 (0.53,2.75) | 1.61 (0.74,3.52) | **3.91 (1.59,9.61)**** | 1.23 (0.14,10.99) | **1.35 (1.05,1.75)*** |
|  | Model 3 | 1.00 (Ref) | 1.25 (0.55,2.80) | 1.87 (0.87,4.01) | **5.89 (2.47,14.02)***** | 2.10 (0.35,12.63) | **1.53 (1.18,1.99)**** |
| Smoking status |  |  |  |  |  |  |  |
| No | Model 1 | 1.00 (Ref) | 1.32 (0.91,1.91) | **1.67 (1.14,2.43)**** | **2.09 (1.39,3.15)***** | **2.08 (1.23,3.51)**** | **1.23 (1.12,1.35)***** |
|  | Model 2 | 1.00 (Ref) | 1.07 (0.73,1.55) | 1.38 (0.94,2.02) | **1.83 (1.21,2.78)*** | 1.59 (0.94,2.68) | **1.20 (1.08,1.32)***** |
|  | Model 3 | 1.00 (Ref) | 1.14 (0.78,1.67) | **1.53 (1.03,2.25)*** | **1.99 (1.28,3.09)**** | 1.59 (0.87,2.92) | **1.21 (1.09,1.35)***** |
| Yes | Model 1 | 1.00 (Ref) | 1.67 (0.63,4.40) | 1.82 (0.66,5.04) | **3.33 (1.16,9.56)*** | 0.00 (0.00,0.00) | 1.23 (0.96,1.57) |
|  | Model 2 | 1.00 (Ref) | 1.64 (0.63,4.26) | 1.54 (0.57,4.15) | **3.39 (1.13,10.17)*** | 0.00 (0.00,0.00) | 1.25 (0.95,1.64) |
|  | Model 3 | 1.00 (Ref) | 2.03 (0.70,5.86) | 2.44 (0.74,8.09) | **4.80 (1.60,14.42)**** | 0.00 (0.00,0.00) | **1.41 (1.07,1.86)*** |
| Alcohol consumption |  |  |  |  |  |  |  |
| No | Model 1 | 1.00 (Ref) | 1.35 (0.93,1.96) | **1.62 (1.10,2.38)*** | **2.45 (1.64,3.67)***** | **2.09 (1.22,3.59)**** | **1.26 (1.15,1.39)***** |
|  | Model 2 | 1.00 (Ref) | 1.05 (0.72,1.54) | 1.34 (0.91,1.97) | **2.06 (1.36,3.11)**** | 1.57 (0.91,2.71) | **1.22 (1.11,1.35)***** |
|  | Model 3 | 1.00 (Ref) | 1.11 (0.76,1.63) | 1.43 (0.97,2.12) | **2.19 (1.42,3.38)***** | 1.48 (0.80,2.74) | **1.23 (1.10,1.37)***** |
| Yes | Model 1 | 1.00 (Ref) | 1.65 (0.64,4.26) | 2.25 (0.87,5.84) | 1.26 (0.37,4.25) | 1.47 (0.30,7.32) | 1.08 (0.87,1.34) |
|  | Model 2 | 1.00 (Ref) | 1.84 (0.72,4.74) | 2.31 (0.89,5.99) | 1.74 (0.50,6.11) | 1.24 (0.30,5.14) | 1.10 (0.89,1.36) |
|  | Model 3 | 1.00 (Ref) | 2.04 (0.70,5.97) | 2.81 (0.95,8.33) | 1.80 (0.43,7.42) | 1.29 (0.12,13.57) | 1.17 (0.90,1.51) |
| Low grip strength |  |  |  |  |  |  |  |
| No | Model 1 | 1.00 (Ref) | 0.85 (0.45,1.62) | 1.14 (0.59,2.19) | 1.41 (0.69,2.86) | 1.44 (0.57,3.66) | 1.15 (0.95,1.39) |
|  | Model 2 | 1.00 (Ref) | 0.84 (0.43,1.66) | 1.04 (0.53,2.04) | 1.36 (0.64,2.86) | 1.15 (0.44,2.97) | 1.11 (0.91,1.34) |
|  | Model 3 | 1.00 (Ref) | 0.86 (0.45,1.66) | 1.01 (0.53,1.95) | 1.33 (0.62,2.86) | 1.07 (0.39,2.89) | 1.09 (0.88,1.33) |
| Yes | Model 1 | 1.00 (Ref) | **1.60 (1.05,2.45)*** | **2.02 (1.31,3.13)**** | **2.79 (1.75,4.46)***** | **2.42 (1.29,4.56)**** | **1.29 (1.17,1.43)***** |
|  | Model 2 | 1.00 (Ref) | 1.33 (0.87,2.03) | **1.76 (1.15,2.70)***** | **2.37 (1.48,3.79)***** | 1.88 (0.99,3.55) | **1.25 (1.12,1.39)***** |
|  | Model 3 | 1.00 (Ref) | 1.45 (0.94,2.24) | **1.92 (1.23,2.99)**** | **2.65 (1.62,4.33)***** | 1.74 (0.84,3.59) | **1.26 (1.13,1.41)***** |
| Anemia |  |  |  |  |  |  |  |
| No | Model 1 | 1.00 (Ref) | **2.16 (1.21,3.86)**** | **2.31 (1.27,4.18)**** | **2.91 (1.56,5.43)**** | **2.46 (1.13,5.38)*** | **1.21 (1.07,1.36)**** |
|  | Model 2 | 1.00 (Ref) | **1.84 (1.03,3.29)*** | **1.92 (1.06,3.47)*** | **2.60 (1.40,4.86)**** | **1.94 (0.87,4.34)** | **1.18 (1.04,1.33)**** |
|  | Model 3 | 1.00 (Ref) | **1.98 (1.09,3.60)*** | **2.09 (1.13,3.85)*** | **2.91 (1.51,5.61)**** | 1.75 (0.70,4.38) | **1.19 (1.05,1.36)**** |
| Yes | Model 1 | 1.00 (Ref) | 1.04 (0.66,1.63) | **1.63 (1.03,2.57)*** | **2.38 (1.44,3.94)***** | **2.50 (1.24,5.00)*** | **1.35 (1.18,1.54)***** |
|  | Model 2 | 1.00 (Ref) | 0.81 (0.51,1.29) | 1.33 (0.83,2.13) | **1.82 (1.04,3.17)*** | 1.79 (0.92,3.50) | **1.27 (1.10,1.47)**** |
|  | Model 3 | 1.00 (Ref) | 0.84 (0.53,1.33) | 1.40 (0.87,2.24) | 1.74 (0.98,3.10) | 1.69 (0.78,3.67) | **1.25 (1.07,1.46)**** |
| Low density lipoprotein cholesterol |  |  |  |  |  |  |  |
| Normal | Model 1 | 1.00 (Ref) | 1.25 (0.80,1.93) | **1.84 (1.18,2.88)**** | **2.47 (1.53,3.98)***** | 1.61 (0.75,3.43) | **1.27 (1.13,1.42)***** |
|  | Model 2 | 1.00 (Ref) | 0.98 (0.64,1.52) | 1.37 (0.88,2.14) | **1.96 (1.20,3.20)**** | 1.25 (0.62,2.54) | **1.21 (1.07,1.37)**** |
|  | Model 3 | 1.00 (Ref) | 1.03 (0.66,1.60) | 1.54 (0.98,2.41) | **2.13 (1.29,3.51)**** | 1.30 (0.60,2.78) | **1.24 (1.09,1.40)**** |
| High | Model 1 | 1.00 (Ref) | 1.63 (0.90,2.93) | 1.61 (0.88,2.97) | **2.24 (1.17,4.31)**** | 2.17 (0.93,5.05) | **1.19 (1.03,1.38)*** |
|  | Model 2 | 1.00 (Ref) | 1.48 (0.81,2.72) | 1.59 (0.86,2.96) | **2.13 (1.08,4.19)*** | 1.84 (0.75,4.52) | **1.18 (1.02,1.37)*** |
|  | Model 3 | 1.00 (Ref) | 1.55 (0.82,2.91) | 1.61 (0.84,3.10) | **2.19 (1.04,4.62)*** | 1.87 (0.73,4.76) | 1.18 (1.00,1.39) |
| Total cholesterol |  |  |  |  |  |  |  |
| Normal | Model 1 | 1.00 (Ref) | 1.01 (0.64,1.61) | **1.67 (1.05,2.64)*** | **1.98 (1.17,3.34)*** | 1.40 (0.63,3.12) | **1.23 (1.08,1.40)**** |
|  | Model 2 | 1.00 (Ref) | 0.75 (0.48,1.19) | 1.23 (0.78,1.96) | 1.54 (0.90,2.62) | 1.02 (0.48,2.18) | **1.17 (1.02,1.35)*** |
|  | Model 3 | 1.00 (Ref) | 0.81 (0.51,1.28) | 1.30 (0.82,2.07) | 1.65 (0.98,2.79) | 1.16 (0.55,2.46) | **1.22 (1.05,1.40)**** |
| High | Model 1 | 1.00 (Ref) | **1.93 (1.12,3.33)*** | **1.88 (1.07,3.31)*** | **2.79 (1.56,5.01)**** | **2.86 (1.40,5.85)**** | **1.25 (1.10,1.41)***** |
|  | Model 2 | 1.00 (Ref) | **1.77 (1.01,3.08)*** | **1.78 (1.00,3.14)*** | **2.63 (1.44,4.80)**** | **2.56 (1.23,5.32)*** | **1.23 (1.09,1.40)**** |
|  | Model 3 | 1.00 (Ref) | 1.71 (0.98,2.99) | 1.64 (0.92,2.93) | **2.53 (1.35,4.77)*** | 1.73 (0.73,4.12) | **1.20 (1.04,1.39)*** |
| Serum creatinine |  |  |  |  |  |  |  |
| Normal | Model 1 | 1.00 (Ref) | 1.37 (0.91,2.06) | **1.81 (1.20,2.74)**** | **2.29 (1.47,3.59)***** | **2.25 (1.27,3.98)**** | **1.26 (1.14,1.39)***** |
|  | Model 2 | 1.00 (Ref) | 1.09 (0.72,1.64) | 1.47 (0.97,2.23) | **1.96 (1.24,3.09)**** | **1.84 (1.04,3.25)*** | **1.24 (1.11,1.37)***** |
|  | Model 3 | 1.00 (Ref) | 1.19 (0.78,1.82) | **1.66 (1.08,2.54)*** | **2.20 (1.35,3.59)**** | **2.12 (1.13,3.95)*** | **1.28 (1.14,1.43)***** |
| Low | Model 1 | 1.00 (Ref) | 1.19 (0.62,2.32) | 1.25 (0.61,2.58) | 1.90 (0.89,4.02) | 1.32 (0.39,4.46) | 1.16 (0.95,1.41) |
|  | Model 2 | 1.00 (Ref) | 1.13 (0.60,2.14) | 1.21 (0.60,2.43) | 1.69 (0.80,3.56) | 0.87 (0.21,3.51) | 1.11 (0.91,1.35) |
|  | Model 3 | 1.00 (Ref) | 1.22 (0.62,2.41) | 1.28 (0.61,2.69) | 1.87 (0.84,4.19) | 0.00 (0.00,0.00) | 1.07 (0.86,1.32) |
| Uric acid |  |  |  |  |  |  |  |
| Normal | Model 1 | 1.00 (Ref) | **1.47 (1.01,2.13)*** | **1.53 (1.03,2.27)*** | **2.20 (1.43,3.40)***** | 1.81 (0.95,3.47) | **1.20 (1.08,1.33)**** |
|  | Model 2 | 1.00 (Ref) | 1.19 (0.82,1.72) | 1.28 (0.86,1.91) | **1.94 (1.25,3.02)**** | 1.45 (0.78,2.70) | **1.17 (1.05,1.30)**** |
|  | Model 3 | 1.00 (Ref) | 1.32 (0.91,1.92) | 1.43 (0.95,2.14) | **2.17 (1.37,3.42)**** | 1.30 (0.63,2.67) | **1.18 (1.05,1.32)**** |
| High | Model 1 | 1.00 (Ref) | 0.79 (0.30,2.09) | 1.76 (0.71,4.35) | 1.79 (0.70,4.55) | 1.70 (0.60,4.83) | **1.26 (1.06,1.50)**** |
|  | Model 2 | 1.00 (Ref) | 0.73 (0.26,2.10) | 1.54 (0.58,4.13) | 1.62 (0.58,4.47) | 1.34 (0.44,4.14) | **1.22 (1.02,1.45)*** |
|  | Model 3 | 1.00 (Ref) | 0.69 (0.23,2.05) | 1.69 (0.61,4.65) | 1.64 (0.55,4.83) | 1.38 (0.38,4.98) | **1.25 (1.01,1.55)*** |
| Aspartate aminotransferase |  |  |  |  |  |  |  |
| Normal | Model 1 | 1.00 (Ref) | 1.17 (0.80,1.69) | **1.60 (1.10,2.33)*** | **1.91 (1.26,2.91)**** | **1.70 (0.96,3.02)** | **1.21 (1.10,1.34)***** |
|  | Model 2 | 1.00 (Ref) | 0.96 (0.66,1.40) | 1.33 (0.91,1.94) | **1.66 (1.08,2.55)*** | 1.32 (0.74,2.37) | **1.17 (1.05,1.30)**** |
|  | Model 3 | 1.00 (Ref) | 1.05 (0.71,1.54) | **1.49 (1.01,2.20)*** | **1.80 (1.15,2.82)*** | 1.32 (0.69,2.52) | **1.19 (1.07,1.33)**** |
| High | Model 1 | 1.00 (Ref) | **3.14 (1.12,8.82)*** | 2.52 (0.85,7.44) | **5.09 (1.76,14.78)**** | **4.53 (1.27,16.17)*** | **1.33 (1.11,1.61)**** |
|  | Model 2 | 1.00 (Ref) | 2.56 (0.90,7.26) | 2.02 (0.70,5.84) | **4.28 (1.46,12.56)**** | **3.52 (1.04,11.94)*** | **1.29 (1.06,1.57)*** |
|  | Model 3 | 1.00 (Ref) | 2.70 (0.97,7.55) | 2.25 (0.76,6.62) | **5.26 (1.78,15.51)**** | 4.34 (0.98,19.15) | **1.40 (1.10,1.79)**** |
| Alanine aminotransferase |  |  |  |  |  |  |  |
| Normal | Model 1 | 1.00 (Ref) | 1.32 (0.93,1.88) | **1.73 (1.21,2.48)**** | **2.28 (1.54,3.37)***** | **1.86 (1.08,3.21)*** | **1.24 (1.13,1.36)***** |
|  | Model 2 | 1.00 (Ref) | 1.10 (0.77,1.56) | **1.44 (1.01,2.07)*** | **1.94 (1.30,2.91)**** | 1.49 (0.86,2.58) | **1.20 (1.09,1.32)***** |
|  | Model 3 | 1.00 (Ref) | 1.18 (0.82,1.69) | **1.58 (1.09,2.30)*** | **2.14 (1.40,3.26)***** | 1.43 (0.76,2.67) | **1.22 (1.10,1.35)***** |
| High | Model 1 | 1.00 (Ref) | 3.05 (0.38,24.22) | 0.91 (0.09,9.68) | 2.78 (0.35,22.08) | 4.43 (0.49,39.74) | 1.23 (0.81,1.87) |
|  | Model 2 | 1.00 (Ref) | 2.78 (0.26,29.33) | 0.80 (0.06,10.23) | 2.65 (0.27,25.61) | 3.23 (0.30,35.19) | 1.15 (0.78,1.70) |
|  | Model 3 | 1.00 (Ref) | 6.18 (0.44,87.63) | 1.38 (0.11,17.36) | 3.10 (0.19,50.79) | 4.52 (0.16,129.10) | 1.08 (0.65,1.78) |

**Notes:** *, *P*<0.05; **, *P*<0.01; ***, *P*<0.001. Model 1 was unadjusted model; Model 2 adjusted for sex, age, ethnic, marital status, educational attainment, occupation,annual income, cerebrovascular disease, rheumatism, osteoarthropathy, smoking status, and alcohol consumption; Model 3 further adjusted hand grip strength, anemia, total cholesterol, low density lipoprotein cholesterol, aspartate aminotransferase, alanine aminotransferasee, serum creatinine, and uric acid.

**Abbreviations:** MetS, metabolic syndrome; ADL, activities of daily living; IADL, instrumental activities of daily living; PR, prevalence ratio; CI, confidence interval

**Supplementary Table S11.** Subgroup analysis of associations of individual MetS components with ADL disability

| Variables | Models | ADL disability [PR (95% CI)] | | | | |
| --- | --- | --- | --- | --- | --- | --- |
|  |  | Elevated blood pressure^a^ | Elevated fasting glucose^b^ | Elevated triglycerides^c^ | Reduced HDL cholesterol^d^ | Abdominal obesity^e^ |
| Gender |  |  |  |  |  |  |
| Male | Model 1 | 1.02 (1.00,1.05) | 1.03 (1.00,1.06) | 0.97 (0.94,1.01) | 1.04 (0.97,1.11) | **1.04 (1.00,1.07)*** |
|  | Model 2 | 1.01 (0.99,1.04) | **1.03 (1.00,1.07)*** | 1.00 (0.97,1.03) | 1.04 (0.98,1.11) | **1.04 (1.01,1.08)**** |
|  | Model 3 | 1.01 (0.98,1.04) | 1.03 (1.00,1.06) | 0.99 (0.96,1.03) | 1.04 (0.98,1.11) | **1.05 (1.01,1.09)**** |
| Female | Model 1 | 1.02 (1.00,1.04) | 1.01 (0.98,1.04) | 1.01 (0.99,1.04) | **1.03 (1.00,1.06)*** | 1.02 (1.00,1.04) |
|  | Model 2 | 1.00 (0.98,1.02) | 0.99 (0.96,1.02) | 1.01 (0.99,1.04) | 1.02 (1.00,1.05) | **1.02 (1.00,1.05)*** |
|  | Model 3 | 1.01 (0.99,1.03) | 1.00 (0.97,1.03) | 1.01 (0.98,1.05) | 1.02 (0.99,1.05) | **1.03 (1.00,1.05)*** |
| Age group |  |  |  |  |  |  |
| 60–69 years | Model 1 | **1.02 (1.00,1.03)*** | 1.00 (0.98,1.02) | 1.00 (0.98,1.02) | 0.99 (0.97,1.01) | 1.02 (1.00,1.04) |
|  | Model 2 | **1.02 (1.01,1.04)**** | 1.00 (0.98,1.02) | 1.00 (0.98,1.03) | 0.98 (0.96,1.01) | 1.01 (0.99,1.03) |
|  | Model 3 | **1.02 (1.01,1.04)*** | 1.00 (0.98,1.03) | 1.01 (0.98,1.03) | 0.98 (0.96,1.01) | 1.01 (0.99,1.03) |
| ≥ 70 years | Model 1 | 1.00 (0.98,1.03) | 1.04 (1.00,1.07) | 1.01 (0.97,1.05) | **1.07 (1.02,1.11)**** | **1.03 (1.00,1.06)*** |
|  | Model 2 | 1.01 (0.98,1.04) | 1.03 (0.99,1.07) | 1.01 (0.97,1.04) | **1.08 (1.03,1.13)**** | **1.04 (1.01,1.08)**** |
|  | Model 3 | 1.01 (0.98,1.04) | 1.03 (0.99,1.07) | 1.01 (0.96,1.05) | **1.07 (1.02,1.12)**** | **1.05 (1.02,1.09)**** |
| Ethnic |  |  |  |  |  |  |
| Non–zhuang | Model 1 | 1.00 (0.96,1.05) | 1.04 (0.98,1.11) | 1.03 (0.97,1.09) | 1.06 (0.99,1.14) | 0.98 (0.93,1.03) |
|  | Model 2 | 0.98 (0.94,1.03) | 1.04 (0.98,1.10) | 1.05 (0.99,1.11) | **1.08 (1.00,1.16)*** | 0.99 (0.94,1.03) |
|  | Model 3 | 1.00 (0.95,1.05) | 1.04 (0.98,1.11) | 1.04 (0.96,1.12) | 1.07 (0.99,1.17) | 0.99 (0.94,1.04) |
| Zhuang | Model 1 | **1.02 (1.01,1.04)**** | 1.02 (0.99,1.04) | 0.99 (0.97,1.01) | 1.02 (1.00,1.05) | **1.03 (1.01,1.05)**** |
|  | Model 2 | 1.01 (0.99,1.03) | 1.01 (0.98,1.03) | 1.00 (0.98,1.02) | 1.02 (0.99,1.04) | **1.04 (1.02,1.06)***** |
|  | Model 3 | 1.01 (0.99,1.03) | 1.00 (0.98,1.03) | 1.00 (0.97,1.02) | 1.01 (0.99,1.04) | **1.04 (1.02,1.06)***** |
| Marital status |  |  |  |  |  |  |
| Partnered | Model 1 | **1.03 (1.01,1.05)**** | 1.02 (0.99,1.04) | 0.99 (0.97,1.02) | 1.01 (0.98,1.04) | 1.02 (1.00,1.04) |
|  | Model 2 | **1.02 (1.00,1.04)*** | 1.01 (0.99,1.04) | 1.00 (0.98,1.03) | 1.01 (0.98,1.04) | **1.02 (1.00,1.05)*** |
|  | Model 3 | **1.02 (1.00,1.04)*** | 1.01 (0.99,1.04) | 1.01 (0.98,1.04) | 1.01 (0.98,1.04) | **1.02 (1.00,1.05)*** |
| Single | Model 1 | 1.00 (0.97,1.04) | 1.03 (0.98,1.07) | 1.01 (0.97,1.05) | **1.05 (1.01,1.10)*** | **1.03 (1.00,1.07)*** |
|  | Model 2 | 0.99 (0.95,1.02) | 1.00 (0.96,1.04) | 1.02 (0.98,1.06) | **1.04 (1.00,1.09)*** | **1.04 (1.00,1.07)*** |
|  | Model 3 | 1.00 (0.96,1.03) | 1.00 (0.96,1.05) | 1.00 (0.95,1.05) | **1.06 (1.00,1.11)*** | **1.04 (1.01,1.08)*** |
| Educational attainment |  |  |  |  |  |  |
| Less than primary school | Model 1 | **1.03 (1.00,1.05)*** | 1.01 (0.98,1.05) | 1.01 (0.98,1.04) | **1.05 (1.01,1.09)*** | 1.02 (1.00,1.05) |
|  | Model 2 | 1.01 (0.98,1.03) | 0.99 (0.95,1.02) | 1.01 (0.98,1.05) | **1.04 (1.00,1.07)*** | **1.03 (1.00,1.06)*** |
|  | Model 3 | 1.01 (0.99,1.04) | 0.99 (0.95,1.02) | 1.02 (0.98,1.06) | 1.03 (0.99,1.07) | **1.03 (1.01,1.06)*** |
| Primary school | Model 1 | 1.02 (0.99,1.05) | 1.03 (0.99,1.07) | 1.00 (0.96,1.04) | 1.01 (0.96,1.05) | 1.02 (0.99,1.06) |
|  | Model 2 | 1.01 (0.98,1.04) | 1.04 (1.00,1.08) | 1.00 (0.97,1.04) | 1.01 (0.97,1.06) | 1.03 (0.99,1.06) |
|  | Model 3 | 1.01 (0.98,1.04) | 1.04 (1.00,1.08) | 1.00 (0.95,1.04) | 1.02 (0.97,1.07) | 1.03 (0.99,1.06) |
| High school and above | Model 1 | 1.01 (0.98,1.04) | 1.03 (0.98,1.07) | 0.98 (0.95,1.01) | 0.98 (0.93,1.04) | 1.03 (0.99,1.07) |
|  | Model 2 | 1.00 (0.97,1.04) | 1.02 (0.98,1.06) | 0.99 (0.96,1.02) | 0.99 (0.94,1.05) | 1.03 (1.00,1.07) |
|  | Model 3 | 1.00 (0.97,1.03) | 1.01 (0.97,1.06) | 0.98 (0.95,1.02) | 0.99 (0.93,1.05) | 1.04 (1.00,1.08) |
| Occupation |  |  |  |  |  |  |
| Non–farmer | Model 1 | 0.97 (0.91,1.02) | 1.04 (0.97,1.11) | 0.99 (0.93,1.04) | 1.00 (0.94,1.07) | 1.05 (1.00,1.10) |
|  | Model 2 | 0.96 (0.92,1.01) | 1.04 (0.98,1.11) | 1.00 (0.95,1.06) | 1.01 (0.95,1.08) | **1.05 (1.00,1.10)*** |
|  | Model 3 | 0.96 (0.92,1.01) | 1.05 (0.98,1.12) | 1.01 (0.94,1.08) | 1.01 (0.93,1.09) | **1.06 (1.00,1.11)*** |
| Farmer | Model 1 | **1.03 (1.01,1.05)**** | 1.02 (1.00,1.04) | 1.00 (0.98,1.02) | **1.03 (1.01,1.06)*** | **1.02 (1.00,1.04)*** |
|  | Model 2 | 1.01 (0.99,1.03) | 1.01 (0.98,1.03) | 1.01 (0.99,1.03) | 1.03 (1.00,1.05) | **1.03 (1.01,1.05)**** |
|  | Model 3 | 1.01 (1.00,1.03) | 1.01 (0.98,1.03) | 1.00 (0.98,1.03) | 1.02 (1.00,1.05) | **1.03 (1.01,1.05)**** |
| Annual income |  |  |  |  |  |  |
| ＜10000 renminbi | Model 1 | 1.03 (1.00,1.06) | 1.03 (0.99,1.07) | 1.01 (0.97,1.05) | **1.06 (1.01,1.11)*** | 1.02 (0.99,1.06) |
|  | Model 2 | 1.01 (0.98,1.04) | 1.02 (0.98,1.06) | 1.02 (0.98,1.06) | **1.05 (1.00,1.09)*** | 1.02 (0.99,1.05) |
|  | Model 3 | 1.01 (0.99,1.04) | 1.01 (0.98,1.05) | 1.02 (0.97,1.06) | **1.05 (1.00,1.10)*** | 1.02 (0.98,1.05) |
| 10000–29999 renminbi | Model 1 | 1.03 (1.00,1.06) | 1.01 (0.97,1.06) | 0.99 (0.95,1.03) | 1.00 (0.96,1.05) | **1.04 (1.01,1.07)*** |
|  | Model 2 | 1.02 (0.99,1.04) | 1.01 (0.97,1.05) | 0.99 (0.96,1.03) | 1.01 (0.96,1.05) | **1.03 (1.00,1.07)*** |
|  | Model 3 | 1.02 (0.99,1.05) | 1.01 (0.97,1.06) | 0.99 (0.95,1.03) | 1.00 (0.96,1.05) | **1.04 (1.01,1.08)*** |
| ≥ 30000 renminbi | Model 1 | 1.01 (0.99,1.04) | 1.01 (0.98,1.05) | 0.99 (0.96,1.02) | 1.01 (0.97,1.05) | **1.03 (1.00,1.06)*** |
|  | Model 2 | 1.00 (0.97,1.02) | 1.00 (0.97,1.03) | 1.00 (0.97,1.03) | 1.01 (0.97,1.05) | **1.04 (1.01,1.06)*** |
|  | Model 3 | 1.00 (0.97,1.03) | 1.00 (0.96,1.03) | 1.00 (0.96,1.04) | 1.00 (0.96,1.05) | **1.05 (1.02,1.08)**** |
| Cerebrovascular disease |  |  |  |  |  |  |
| No | Model 1 | **1.02 (1.01,1.04)*** | 1.02 (1.00,1.05) | 0.99 (0.97,1.01) | **1.03 (1.00,1.05)*** | **1.02 (1.01,1.04)**** |
|  | Model 2 | 1.01 (0.99,1.02) | 1.01 (0.99,1.03) | 1.00 (0.98,1.02) | 1.02 (1.00,1.05) | **1.03 (1.01,1.05)**** |
|  | Model 3 | 1.01 (0.99,1.03) | 1.01 (0.99,1.03) | 1.00 (0.97,1.02) | 1.02 (0.99,1.05) | **1.03 (1.01,1.05)**** |
| Yes | Model 1 | 1.03 (0.96,1.11) | 0.97 (0.87,1.09) | 1.11 (0.99,1.25) | 1.11 (0.96,1.29) | 1.01 (0.93,1.10) |
|  | Model 2 | 1.03 (0.96,1.11) | 0.98 (0.87,1.10) | **1.12 (1.00,1.25)*** | 1.07 (0.94,1.21) | 0.99 (0.91,1.08) |
|  | Model 3 | 1.04 (0.96,1.12) | 0.97 (0.87,1.09) | **1.19 (1.04,1.35)**** | 1.04 (0.91,1.19) | 1.01 (0.91,1.11) |
| Rheumatism |  |  |  |  |  |  |
| No | Model 1 | **1.02 (1.00,1.04)*** | 1.02 (1.00,1.05) | 1.00 (0.98,1.02) | 1.02 (0.99,1.05) | **1.02 (1.00,1.04)*** |
|  | Model 2 | 1.01 (0.99,1.02) | 1.01 (0.99,1.03) | 1.01 (0.99,1.03) | 1.02 (0.99,1.04) | **1.03 (1.01,1.05)**** |
|  | Model 3 | 1.01 (0.99,1.03) | 1.01 (0.99,1.03) | 1.01 (0.98,1.03) | 1.01 (0.98,1.04) | **1.04 (1.01,1.06)**** |
| Yes | Model 1 | 1.03 (0.98,1.08) | 1.02 (0.94,1.10) | 0.98 (0.92,1.04) | **1.14 (1.04,1.26)**** | 1.02 (0.96,1.08) |
|  | Model 2 | 1.02 (0.97,1.06) | 1.01 (0.94,1.09) | 0.98 (0.92,1.04) | **1.13 (1.03,1.23)**** | 1.00 (0.94,1.06) |
|  | Model 3 | 1.02 (0.98,1.07) | 1.02 (0.94,1.10) | 0.97 (0.89,1.05) | **1.14 (1.03,1.26)**** | 1.00 (0.94,1.07) |
| Osteoarthropathy |  |  |  |  |  |  |
| No | Model 1 | **1.02 (1.00,1.04)*** | 1.01 (0.99,1.04) | 1.01 (0.98,1.03) | 1.02 (1.00,1.05) | **1.02 (1.00,1.04)*** |
|  | Model 2 | 1.00 (0.98,1.02) | 1.01 (0.98,1.03) | 1.01 (0.99,1.04) | 1.02 (0.99,1.04) | **1.03 (1.01,1.05)**** |
|  | Model 3 | 1.01 (0.99,1.02) | 1.00 (0.98,1.03) | 1.01 (0.98,1.04) | 1.02 (0.99,1.05) | **1.03 (1.01,1.05)**** |
| Yes | Model 1 | **1.04 (1.00,1.08)*** | 1.08 (1.00,1.18) | **0.94 (0.90,0.98)**** | 1.09 (1.00,1.19) | 1.02 (0.97,1.07) |
|  | Model 2 | 1.03 (0.99,1.07) | 1.07 (0.99,1.15) | **0.95 (0.91,1.00)*** | **1.09 (1.00,1.18)*** | 1.02 (0.97,1.08) |
|  | Model 3 | 1.03 (0.99,1.08) | 1.08 (0.99,1.16) | 0.97 (0.91,1.02) | 1.08 (0.99,1.18) | 1.04 (0.98,1.09) |
| Smoking status |  |  |  |  |  |  |
| No | Model 1 | **1.02 (1.00,1.04)*** | 1.02 (0.99,1.04) | 1.01 (0.98,1.03) | **1.03 (1.00,1.06)*** | **1.02 (1.00,1.04)*** |
|  | Model 2 | 1.00 (0.98,1.02) | 1.01 (0.98,1.03) | 1.01 (0.99,1.03) | 1.03 (1.00,1.05) | **1.03 (1.01,1.05)**** |
|  | Model 3 | 1.00 (0.99,1.02) | 1.01 (0.98,1.03) | 1.01 (0.98,1.04) | 1.03 (1.00,1.06) | **1.03 (1.01,1.05)**** |
| Yes | Model 1 | **1.04 (1.01,1.08)*** | 1.04 (0.99,1.09) | **0.96 (0.92,1.00)*** | 1.01 (0.92,1.12) | 1.03 (0.98,1.09) |
|  | Model 2 | **1.04 (1.00,1.07)*** | 1.04 (0.99,1.09) | 0.98 (0.95,1.03) | 1.00 (0.92,1.09) | 1.03 (0.98,1.08) |
|  | Model 3 | 1.04 (1.00,1.07) | 1.04 (0.99,1.09) | 0.99 (0.94,1.04) | 1.01 (0.93,1.10) | 1.04 (0.98,1.10) |
| Alcohol consumption |  |  |  |  |  |  |
| No | Model 1 | **1.03 (1.01,1.05)**** | 1.02 (0.99,1.05) | 1.01 (0.98,1.03) | 1.02 (1.00,1.05) | **1.02 (1.00,1.04)*** |
|  | Model 2 | 1.01 (0.99,1.02) | 1.00 (0.98,1.03) | 1.01 (0.99,1.04) | 1.02 (1.00,1.05) | **1.03 (1.01,1.05)**** |
|  | Model 3 | 1.01 (0.99,1.03) | 1.00 (0.98,1.03) | 1.01 (0.98,1.04) | 1.03 (1.00,1.06) | **1.03 (1.01,1.06)**** |
| Yes | Model 1 | 1.01 (0.98,1.04) | 1.03 (0.99,1.07) | 0.98 (0.95,1.02) | 1.04 (0.96,1.13) | 1.02 (0.98,1.06) |
|  | Model 2 | 1.01 (0.98,1.04) | 1.03 (1.00,1.07) | 0.99 (0.96,1.03) | 1.05 (0.97,1.13) | 1.02 (0.99,1.06) |
|  | Model 3 | 1.01 (0.98,1.04) | 1.03 (0.99,1.07) | 0.99 (0.95,1.03) | 1.03 (0.95,1.12) | 1.03 (0.99,1.07) |
| Low grip strength |  |  |  |  |  |  |
| No | Model 1 | 1.01 (0.99,1.03) | 1.01 (0.98,1.04) | 0.99 (0.97,1.02) | 1.01 (0.98,1.04) | 1.02 (0.99,1.04) |
|  | Model 2 | 1.01 (0.99,1.03) | 1.01 (0.98,1.04) | 1.00 (0.97,1.02) | 1.01 (0.97,1.04) | 1.01 (0.99,1.03) |
|  | Model 3 | 1.01 (0.99,1.03) | 1.01 (0.98,1.04) | 1.01 (0.98,1.04) | 1.00 (0.96,1.03) | 1.01 (0.99,1.03) |
| Yes | Model 1 | **1.03 (1.00,1.05)*** | 1.02 (0.99,1.06) | 1.01 (0.98,1.05) | 1.03 (0.99,1.07) | **1.04 (1.01,1.07)**** |
|  | Model 2 | 1.01 (0.98,1.03) | 1.01 (0.98,1.04) | 1.02 (0.99,1.05) | 1.03 (0.99,1.07) | **1.05 (1.02,1.08)***** |
|  | Model 3 | 1.01 (0.99,1.03) | 1.01 (0.98,1.04) | 1.00 (0.97,1.04) | 1.04 (1.00,1.08) | **1.05 (1.02,1.08)***** |
| Anemia |  |  |  |  |  |  |
| No | Model 1 | **1.02 (1.01,1.04)*** | 1.01 (0.99,1.04) | 1.00 (0.97,1.02) | 1.02 (0.98,1.05) | 1.02 (1.00,1.04) |
|  | Model 2 | 1.01 (0.99,1.03) | 1.00 (0.98,1.03) | 1.00 (0.98,1.03) | 1.01 (0.98,1.04) | 1.02 (1.00,1.04) |
|  | Model 3 | 1.02 (1.00,1.04) | 1.00 (0.98,1.03) | 1.01 (0.98,1.04) | 1.01 (0.98,1.05) | 1.02 (1.00,1.04) |
| Yes | Model 1 | 1.02 (1.00,1.05) | 1.04 (0.99,1.08) | 1.02 (0.97,1.07) | **1.05 (1.00,1.10)*** | **1.04 (1.01,1.08)*** |
|  | Model 2 | 1.00 (0.97,1.02) | 1.03 (0.99,1.07) | 1.02 (0.97,1.06) | **1.05 (1.00,1.09)*** | **1.05 (1.02,1.09)**** |
|  | Model 3 | 1.00 (0.97,1.03) | 1.03 (0.99,1.08) | 1.00 (0.95,1.05) | 1.05 (1.00,1.10) | **1.06 (1.02,1.10)**** |
| Low density lipoprotein cholesterol |  |  |  |  |  |  |
| Normal | Model 1 | 1.02 (1.00,1.04) | **1.05 (1.01,1.08)**** | 0.99 (0.96,1.02) | 1.03 (0.99,1.06) | **1.03 (1.01,1.06)*** |
|  | Model 2 | 1.00 (0.98,1.02) | **1.04 (1.00,1.07)*** | 1.00 (0.97,1.03) | 1.02 (0.99,1.05) | **1.04 (1.01,1.06)**** |
|  | Model 3 | 1.01 (0.98,1.03) | **1.04 (1.01,1.07)*** | 1.00 (0.97,1.04) | 1.02 (0.98,1.05) | **1.04 (1.01,1.07)**** |
| High | Model 1 | **1.03 (1.00,1.05)*** | 0.98 (0.95,1.01) | 1.01 (0.98,1.04) | 1.03 (0.99,1.08) | 1.02 (0.99,1.04) |
|  | Model 2 | 1.02 (0.99,1.04) | 0.97 (0.94,1.00) | 1.01 (0.98,1.04) | 1.03 (0.98,1.08) | 1.02 (1.00,1.05) |
|  | Model 3 | 1.02 (0.99,1.04) | 0.98 (0.94,1.01) | 1.01 (0.98,1.05) | 1.03 (0.98,1.09) | 1.02 (1.00,1.05) |
| Total cholesterol |  |  |  |  |  |  |
| Normal | Model 1 | 1.02 (0.99,1.04) | 1.04 (1.00,1.07) | 0.99 (0.95,1.03) | 1.03 (0.99,1.06) | **1.03 (1.00,1.06)*** |
|  | Model 2 | 1.00 (0.97,1.02) | 1.02 (0.99,1.06) | 1.00 (0.96,1.03) | 1.02 (0.99,1.06) | **1.04 (1.01,1.07)**** |
|  | Model 3 | 1.00 (0.98,1.03) | 1.03 (0.99,1.06) | 0.99 (0.95,1.03) | 1.02 (0.99,1.05) | **1.05 (1.02,1.08)**** |
| High | Model 1 | **1.03 (1.00,1.05)*** | 1.01 (0.98,1.04) | 1.01 (0.98,1.03) | 1.03 (0.98,1.08) | 1.02 (1.00,1.04) |
|  | Model 2 | 1.01 (0.99,1.04) | 1.00 (0.97,1.03) | 1.01 (0.99,1.04) | 1.02 (0.98,1.07) | **1.02 (1.00,1.05)*** |
|  | Model 3 | 1.01 (0.99,1.04) | 1.00 (0.97,1.02) | 1.01 (0.98,1.04) | 1.03 (0.98,1.08) | **1.03 (1.00,1.05)*** |
| Serum creatinine |  |  |  |  |  |  |
| Normal | Model 1 | 1.02 (1.00,1.04) | 1.01 (0.98,1.03) | 1.00 (0.98,1.03) | **1.04 (1.01,1.08)**** | **1.02 (1.00,1.04)*** |
|  | Model 2 | 1.00 (0.98,1.02) | 1.00 (0.98,1.02) | 1.01 (0.99,1.04) | **1.04 (1.01,1.07)**** | **1.03 (1.01,1.05)**** |
|  | Model 3 | 1.01 (0.99,1.02) | 1.00 (0.98,1.03) | 1.02 (0.99,1.04) | 1.03 (1.00,1.06) | **1.03 (1.01,1.06)**** |
| Low | Model 1 | 1.02 (0.99,1.06) | **1.06 (1.01,1.12)*** | 0.98 (0.94,1.03) | 0.98 (0.93,1.04) | 1.02 (0.98,1.06) |
|  | Model 2 | 1.02 (0.98,1.06) | 1.05 (1.00,1.10) | 0.98 (0.94,1.03) | 0.99 (0.93,1.04) | 1.02 (0.98,1.06) |
|  | Model 3 | 1.02 (0.98,1.06) | 1.03 (0.98,1.09) | 0.97 (0.92,1.03) | 1.00 (0.94,1.06) | 1.03 (0.98,1.07) |
| Uric acid |  |  |  |  |  |  |
| Normal | Model 1 | **1.03 (1.01,1.04)**** | 1.01 (0.99,1.04) | 0.99 (0.97,1.02) | 1.03 (1.00,1.05) | 1.02 (1.00,1.04) |
|  | Model 2 | 1.01 (0.99,1.03) | 1.01 (0.98,1.03) | 1.00 (0.98,1.02) | 1.02 (1.00,1.05) | **1.02 (1.00,1.04)*** |
|  | Model 3 | 1.01 (0.99,1.03) | 1.00 (0.98,1.03) | 0.99 (0.97,1.02) | 1.02 (0.99,1.05) | **1.03 (1.01,1.05)*** |
| High | Model 1 | 0.99 (0.94,1.04) | 1.04 (0.99,1.10) | 1.00 (0.96,1.04) | 1.07 (0.99,1.16) | 1.04 (1.00,1.09) |
|  | Model 2 | 0.99 (0.94,1.03) | 1.02 (0.97,1.07) | 1.02 (0.98,1.06) | 1.05 (0.98,1.13) | **1.05 (1.01,1.09)*** |
|  | Model 3 | 0.99 (0.95,1.04) | 1.03 (0.98,1.09) | 1.02 (0.97,1.08) | 1.03 (0.96,1.12) | **1.05 (1.01,1.10)*** |
| Aspartate aminotransferase |  |  |  |  |  |  |
| Normal | Model 1 | 1.01 (1.00,1.03) | 1.02 (0.99,1.05) | 1.01 (0.98,1.03) | 1.02 (0.99,1.05) | 1.02 (1.00,1.04) |
|  | Model 2 | 1.00 (0.98,1.02) | 1.01 (0.99,1.03) | 1.01 (0.99,1.04) | 1.02 (0.99,1.05) | **1.03 (1.00,1.05)*** |
|  | Model 3 | 1.00 (0.98,1.02) | 1.01 (0.98,1.03) | 1.01 (0.98,1.04) | 1.02 (0.99,1.05) | **1.03 (1.01,1.05)**** |
| High | Model 1 | **1.05 (1.02,1.08)**** | 1.02 (0.98,1.07) | 0.98 (0.94,1.02) | **1.07 (1.00,1.14)*** | 1.03 (0.99,1.08) |
|  | Model 2 | **1.04 (1.01,1.07)*** | 1.02 (0.97,1.06) | 0.98 (0.94,1.02) | 1.06 (0.99,1.13) | **1.04 (1.00,1.08)*** |
|  | Model 3 | **1.04 (1.01,1.08)*** | 1.02 (0.97,1.07) | 0.99 (0.94,1.04) | 1.04 (0.98,1.12) | 1.04 (1.00,1.09) |
| Alanine aminotransferase |  |  |  |  |  |  |
| Normal | Model 1 | **1.02 (1.00,1.04)*** | **1.03 (1.00,1.05)*** | 1.00 (0.98,1.02) | **1.03 (1.00,1.06)*** | **1.02 (1.00,1.04)*** |
|  | Model 2 | 1.00 (0.99,1.02) | 1.02 (0.99,1.04) | 1.01 (0.99,1.03) | 1.02 (1.00,1.05) | **1.02 (1.01,1.04)*** |
|  | Model 3 | 1.01 (0.99,1.02) | 1.02 (0.99,1.04) | 1.01 (0.98,1.03) | 1.03 (1.00,1.05) | **1.03 (1.01,1.05)**** |
| High | Model 1 | 1.03 (0.97,1.11) | 0.96 (0.90,1.02) | 0.97 (0.91,1.03) | 1.04 (0.94,1.16) | **1.09 (1.02,1.16)**** |
|  | Model 2 | 1.03 (0.96,1.10) | 0.96 (0.90,1.02) | 0.96 (0.90,1.02) | 1.04 (0.94,1.15) | **1.10 (1.03,1.17)**** |
|  | Model 3 | 1.06 (0.98,1.13) | 0.96 (0.90,1.03) | 0.98 (0.90,1.07) | 0.99 (0.89,1.10) | **1.07 (1.00,1.14)*** |

**Notes:** ^a^, the reference group was non–elevated blood pressure; ^b^, the reference group was non–elevated fasting glucose; ^c^, the reference group was non–elevated triglycerides; ^d^, the reference group was non–reduced HDL cholesterol; ^e^, the reference group was non–abdominal obesity. *, *P*<0.05; **, *P*<0.01; ***, *P*<0.001. Model 1 was unadjusted model; Model 2 adjusted for sex, age, ethnic, marital status, educational attainment, occupation, annual income, cerebrovascular disease, rheumatism, osteoarthropathy, smoking status, and alcohol consumption; Model 3 further adjusted hand grip strength, anemia, total cholesterol, low density lipoprotein cholesterol, aspartate aminotransferase, alanine aminotransferasee, serum creatinine, and uric acid.

**Abbreviations:** MetS, metabolic syndrome; ADL, activities of daily living; PR, prevalence ratio; CI, confidence interval; HDL, high density lipoprotein.

**Supplementary Table S12.** Subgroup analysis of associations between individual MetS components and IADL disability

| Variables | Models | IADL disability [PR (95% CI)] | | | | |
| --- | --- | --- | --- | --- | --- | --- |
|  |  | Elevated blood pressure ^a^ | Elevated fasting glucose ^b^ | Elevated triglycerides ^c^ | Reduced HDL cholesterol ^d^ | Abdominal obesity ^e^ |
| Gender |  |  |  |  |  |  |
| Male | Model 1 | **1.05 (1.02,1.09)**** | 1.01 (0.97,1.05) | 0.97 (0.93,1.01) | 1.02 (0.94,1.10) | 0.99 (0.95,1.03) |
|  | Model 2 | 1.03 (1.00,1.06) | 1.01 (0.97,1.05) | 1.00 (0.97,1.04) | 1.02 (0.95,1.10) | 1.01 (0.97,1.04) |
|  | Model 3 | 1.03 (1.00,1.06) | 1.00 (0.96,1.04) | 0.99 (0.95,1.04) | 1.02 (0.95,1.10) | 1.01 (0.97,1.05) |
| Female | Model 1 | **1.04 (1.01,1.07)*** | **1.04 (1.00,1.09)*** | 0.97 (0.94,1.01) | 0.97 (0.94,1.01) | 1.00 (0.98,1.03) |
|  | Model 2 | 1.01 (0.98,1.04) | 1.01 (0.97,1.05) | 0.98 (0.95,1.02) | **0.96 (0.93,0.99)**** | 1.01 (0.98,1.04) |
|  | Model 3 | 1.01 (0.99,1.04) | 1.01 (0.98,1.05) | 0.98 (0.93,1.02) | **0.96 (0.92,0.99)*** | 1.01 (0.98,1.04) |
| Age group |  |  |  |  |  |  |
| 60–69 years | Model 1 | 0.99 (0.97,1.02) | 0.99 (0.95,1.03) | 0.98 (0.94,1.01) | 0.98 (0.94,1.02) | 1.00 (0.97,1.03) |
|  | Model 2 | 1.01 (0.98,1.04) | 1.02 (0.98,1.06) | 1.00 (0.97,1.04) | **0.94 (0.90,0.98)**** | 0.98 (0.95,1.02) |
|  | Model 3 | 1.02 (0.99,1.05) | 1.02 (0.98,1.06) | 0.99 (0.95,1.03) | **0.94 (0.89,0.98)**** | 0.98 (0.95,1.02) |
| ≥ 70 years | Model 1 | 1.02 (0.99,1.06) | 1.02 (0.98,1.06) | 0.97 (0.93,1.02) | **1.05 (1.01,1.10)*** | **1.05 (1.02,1.09)**** |
|  | Model 2 | 1.03 (1.00,1.07) | 1.01 (0.97,1.05) | 0.98 (0.94,1.02) | 1.00 (0.96,1.05) | 1.03 (1.00,1.07) |
|  | Model 3 | 1.02 (0.99,1.06) | 1.01 (0.97,1.05) | 0.98 (0.93,1.03) | 0.99 (0.95,1.04) | 1.03 (1.00,1.07) |
| Ethnic |  |  |  |  |  |  |
| Non–zhuang | Model 1 | 0.97 (0.92,1.03) | 1.02 (0.95,1.09) | 1.00 (0.94,1.07) | 1.06 (0.98,1.14) | 0.98 (0.93,1.04) |
|  | Model 2 | 0.96 (0.91,1.01) | 1.04 (0.97,1.11) | 1.03 (0.97,1.10) | 1.03 (0.95,1.11) | 0.97 (0.92,1.02) |
|  | Model 3 | 0.96 (0.91,1.02) | 1.04 (0.98,1.12) | 1.01 (0.94,1.09) | 1.05 (0.96,1.14) | 0.97 (0.91,1.03) |
| Zhuang | Model 1 | **1.04 (1.01,1.06)**** | 1.01 (0.98,1.04) | **0.95 (0.92,0.98)**** | 1.02 (0.98,1.05) | **1.03 (1.01,1.06)*** |
|  | Model 2 | 1.02 (1.00,1.05) | 1.00 (0.97,1.03) | 0.98 (0.95,1.01) | **0.95 (0.92,0.98)**** | 1.02 (0.99,1.04) |
|  | Model 3 | 1.02 (1.00,1.05) | 1.00 (0.97,1.03) | 0.97 (0.94,1.01) | **0.95 (0.92,0.98)**** | 1.02 (0.99,1.04) |
| Marital status |  |  |  |  |  |  |
| Partnered | Model 1 | 1.02 (0.99,1.04) | 1.00 (0.97,1.04) | 0.97 (0.94,1.00) | 1.01 (0.97,1.05) | 1.02 (0.99,1.04) |
|  | Model 2 | 1.02 (0.99,1.04) | 1.02 (0.99,1.05) | 1.00 (0.97,1.03) | **0.96 (0.92,1.00)*** | 0.99 (0.97,1.02) |
|  | Model 3 | 1.02 (0.99,1.04) | 1.02 (0.99,1.05) | 1.00 (0.96,1.04) | 0.96 (0.92,1.00) | 0.99 (0.96,1.02) |
| Single | Model 1 | 1.03 (0.99,1.08) | 1.03 (0.98,1.08) | 0.96 (0.91,1.01) | 1.01 (0.96,1.06) | 1.04 (1.00,1.08) |
|  | Model 2 | 1.01 (0.97,1.05) | 0.99 (0.94,1.04) | 0.98 (0.93,1.02) | 0.97 (0.93,1.02) | **1.04 (1.00,1.08)*** |
|  | Model 3 | 1.01 (0.97,1.05) | 0.99 (0.94,1.04) | **0.94 (0.89,1.00)*** | 0.99 (0.94,1.05) | **1.05 (1.01,1.09)*** |
| Educational attainment |  |  |  |  |  |  |
| Less than primary school | Model 1 | 1.03 (0.99,1.06) | 1.03 (0.99,1.08) | 0.99 (0.95,1.03) | 0.99 (0.95,1.03) | 1.01 (0.97,1.04) |
|  | Model 2 | 1.00 (0.97,1.03) | 0.99 (0.95,1.03) | 1.00 (0.96,1.04) | **0.96 (0.92,1.00)*** | 1.01 (0.97,1.04) |
|  | Model 3 | 1.01 (0.97,1.04) | 0.99 (0.95,1.03) | 1.00 (0.95,1.05) | **0.95 (0.91,1.00)*** | 1.00 (0.97,1.04) |
| Primary school | Model 1 | 1.03 (0.99,1.07) | 1.01 (0.97,1.06) | 0.98 (0.93,1.03) | 1.00 (0.95,1.06) | 1.01 (0.97,1.05) |
|  | Model 2 | 1.03 (0.99,1.07) | 1.04 (0.99,1.09) | 0.99 (0.94,1.03) | 0.97 (0.91,1.02) | 1.00 (0.96,1.04) |
|  | Model 3 | 1.03 (0.99,1.07) | 1.04 (0.99,1.09) | 0.97 (0.91,1.02) | 0.98 (0.92,1.04) | 1.00 (0.96,1.04) |
| High school and above | Model 1 | **1.05 (1.01,1.09)*** | 1.02 (0.97,1.08) | 0.96 (0.92,1.00) | 0.98 (0.91,1.05) | 1.02 (0.98,1.07) |
|  | Model 2 | 1.03 (0.99,1.07) | 1.02 (0.97,1.07) | 0.97 (0.92,1.01) | 0.98 (0.91,1.06) | 1.04 (0.99,1.09) |
|  | Model 3 | 1.03 (0.99,1.07) | 1.02 (0.96,1.07) | 0.95 (0.90,1.01) | 0.98 (0.90,1.06) | 1.04 (0.99,1.09) |
| Occupation |  |  |  |  |  |  |
| Non–farmer | Model 1 | 1.01 (0.95,1.08) | 1.02 (0.95,1.11) | 0.99 (0.93,1.07) | 0.97 (0.89,1.05) | 1.01 (0.94,1.07) |
|  | Model 2 | 1.00 (0.94,1.06) | 1.03 (0.96,1.11) | 1.02 (0.95,1.09) | 0.98 (0.91,1.06) | 1.02 (0.96,1.08) |
|  | Model 3 | 1.00 (0.95,1.07) | 1.04 (0.97,1.12) | 1.04 (0.96,1.13) | 0.96 (0.88,1.05) | 1.02 (0.96,1.08) |
| Farmer | Model 1 | **1.03 (1.01,1.06)*** | 1.01 (0.98,1.04) | **0.97 (0.94,0.99)*** | 1.03 (0.99,1.06) | **1.03 (1.01,1.06)**** |
|  | Model 2 | 1.02 (0.99,1.04) | 1.01 (0.98,1.04) | 0.99 (0.96,1.01) | **0.96 (0.93,0.99)*** | 1.01 (0.98,1.03) |
|  | Model 3 | 1.02 (0.99,1.04) | 1.00 (0.98,1.03) | 0.98 (0.94,1.01) | 0.96 (0.93,1.00) | 1.01 (0.98,1.03) |
| Annual income |  |  |  |  |  |  |
| ＜10000 renminbi | Model 1 | 1.01 (0.98,1.05) | 1.03 (0.98,1.07) | 0.96 (0.92,1.01) | **1.06 (1.01,1.12)*** | **1.05 (1.01,1.09)*** |
|  | Model 2 | 1.02 (0.99,1.06) | 1.02 (0.98,1.06) | 1.00 (0.96,1.04) | 1.00 (0.95,1.04) | 1.01 (0.97,1.04) |
|  | Model 3 | 1.02 (0.99,1.06) | 1.01 (0.97,1.06) | 0.99 (0.94,1.04) | 1.00 (0.95,1.06) | 0.99 (0.95,1.03) |
| 10000–29999 renminbi | Model 1 | 1.04 (1.00,1.09) | 1.00 (0.95,1.06) | 0.95 (0.90,1.01) | 0.97 (0.91,1.04) | 1.02 (0.98,1.07) |
|  | Model 2 | 1.01 (0.97,1.06) | 1.02 (0.97,1.08) | 0.99 (0.94,1.05) | **0.92 (0.87,0.97)**** | 0.99 (0.95,1.04) |
|  | Model 3 | 1.02 (0.98,1.07) | 1.01 (0.96,1.07) | 0.97 (0.91,1.03) | **0.93 (0.87,1.00)*** | 0.99 (0.95,1.04) |
| ≥ 30000 renminbi | Model 1 | 1.03 (0.99,1.07) | 0.99 (0.94,1.04) | 0.96 (0.91,1.00) | 0.99 (0.94,1.04) | 1.03 (0.99,1.07) |
|  | Model 2 | 1.01 (0.98,1.05) | 0.99 (0.95,1.03) | 0.98 (0.94,1.02) | 0.95 (0.91,1.00) | 1.03 (0.99,1.07) |
|  | Model 3 | 1.01 (0.97,1.05) | 1.00 (0.95,1.04) | 0.99 (0.93,1.04) | **0.94 (0.89,1.00)*** | **1.05 (1.01,1.09)*** |
| Cerebrovascular disease |  |  |  |  |  |  |
| No | Model 1 | **1.03 (1.00,1.05)*** | 1.01 (0.98,1.04) | **0.96 (0.93,0.98)**** | 1.02 (0.99,1.06) | **1.03 (1.00,1.05)*** |
|  | Model 2 | 1.01 (0.99,1.04) | 1.01 (0.98,1.04) | 0.99 (0.96,1.01) | **0.97 (0.94,1.00)*** | 1.01 (0.99,1.03) |
|  | Model 3 | 1.02 (0.99,1.04) | 1.01 (0.98,1.04) | 0.98 (0.95,1.01) | 0.97 (0.93,1.00) | 1.01 (0.98,1.03) |
| Yes | Model 1 | 1.01 (0.91,1.13) | 0.96 (0.82,1.12) | 1.10 (0.97,1.26) | 1.01 (0.86,1.18) | 1.06 (0.94,1.19) |
|  | Model 2 | 1.01 (0.92,1.11) | 0.95 (0.83,1.10) | 1.08 (0.97,1.21) | **0.85 (0.73,0.98)*** | 1.08 (0.96,1.22) |
|  | Model 3 | 1.01 (0.91,1.11) | 0.95 (0.82,1.10) | 1.11 (0.96,1.28) | **0.83 (0.71,0.98)*** | 1.10 (0.97,1.24) |
| Rheumatism |  |  |  |  |  |  |
| No | Model 1 | **1.03 (1.01,1.06)*** | 1.01 (0.98,1.04) | **0.96 (0.93,0.99)**** | 1.02 (0.98,1.05) | **1.03 (1.00,1.05)*** |
|  | Model 2 | 1.02 (1.00,1.04) | 1.01 (0.98,1.04) | 0.99 (0.96,1.01) | **0.96 (0.93,0.99)*** | 1.01 (0.99,1.04) |
|  | Model 3 | 1.02 (1.00,1.05) | 1.00 (0.98,1.03) | 0.98 (0.94,1.01) | **0.96 (0.93,1.00)*** | 1.01 (0.99,1.04) |
| Yes | Model 1 | 1.00 (0.94,1.07) | 1.03 (0.93,1.13) | 1.00 (0.91,1.10) | 1.08 (0.97,1.20) | 1.00 (0.93,1.09) |
|  | Model 2 | 1.00 (0.94,1.06) | 1.04 (0.95,1.14) | 1.04 (0.96,1.13) | 0.99 (0.91,1.08) | 0.95 (0.89,1.02) |
|  | Model 3 | 1.01 (0.95,1.07) | 1.06 (0.97,1.17) | 1.01 (0.91,1.12) | 0.98 (0.89,1.08) | 0.95 (0.89,1.03) |
| Osteoarthropathy |  |  |  |  |  |  |
| No | Model 1 | 1.02 (1.00,1.05) | 1.00 (0.97,1.04) | **0.96 (0.93,0.99)**** | 1.02 (0.98,1.05) | **1.03 (1.00,1.05)*** |
|  | Model 2 | 1.01 (0.99,1.03) | 1.00 (0.98,1.03) | 0.99 (0.96,1.01) | **0.96 (0.93,0.99)**** | 1.01 (0.99,1.03) |
|  | Model 3 | 1.01 (0.99,1.03) | 1.00 (0.98,1.03) | 0.98 (0.95,1.01) | **0.96 (0.92,0.99)*** | 1.01 (0.99,1.04) |
| Yes | Model 1 | 1.04 (0.99,1.11) | 1.06 (0.97,1.16) | 0.98 (0.91,1.06) | 1.08 (0.98,1.19) | 1.03 (0.97,1.10) |
|  | Model 2 | 1.04 (0.98,1.09) | 1.07 (0.98,1.18) | 1.02 (0.95,1.11) | 1.02 (0.93,1.12) | 1.02 (0.96,1.09) |
|  | Model 3 | 1.05 (0.99,1.11) | 1.09 (0.99,1.19) | 1.00 (0.92,1.10) | 1.04 (0.94,1.16) | 1.01 (0.95,1.08) |
| Smoking status |  |  |  |  |  |  |
| No | Model 1 | **1.03 (1.00,1.05)*** | 1.01 (0.98,1.04) | 0.98 (0.95,1.01) | 1.01 (0.97,1.04) | 1.02 (0.99,1.04) |
|  | Model 2 | 1.01 (0.99,1.03) | 1.01 (0.98,1.04) | 0.99 (0.97,1.02) | **0.96 (0.93,0.99)*** | 1.01 (0.99,1.03) |
|  | Model 3 | 1.01 (0.99,1.04) | 1.00 (0.97,1.03) | 0.98 (0.95,1.02) | 0.97 (0.93,1.00) | 1.01 (0.98,1.03) |
| Yes | Model 1 | 1.04 (0.99,1.09) | 1.05 (0.98,1.12) | **0.92 (0.87,0.97)**** | 1.01 (0.89,1.14) | 1.02 (0.96,1.08) |
|  | Model 2 | 1.02 (0.97,1.08) | 1.03 (0.97,1.09) | 0.96 (0.91,1.01) | 0.97 (0.87,1.09) | 1.03 (0.97,1.09) |
|  | Model 3 | 1.03 (0.98,1.09) | 1.04 (0.97,1.10) | 0.95 (0.90,1.02) | 0.97 (0.88,1.08) | 1.05 (0.99,1.11) |
| Alcohol consumption |  |  |  |  |  |  |
| No | Model 1 | **1.04 (1.01,1.07)**** | **1.03 (1.00,1.07)*** | 0.98 (0.95,1.01) | 1.00 (0.97,1.03) | 1.02 (0.99,1.04) |
|  | Model 2 | 1.01 (0.99,1.03) | 1.02 (0.99,1.05) | 0.99 (0.96,1.03) | **0.96 (0.93,0.99)**** | 1.01 (0.99,1.04) |
|  | Model 3 | 1.01 (0.99,1.04) | 1.02 (0.98,1.05) | 0.99 (0.95,1.02) | **0.96 (0.93,1.00)*** | 1.01 (0.98,1.03) |
| Yes | Model 1 | 1.02 (0.98,1.07) | 0.99 (0.95,1.04) | 0.96 (0.91,1.01) | 1.03 (0.93,1.14) | 1.00 (0.95,1.05) |
|  | Model 2 | 1.03 (0.99,1.08) | 0.99 (0.95,1.04) | 0.98 (0.93,1.02) | 1.00 (0.90,1.12) | 1.00 (0.96,1.05) |
|  | Model 3 | 1.03 (0.99,1.08) | 0.99 (0.94,1.04) | 0.96 (0.91,1.02) | 0.99 (0.89,1.11) | 1.01 (0.96,1.06) |
| Low grip strength |  |  |  |  |  |  |
| No | Model 1 | 1.01 (0.98,1.05) | 1.00 (0.95,1.04) | 0.97 (0.94,1.01) | 1.03 (0.98,1.08) | 1.01 (0.98,1.05) |
|  | Model 2 | 1.02 (0.99,1.06) | 1.01 (0.97,1.06) | 1.00 (0.96,1.03) | 0.98 (0.93,1.03) | 1.00 (0.96,1.03) |
|  | Model 3 | 1.02 (0.99,1.06) | 1.02 (0.98,1.06) | 0.99 (0.94,1.04) | 0.97 (0.92,1.03) | 0.98 (0.95,1.02) |
| Yes | Model 1 | 1.03 (1.00,1.06) | 1.01 (0.97,1.05) | 0.97 (0.94,1.01) | 1.01 (0.97,1.05) | **1.06 (1.02,1.09)**** |
|  | Model 2 | 1.01 (0.98,1.04) | 1.00 (0.97,1.04) | 0.99 (0.96,1.03) | **0.95 (0.91,0.99)*** | 1.03 (1.00,1.06) |
|  | Model 3 | 1.01 (0.98,1.04) | 1.00 (0.96,1.04) | 0.98 (0.94,1.02) | 0.96 (0.92,1.00) | 1.03 (1.00,1.06) |
| Anemia |  |  |  |  |  |  |
| No | Model 1 | 1.02 (0.99,1.05) | 1.01 (0.97,1.04) | 0.98 (0.95,1.01) | 1.02 (0.98,1.07) | 1.02 (0.99,1.05) |
|  | Model 2 | 1.01 (0.98,1.04) | 1.01 (0.97,1.04) | 1.01 (0.98,1.04) | **0.96 (0.92,1.00)*** | 1.00 (0.98,1.03) |
|  | Model 3 | 1.01 (0.98,1.04) | 1.00 (0.96,1.03) | 1.00 (0.96,1.04) | 0.96 (0.92,1.00) | 0.99 (0.97,1.02) |
| Yes | Model 1 | **1.05 (1.01,1.09)**** | 1.03 (0.98,1.08) | 0.95 (0.90,1.00) | 1.02 (0.96,1.07) | **1.05 (1.01,1.09)*** |
|  | Model 2 | 1.03 (0.99,1.06) | 1.02 (0.98,1.07) | **0.95 (0.90,1.00)*** | 0.98 (0.93,1.02) | 1.03 (0.99,1.07) |
|  | Model 3 | 1.03 (0.99,1.06) | 1.03 (0.98,1.08) | **0.93 (0.88,0.99)*** | 0.98 (0.92,1.03) | 1.04 (1.00,1.08) |
| Low density lipoprotein cholesterol |  |  |  |  |  |  |
| Normal | Model 1 | **1.04 (1.01,1.07)*** | 1.01 (0.97,1.05) | **0.94 (0.90,0.98)**** | 1.02 (0.98,1.06) | 1.03 (1.00,1.07) |
|  | Model 2 | 1.03 (1.00,1.06) | 1.00 (0.97,1.04) | 0.98 (0.94,1.01) | **0.95 (0.91,0.98)**** | 1.02 (0.98,1.05) |
|  | Model 3 | **1.03 (1.00,1.06)*** | 1.00 (0.97,1.04) | 0.99 (0.95,1.03) | **0.93 (0.90,0.97)***** | 1.02 (0.99,1.05) |
| High | Model 1 | 1.01 (0.98,1.05) | 1.02 (0.97,1.06) | 1.00 (0.96,1.04) | 1.03 (0.97,1.09) | 1.03 (0.99,1.06) |
|  | Model 2 | 0.99 (0.96,1.03) | 1.02 (0.98,1.06) | 1.01 (0.97,1.05) | 0.99 (0.93,1.05) | 1.01 (0.97,1.04) |
|  | Model 3 | 0.99 (0.96,1.03) | 1.02 (0.98,1.06) | 1.00 (0.96,1.04) | 1.00 (0.94,1.06) | 1.01 (0.97,1.04) |
| Total cholesterol |  |  |  |  |  |  |
| Normal | Model 1 | **1.04 (1.01,1.07)*** | 1.00 (0.96,1.05) | **0.93 (0.88,0.98)**** | 1.03 (0.99,1.07) | 1.02 (0.99,1.06) |
|  | Model 2 | 1.03 (0.99,1.06) | 1.00 (0.96,1.04) | 0.97 (0.93,1.01) | **0.95 (0.92,0.99)**** | 1.02 (0.99,1.06) |
|  | Model 3 | 1.03 (1.00,1.06) | 1.01 (0.97,1.05) | 0.97 (0.92,1.01) | **0.95 (0.91,0.98)**** | 1.02 (0.98,1.06) |
| High | Model 1 | 1.02 (0.98,1.05) | 1.02 (0.98,1.06) | 0.98 (0.95,1.01) | 1.01 (0.95,1.07) | 1.03 (1.00,1.06) |
|  | Model 2 | 1.00 (0.98,1.03) | 1.02 (0.98,1.06) | 1.01 (0.97,1.04) | 0.96 (0.91,1.02) | 1.01 (0.98,1.04) |
|  | Model 3 | 1.00 (0.97,1.03) | 1.01 (0.97,1.05) | 0.99 (0.96,1.03) | 0.97 (0.92,1.03) | 1.00 (0.97,1.03) |
| Serum creatinine |  |  |  |  |  |  |
| Normal | Model 1 | 1.03 (1.00,1.05) | 1.00 (0.97,1.03) | **0.96 (0.93,0.99)*** | **1.05 (1.01,1.08)*** | **1.03 (1.00,1.06)*** |
|  | Model 2 | 1.01 (0.98,1.03) | 1.00 (0.97,1.03) | 0.99 (0.96,1.02) | 0.98 (0.95,1.01) | 1.02 (0.99,1.04) |
|  | Model 3 | 1.01 (0.99,1.04) | 1.00 (0.97,1.04) | 0.98 (0.94,1.01) | 0.99 (0.95,1.02) | 1.02 (0.99,1.05) |
| Low | Model 1 | 1.02 (0.97,1.07) | 1.05 (0.99,1.12) | 0.98 (0.92,1.05) | 0.92 (0.85,1.00) | 1.01 (0.96,1.06) |
|  | Model 2 | 1.02 (0.97,1.07) | 1.04 (0.98,1.10) | 0.99 (0.93,1.06) | **0.89 (0.83,0.96)**** | 0.98 (0.94,1.03) |
|  | Model 3 | 1.02 (0.97,1.07) | 1.02 (0.96,1.08) | 1.00 (0.93,1.08) | **0.87 (0.80,0.95)**** | 0.98 (0.93,1.03) |
| Uric acid |  |  |  |  |  |  |
| Normal | Model 1 | **1.03 (1.00,1.05)*** | 1.00 (0.97,1.04) | **0.95 (0.92,0.98)**** | 1.01 (0.98,1.05) | 1.03 (1.00,1.05) |
|  | Model 2 | 1.01 (0.99,1.03) | 1.01 (0.98,1.04) | **0.97 (0.94,1.00)*** | **0.96 (0.93,0.99)*** | 1.01 (0.98,1.03) |
|  | Model 3 | 1.01 (0.99,1.04) | 1.01 (0.98,1.04) | 0.97 (0.93,1.00) | **0.96 (0.93,1.00)*** | 1.01 (0.98,1.03) |
| High | Model 1 | 1.03 (0.96,1.09) | 1.04 (0.98,1.10) | 0.99 (0.94,1.05) | **1.10 (1.01,1.20)*** | 1.03 (0.98,1.09) |
|  | Model 2 | 1.04 (0.98,1.10) | 1.01 (0.96,1.06) | 1.02 (0.97,1.07) | 1.00 (0.93,1.09) | 1.01 (0.96,1.06) |
|  | Model 3 | 1.05 (0.99,1.11) | 1.01 (0.96,1.07) | 1.01 (0.95,1.08) | 1.00 (0.91,1.09) | 1.02 (0.96,1.07) |
| Aspartate aminotransferase |  |  |  |  |  |  |
| Normal | Model 1 | **1.03 (1.00,1.05)*** | 1.02 (0.99,1.05) | 0.97 (0.94,1.00) | 1.01 (0.98,1.05) | 1.02 (0.99,1.05) |
|  | Model 2 | 1.01 (0.99,1.04) | 1.02 (0.99,1.05) | 0.99 (0.96,1.02) | **0.96 (0.93,0.99)*** | 1.01 (0.98,1.03) |
|  | Model 3 | 1.01 (0.99,1.04) | 1.01 (0.98,1.04) | 0.97 (0.94,1.01) | 0.97 (0.94,1.01) | 1.00 (0.98,1.03) |
| High | Model 1 | 1.02 (0.97,1.08) | 0.99 (0.93,1.05) | **0.94 (0.89,1.00)*** | 1.07 (0.99,1.15) | 1.05 (0.99,1.10) |
|  | Model 2 | 1.01 (0.96,1.07) | 1.00 (0.94,1.06) | 0.97 (0.92,1.03) | 0.99 (0.92,1.06) | 1.03 (0.98,1.08) |
|  | Model 3 | 1.01 (0.96,1.06) | 1.00 (0.94,1.07) | 1.00 (0.94,1.06) | 0.97 (0.89,1.04) | 1.03 (0.98,1.09) |
| Alanine aminotransferase |  |  |  |  |  |  |
| Normal | Model 1 | **1.03 (1.00,1.05)*** | 1.02 (0.99,1.05) | **0.96 (0.94,0.99)*** | 1.02 (0.98,1.05) | **1.03 (1.00,1.05)*** |
|  | Model 2 | 1.01 (0.99,1.04) | 1.01 (0.99,1.04) | 0.99 (0.96,1.02) | **0.96 (0.93,0.99)**** | 1.01 (0.98,1.03) |
|  | Model 3 | 1.01 (0.99,1.04) | 1.01 (0.98,1.04) | 0.98 (0.95,1.02) | **0.96 (0.93,1.00)*** | 1.01 (0.98,1.03) |
| High | Model 1 | 1.01 (0.91,1.12) | 0.99 (0.90,1.09) | 1.00 (0.91,1.10) | 1.08 (0.95,1.22) | 1.07 (0.98,1.16) |
|  | Model 2 | 1.01 (0.92,1.11) | 1.01 (0.92,1.11) | 0.99 (0.90,1.08) | 1.05 (0.94,1.19) | 1.06 (0.97,1.16) |
|  | Model 3 | 1.03 (0.93,1.14) | 1.01 (0.91,1.12) | 0.97 (0.86,1.08) | 1.01 (0.88,1.15) | 1.03 (0.94,1.14) |

**Notes:** ^a^, the reference group was non–elevated blood pressure; ^b^, the reference group was non–elevated fasting glucose; ^c^, the reference group was non–elevated triglycerides; ^d^, the reference group was non–reduced HDL cholesterol; ^e^, the reference group was non–abdominal obesity. *, *P*<0.05; **, *P*<0.01; ***, *P*<0.001. Model 1 was unadjusted model; Model 2 adjusted for sex, age, ethnic, marital status, educational attainment, occupation, annual income, cerebrovascular disease, rheumatism, osteoarthropathy, smoking status, and alcohol consumption;Model 3 further adjusted hand grip strength, anemia, total cholesterol, low density lipoprotein cholesterol, aspartate aminotransferase, alanine aminotransferasee, serum creatinine, and uric acid.

**Abbreviations:** MetS, metabolic syndrome; IADL, instrumental activities of daily living; PR, prevalence ratio; CI, confidence interval; HDL, high density lipoprotein.

**Supplementary Table S13.** Subgroup analysis of associations between individual MetS components and comorbid ADL­IADL disability

| Variables | Models | Comorbid ADL­IADL disability [PR (95% CI)] | | | | |
| --- | --- | --- | --- | --- | --- | --- |
|  |  | Elevated blood pressure ^a^ | Elevated fasting glucose ^b^ | Elevated triglycerides ^c^ | Reduced HDL cholesterol ^d^ | Abdominal obesity ^e^ |
| Gender |  |  |  |  |  |  |
| Male | Model 1 | 1.39 (0.92,2.11) | 1.30 (0.89,1.88) | 0.68 (0.43,1.09) | 1.69 (0.90,3.18) | 1.41 (0.97,2.06) |
|  | Model 2 | 1.25 (0.84,1.86) | 1.38 (0.96,1.98) | 0.98 (0.64,1.50) | 1.56 (0.87,2.80) | **1.45 (1.01,2.08)*** |
|  | Model 3 | 1.20 (0.80,1.79) | 1.28 (0.87,1.88) | 0.93 (0.56,1.56) | 1.52 (0.80,2.89) | 1.45 (0.99,2.13) |
| Female | Model 1 | 1.29 (0.96,1.73) | 1.23 (0.87,1.73) | 1.20 (0.88,1.65) | 1.36 (1.00,1.86) | 1.21 (0.93,1.59) |
|  | Model 2 | 1.13 (0.85,1.51) | 0.98 (0.70,1.36) | 1.14 (0.83,1.56) | 1.25 (0.93,1.67) | 1.26 (0.97,1.65) |
|  | Model 3 | 1.25 (0.92,1.69) | 1.03 (0.73,1.46) | 1.09 (0.73,1.62) | 1.25 (0.87,1.79) | 1.29 (0.97,1.70) |
| Age group |  |  |  |  |  |  |
| 60–69 years | Model 1 | 1.59 (1.00,2.53) | 1.07 (0.62,1.84) | 0.93 (0.57,1.54) | 0.73 (0.36,1.50) | 1.14 (0.74,1.76) |
|  | Model 2 | **1.69 (1.06,2.69)*** | 1.16 (0.69,1.97) | 1.02 (0.61,1.69) | 0.63 (0.30,1.30) | 1.02 (0.65,1.62) |
|  | Model 3 | **1.70 (1.07,2.71)*** | 1.13 (0.65,1.95) | 1.01 (0.54,1.89) | 0.62 (0.29,1.32) | 0.93 (0.58,1.49) |
| ≥ 70 years | Model 1 | 1.04 (0.79,1.37) | 1.26 (0.95,1.67) | 1.12 (0.83,1.51) | **1.63 (1.22,2.17)**** | **1.33 (1.04,1.70)*** |
|  | Model 2 | 1.06 (0.81,1.39) | 1.20 (0.90,1.59) | 1.11 (0.82,1.50) | **1.76 (1.31,2.38)***** | **1.43 (1.11,1.85)**** |
|  | Model 3 | 1.10 (0.82,1.47) | 1.25 (0.93,1.68) | 1.15 (0.80,1.66) | **1.64 (1.16,2.32)**** | **1.53 (1.17,2.00)**** |
| Ethnic |  |  |  |  |  |  |
| Non–zhuang | Model 1 | 0.92 (0.56,1.51) | 1.48 (0.87,2.52) | 1.20 (0.69,2.06) | **1.77 (1.01,3.09)*** | 0.72 (0.43,1.20) |
|  | Model 2 | 0.68 (0.42,1.12) | 1.56 (0.96,2.54) | 1.54 (0.90,2.63) | **1.87 (1.06,3.29)*** | 0.75 (0.47,1.20) |
|  | Model 3 | 0.78 (0.45,1.35) | 1.58 (0.95,2.62) | 1.35 (0.63,2.89) | 2.14 (1.00,4.57) | **0.60 (0.36,1.00)*** |
| Zhuang | Model 1 | **1.47 (1.12,1.95)**** | 1.18 (0.88,1.58) | 0.90 (0.67,1.22) | 1.34 (0.98,1.84) | **1.41 (1.10,1.79)**** |
|  | Model 2 | 1.26 (0.97,1.65) | 1.06 (0.81,1.40) | 0.95 (0.72,1.26) | 1.16 (0.87,1.55) | **1.54 (1.22,1.95)***** |
|  | Model 3 | 1.26 (0.96,1.65) | 1.03 (0.77,1.38) | 0.97 (0.69,1.36) | 1.11 (0.79,1.55) | **1.59 (1.24,2.03)***** |
| Marital status |  |  |  |  |  |  |
| Partnered | Model 1 | **1.57 (1.13,2.19)**** | 1.20 (0.84,1.71) | 0.89 (0.61,1.29) | 1.10 (0.70,1.72) | 1.16 (0.85,1.58) |
|  | Model 2 | **1.40 (1.01,1.94)*** | 1.22 (0.86,1.72) | 1.00 (0.71,1.41) | 1.16 (0.75,1.80) | 1.19 (0.87,1.63) |
|  | Model 3 | **1.42 (1.02,1.98)*** | 1.20 (0.84,1.71) | 1.16 (0.78,1.74) | 1.04 (0.65,1.66) | 1.14 (0.82,1.57) |
| Single | Model 1 | 1.02 (0.72,1.44) | 1.30 (0.91,1.86) | 1.13 (0.79,1.62) | **1.54 (1.09,2.18)*** | **1.39 (1.02,1.88)*** |
|  | Model 2 | 0.96 (0.70,1.31) | 1.08 (0.77,1.49) | 1.14 (0.79,1.65) | **1.46 (1.06,2.02)*** | **1.45 (1.08,1.93)*** |
|  | Model 3 | 1.04 (0.74,1.45) | 1.14 (0.79,1.64) | 0.89 (0.56,1.41) | **1.71 (1.13,2.58)*** | **1.52 (1.11,2.07)**** |
| Educational attainment |  |  |  |  |  |  |
| Less than primary school | Model 1 | 1.36 (0.99,1.88) | 1.24 (0.87,1.78) | 1.12 (0.79,1.59) | **1.55 (1.11,2.16)**** | 1.14 (0.85,1.52) |
|  | Model 2 | 1.15 (0.85,1.56) | 0.97 (0.70,1.34) | 1.14 (0.82,1.58) | **1.37 (1.02,1.84)*** | 1.24 (0.94,1.65) |
|  | Model 3 | 1.26 (0.92,1.73) | 0.98 (0.69,1.37) | 1.12 (0.74,1.69) | 1.33 (0.92,1.92) | 1.28 (0.95,1.73) |
| Primary school | Model 1 | 1.33 (0.87,2.05) | 1.31 (0.85,1.99) | 1.02 (0.63,1.64) | 1.03 (0.59,1.79) | 1.33 (0.90,1.98) |
|  | Model 2 | 1.20 (0.80,1.82) | 1.42 (0.94,2.15) | 1.08 (0.69,1.68) | 1.15 (0.65,2.03) | 1.36 (0.90,2.06) |
|  | Model 3 | 1.25 (0.81,1.94) | 1.37 (0.88,2.12) | 1.04 (0.64,1.71) | 1.21 (0.65,2.24) | 1.36 (0.88,2.09) |
| High school and above | Model 1 | 1.23 (0.61,2.48) | 1.41 (0.72,2.76) | 0.63 (0.31,1.28) | 0.96 (0.29,3.19) | 1.59 (0.87,2.91) |
|  | Model 2 | 1.03 (0.51,2.09) | 1.24 (0.60,2.55) | 0.69 (0.33,1.48) | 1.23 (0.36,4.23) | 1.73 (0.97,3.07) |
|  | Model 3 | 0.95 (0.47,1.95) | 1.19 (0.52,2.72) | 0.61 (0.19,1.99) | 1.14 (0.33,3.91) | 1.80 (0.94,3.46) |
| Occupation |  |  |  |  |  |  |
| Non–farmer | Model 1 | 0.53 (0.21,1.29) | 1.69 (0.63,4.52) | 0.89 (0.33,2.40) | 0.75 (0.20,2.85) | **2.69 (1.07,6.73)*** |
|  | Model 2 | 0.60 (0.26,1.41) | 2.36 (0.90,6.15) | 1.46 (0.50,4.23) | 0.55 (0.18,1.63) | 2.62 (0.97,7.07) |
|  | Model 3 | 0.63 (0.27,1.48) | **2.80 (1.13,6.98)*** | 1.41 (0.35,5.74) | 0.58 (0.15,2.25) | **2.77 (1.08,7.12)*** |
| Farmer | Model 1 | **1.44 (1.12,1.86)**** | 1.23 (0.95,1.60) | 0.98 (0.74,1.28) | **1.49 (1.12,1.97)**** | 1.23 (0.98,1.55) |
|  | Model 2 | 1.21 (0.95,1.54) | 1.10 (0.86,1.41) | 1.03 (0.80,1.34) | **1.32 (1.02,1.72)*** | **1.28 (1.02,1.60)*** |
|  | Model 3 | **1.29 (1.00,1.65)*** | 1.07 (0.82,1.40) | 1.04 (0.76,1.41) | 1.27 (0.93,1.74) | **1.28 (1.01,1.63)*** |
| Annual income |  |  |  |  |  |  |
| ＜10000 renminbi | Model 1 | 1.32 (0.94,1.85) | 1.35 (0.96,1.90) | 1.01 (0.70,1.46) | 1.62 (1.12,2.33) | 1.21 (0.88,1.66) |
|  | Model 2 | 1.17 (0.85,1.61) | 1.24 (0.89,1.71) | 1.13 (0.80,1.61) | **1.47 (1.04,2.09)*** | 1.15 (0.84,1.59) |
|  | Model 3 | 1.28 (0.92,1.78) | 1.20 (0.84,1.70) | 1.11 (0.72,1.71) | **1.54 (1.01,2.33)*** | 1.08 (0.76,1.52) |
| 10000–29999 renminbi | Model 1 | 1.36 (0.81,2.28) | 1.21 (0.69,2.13) | 0.99 (0.57,1.72) | 1.20 (0.64,2.26) | 1.51 (0.96,2.37) |
|  | Model 2 | 1.20 (0.70,2.06) | 1.24 (0.69,2.21) | 1.10 (0.62,1.96) | 1.12 (0.61,2.06) | 1.29 (0.82,2.01) |
|  | Model 3 | 1.31 (0.74,2.31) | 1.31 (0.73,2.36) | 0.99 (0.51,1.93) | 1.04 (0.53,2.05) | 1.45 (0.89,2.36) |
| ≥ 30000 renminbi | Model 1 | 1.33 (0.84,2.12) | 1.04 (0.63,1.73) | 0.78 (0.47,1.30) | 1.17 (0.67,2.04) | **1.49 (1.00,2.22)*** |
|  | Model 2 | 1.23 (0.81,1.88) | 0.89 (0.56,1.41) | 0.84 (0.51,1.40) | 1.00 (0.59,1.67) | **1.71 (1.16,2.53)**** |
|  | Model 3 | 1.25 (0.80,1.97) | 0.89 (0.55,1.44) | 0.94 (0.50,1.75) | 0.83 (0.43,1.59) | **2.11 (1.38,3.20)***** |
| Cerebrovascular disease |  |  |  |  |  |  |
| No | Model 1 | **1.33 (1.04,1.71)*** | 1.28 (0.99,1.66) | 0.91 (0.69,1.19) | **1.38 (1.04,1.84)*** | **1.29 (1.03,1.61)*** |
|  | Model 2 | 1.13 (0.89,1.43) | 1.16 (0.90,1.48) | 1.00 (0.77,1.30) | 1.30 (0.99,1.70) | **1.36 (1.09,1.69)**** |
|  | Model 3 | 1.19 (0.93,1.53) | 1.14 (0.88,1.48) | 0.98 (0.72,1.35) | 1.25 (0.91,1.71) | **1.37 (1.09,1.73)**** |
| Yes | Model 1 | 1.29 (0.45,3.71) | 0.77 (0.20,3.00) | **2.75 (1.04,7.26)*** | 2.35 (0.85,6.48) | 1.14 (0.44,2.95) |
|  | Model 2 | 2.10 (0.64,6.88) | 0.77 (0.23,2.62) | 2.81 (0.95,8.30) | 1.40 (0.52,3.79) | 0.93 (0.32,2.66) |
|  | Model 3 | 2.25 (0.77,6.59) | 0.41 (0.11,1.47) | **10.37 (1.79,60.10)**** | 0.87 (0.09,8.18) | 0.69 (0.21,2.30) |
| Rheumatism |  |  |  |  |  |  |
| No | Model 1 | **1.34 (1.03,1.73)*** | 1.24 (0.95,1.63) | 0.99 (0.75,1.30) | 1.28 (0.95,1.73) | **1.29 (1.03,1.63)*** |
|  | Model 2 | 1.16 (0.91,1.49) | 1.14 (0.89,1.47) | 1.08 (0.83,1.41) | 1.21 (0.91,1.60) | **1.39 (1.11,1.75)**** |
|  | Model 3 | 1.23 (0.95,1.59) | 1.13 (0.86,1.48) | 1.08 (0.79,1.47) | 1.16 (0.83,1.62) | **1.40 (1.10,1.79)**** |
| Yes | Model 1 | 1.34 (0.68,2.64) | 1.27 (0.56,2.90) | 0.86 (0.37,1.97) | **3.14 (1.54,6.39)**** | 1.07 (0.53,2.16) |
|  | Model 2 | 1.48 (0.78,2.80) | 1.39 (0.50,3.88) | 0.70 (0.24,2.00) | **2.58 (1.27,5.24)**** | 0.73 (0.36,1.48) |
|  | Model 3 | 1.51 (0.82,2.80) | 1.26 (0.42,3.72) | 0.76 (0.24,2.42) | 2.33 (0.96,5.67) | 0.70 (0.32,1.54) |
| Osteoarthropathy |  |  |  |  |  |  |
| No | Model 1 | 1.25 (0.96,1.62) | 1.18 (0.90,1.54) | 1.09 (0.83,1.43) | 1.32 (0.98,1.78) | 1.26 (1.00,1.60) |
|  | Model 2 | 1.07 (0.83,1.36) | 1.07 (0.83,1.38) | 1.16 (0.89,1.50) | 1.18 (0.90,1.55) | **1.35 (1.07,1.70)*** |
|  | Model 3 | 1.12 (0.86,1.44) | 1.05 (0.80,1.38) | 1.12 (0.82,1.53) | 1.16 (0.84,1.60) | **1.36 (1.07,1.74)*** |
| Yes | Model 1 | **2.00 (1.06,3.78)*** | **2.30 (1.16,4.56)*** | **0.29 (0.09,0.90)*** | **2.75 (1.38,5.47)**** | 1.24 (0.71,2.17) |
|  | Model 2 | 1.81 (0.98,3.36) | **2.22 (1.22,4.06)**** | 0.34 (0.11,1.04) | **2.55 (1.37,4.74)**** | 1.25 (0.71,2.18) |
|  | Model 3 | **1.98 (1.06,3.70)*** | **2.11 (1.14,3.90)*** | 0.44 (0.15,1.29) | **2.38 (1.13,5.04)*** | 1.39 (0.76,2.55) |
| Smoking status |  |  |  |  |  |  |
| No | Model 1 | 1.26 (0.97,1.62) | 1.18 (0.90,1.56) | 1.14 (0.87,1.49) | **1.38 (1.04,1.83)*** | 1.22 (0.97,1.54) |
|  | Model 2 | 1.07 (0.84,1.36) | 1.07 (0.82,1.39) | 1.16 (0.89,1.51) | **1.31 (1.00,1.72)*** | **1.33 (1.06,1.68)*** |
|  | Model 3 | 1.12 (0.87,1.45) | 1.08 (0.81,1.44) | 1.10 (0.80,1.52) | 1.35 (0.97,1.87) | **1.37 (1.07,1.74)*** |
| Yes | Model 1 | 2.13 (0.96,4.71) | 1.76 (0.95,3.24) | **0.29 (0.11,0.82)*** | 1.33 (0.46,3.87) | 1.57 (0.83,2.97) |
|  | Model 2 | 1.94 (0.90,4.15) | 1.45 (0.87,2.42) | 0.52 (0.20,1.37) | 0.98 (0.21,4.54) | 1.31 (0.67,2.54) |
|  | Model 3 | **2.45 (1.06,5.64)*** | 1.52 (0.94,2.45) | 0.63 (0.21,1.90) | 1.11 (0.43,2.90) | 1.36 (0.66,2.79) |
| Alcohol consumption |  |  |  |  |  |  |
| No | Model 1 | **1.40 (1.08,1.82)*** | 1.30 (0.98,1.72) | 1.12 (0.85,1.48) | 1.28 (0.96,1.71) | 1.22 (0.97,1.55) |
|  | Model 2 | 1.16 (0.90,1.48) | 1.13 (0.86,1.48) | 1.13 (0.86,1.49) | 1.26 (0.96,1.64) | **1.36 (1.08,1.71)**** |
|  | Model 3 | 1.20 (0.93,1.56) | 1.13 (0.85,1.50) | 1.05 (0.75,1.47) | 1.31 (0.95,1.82) | **1.36 (1.07,1.74)*** |
| Yes | Model 1 | 1.16 (0.62,2.16) | 1.31 (0.73,2.33) | 0.65 (0.33,1.31) | 2.05 (0.89,4.72) | 1.19 (0.65,2.16) |
|  | Model 2 | 1.21 (0.67,2.21) | 1.25 (0.72,2.20) | 0.69 (0.37,1.31) | 1.92 (0.85,4.32) | 1.17 (0.61,2.24) |
|  | Model 3 | 1.29 (0.67,2.49) | 1.24 (0.68,2.28) | 0.76 (0.34,1.70) | 1.45 (0.55,3.83) | 1.22 (0.60,2.49) |
| Low grip strength |  |  |  |  |  |  |
| No | Model 1 | 1.28 (0.79,2.07) | 1.28 (0.75,2.18) | 0.96 (0.57,1.61) | 1.09 (0.58,2.07) | 1.19 (0.75,1.89) |
|  | Model 2 | 1.20 (0.74,1.94) | 1.20 (0.70,2.03) | 0.99 (0.60,1.63) | 0.98 (0.52,1.84) | 1.16 (0.72,1.85) |
|  | Model 3 | 1.23 (0.78,1.95) | 1.15 (0.68,1.95) | 1.18 (0.65,2.14) | 0.87 (0.44,1.71) | 1.01 (0.63,1.63) |
| Yes | Model 1 | **1.34 (1.01,1.78)*** | 1.19 (0.89,1.60) | 1.07 (0.79,1.45) | **1.40 (1.02,1.92)*** | **1.43 (1.12,1.84)**** |
|  | Model 2 | 1.17 (0.90,1.54) | 1.09 (0.83,1.44) | 1.10 (0.82,1.48) | 1.29 (0.96,1.75) | **1.51 (1.18,1.94)**** |
|  | Model 3 | 1.20 (0.91,1.58) | 1.11 (0.83,1.48) | 1.02 (0.72,1.45) | 1.37 (0.98,1.91) | **1.54 (1.19,1.99)**** |
| Anemia |  |  |  |  |  |  |
| No | Model 1 | **1.55 (1.08,2.23)*** | 1.31 (0.93,1.85) | 0.93 (0.65,1.32) | 1.27 (0.84,1.92) | 1.16 (0.85,1.58) |
|  | Model 2 | 1.39 (0.98,1.97) | 1.12 (0.80,1.56) | 1.04 (0.74,1.45) | 1.19 (0.81,1.75) | 1.18 (0.87,1.59) |
|  | Model 3 | **1.45 (1.01,2.07)*** | 1.09 (0.77,1.53) | 1.11 (0.75,1.65) | 1.19 (0.77,1.84) | 1.13 (0.83,1.55) |
| Yes | Model 1 | 1.25 (0.90,1.73) | 1.25 (0.86,1.81) | 1.24 (0.84,1.83) | **1.53 (1.06,2.20)*** | **1.47 (1.09,1.99)*** |
|  | Model 2 | 1.00 (0.72,1.38) | 1.21 (0.84,1.73) | 1.14 (0.79,1.65) | **1.44 (1.01,2.05)*** | 1.53 (1.11,2.12) |
|  | Model 3 | 1.05 (0.75,1.47) | 1.21 (0.83,1.78) | 0.97 (0.63,1.51) | 1.40 (0.90,2.18) | **1.57 (1.11,2.21)*** |
| Low density lipoprotein cholesterol |  |  |  |  |  |  |
| Normal | Model 1 | 1.28 (0.94,1.73) | **1.57 (1.15,2.15)**** | 0.86 (0.58,1.27) | 1.37 (0.97,1.93) | 1.34 (0.99,1.81) |
|  | Model 2 | 1.04 (0.78,1.40) | **1.40 (1.04,1.89)*** | 1.02 (0.71,1.45) | 1.21 (0.88,1.64) | 1.34 (1.00,1.81) |
|  | Model 3 | 1.11 (0.82,1.51) | **1.41 (1.03,1.93)*** | 1.08 (0.74,1.59) | 1.10 (0.77,1.57) | **1.39 (1.03,1.89)*** |
| High | Model 1 | 1.45 (0.97,2.18) | 0.82 (0.52,1.31) | 1.13 (0.77,1.67) | 1.51 (0.93,2.47) | 1.20 (0.86,1.66) |
|  | Model 2 | 1.33 (0.88,2.00) | 0.76 (0.47,1.22) | 1.11 (0.76,1.61) | 1.49 (0.91,2.43) | 1.31 (0.94,1.83) |
|  | Model 3 | 1.34 (0.88,2.02) | 0.75 (0.46,1.22) | 1.14 (0.76,1.70) | 1.51 (0.91,2.51) | 1.29 (0.90,1.83) |
| Total cholesterol |  |  |  |  |  |  |
| Normal | Model 1 | 1.17 (0.84,1.62) | **1.50 (1.06,2.13)*** | 0.82 (0.51,1.33) | 1.37 (0.96,1.94) | 1.29 (0.92,1.79) |
|  | Model 2 | 0.93 (0.68,1.27) | 1.25 (0.89,1.75) | 0.92 (0.59,1.43) | 1.21 (0.87,1.69) | **1.47 (1.07,2.03)*** |
|  | Model 3 | 1.04 (0.75,1.45) | 1.36 (0.96,1.95) | 0.88 (0.52,1.50) | 1.15 (0.81,1.62) | **1.51 (1.09,2.10)*** |
| High | Model 1 | **1.56 (1.08,2.25)*** | 1.05 (0.73,1.51) | 1.09 (0.79,1.50) | 1.45 (0.89,2.34) | 1.28 (0.95,1.71) |
|  | Model 2 | 1.40 (0.97,2.03) | 1.00 (0.69,1.44) | 1.15 (0.84,1.57) | 1.38 (0.86,2.22) | 1.30 (0.97,1.75) |
|  | Model 3 | 1.37 (0.95,1.99) | 0.92 (0.62,1.37) | 1.12 (0.80,1.56) | 1.47 (0.91,2.36) | 1.26 (0.92,1.73) |
| Serum creatinine |  |  |  |  |  |  |
| Normal | Model 1 | 1.28 (0.97,1.69) | 1.13 (0.83,1.53) | 1.07 (0.80,1.43) | **1.68 (1.26,2.26)***** | 1.28 (0.99,1.64) |
|  | Model 2 | 1.09 (0.84,1.42) | 1.04 (0.78,1.38) | 1.16 (0.88,1.52) | **1.52 (1.15,2.00)**** | **1.39 (1.09,1.78)**** |
|  | Model 3 | 1.18 (0.89,1.55) | 1.06 (0.78,1.44) | 1.25 (0.89,1.75) | 1.37 (0.97,1.93) | **1.43 (1.10,1.86)**** |
| Low | Model 1 | 1.34 (0.82,2.19) | **1.68 (1.04,2.72)*** | 0.74 (0.39,1.43) | 0.72 (0.30,1.77) | 1.20 (0.76,1.91) |
|  | Model 2 | 1.29 (0.79,2.09) | 1.53 (0.93,2.52) | 0.73 (0.37,1.44) | 0.74 (0.31,1.73) | 1.08 (0.66,1.77) |
|  | Model 3 | 1.29 (0.78,2.14) | 1.39 (0.82,2.35) | 0.65 (0.28,1.48) | 0.80 (0.33,1.93) | 1.04 (0.60,1.80) |
| Uric acid |  |  |  |  |  |  |
| Normal | Model 1 | **1.43 (1.09,1.87)**** | 1.17 (0.86,1.58) | 0.90 (0.64,1.27) | 1.37 (1.00,1.88) | 1.17 (0.91,1.52) |
|  | Model 2 | 1.20 (0.92,1.56) | 1.13 (0.84,1.51) | 0.97 (0.70,1.33) | 1.22 (0.91,1.64) | 1.27 (0.98,1.64) |
|  | Model 3 | 1.27 (0.97,1.66) | 1.08 (0.79,1.47) | 0.90 (0.61,1.32) | 1.24 (0.88,1.75) | 1.30 (0.99,1.70) |
| High | Model 1 | 0.84 (0.50,1.41) | 1.42 (0.89,2.26) | 0.98 (0.64,1.51) | **1.85 (1.08,3.19)*** | 1.50 (0.97,2.32) |
|  | Model 2 | 0.86 (0.53,1.38) | 1.13 (0.74,1.74) | 1.15 (0.76,1.75) | 1.53 (0.91,2.56) | 1.48 (0.99,2.24) |
|  | Model 3 | 0.93 (0.55,1.58) | 1.27 (0.79,2.03) | 1.27 (0.74,2.17) | 1.38 (0.69,2.74) | 1.37 (0.87,2.14) |
| Aspartate aminotransferase |  |  |  |  |  |  |
| Normal | Model 1 | 1.22 (0.94,1.59) | 1.26 (0.95,1.68) | 1.08 (0.80,1.45) | 1.26 (0.92,1.72) | 1.23 (0.97,1.58) |
|  | Model 2 | 1.02 (0.80,1.31) | 1.14 (0.87,1.48) | 1.14 (0.86,1.51) | 1.19 (0.90,1.59) | **1.32 (1.04,1.69)*** |
|  | Model 3 | 1.06 (0.82,1.37) | 1.14 (0.86,1.51) | 1.06 (0.75,1.50) | 1.24 (0.88,1.76) | **1.37 (1.07,1.77)*** |
| High | Model 1 | **2.08 (1.09,3.94)*** | 1.22 (0.70,2.12) | 0.72 (0.41,1.27) | **2.35 (1.36,4.05)**** | 1.35 (0.83,2.18) |
|  | Model 2 | 1.87 (0.99,3.54) | 1.10 (0.63,1.91) | 0.77 (0.44,1.35) | **1.96 (1.13,3.40)*** | 1.44 (0.90,2.29) |
|  | Model 3 | 1.92 (0.98,3.76) | 1.21 (0.66,2.20) | 1.04 (0.50,2.17) | 1.57 (0.79,3.09) | 1.42 (0.85,2.36) |
| Alanine aminotransferase |  |  |  |  |  |  |
| Normal | Model 1 | **1.32 (1.03,1.69)*** | **1.32 (1.02,1.71)*** | 1.02 (0.78,1.33) | **1.39 (1.04,1.86)*** | 1.22 (0.97,1.53) |
|  | Model 2 | 1.13 (0.89,1.43) | 1.20 (0.94,1.54) | 1.09 (0.84,1.42) | 1.28 (0.98,1.66) | **1.28 (1.02,1.60)*** |
|  | Model 3 | 1.17 (0.92,1.49) | 1.19 (0.91,1.54) | 1.06 (0.78,1.43) | 1.29 (0.95,1.75) | **1.32 (1.04,1.67)*** |
| High | Model 1 | 1.40 (0.41,4.74) | 0.63 (0.20,2.00) | 0.70 (0.29,1.70) | 1.96 (0.77,5.01) | 2.44 (0.98,6.11) |
|  | Model 2 | 1.18 (0.37,3.75) | 0.58 (0.19,1.75) | 0.62 (0.25,1.55) | 1.51 (0.53,4.32) | **2.53 (1.02,6.30)*** |
|  | Model 3 | 2.59 (0.30,21.98) | 0.63 (0.13,3.03) | 1.01 (0.19,5.34) | 0.59 (0.11,3.11) | 1.45 (0.59,3.52) |

**Notes:** ^a^, the reference group was non–elevated blood pressure; ^b^, the reference group was non–elevated fasting glucose; ^c^, the reference group was non–elevated triglycerides; ^d^, the reference group was non–reduced HDL cholesterol; ^e^, the reference group was non–abdominal obesity. *, *P*<0.05; **, *P*<0.01; ***, *P*<0.001. Model 1 was unadjusted model; Model 2 adjusted for sex, age, ethnic, marital status, educational attainment, occupation, annual income, cerebrovascular disease, rheumatism, osteoarthropathy, smoking status, and alcohol consumption; Model 3 further adjusted hand grip strength, anemia, total cholesterol, low density lipoprotein cholesterol, aspartate aminotransferase, alanine aminotransferasee, serum creatinine, and uric acid.

**Abbreviations:** MetS, metabolic syndrome; ADL, activities of daily living; IADL, instrumental activities of daily living; PR, prevalence ratio; CI, confidence interval; HDL, high density lipoprotein.

**Supplementary Table S14.** Interaction effects of covariates and MetS on functional disability

| Functional disability/Covariates | Multiplicative interaction [PR (95%CI)] | Additive interaction [PR (95%CI)] | | |
| --- | --- | --- | --- | --- |
|  |  | RERI | AP | SI |
| **ADL disability** |  |  |  |  |
| Gender | 1.43 (0.88, 2.31) | 0.45 (–0.16, 1.06) | 0.28 (–0.08, 0.63) | 3.63 (0.13, 101.52) |
| Age group | 0.89 (0.54, 1.46) | 0.88 (–0.36, 2.12) | 0.19 (–0.05, 0.44) | 1.33 (0.89, 1.99) |
| Ethnic | 1.003 (0.60, 1.68) | –0.15 (–0.85, 0.55) | –0.14 (–0.75, 0.48) | 0.46 (0.03, 7.81) |
| Marital status | 1.14 (0.73, 1.76) | 0.66 (–0.25, 1.56) | 0.23 (–0.04, 0.51) | 1.57 (0.84, 2.94) |
| Educational attainment (primary school ) | 0.93 (0.57, 1.52) | –0.15 (–0.86, 0.56) | –0.10 (–0.60, 0.40) | 0.76 (0.21, 2.77) |
| Educational attainment (high school and above) | 0.88 (0.45, 1.72) | –0.36 (–1.09, 0.37) | –0.36 (–1.22, 0.50) | 0.003 (0.001, 17.27) |
| Occupation | 1.16 (0.47, 2.84) | 0.59 (–0.59, 1.76) | 0.23 (–0.25, 0.70) | 1.6 (0.43, 5.98) |
| Annual income (10000–29999 renminbi) | 1.06 (0.60, 1.87) | –0.15 (–0.84, 0.53) | –0.14 (–0.79, 0.51) | 0.43 (0.01, 33.87) |
| Annual income ( ≥ 30000 renminbi) | 1.02 (0.61, 1.69) | –0.19 (–0.81, 0.43) | –0.18 (–0.78, 0.43) | 0.29 (0.01, 43.12) |
| Cerebrovascular disease | 1.30 (0.46, 3.67) | 0.56 (–1.53, 2.66) | 0.25 (–0.47, 0.97) | 1.82 (0.29, 11.57) |
| Rheumatism | 1.26 (0.59, 2.68) | 0.36 (–0.94, 1.65) | 0.19 (–0.39, 0.76) | 1.66 (0.33, 8.30) |
| Osteoarthropathy | 1.25 (0.60, 2.60) | 0.37 (–0.94, 1.68) | 0.19 (–0.37, 0.75) | 1.66 (0.36, 7.72) |
| Smoking status | 1.17 (0.60, 2.30) | 0.11 (–0.82, 1.03) | 0.07 (–0.52, 0.66) | 1.27 (0.17, 9.64) |
| Alcohol consumption | 0.67 (0.35, 1.28) | –0.60 (–1.27, 0.07) | –0.66 (–1.67, 0.35) | NA^a^ |
| Low handgrip strength | 1.15 (0.69, 1.92) | **1.08 (0.03, 2.12)** | **0.29 (0.05, 0.53)** | 1.65 (0.98, 2.80) |
| Anemia | 1.49 (0.96, 2.31) | **1.15 (0.22, 2.08)** | **0.39 (0.16, 0.62)** | **2.39 (1.15, 4.96)** |
| Low density lipoprotein cholesterol | 0.78 (0.49, 1.24) | –0.40 (–1.08, 0.27) | –0.30 (–0.85, 0.26) | 0.48 (0.12, 1.81) |
| Total cholesterol | 0.97 (0.62, 1.50) | –0.11 (–0.76, 0.53) | –0.08 (–0.53, 0.37) | 0.8 (0.24, 2.68) |
| Serum creatinine | 1.05 (0.61, 1.78) | 0.14 (–0.74, 1.01) | 0.07 (–0.38, 0.52) | 1.19 (0.40, 3.58) |
| Uric acid | 0.91 (0.56, 1.48) | –0.01 (–0.80, 0.78) | 0.00 (–0.42, 0.41) | 0.99 (0.41, 2.39) |
| Aspartate aminotransferase | 1.34 (0.80, 2.25) | 0.35 (–0.39, 1.10) | 0.21 (–0.17, 0.59) | 1.98 (0.44, 9.04) |
| Alanine aminotransferase | 1.01 (0.43, 2.36) | –0.06 (–1.11, 0.98) | –0.04 (–0.78, 0.70) | 0.88 (0.09, 8.09) |
| **IADL disability** |  |  |  |  |
| Gender | 0.99 (0.74, 1.36) | –0.03 (–0.40, 0.33) | –0.02 (–0.21, 0.17) | 0.96 (0.66, 1.41) |
| Age group | **1.46 (1.12, 1.90)** | **0.48 (0.17, 0.80)** | **0.23 (0.09, 0.36)** | **1.74 (1.15, 2.64)** |
| Ethnic | 0.98 (0.75, 1.30) | –0.02 (–0.29, 0.26) | –0.02 (–0.34, 0.30) | 1.13 (0.13, 9.80) |
| Marital status | 1.06 (0.84, 1.33) | 0.08 (–0.23, 0.39) | 0.05 (–0.13, 0.23) | 1.14 (0.70, 1.87) |
| Educational attainment (primary school ) | 1.05 (0.81, 1.37) | 0.04 (–0.16, 0.23) | 0.06 (–0.24, 0.36) | 0.91 (0.54, 1.52) |
| Educational attainment (high school and above) | 1.06 (0.65, 1.71) | 0.02 (–0.16, 0.21) | 0.08 (–0.54, 0.71) | 0.97 (0.75, 1.25) |
| Occupation | 1.39 (0.69, 2.80) | 0.41 (–0.19, 1.01) | 0.15 (–0.08, 0.38) | 1.30 (0.80, 2.11) |
| Annual income (10000–29999 renminbi) | 0.85 (0.63, 1.13) | –0.17 (–0.42, 0.09) | –0.22 (–0.60, 0.15) | 2.80 (0.29, 27.18) |
| Annual income ( ≥ 30000 renminbi) | 0.83 (0.63, 1.11) | –0.17 (–0.39, 0.05) | –0.29 (–0.71, 0.13) | 1.63 (0.75, 3.52) |
| Cerebrovascular disease | 1.24 (0.67, 2.30) | 0.21 (–0.46, 0.89) | 0.19 (–0.31, 0.69) | NA^a^ |
| Rheumatism | 0.87 (0.55, 1.39) | –0.13 (–0.56, 0.29) | –0.15 (–0.69, 0.39) | NA^a^ |
| Osteoarthropathy | 1.18 (0.77, 1.81) | 0.15 (–0.27, 0.58) | 0.15 (–0.22, 0.52) | NA^a^ |
| Smoking status | 1.26 (0.80, 1.98) | 0.14 (–0.15, 0.42) | 0.22 (–0.17, 0.62) | 0.74 (0.37, 1.48) |
| Alcohol consumption | 0.76 (0.51, 1.14) | –0.16 (–0.38, 0.06) | –0.36 (–0.94, 0.23) | 1.41 (0.87, 2.31) |
| Low handgrip strength | 1.24 (0.95, 1.60) | 0.30 (–0.01, 0.61) | 0.16 (0.01, 0.32) | 1.54 (0.92, 2.58) |
| Anemia | **1.24 (0.99, 1.56)** | **0.30 (0.01, 0.60)** | **0.19 (0.02, 0.36)** | 2.07 (0.92, 4.62) |
| Low density lipoprotein cholesterol | 1.08 (0.85, 1.37) | 0.07 (–0.16, 0.30) | 0.07 (–0.16, 0.31) | 0.34 (0.01, 39.95) |
| Total cholesterol | 0.99 (0.78, 1.25) | –0.01 (–0.25, 0.23) | –0.01 (–0.26, 0.23) | 2.02 (0.01, 18.34) |
| Serum creatinine | 1.09 (0.83, 1.43) | 0.10 (–0.21, 0.42) | 0.08 (–0.16, 0.32) | 1.70 (0.33, 8.77) |
| Uric acid | 1.24 (0.96, 1.61) | 0.22 (–0.05, 0.48) | 0.20 (–0.02, 0.41) | NA^a^ |
| Aspartate aminotransferase | 1.07 (0.81, 1.41) | 0.06 (–0.21, 0.34) | 0.06 (–0.20, 0.33) | NA^a^ |
| Alanine aminotransferase | 1.47 (0.93, 2.31) | 0.31 (–0.08, 0.69) | 0.31 (–0.01, 0.64) | 0.07 (0.01, 33.92) |
| **Comorbid ADL**–**IADL disability** |  |  |  |  |
| Gender | 1.52 (0.90, 2.57) | 0.56 (–0.08, 1.20) | 0.33 (–0.02, 0.69) | 5.87 (0.03, 1102.02) |
| Age group | 0.99 (0.58, 1.72) | 1.15 (–0.19, 2.49) | 0.24 (0.01, 0.48) | 1.43 (0.94, 2.18) |
| Ethnic | 1.12 (0.64, 1.96) | 0.01 (–0.68, 0.68) | 0.01 (–0.63, 0.63) | 0.99 (0.00, 6468.95) |
| Marital status | 1.28 (0.80, 2.04) | 0.90 (–0.07, 1.87) | **0.30 (0.04, 0.57)** | 1.84 (0.95, 3.59) |
| Educational attainment (primary school ) | 0.92 (0.55, 1.56) | –0.18 (–0.88, 0.52) | –0.14 (–0.70, 0.43) | 0.65 (0.11, 3.79) |
| Educational attainment (high school and above) | 0.96 (0.45, 2.03) | –0.33 (–1.03, 0.36) | –0.42 (–1.47, 0.63) | NA^a^ |
| Occupation | 1.13 (0.43, 2.99) | 0.57 (–0.70, 1.84) | 0.22 (–0.29, 0.72) | 1.54 (0.41, 5.78) |
| Annual income (10000–29999 renminbi) | 1.19 (0.66, 2.18) | –0.02 (–0.71, 0.68) | –0.01 (–0.63, 0.61) | 0.89 (0.01, 128.38) |
| Annual income ( ≥ 30000 renminbi) | 1.01 (0.58, 1.75) | –0.19 (–0.81, 0.43) | –0.20 (–0.88, 0.49) | NA^a^ |
| Cerebrovascular disease | 1.45 (0.51, 4.14) | 0.82 (–1.47, 3.11) | 0.33 (–0.32, 0.98) | 2.26 (0.37, 13.96) |
| Rheumatism | 1.16 (0.52, 2.61) | 0.27 (–1.09, 1.62) | 0.15 (–0.50, 0.79) | 1.47 (0.25, 8.56) |
| Osteoarthropathy | 1.47 (0.70, 3.10) | 0.65 (–0.76, 2.07) | 0.31 (–0.18, 0.80) | 2.41 (0.49, 11.81) |
| Smoking status | 1.10 (0.52, 2.31) | 0.01 (–0.93, 0.94) | 0.01 (–0.69, 0.70) | 1.02 (0.06, 17.42) |
| Alcohol consumption | **0.44 (0.19, 0.98)** | **–0.93 (–1.57, –0.28)** | –1.60 (–3.64, 0.43) | NA^a^ |
| Low handgrip strength | 1.15 (0.66, 2.01) | 1.08 (–0.05, 2.21) | **0.28 (0.03, 0.54)** | 1.63 (0.94, 2.81) |
| Anemia | 1.45 (0.91, 2.31) | **1.14 (0.12, 2.15)** | **0.37 (0.13, 0.62)** | **2.26 (1.08, 4.73)** |
| Low density lipoprotein cholesterol | 0.89 (0.55, 1.45) | –0.21 (–0.90, 0.48) | –0.15 (–0.68, 0.37) | 0.65 (0.16, 2.68) |
| Total cholesterol | 1.07 (0.66, 1.72) | 0.03 (–0.63, 0.68) | 0.02 (–0.44, 0.47) | 1.07 (0.21, 5.36) |
| Serum creatinine | 0.95 (0.53, 1.68) | –0.01 (–0.93, 0.91) | 0.00 (–0.53, 0.52) | 0.99 (0.29, 3.38) |
| Uric acid | 0.94 (0.56, 1.56) | 0.04 (–0.79, 0.87) | 0.02 (–0.41, 0.46) | 1.05 (0.41, 2.71) |
| Aspartate aminotransferase | 1.36 (0.78, 2.35) | 0.35 (–0.42, 1.12) | 0.21 (–0.19, 0.62) | 2.20 (0.34, 14.22) |
| Alanine aminotransferase | 1.16 (0.46, 2.91) | 0.07 (–1.00, 1.14) | 0.05 (–0.68, 0.78) | 1.20 (0.08, 18.23) |

**Notes:** RERI=0, AP=0, and SI=1 indicate the absence of interactive effects between main explanatory variables and covariates. Conversely, when RERI>0, AP>0, and SI>1, this signifies that the combined effects between main explanatory variables and covariates exceed the sum of their individual effects, suggesting synergistic effects. Conversely, if RERI<0, AP<0, and SI<1, it indicates that the combined effects are smaller than the sum of the individual effects of main explanatory variables and covariates. ^a^, NA indicated the SI of part of covariates in addictive interaction analysis were null because of meaningless statistical value. Interaction analyses were adjusted for sex, age, ethnic, marital status, educational attainment, occupation, annual income, cerebrovascular disease, rheumatism, osteoarthropathy, smoking status, alcohol consumption, hand grip strength, anemia, total cholesterol, low density lipoprotein cholesterol, aspartate aminotransferase, alanine aminotransferase, serum creatinine, and uric acid.

**Abbreviations:** MetS, metabolic syndrome; ADL, activities of daily living; PR, prevalence ratio; CI, confidence interval; RERI, relative excess risk due to interaction; AP, attribuSupplementary Table proportion due to interaction; SI, synergy index.

**Supplementary Table S15.** Interaction effects of covariates and number of MetS components on ADL disability

| Variables | Number of  MetS component | Multiplicative interaction [PR (95%CI)] | Additive interaction [PR (95%CI)] | | |
| --- | --- | --- | --- | --- | --- |
|  |  |  | RERI | AP | S |
| Gender | 1 | 0.91 (0.47, 1.77) | -0.11 (-0.87, 0.64) | -0.09 (-0.68, 0.50) | 0.68 (0.10, 4.58) |
|  | 2 | 0.86 (0.42, 1.75) | -0.44 (-1.41, 0.52) | -0.30 (-0.90, 0.30) | 0.52 (0.19, 1.42) |
|  | 3 | 0.80 (0.39, 1.66) | 0.51 (-0.57, 1.59) | 0.21 (-0.24, 0.66) | 1.57 (0.45, 5.48) |
|  | ≥ 4 | 1.18 (0.41, 3.39) | 0.35 (-1.35, 2.05) | 0.16 (-0.62, 0.94) | 1.44 (0.18, 11.77) |
| Age group | 1 | 1.54 (0.78, 3.06) | 0.81 (-0.15, 1.76) | 0.30 (-0.06, 0.66) | 1.89 (0.59, 6.04) |
|  | 2 | 1.58 (0.76, 3.25) | **1.41 (0.28, 2.54)** | **0.39 (0.10, 0.69)** | 2.20 (0.84, 5.75) |
|  | 3 | 2.10 (0.98, 4.51) | 1.55 (-0.02, 3.13) | 0.35 (0.04, 0.65) | 1.81 (0.88, 3.73) |
|  | ≥ 4 | 1.77 (0.59, 5.35) | 2.14 (-0.15, 4.44) | 0.49 (0.11, 0.86) | 2.69 (0.75, 9.64) |
| Ethnic | 1 | 1.88 (0.83, 4.27) | 0.46 (-0.05, 0.96) | 0.64 (-0.33, 1.61) | **0.38 (0.17, 0.87)** |
|  | 2 | 2.09 (0.91, 4.83) | 0.03 (-0.77, 0.83) | 0.03 (-0.90, 0.97) | 0.83 (0.01, 75.44) |
|  | 3 | 1.60 (0.70, 3.66) | -0.27 (-1.36, 0.83) | -0.24 (-1.16, 0.68) | 0.29 (0.004, 17.10) |
|  | ≥ 4 | 2.72 (0.79, 9.28) | 0.81 (-0.15, 1.77) | 0.70 (-0.17, 1.56) | NA^a^ |
| Marital status | 1 | 0.87 (0.45, 1.67) | -0.06 (-1.17, 1.04) | -0.03 (-0.54, 0.49) | 0.95 (0.38, 2.37) |
|  | 2 | 0.94 (0.46, 1.90) | -0.16 (-1.39, 1.07) | -0.06 (-0.56, 0.43) | 0.90 (0.43, 1.90) |
|  | 3 | 0.90 (0.43, 1.85) | 0.27 (-1.36, 1.89) | 0.08 (-0.37, 0.52) | 1.12 (0.56, 2.23) |
|  | ≥ 4 | 2.02 (0.73, 5.60) | **2.41 (0.02, 4.80)** | **0.54 (0.19, 0.88)** | 3.24 (0.82, 12.77) |
| Educational attainment (primary school ) | 1 | 1.10 (0.53, 2.29) | 0.10 (-0.66, 0.87) | 0.08 (-0.49, 0.64) | 1.38 (0.07, 28.07) |
|  | 2 | 1.30 (0.60, 2.85) | -0.21 (-1.17, 0.75) | -0.13 (-0.73, 0.46) | 0.74 (0.22, 2.46) |
|  | 3 | 1.13 (0.51, 2.51) | -0.02 (-1.33, 1.29) | -0.01 (-0.55, 0.54) | 0.99 (0.39, 2.49) |
|  | ≥ 4 | 0.64 (0.20, 2.06) | -0.84 (-2.69, 1.00) | -0.62 (-2.29, 1.04) | 0.30 (0.01, 8.71) |
| Educational attainment (high school and above) | 1 | 0.61 (0.25, 1.48) | -0.53 (-1.55, 0.49) | -0.63 (-1.85, 0.59) | NA^a^ |
|  | 2 | 0.80 (0.30, 2.15) | -0.82 (-2.02, 0.38) | -0.75 (-1.92, 0.42) | 0.10 (0.01, 11.42) |
|  | 3 | 0.51 (0.17, 1.49) | -1.35 (-2.93, 0.23) | -1.15 (-2.84, 0.54) | 0.11 (0.01, 16.04) |
|  | ≥ 4 | 0.94 (0.28, 3.15) | -0.08 (-2.36, 2.20) | -0.04 (-1.08, 1.01) | 0.94 (0.15, 5.77) |
| Occupation | 1 | 3.54 (0.71, 17.56) | **0.95 (0.40, 1.51)** | 0.76 (-0.20, 1.72) | NA^a^ |
|  | 2 | 2.51 (0.42, 15.12) | 0.41 (-0.69, 1.50) | 0.26 (-0.63, 1.14) | 3.39 (0.01, 5.76) |
|  | 3 | 1.05 (0.22, 4.99) | 0.94 (-0.23, 2.11) | 0.43 (-0.28, 1.14) | 4.74 (0.01, 33.76) |
|  | ≥ 4 | 2.86 (0.30, 27.34) | 1.32 (-0.39, 3.03) | 0.66 (-0.27, 1.59) | NA^a^ |
| Annual income (10000-29999 renminbi) | 1 | 0.63 (0.28, 1.43) | -0.50 (-1.42, 0.43) | -0.63 (-1.75, 0.49) | NA^a^ |
|  | 2 | 0.51 (0.22, 1.19) | -0.61 (-1.70, 0.48) | -0.46 (-1.28, 0.35) | 0.34 (0.06, 1.96) |
|  | 3 | 0.59 (0.26, 1.38) | -0.88 (-2.35, 0.58) | -0.58 (-1.63, 0.48) | 0.37 (0.07, 1.93) |
|  | ≥ 4 | 1.00 (0.33, 3.05) | -0.10 (-2.06, 1.85) | -0.05 (-1.05, 0.94) | 0.91 (0.14, 5.70) |
| Annual income ( ≥ 30000 renminbi) | 1 | 1.004 (0.47, 2.16) | -0.08 (-0.84, 0.68) | -0.07 (-0.78, 0.63) | 0.45 (0.01, 17.10) |
|  | 2 | 1.21 (0.51, 2.84) | -0.80 (-1.87, 0.26) | -0.81 (-1.83, 0.21) | NA^a^ |
|  | 3 | 0.80 (0.33, 1.95) | -0.52 (-1.80, 0.76) | -0.30 (-1.03, 0.44) | 0.59 (0.20, 1.79) |
|  | ≥ 4 | 0.68 (0.20, 2.26) | -0.82 (-2.60, 0.96) | -0.71 (-2.57, 1.14) | 0.15 (0.01, 1.62) |
| Cerebrovascular disease | 1 | 2.67 (0.34, 20.87) | 0.85 (-0.49, 2.20) | 0.55 (-0.14, 1.25) | NA^a^ |
|  | 2 | 2.18 (0.27, 17.33) | 0.67 (-1.07, 2.40) | 0.38 (-0.39, 1.16) | 11.09 (0.01, 22.56) |
|  | 3 | 2.22 (0.26, 18.74) | 0.45 (-2.44, 3.35) | 0.22 (-0.92, 1.36) | 1.73 (0.08, 36.81) |
|  | ≥ 4 | 5.44 (0.54, 54.89) | 3.44 (-2.30, 9.17) | **0.72 (0.31, 1.14)** | 11.88 (0.15, 94.03) |
| Rheumatism | 1 | 1.20 (0.39, 3.65) | 0.12 (-0.83, 1.07) | 0.11 (-0.72, 0.94) | NA |
|  | 2 | 1.10 (0.36, 3.39) | 0.54 (-0.72, 1.81) | 0.29 (-0.29, 0.88) | 2.72 (0.12, 61.59) |
|  | 3 | 1.38 (0.43, 4.46) | 1.17 (-1.18, 3.52) | 0.39 (-0.15, 0.92) | 2.38 (0.49, 11.66) |
|  | ≥ 4 | 1.06 (0.19, 5.82) | -0.15 (-2.52, 2.21) | -0.10 (-1.71, 1.51) | 0.79 (0.02, 38.97) |
| Osteoarthropathy | 1 | 1.04 (0.44, 2.45) | 0.02 (-0.83, 0.88) | 0.02 (-0.71, 0.75) | 1.16 (0.01, 4.56) |
|  | 2 | 1.02 (0.41, 2.52) | 0.27 (-0.72, 1.27) | 0.16 (-0.38, 0.70) | 1.60 (0.23, 11.18) |
|  | 3 | 1.13 (0.46, 2.78) | 1.01 (-1.13, 3.15) | 0.34 (-0.18, 0.86) | 2.03 (0.53, 7.86) |
|  | ≥ 4 | 0.67 (0.09, 5.20) | -0.71 (-3.21, 1.80) | -0.59 (-3.72, 2.54) | 0.21 (0.01, 3.35) |
| Smoking status | 1 | 1.19 (0.46, 3.12) | 0.11 (-0.70, 0.91) | 0.10 (-0.65, 0.85) | NA^a^ |
|  | 2 | 1.27 (0.45, 3.57) | -0.05 (-1.01, 0.91) | -0.04 (-0.81, 0.72) | 0.84 (0.04, 19.22) |
|  | 3 | 1.09 (0.37, 3.24) | 0.68 (-0.84, 2.21) | 0.28 (-0.23, 0.79) | 1.90 (0.44, 8.25) |
|  | ≥ 4 | NA^b^ | NA^b^ | NA^b^ | NA^a^ |
| Alcohol consumption | 1 | 1.19 (0.47, 3.05) | 0.06 (-0.64, 0.76) | 0.06 (-0.69, 0.81) | 0.53 (0.01, 5.46) |
|  | 2 | 1.23 (0.44, 3.39) | 0.15 (-0.66, 0.97) | 0.11 (-0.48, 0.71) | 1.81 (0.03, 11.58) |
|  | 3 | 1.39 (0.50, 3.90) | -0.74 (-1.90, 0.42) | -0.63 (-1.85, 0.59) | 0.20 (0.002, 14.20) |
|  | ≥ 4 | 0.91 (0.22, 3.81) | -0.51 (-2.15, 1.14) | -0.44 (-2.23, 1.36) | 0.23 (0.01, 10.58) |
| Low handgrip strength | 1 | 1.83 (0.89, 3.76) | **0.85 (0.16, 1.54)** | **0.41 (0.03, 0.80)** | 5.32 (0.03, 10.82) |
|  | 2 | 1.88 (0.87, 4.07) | **1.002 (0.15, 1.85)** | **0.38 (0.05, 0.72)** | 2.63 (0.48, 14.27) |
|  | 3 | 1.78 (0.81, 3.90) | **1.86 (0.54, 3.18)** | **0.50 (0.22, 0.78)** | 3.15 (0.80, 12.37) |
|  | ≥ 4 | 1.64 (0.58, 4.63) | 1.49 (-0.46, 3.45) | 0.44 (-0.01, 0.90) | 2.73 (0.47, 15.71) |
| Anemia | 1 | 0.65 (0.34, 1.28) | -0.50 (-1.64, 0.64) | -0.25 (-0.79, 0.29) | 0.67 (0.33, 1.38) |
|  | 2 | 0.48 (0.23, 1.01) | 0.25 (-0.96, 1.45) | 0.08 (-0.31, 0.47) | 1.14 (0.59, 2.19) |
|  | 3 | 0.71 (0.33, 1.50) | 1.50 (-0.34, 3.34) | **0.31 (0.01, 0.63)** | 1.65 (0.86, 3.18) |
|  | ≥ 4 | 1.27 (0.49, 3.29) | 2.04 (-1.05, 5.13) | 0.41 (-0.02, 0.83) | 2.03 (0.74, 5.54) |
| Low density lipoprotein cholesterol | 1 | 1.32 (0.67, 2.63) | 0.29 (-0.34, 0.92) | 0.23 (-0.28, 0.73) | NA^a^ |
|  | 2 | 1.31 (0.63, 2.72) | -0.21 (-1.02, 0.60) | -0.15 (-0.74, 0.44) | 0.64 (0.15, 2.75) |
|  | 3 | 0.88 (0.41, 1.87) | -0.48 (-1.63, 0.66) | -0.27 (-0.94, 0.40) | 0.62 (0.22, 1.75) |
|  | ≥ 4 | 0.98 (0.34, 2.81) | -0.16 (-1.77, 1.44) | -0.10 (-1.14, 0.94) | 0.78 (0.08, 8.02) |
| Total cholesterol | 1 | 1.65 (0.85, 3.18) | 0.46 (-0.06, 0.97) | 0.39 (-0.09, 0.88) | NA^a^ |
|  | 2 | 1.91 (0.93, 3.89) | -0.22 (-0.97, 0.53) | -0.19 (-0.80, 0.43) | 0.46 (0.06, 3.67) |
|  | 3 | 1.13 (0.54, 2.33) | -0.08 (-1.08, 0.92) | -0.05 (-0.62, 0.53) | 0.90 (0.27, 3.03) |
|  | ≥ 4 | 1.47 (0.55, 3.87) | 0.36 (-1.03, 1.74) | 0.21 (-0.55, 0.97) | 1.96 (0.06, 59.77) |
| Serum creatinine | 1 | 1.01 (0.47, 2.15) | 0.05 (-0.86, 0.96) | 0.03 (-0.60, 0.66) | 1.12 (0.12, 10.64) |
|  | 2 | 0.87 (0.40, 1.90) | -0.21 (-1.30, 0.88) | -0.13 (-0.81, 0.56) | 0.75 (0.18, 3.09) |
|  | 3 | 0.69 (0.30, 1.59) | 0.22 (-1.24, 1.69) | 0.09 (-0.46, 0.64) | 1.17 (0.42, 3.27) |
|  | ≥ 4 | 0.84 (0.25, 2.82) | -0.19 (-2.42, 2.04) | -0.09 (-1.25, 1.06) | 0.85 (0.11, 6.28) |
| Uric acid | 1 | 0.68 (0.24, 1.87) | -0.46 (-1.74, 0.83) | -0.41 (-1.60, 0.79) | 0.22 (0.01, 23.06) |
|  | 2 | 0.54 (0.19, 1.52) | 0.62 (-0.72, 1.96) | 0.26 (-0.28, 0.80) | 1.83 (0.34, 9.98) |
|  | 3 | 1.15 (0.43, 3.09) | 0.06 (-1.50, 1.61) | 0.02 (-0.60, 0.65) | 1.04 (0.35, 3.08) |
|  | ≥ 4 | 1.03 (0.32, 3.37) | 0.30 (-1.69, 2.29) | 0.12 (-0.66, 0.91) | 1.27 (0.24, 6.76) |
| Aspartate aminotransferase | 1 | **3.04 (1.03, 9.00)** | **0.74 (0.16, 1.32)** | **0.63 (0.19, 1.07)** | NA^a^ |
|  | 2 | 2.70 (0.90, 8.07) | 0.30 (-0.40, 1.00) | 0.26 (-0.30, 0.83) | NA^a^ |
|  | 3 | 1.57 (0.50, 4.96) | 0.83 (-0.21, 1.87) | **0.41 (0.01, 0.81)** | 4.87 (0.11, 20.87) |
|  | ≥ 4 | 3.29 (0.87, 12.48) | 0.94 (-0.73, 2.62) | 0.48 (-0.11, 1.07) | 80.38 (0.01, 327.34) |
| Alanine transaminas | 1 | 2.49 (0.31, 20.32) | 0.88 (-0.75, 2.51) | 0.54 (-0.22, 1.29) | NA^a^ |
|  | 2 | 2.31 (0.28, 18.87) | -0.20 (-1.66, 1.26) | -0.20 (-1.82, 1.41) | NA^a^ |
|  | 3 | 0.53 (0.05, 5.74) | -0.02 (-1.72, 1.68) | -0.01 (-0.99, 0.97) | 0.98 (0.10, 9.53) |
|  | ≥ 4 | 2.33 (0.25, 22.13) | 0.94 (-1.87, 3.74) | 0.40 (-0.45, 1.24) | 3.15 (0.08, 120.77) |

**Notes:** RERI=0, AP=0, and SI=1 indicate the absence of interactive effects between main explanatory variables and covariates. Conversely, when RERI>0, AP>0, and SI>1, this signifies that the combined effects between main explanatory variables and covariates exceed the sum of their individual effects, suggesting synergistic effects. Conversely, if RERI<0, AP<0, and SI<1, it indicates that the combined effects are smaller than the sum of the individual effects of main explanatory variables and covariates. ^a^, NA indicated the SI of part of covariates in addictive interaction analysis were null because of meaningless statistical value; ^b^, NA indicated the multiplicative interaction and RERI and AP in addictive interaction analysis were null because meaningless statistical value cannot calculate 95%CI.Interaction analyses were adjusted for sex, age, ethnic, marital status, educational attainment, occupation, annual income, cerebrovascular disease, rheumatism, osteoarthropathy, smoking status, alcohol consumption, hand grip strength, anemia, total cholesterol, low density lipoprotein cholesterol, aspartate aminotransferase, alanine aminotransferase, serum creatinine, and uric acid.

**Abbreviations:** MetS, metabolic syndrome; ADL, activities of daily living; PR, prevalence ratio; CI, confidence interval; RERI, relative excess risk due to interaction; AP, attribuSupplementary Table proportion due to interaction; SI, synergy index.

**Supplementary Table S16.** Interaction effects of covariates and number of MetS components on IADL disability

| Variables | Number of  MetS component | Multiplicative interaction [PR (95%CI)] | Additive interaction [PR (95%CI)] | | |
| --- | --- | --- | --- | --- | --- |
|  |  |  | RERI | AP | SI |
| Gender | 1 | 0.95 (0.68, 1.31) | 0.10 (–0.33, 0.53) | 0.04 (–0.14, 0.22) | 1.08 (0.76, 1.52) |
|  | 2 | 0.97 (0.69, 1.36) | 0.21 (–0.25, 0.67) | 0.08 (–0.10, 0.27) | 1.15 (0.81, 1.66) |
|  | 3 | 0.91 (0.60, 1.39) | 0.01 (–0.57, 0.60) | 0.01 (–0.25, 0.26) | 1.01 (0.64, 1.59) |
|  | ≥ 4 | 1.17 (0.58, 2.35) | 0.25 (–0.56, 1.06) | 0.11 (–0.24, 0.46) | 1.25 (0.57, 2.74) |
| Age group | 1 | 1.21 (0.92, 1.59) | 0.30 (–0.03, 0.62) | 0.16 (–0.02, 0.35) | 1.57 (0.78, 3.18) |
|  | 2 | 1.32 (0.99, 1.76) | **0.50 (0.15, 0.85)** | **0.24 (0.07, 0.42)** | 1.89 (0.94, 3.81) |
|  | 3 | **1.85 (1.28, 2.67)** | **0.87 (0.45, 1.28)** | **0.40 (0.22, 0.58)** | 3.81 (0.94, 15.44) |
|  | ≥ 4 | 1.52 (0.90, 2.58) | 0.56 (–0.05, 1.17) | **0.29 (0.01, 0.57)** | 2.53 (0.60, 10.66) |
| Ethnic | 1 | 1.18 (0.83, 1.68) | 0.13 (–0.18, 0.45) | 0.15 (–0.24, 0.55) | 0.50 (0.21, 1.21) |
|  | 2 | 1.23 (0.85, 1.79) | 0.17 (–0.16, 0.50) | 0.18 (–0.20, 0.56) | 0.25 (0.01, 5.04) |
|  | 3 | 1.07 (0.71, 1.61) | 0.03 (–0.38, 0.43) | 0.03 (–0.44, 0.51) | 0.82 (0.08, 8.63) |
|  | ≥ 4 | 1.33 (0.74, 2.39) | 0.25 (–0.24, 0.74) | 0.29 (–0.29, 0.87) | 0.37 (0.06, 2.29) |
| Marital status | 1 | 1.04 (0.79, 1.37) | 0.12 (–0.25, 0.48) | 0.07 (–0.14, 0.28) | 1.20 (0.64, 2.23) |
|  | 2 | 1.11 (0.84, 1.48) | 0.26 (–0.13, 0.64) | 0.14 (–0.07, 0.34) | 1.41 (0.76, 2.59) |
|  | 3 | 1.07 (0.77, 1.50) | 0.18 (–0.29, 0.66) | 0.10 (–0.15, 0.36) | 1.29 (0.63, 2.67) |
|  | ≥ 4 | 1.33 (0.82, 2.15) | 0.42 (–0.22, 1.06) | 0.23 (–0.08, 0.54) | 2.00 (0.58, 6.92) |
| Educational attainment (primary school ) | 1 | 1.02 (0.76, 1.37) | –0.03 (–0.26, 0.20) | –0.04 (–0.37, 0.29) | 1.10 (0.47, 2.54) |
|  | 2 | 0.97 (0.71, 1.34) | –0.10 (–0.36, 0.15) | –0.14 (–0.49, 0.21) | 1.63 (0.33, 8.02) |
|  | 3 | 1.10 (0.76, 1.60) | 0.02 (–0.28, 0.32) | 0.03 (–0.37, 0.42) | 0.92 (0.27, 3.10) |
|  | ≥ 4 | 0.91 (0.51, 1.63) | –0.08 (–0.50, 0.34) | –0.13 (–0.88, 0.62) | 1.24 (0.37, 4.12) |
| Educational attainment  (high school and above) | 1 | 1.36 (0.75, 2.44) | 0.01 (–0.21, 0.22) | 0.02 (–0.61, 0.65) | 0.99 (0.72, 1.37) |
|  | 2 | 1.19 (0.63, 2.22) | –0.12 (–0.36, 0.13) | –0.36 (–1.14, 0.42) | 1.21 (0.79, 1.87) |
|  | 3 | 1.07 (0.50, 2.28) | –0.08 (–0.36, 0.20) | –0.30 (–1.40, 0.81) | 1.13 (0.73, 1.73) |
|  | ≥ 4 | 2.02 (0.84, 4.88) | 0.20 (–0.24, 0.64) | 0.42 (–0.33, 1.17) | 0.72 (0.34, 1.51) |
| Occupation | 1 | 0.63 (0.19, 2.12) | –0.15 (–1.82, 1.52) | –0.03 (–0.33, 0.27) | 0.97 (0.68, 1.37) |
|  | 2 | 0.47 (0.15, 1.54) | –0.51 (–2.61, 1.59) | –0.09 (–0.39, 0.21) | 0.90 (0.66, 1.24) |
|  | 3 | 0.70 (0.19, 2.53) | 0.33 (–1.30, 1.96) | 0.06 (–0.26, 0.38) | 1.08 (0.70, 1.65) |
|  | ≥ 4 | 1.55 (0.17, 14.22) | 1.06 (–0.85, 2.97) | 0.20 (–0.19, 0.59) | 1.32 (0.67, 2.59) |
| Annual income (10000–29999 renminbi) | 1 | 1.05 (0.77, 1.44) | 0.04 (–0.23, 0.30) | 0.04 (–0.29, 0.38) | 0.86 (0.29, 2.52) |
|  | 2 | 1.01 (0.73, 1.41) | –0.03 (–0.32, 0.27) | –0.03 (–0.36, 0.31) | 1.28 (0.05, 32.33) |
|  | 3 | 0.88 (0.58, 1.33) | –0.14 (–0.51, 0.22) | –0.19 (–0.70, 0.32) | 2.49 (0.06, 99.25) |
|  | ≥ 4 | 0.84 (0.49, 1.45) | –0.22 (–0.74, 0.29) | –0.27 (–0.96, 0.42) | NA^a^ |
| Annual income ( ≥ 30000 renminbi) | 1 | **1.50 (1.06, 2.12)** | **0.23 (0.01, 0.44)** | **0.33 (0.01, 0.66)** | **0.58 (0.38, 0.89)** |
|  | 2 | 1.28 (0.89, 1.85) | 0.07 (–0.19, 0.32) | 0.10 (–0.28, 0.47) | 0.83 (0.44, 1.59) |
|  | 3 | 1.30 (0.86, 1.97) | 0.07 (–0.23, 0.38) | 0.11 (–0.34, 0.56) | 0.81 (0.36, 1.81) |
|  | ≥ 4 | 0.56 (0.26, 1.19) | –0.41 (–0.86, 0.03) | –1.26 (–3.09, 0.57) | 2.61 (0.52, 13.19) |
| Cerebrovascular disease | 1 | 0.72 (0.37, 1.37) | –0.34 (–1.00, 0.32) | –0.39 (–1.21, 0.43) | NA^a^ |
|  | 2 | 1.0 (0.51, 1.94) | 0.01 (–0.75, 0.78) | 0.01 (–0.57, 0.59) | 1.04 (0.09, 12.69) |
|  | 3 | 0.73 (0.27, 2.02) | –0.32 (–1.31, 0.66) | –0.35 (–1.66, 0.96) | NA^a^ |
|  | ≥ 4 | 1.83 (0.77, 4.35) | 0.97 (–0.52, 2.45) | 0.46 (0.00, 0.91) | 7.32 (0.09, 58.69) |
| Rheumatism | 1 | 0.83 (0.55, 1.25) | –0.21 (–0.65, 0.24) | –0.20 (–0.65, 0.24) | 0.10 (0.01, 20.46) |
|  | 2 | 1.19 (0.77, 1.82) | 0.25 (–0.26, 0.77) | 0.17 (–0.15, 0.48) | 1.91 (0.38, 9.57) |
|  | 3 | 0.80 (0.41, 1.56) | –0.25 (–0.95, 0.45) | –0.24 (–1.04, 0.55) | 0.06 (0.01, 20.99) |
|  | ≥ 4 | 0.88 (0.40, 1.95) | –0.14 (–0.99, 0.71) | –0.13 (–1.00, 0.74) | 0.31 (0.01, 3.62) |
| Osteoarthropathy | 1 | 1.06 (0.72, 1.54) | 0.02 (–0.30, 0.35) | 0.02 (–0.33, 0.38) | 0.80 (0.03, 19.86) |
|  | 2 | 1.30 (0.89, 1.90) | 0.24 (–0.12, 0.59) | 0.20 (–0.08, 0.48) | NA^a^ |
|  | 3 | 1.35 (0.78, 2.36) | 0.28 (–0.31, 0.87) | 0.24 (–0.18, 0.66) | NA^a^ |
|  | ≥ 4 | 1.20 (0.48, 3.00) | 0.15 (–0.75, 1.05) | 0.15 (–0.63, 0.93) | NA^a^ |
| Smoking status | 1 | 0.74 (0.46, 1.19) | –0.25 (–0.56, 0.07) | –0.47 (–1.10, 0.17) | 2.15 (0.52, 8.86) |
|  | 2 | 0.80 (0.49, 1.33) | –0.25 (–0.60, 0.10) | –0.41 (–1.03, 0.22) | 2.96 (0.24, 36.47) |
|  | 3 | 1.22 (0.69, 2.15) | 0.10 (–0.35, 0.54) | 0.12 (–0.38, 0.61) | 0.61 (0.05, 7.46) |
|  | ≥ 4 | 0.26 (0.04, 1.83) | –0.57 (–1.09, –0.06) | –3.16 (–11.33, 5.00) | 3.34 (0.66, 16.98) |
| Alcohol consumption | 1 | 0.87 (0.57, 1.32) | –0.18 (–0.48, 0.12) | –0.28 (–0.75, 0.19) | 2.02 (0.37, 11.02) |
|  | 2 | 0.92 (0.60, 1.43) | –0.17 (–0.50, 0.15) | –0.23 (–0.69, 0.22) | 2.81 (0.10, 78.63) |
|  | 3 | 0.74 (0.43, 1.29) | –0.30 (–0.69, 0.08) | –0.53 (–1.32, 0.27) | 3.43 (0.22, 53.86) |
|  | ≥ 4 | 0.57 (0.23, 1.43) | –0.40 (–0.90, 0.11) | –0.94 (–2.75, 0.86) | 3.14 (0.35, 28.36) |
| Low handgrip strength | 1 | 1.004 (0.75, 1.35) | 0.08 (–0.28, 0.44) | 0.05 (–0.16, 0.25) | 1.11 (0.67, 1.86) |
|  | 2 | 1.02 (0.75, 1.40) | 0.17 (–0.21, 0.56) | 0.09 (–0.11, 0.29) | 1.21 (0.74, 1.99) |
|  | 3 | 1.46 (1.0, 2.14) | **0.64 (0.20, 1.09)** | **0.29 (0.10, 0.49)** | 2.19 (0.91, 5.30) |
|  | ≥ 4 | 0.84 (0.51, 1.38) | –0.16 (–0.89, 0.56) | –0.09 (–0.52, 0.33) | 0.82 (0.35, 1.93) |
| Anemia | 1 | 1.06 (0.81, 1.39) | 0.14 (–0.18, 0.46) | 0.09 (–0.11, 0.29) | 1.30 (0.63, 2.67) |
|  | 2 | 0.92 (0.69, 1.22) | –0.04 (–0.41, 0.33) | –0.03 (–0.25, 0.20) | 0.94 (0.53, 1.64) |
|  | 3 | 1.15 (0.82, 1.61) | 0.30 (–0.16, 0.75) | 0.16 (–0.07, 0.40) | 1.59 (0.69, 3.65) |
|  | ≥ 4 | 1.45 (0.91, 2.32) | 0.65 (–0.05, 1.36) | 0.32 (0.04, 0.60) | 2.70 (0.72, 10.05) |
| Low density lipoprotein cholesterol | 1 | 1.24 (0.93, 1.66) | 0.19 (–0.06, 0.44) | 0.18 (–0.07, 0.43) | NA |
|  | 2 | 1.03 (0.76, 1.39) | –0.03 (–0.31, 0.26) | –0.02 (–0.30, 0.26) | 0.57 (0.01, 146.47) |
|  | 3 | 1.10 (0.77, 1.57) | 0.06 (–0.29, 0.40) | 0.06 (–0.28, 0.39) | NA^a^ |
|  | ≥ 4 | **1.70 (1.01, 2.85)** | **0.51 (0.01, 1.01)** | **0.43 (0.07, 0.79)** | NA^a^ |
| Total cholesterol | 1 | 1.14 (0.87, 1.50) | 0.13 (–0.12, 0.38) | 0.12 (–0.12, 0.36) | NA^a^ |
|  | 2 | 1.03 (0.77, 1.36) | 0.01 (–0.29, 0.29) | 0.01 (–0.26, 0.26) | 0.99 (0.09, 10.64) |
|  | 3 | 0.96 (0.68, 1.34) | –0.07 (–0.44, 0.29) | –0.07 (–0.42, 0.28) | 0.35 (0.01, 49.40) |
|  | ≥ 4 | 1.51 (0.91, 2.52) | 0.41 (–0.07, 0.90) | 0.35 (–0.02, 0.73) | NA^a^ |
| Serum creatinine | 1 | 0.87 (0.64, 1.17) | –0.15 (–0.54, 0.24) | –0.11 (–0.40, 0.18) | 0.71 (0.32, 1.58) |
|  | 2 | 0.78 (0.57, 1.09) | –0.27 (–0.71, 0.16) | –0.20 (–0.53, 0.13) | 0.57 (0.25, 1.33) |
|  | 3 | 0.98 (0.68, 1.43) | 0.04 (–0.47, 0.55) | 0.02 (–0.30, 0.35) | 1.07 (0.41, 2.83) |
|  | ≥ 4 | 0.80 (0.44, 1.45) | –0.26 (–1.02, 0.50) | –0.20 (–0.87, 0.47) | 0.52 (0.05, 5.45) |
| Uric acid | 1 | 1.47 (0.83, 2.58) | 0.28 (–0.12, 0.67) | 0.27 (–0.10, 0.64) | NA^a^ |
|  | 2 | 1.53 (0.87, 2.69) | 0.32 (–0.09, 0.72) | 0.28 (–0.06, 0.61) | NA^a^ |
|  | 3 | **1.81 (1.003, 3.25)** | **0.51 (0.06, 0.96)** | **0.42 (0.08, 0.75)** | NA^a^ |
|  | ≥ 4 | 1.95 (0.99, 3.84) | **0.59 (0.01, 1.17)** | **0.48 (0.09, 0.88)** | NA^a^ |
| Aspartate aminotransferase | 1 | 0.86 (0.62, 1.19) | –0.16 (–0.53, 0.20) | –0.15 (–0.48, 0.19) | 0.37 (0.04, 3.14) |
|  | 2 | 0.77 (0.55, 1.10) | –0.29 (–0.69, 0.10) | –0.27 (–0.65, 0.11) | 0.25 (0.02, 2.79) |
|  | 3 | 0.85 (0.57, 1.27) | –0.18 (–0.63, 0.27) | –0.16 (–0.58, 0.26) | 0.42 (0.04, 4.72) |
|  | ≥ 4 | 1.13 (0.66, 1.93) | 0.16 (–0.50, 0.81) | 0.12 (–0.34, 0.58) | 1.89 (0.11, 32.84) |
| Alanine aminotransferase | 1 | 0.72 (0.33, 1.56) | –0.32 (–1.02, 0.39) | –0.40 (–1.41, 0.60) | NA^a^ |
|  | 2 | 0.54 (0.24, 1.19) | –0.58 (–1.27, 0.11) | –0.89 (–2.22, 0.44) | NA^a^ |
|  | 3 | 0.91 (0.44, 1.90) | –0.11 (–0.83, 0.61) | –0.10 (–0.82, 0.61) | 0.29 (0.01, 47.93) |
|  | ≥ 4 | 1.35 (0.58, 3.15) | 0.36 (–0.63, 1.34) | 0.25 (–0.35, 0.86) | 8.68 (0.01, 13.93) |

**Notes:** RERI=0, AP=0, and SI=1 indicate the absence of interactive effects between main explanatory variables and covariates. Conversely, when RERI>0, AP>0, and SI>1, this signifies that the combined effects between main explanatory variables and covariates exceed the sum of their individual effects, suggesting synergistic effects. Conversely, if RERI<0, AP<0, and SI<1, it indicates that the combined effects are smaller than the sum of the individual effects of main explanatory variables and covariates. ^a^, NA indicated the SI of part of covariates in addictive interaction analysis were null because of meaningless statistical value.Interaction analyses were adjusted for sex, age, ethnic, marital status, educational attainment, occupation, annual income, cerebrovascular disease, rheumatism, osteoarthropathy, smoking status, alcohol consumption, hand grip strength, anemia, total cholesterol, low density lipoprotein cholesterol, aspartate aminotransferase, alanine aminotransferase, serum creatinine, and uric acid.

**Abbeviations:** MetS, metabolic syndrome; IADL, instrumental activities of daily living; PR, prevalence ratio; CI, confidence interval; RERI, relative excess risk due to interaction; AP, attribuSupplementary Table proportion due to interaction; SI, synergy index.

**Supplementary Table S17.** Interaction effects of covariates and number of MetS components on comorbid ADL­IADL disability

| Variables | Number of  MetS component | Multiplicative interaction [PR (95%CI)] | Additive interaction [PR (95%CI)] | | |
| --- | --- | --- | --- | --- | --- |
|  |  |  | RERI | AP | SI |
| Gender | 1 | 0.86 (0.42, 1.75) | –0.18 (–1.05, 0.69) | –0.13 (–0.72, 0.46) | 0.69 (0.18, 2.60) |
|  | 2 | 1.58 (0.76, 3.25) | 0.96 (–0.04, 1.95) | 0.34 (–0.02, 0.70) | 2.12 (0.57, 7.89) |
|  | 3 | 2.09 (0.91, 4.83) | **0.49 (0.01, 0.98)** | 0.74 (–0.30, 1.78) | **0.41 (0.21, 0.78)** |
|  | ≥ 4 | 0.94 (0.46, 1.90) | 0.13 (–1.01, 1.27) | 0.06 (–0.44, 0.56) | 1.11 (0.41, 3.03) |
| Age group | 1 | 1.30 (0.60, 2.85) | 0.22 (–0.48, 0.93) | 0.18 (–0.40, 0.75) | 6.57 (0.01, 64.01) |
|  | 2 | 0.80 (0.30, 2.15) | –0.27 (–1.15, 0.61) | –0.36 (–1.55, 0.83) | NA^a^ |
|  | 3 | 2.51 (0.42, 15.12) | **0.98 (0.16, 1.79)** | 0.55 (–0.33, 1.43) | NA^a^ |
|  | ≥ 4 | 0.51 (0.22, 1.19) | –0.73 (–1.81, 0.34) | –0.95 (–2.27, 0.36) | NA^a^ |
| Ethnic | 1 | 1.21 (0.51, 2.84) | 0.01 (–0.76, 0.78) | 0.01 (–0.69, 0.70) | 1.08 (0.01, 27.29) |
|  | 2 | 2.18 (0.27, 17.33) | 0.71 (–0.80, 2.23) | 0.45 (–0.35, 1.25) | NA^a^ |
|  | 3 | 1.10 (0.36, 3.39) | 0.08 (–1.03, 1.18) | 0.06 (–0.77, 0.89) | 1.33 (0.01, 128.13) |
|  | ≥ 4 | 1.02 (0.41, 2.52) | 0.00 (–0.95, 0.95) | 0.001 (–0.75, 0.75) | 0.99 (0.03, 33.22) |
| Marital status | 1 | 1.27 (0.45, 3.57) | 0.14 (–0.72, 1.00) | 0.12 (–0.61, 0.86) | 28.66 (0.01, 184.19) |
|  | 2 | 1.23 (0.44, 3.39) | 0.04 (–0.72, 0.80) | 0.04 (–0.72, 0.80) | 0.26 (0.01, 14.02) |
|  | 3 | 1.88 (0.87, 4.07) | **0.98 (0.23, 1.72)** | **0.44 (0.06, 0.82)** | 5.40 (0.03, 8.43) |
|  | ≥ 4 | 0.48 (0.23, 1.01) | –1.06 (–2.72, 0.60) | –0.41 (–0.99, 0.17) | 0.60 (0.34, 1.07) |
| Educational attainment (primary school ) | 1 | 1.31 (0.63, 2.72) | 0.29 (–0.41, 0.98) | 0.21 (–0.30, 0.72) | 3.96 (0.01, 24.21) |
|  | 2 | 1.91 (0.93, 3.89) | **0.55 (0.04, 1.06)** | 0.47 (–0.02, 0.96) | NA^a^ |
|  | 3 | 0.87 (0.40, 1.90) | –0.10 (–1.20, 1.00) | –0.06 (–0.71, 0.60) | 0.88 (0.21, 3.62) |
|  | ≥ 4 | 0.54 (0.19, 1.52) | –0.78 (–2.30, 0.73) | –0.67 (–2.07, 0.73) | 0.17 (0.01, 8.87) |
| Educational attainment (high school and above) | 1 | 2.70 (0.90, 8.07) | **0.74 (0.07, 1.40)** | **0.56 (0.10, 1.01)** | NA^a^ |
|  | 2 | 2.31 (0.28, 18.87) | 0.95 (–0.93, 2.83) | 0.50 (–0.26, 1.27) | NA^a^ |
|  | 3 | 0.80 (0.39, 1.66) | –0.33 (–1.37, 0.71) | –0.19 (–0.78, 0.39) | 0.68 (0.26, 1.74) |
|  | ≥ 4 | 2.10 (0.98, 4.51) | **1.92 (0.68, 3.16)** | **0.51 (0.23, 0.79)** | 3.22 (0.82, 12.67) |
| Occupation | 1 | 1.60 (0.70, 3.66) | 0.21 (–0.50, 0.91) | 0.26 (–0.73, 1.26) | 0.51 (0.12, 2.10) |
|  | 2 | 0.90 (0.43, 1.85) | 0.22 (–1.04, 1.47) | 0.08 (–0.38, 0.54) | 1.15 (0.49, 2.68) |
|  | 3 | 1.13 (0.51, 2.51) | –0.02 (–0.91, 0.86) | –0.01 (–0.60, 0.57) | 0.96 (0.18, 5.12) |
|  | ≥ 4 | 0.51 (0.17, 1.49) | –0.85 (–1.96, 0.26) | –1.30 (–3.21, 0.62) | NA^a^ |
| Annual income (10000–29999 renminbi) | 1 | 1.05 (0.22, 4.99) | 0.25 (–1.37, 1.86) | 0.12 (–0.75, 0.98) | 1.28 (0.12, 13.13) |
|  | 2 | 0.59 (0.26, 1.38) | –0.83 (–2.07, 0.40) | –0.67 (–1.64, 0.31) | 0.23 (0.02, 2.47) |
|  | 3 | 0.80 (0.33, 1.95) | –0.66 (–1.73, 0.42) | –0.65 (–1.65, 0.36) | 0.03 (0.01, 5.84) |
|  | ≥ 4 | 2.22 (0.26, 18.74) | 0.81 (–1.19, 2.80) | 0.40 (–0.35, 1.16) | 5.24 (0.01, 42.00) |
| Annual income ( ≥ 30000 renminbi) | 1 | 1.38 (0.43, 4.46) | 0.47 (–0.97, 1.92) | 0.24 (–0.41, 0.88) | 1.92 (0.20, 18.71) |
|  | 2 | 1.13 (0.46, 2.78) | 0.13 (–0.95, 1.22) | 0.08 (–0.54, 0.70) | 1.23 (0.20, 7.47) |
|  | 3 | 1.09 (0.37, 3.24) | –0.10 (–1.13, 0.92) | –0.08 (–0.93, 0.76) | 0.71 (0.02, 21.17) |
|  | ≥ 4 | 1.39 (0.50, 3.90) | 0.13 (–0.75, 1.00) | 0.09 (–0.55, 0.73) | 1.59 (0.03, 78.95) |
| Cerebrovascular disease | 1 | 1.78 (0.81, 3.90) | **1.26 (0.34, 2.18)** | **0.46 (0.12, 0.79)** | 3.48 (0.37, 32.37) |
|  | 2 | 0.71 (0.33, 1.50) | 0.27 (–1.34, 1.88) | 0.07 (–0.33, 0.46) | 1.09 (0.62, 1.94) |
|  | 3 | 0.88 (0.41, 1.87) | –0.32 (–1.22, 0.59) | –0.23 (–0.88, 0.42) | 0.54 (0.13, 2.29) |
|  | ≥ 4 | 1.13 (0.54, 2.33) | –0.14 (–0.88, 0.61) | –0.12 (–0.77, 0.53) | 0.50 (0.03, 9.80) |
| Rheumatism | 1 | 0.69 (0.30, 1.59) | –0.45 (–1.75, 0.85) | –0.25 (–1.03, 0.52) | 0.63 (0.18, 2.22) |
|  | 2 | 1.15 (0.43, 3.09) | 0.60 (–0.96, 2.15) | 0.23 (–0.34, 0.80) | 1.59 (0.37, 6.84) |
|  | 3 | 1.57 (0.50, 4.96) | 0.04 (–0.76, 0.83) | 0.03 (–0.71, 0.78) | 2.86 (0.01, 13.81) |
|  | ≥ 4 | 0.53 (0.05, 5.74) | –0.79 (–2.34, 0.77) | –1.40 (–5.38, 2.58) | NA^a^ |
| Osteoarthropathy | 1 | 1.35 (0.59, 3.08) | 0.77 (–0.42, 1.96) | 0.29 (–0.16, 0.73) | 1.85 (0.46, 7.43) |
|  | 2 | 1.51 (0.67, 3.38) | **2.12 (0.41, 3.84)** | **0.46 (0.17, 0.75)** | 2.39 (0.96, 5.94) |
|  | 3 | 1.48 (0.63, 3.49) | –0.01 (–0.97, 0.95) | –0.01 (–0.97, 0.95) | NA^a^ |
|  | ≥ 4 | 0.97 (0.45, 2.12) | 0.83 (–0.89, 2.56) | 0.22 (–0.19, 0.63) | 1.42 (0.66, 3.08) |
| Smoking status | 1 | 1.22 (0.52, 2.87) | 0.06 (–1.17, 1.30) | 0.03 (–0.55, 0.61) | 1.06 (0.33, 3.43) |
|  | 2 | 0.53 (0.16, 1.72) | –1.13 (–2.56, 0.29) | –1.29 (–3.39, 0.81) | NA^a^ |
|  | 3 | 1.45 (0.28, 7.52) | 1.05 (–0.58, 2.68) | 0.36 (–0.34, 1.06) | 2.19 (0.12, 40.90) |
|  | ≥ 4 | 0.63 (0.25, 1.63) | –0.89 (–2.46, 0.68) | –0.56 (–1.66, 0.53) | 0.39 (0.08, 2.01) |
| Alcohol consumption | 1 | 1.14 (0.45, 2.88) | –0.34 (–1.64, 0.96) | –0.20 (–0.96, 0.57) | 0.68 (0.19, 2.50) |
|  | 2 | 2.0 (0.19, 21.12) | 0.66 (–2.67, 3.99) | 0.28 (–0.79, 1.34) | 1.90 (0.11, 31.31) |
|  | 3 | 1.53 (0.41, 5.71) | 0.88 (–1.69, 3.45) | 0.30 (–0.37, 0.97) | 1.84 (0.37, 9.18) |
|  | ≥ 4 | 1.79 (0.63, 5.07) | 1.43 (–1.03, 3.90) | 0.42 (–0.05, 0.88) | 2.41 (0.63, 9.20) |
| Low handgrip strength | 1 | 1.59 (0.51, 4.94) | 0.50 (–1.11, 2.12) | 0.22 (–0.39, 0.83) | 1.65 (0.33, 8.16) |
|  | 2 | 0.51 (0.14, 1.85) | –1.30 (–2.56, –0.05) | –1.74 (–4.34, 0.87) | NA^a^ |
|  | 3 | 1.99 (0.85, 4.65) | **2.05 (0.60, 3.51)** | **0.54 (0.24, 0.83)** | 3.64 (0.71, 18.71) |
|  | ≥ 4 | 0.82 (0.37, 1.82) | 1.55 (–0.89, 4.00) | 0.26 (–0.09, 0.61) | 1.45 (0.80, 2.65) |
| Anemia | 1 | 0.91 (0.41, 2.04) | –0.41 (–1.65, 0.84) | –0.21 (–0.89, 0.46) | 0.69 (0.24, 1.96) |
|  | 2 | 1.41 (0.64, 3.09) | 0.11 (–0.88, 1.10) | 0.06 (–0.52, 0.65) | 1.18 (0.22, 6.44) |
|  | 3 | 0.83 (0.35, 1.99) | –0.02 (–1.73, 1.69) | –0.01 (–0.64, 0.63) | 0.99 (0.36, 2.69) |
|  | ≥ 4 | 0.81 (0.29, 2.27) | –0.03 (–1.82, 1.75) | –0.01 (–0.69, 0.66) | 0.98 (0.34, 2.85) |
| Low density lipoprotein cholesterol | 1 | 2.67 (0.85, 8.38) | 0.81 (–0.35, 1.97) | 0.38 (–0.06, 0.82) | 3.45 (0.22, 53.12) |
|  | 2 | 1.22 (0.15, 10.07) | –0.18 (–2.07, 1.72) | –0.10 (–1.26, 1.05) | 0.80 (0.08, 8.52) |
|  | 3 | 1.17 (0.37, 3.69) | 0.40 (–1.50, 2.30) | 0.18 (–0.64, 0.99) | 1.47 (0.17, 12.36) |
|  | ≥ 4 | 2.09 (0.62, 7.10) | 2.35 (–0.07, 4.77) | 0.54 (0.16, 0.92) | 3.33 (0.67, 16.57) |
| Total cholesterol | 1 | 3.86 (0.98, 15.20) | **0.98 (0.12, 1.83)** | **0.95 (0.06, 1.83)** | NA^a^ |
|  | 2 | 2.82 (0.89, 8.99) | **2.97 (0.37, 5.58)** | **0.63 (0.32, 0.95)** | 5.16 (0.70, 38.30) |
|  | 3 | 0.69 (0.19, 2.50) | –0.75 (–2.48, 0.98) | –0.69 (–2.67, 1.28) | 0.10 (0.01, 2.59) |
|  | ≥ 4 | 1.17 (0.30, 4.53) | –0.04 (–2.13, 2.05) | –0.02 (–1.22, 1.17) | 0.95 (0.07, 13.43) |
| Serum creatinine | 1 | 1.87 (0.17, 20.68) | 1.23 (–1.26, 3.73) | 0.48 (–0.55, 1.51) | 4.47 (0.01, 45.18) |
|  | 2 | 0.98 (0.32, 3.08) | –0.01 (–2.15, 2.12) | –0.01 (–0.96, 0.95) | 0.99 (0.18, 5.50) |
|  | 3 | 0.56 (0.13, 2.41) | –1.08 (–2.93, 0.78) | –1.40 (–4.63, 1.83) | NA^a^ |
|  | ≥ 4 | 5.50 (0.54, 56.02) | 4.12 (–2.51, 10.74) | **0.75 (0.37, 1.13)** | 12.15 (0.18, 79.89) |
| Uric acid | 1 | 1.06 (0.19, 5.90) | –0.02 (–2.75, 2.71) | –0.01 (–1.50, 1.48) | 0.98 (0.04, 24.87) |
|  | 2 | 0.75 (0.09, 6.00) | –0.54 (–3.39, 2.31) | –0.39 (–3.14, 2.36) | 0.41 (0.01, 4.62) |
|  | 3 | NA^b^ | NA^b^ | NA^b^ | NA^a^ |
|  | ≥ 4 | 0.71 (0.13, 3.83) | –0.81 (–2.49, 0.88) | –0.92 (–3.80, 1.96) | NA^a^ |
| Aspartate aminotransferase | 1 | 1.68 (0.55, 5.18) | 1.51 (–0.59, 3.61) | 0.45 (–0.03, 0.94) | 2.86 (0.40, 20.63) |
|  | 2 | 1.01 (0.36, 2.88) | 2.29 (–1.82, 6.39) | 0.37 (–0.10, 0.83) | 1.77 (0.69, 4.51) |
|  | 3 | 1.35 (0.43, 4.21) | 0.39 (–1.30, 2.08) | 0.21 (–0.63, 1.05) | 1.85 (0.08, 43.14) |
|  | ≥ 4 | 2.05 (0.70, 6.0) | 0.73 (–0.63, 2.10) | 0.42 (–0.28, 1.12) | 298.56 (0.01, 691.68) |
| Alanine aminotransferase | 1 | 0.59 (0.15, 2.26) | –0.80 (–3.28, 1.68) | –0.43 (–2.13, 1.27) | 0.52 (0.04, 6.06) |
|  | 2 | 0.94 (0.28, 3.21) | 0.23 (–2.02, 2.47) | 0.09 (–0.77, 0.95) | 1.18 (0.23, 6.02) |
|  | 3 | 2.66 (0.66, 10.77) | 0.78 (–1.04, 2.60) | 0.41 (–0.29, 1.11) | 7.60 (0.01, 8.48) |
|  | ≥ 4 | 2.38 (0.25, 22.80) | 1.26 (–1.96, 4.48) | 0.46 (–0.33, 1.24) | 3.61 (0.10, 13.51) |

**Notes:** RERI=0, AP=0, and SI=1 indicate the absence of interactive effects between main explanatory variables and covariates. Conversely, when RERI>0, AP>0, and SI>1, this signifies that the combined effects between main explanatory variables and covariates exceed the sum of their individual effects, suggesting synergistic effects. Conversely, if RERI<0, AP<0, and SI<1, it indicates that the combined effects are smaller than the sum of the individual effects of main explanatory variables and covariates. ^a^, NA indicated the SI of part of covariates in addictive interaction analysis were null because of meaningless statistical value; ^b^, NA indicated the multiplicative interaction and RERI and AP in addictive interaction analysis were null because meaningless statistical value can not calculate 95%CI.Interaction analyses were adjusted for sex, age, ethnic, marital status, educational attainment, occupation, annual income, cerebrovascular disease, rheumatism, osteoarthropathy, smoking status, alcohol consumption, hand grip strength, anemia, total cholesterol, low density lipoprotein cholesterol, aspartate aminotransferase, alanine aminotransferase, serum creatinine, and uric acid.

**Abbreviations:** MetS, metabolic syndrome; ADL, activities of daily living; IADL, instrumental activities of daily living; PR, prevalence ratio; CI, confidence interval; RERI, relative excess risk due to interaction; AP, attribuSupplementary Table proportion due to interaction; SI, synergy index.

**Supplementary Table S18.** Interaction effects of covariates and elevated blood pressure on functional disability

| Functional disability/Covariates | Multiplicative interaction [PR (95%CI)] | Additive interaction [PR (95%CI)] | | |
| --- | --- | --- | --- | --- |
|  |  | RERI | AP | SI |
| **ADL disability** |  |  |  |  |
| Gender | 0.97 (0.60, 1.55) | –0.06 (–0.58, 0.45) | –0.05 (–0.46, 0.35) | 0.79 (0.17, 3.67) |
| Age group | 0.64 (0.39, 1.07) | –0.47 (–1.68, 0.75) | –0.11 (–0.40, 0.17) | 0.87 (0.62, 1.21) |
| Ethnic | 1.38 (0.81, 2.34) | 0.19 (–0.25, 0.64) | 0.23 (–0.36, 0.83) | 0.46 (0.16, 1.33) |
| Marital status | 0.66 (0.42, 1.03) | –0.51 (–1.38, 0.37) | –0.22 (–0.59, 0.15) | 0.72 (0.45, 1.17) |
| Educational attainment (primary school ) | 0.93 (0.57, 1.53) | –0.10 (–0.64, 0.45) | –0.07 (–0.50, 0.35) | 0.75 (0.18, 3.09) |
| Educational attainment (high school and above) | 0.92 (0.46, 1.81) | –0.20 (–0.81, 0.41) | –0.21 (–0.87, 0.45) | NA^a^ |
| Occupation | 2.33 (0.98, 5.58) | **0.77 (0.35, 1.19)** | **0.61 (0.01, 1.22)** | NA^a^ |
| Annual income (10000–29999 renminbi) | 1.20 (0.66, 2.18) | 0.01 (–0.47, 0.49) | 0.01 (–0.52, 0.54) | 0.93 (0.01, 1.11) |
| Annual income ( ≥ 30000 renminbi) | 0.94 (0.56, 1.58) | –0.17 (–0.66, 0.32) | –0.20 (–0.76, 0.35) | NA^a^ |
| Cerebrovascular disease | 1.07 (0.36, 3.15) | 0.10 (–1.19, 1.40) | 0.07 (–0.76, 0.89) | 1.24 (0.07, 21.29) |
| Rheumatism | 0.96 (0.47, 1.94) | –0.04 (–0.82, 0.74) | –0.03 (–0.63, 0.56) | 0.89 (0.09, 8.43) |
| Osteoarthropathy | 1.32 (0.69, 2.52) | 0.26 (–0.38, 0.90) | 0.18 (–0.25, 0.62) | 2.84 (0.04, 21.45) |
| Smoking status | 1.60 (0.74, 3.47) | 0.34 (–0.23, 0.90) | 0.28 (–0.17, 0.73) | NA^a^ |
| Alcohol consumption | 0.78 (0.43, 1.43) | –0.31 (–0.91, 0.28) | –0.32 (–0.94, 0.30) | NA^a^ |
| Low handgrip strength | 1.05 (0.63, 1.77) | 0.46 (–0.25, 1.16) | 0.16 (–0.10, 0.43) | 1.33 (0.75, 2.37) |
| Anemia | 0.89 (0.57, 1.40) | 0.08 (–0.59, 0.75) | 0.04 (–0.27, 0.34) | 1.07 (0.59, 1.95) |
| Low density lipoprotein cholesterol | 1.08 (0.68, 1.74) | 0.04 (–0.43, 0.51) | 0.03 (–0.36, 0.42) | 1.25 (0.06, 2.63) |
| Total cholesterol | 1.15 (0.73, 1.82) | 0.11 (–0.32, 0.54) | 0.09 (–0.28, 0.46) | 2.30 (0.01, 11.45) |
| Serum creatinine | 1.07 (0.63, 1.84) | 0.13 (–0.47, 0.74) | 0.09 (–0.32, 0.50) | 1.42 (0.22, 9.02) |
| Uric acid | 0.63 (0.36, 1.09) | –0.62 (–1.59, 0.36) | –0.37 (–0.98, 0.23) | 0.51 (0.22, 1.22) |
| Aspartate aminotransferase | 1.82 (0.94, 3.55) | **0.48 (0.01, 0.96)** | **0.38 (0.02, 0.74)** | NA^a^ |
| Alanine aminotransferase | 1.32 (0.40, 4.37) | 0.21 (–0.76, 1.18) | 0.17 (–0.58, 0.92) | 7.31 (0.01, 26.87) |
| **IADL disability** |  |  |  |  |
| Gender | 0.84 (0.65, 1.08) | –0.03 (–0.40, 0.33) | –0.01 (–0.15, 0.12) | 0.98 (0.79, 1.21) |
| Age group | 1.14 (0.94, 1.40) | 0.21 (–0.04, 0.47) | 0.11 (–0.03, 0.24) | 1.29 (0.89, 1.86) |
| Ethnic | **1.28 (1.01, 1.63)** | **0.21 (0.01, 0.41)** | 0.25 (–0.02, 0.51) | 0.42 (0.25, 0.71) |
| Marital status | 1.05 (0.86, 1.27) | 0.12 (–0.14, 0.38) | 0.07 (–0.08, 0.22) | 1.19 (0.78, 1.82) |
| Educational attainment (primary school ) | 1.04 (0.83, 1.29) | –0.01 (–0.18, 0.15) | –0.02 (–0.26, 0.22) | 1.05 (0.60, 1.83) |
| Educational attainment (high school and above) | 1.46 (0.92, 2.31) | 0.03 (–0.12, 0.19) | 0.09 (–0.37, 0.55) | 0.96 (0.76, 1.20) |
| Occupation | 1.01 (0.53, 1.93) | 0.24 (–0.38, 0.86) | 0.08 (–0.15, 0.30) | 1.13 (0.75, 1.68) |
| Annual income (10000–29999 renminbi) | 1.13 (0.89, 1.44) | 0.08 (–0.12, 0.27) | 0.09 (–0.14, 0.33) | 0.71 (0.33, 1.49) |
| Annual income ( ≥ 30000 renminbi) | 1.09 (0.86, 1.39) | 0.03 (–0.14, 0.20) | 0.05 (–0.23, 0.32) | 0.93 (0.60, 1.42) |
| Cerebrovascular disease | 0.99 (0.59, 1.66) | –0.02 (–0.52, 0.48) | –0.02 (–0.50, 0.46) | 0.64 (0.01, 2.91) |
| Rheumatism | 0.88 (0.65, 1.19) | –0.14 (–0.46, 0.19) | –0.13 (–0.44, 0.19) | 0.38 (0.03, 4.34) |
| Osteoarthropathy | 1.12 (0.84, 1.50) | 0.09 (–0.17, 0.35) | 0.09 (–0.16, 0.34) | NA^a^ |
| Smoking status | 1.16 (0.78, 1.72) | 0.02 (–0.20, 0.24) | 0.03 (–0.33, 0.39) | 0.95 (0.56, 1.61) |
| Alcohol consumption | 0.96 (0.70, 1.33) | –0.10 (–0.31, 0.11) | –0.16 (–0.49, 0.18) | 1.35 (0.64, 2.82) |
| Low handgrip strength | 1.05 (0.84, 1.30) | 0.12 (–0.14, 0.38) | 0.07 (–0.08, 0.21) | 1.17 (0.79, 1.73) |
| Anemia | 1.12 (0.92, 1.36) | 0.18 (–0.04, 0.40) | 0.12 (–0.03, 0.27) | 1.53 (0.76, 3.12) |
| Low density lipoprotein cholesterol | 0.91 (0.74, 1.12) | –0.10 (–0.31, 0.11) | –0.10 (–0.30, 0.10) | 0.21 (0.01, 12.57) |
| Total cholesterol | 0.93 (0.77, 1.13) | –0.08 (–0.29, 0.13) | –0.07 (–0.26, 0.12) | 0.58 (0.19, 1.74) |
| Serum creatinine | 0.99 (0.79, 1.24) | 0.01 (–0.25, 0.27) | 0.01 (–0.20, 0.21) | 1.03 (0.40, 2.63) |
| Uric acid | 1.02 (0.76, 1.39) | 0.02 (–0.28, 0.32) | 0.02 (–0.25, 0.29) | 1.29 (0.03, 62.19) |
| Aspartate aminotransferase | 0.97 (0.75, 1.24) | –0.04 (–0.29, 0.21) | –0.04 (–0.28, 0.20) | 0.61 (0.03, 10.88) |
| Alanine aminotransferase | 1.01 (0.59, 1.76) | –0.02 (–0.45, 0.42) | –0.02 (–0.53, 0.49) | 1.13 (0.04, 32.80) |
| **Comorbid ADL­IADL disability** |  |  |  |  |
| Gender | 0.96 (0.58, 1.58) | –0.05 (–0.61, 0.51) | –0.04 (–0.45, 0.37) | 0.87 (0.23, 3.33) |
| Age group | 0.68 (0.40, 1.16) | –0.35 (–1.65, 0.95) | –0.08 (–0.38, 0.21) | 0.90 (0.64, 1.28) |
| Ethnic | 1.62 (0.93, 2.80) | 0.33 (–0.07, 0.72) | 0.43 (–0.21, 1.06) | 0.42 (0.21, 0.85) |
| Marital status | 0.68 (0.43, 1.09) | –0.43 (–1.36, 0.49) | –0.18 (–0.55, 0.20) | 0.77 (0.47, 1.26) |
| Educational attainment (primary school ) | 0.99 (0.60, 1.68) | –0.04 (–0.57, 0.49) | –0.03 (–0.48, 0.41) | 0.83 (0.08, 8.18) |
| Educational attainment (high school and above) | 0.92 (0.43, 1.97) | –0.22 (–0.80, 0.36) | –0.32 (–1.14, 0.51) | 4.17 (0.01, 35.63) |
| Occupation | 2.37 (0.93, 6.04) | **0.79 (0.34, 1.23)** | 0.61 (–0.02, 1.23) | NA^a^ |
| Annual income (10000–29999 renminbi) | 1.06 (0.57, 1.95) | –0.07 (–0.59, 0.45) | –0.08 (–0.68, 0.51) | 2.00 (0.01, 6.80) |
| Annual income ( ≥ 30000 renminbi) | 1.01 (0.58, 1.76) | –0.12 (–0.61, 0.38) | –0.14 (–0.72, 0.44) | 3.08 (0.01, 11.46) |
| Cerebrovascular disease | 0.99 (0.33, 2.96) | 0.05 (–1.35, 1.46) | 0.03 (–0.84, 0.91) | 1.10 (0.08, 14.63) |
| Rheumatism | 0.90 (0.44, 1.85) | –0.10 (–0.96, 0.75) | –0.07 (–0.71, 0.56) | 0.78 (0.11, 5.53) |
| Osteoarthropathy | 1.45 (0.72, 2.91) | 0.38 (–0.27, 1.03) | 0.27 (–0.16, 0.70) | 11.99 (0.01, 129.41) |
| Smoking status | 1.63 (0.71, 3.72) | 0.33 (–0.26, 0.91) | 0.28 (–0.19, 0.76) | NA^a^ |
| Alcohol consumption | 0.76 (0.40, 1.46) | –0.36 (–0.97, 0.25) | –0.41 (–1.12, 0.31) | NA^a^ |
| Low handgrip strength | 1.08 (0.62, 1.88) | 0.52 (–0.25, 1.28) | 0.17 (–0.10, 0.45) | 1.36 (0.75, 2.45) |
| Anemia | 0.85 (0.52, 1.37) | 0.01 (–0.76, 0.77) | 0.01 (–0.32, 0.32) | 1.00 (0.58, 1.75) |
| Low density lipoprotein cholesterol | 1.10 (0.67, 1.80) | 0.07 (–0.42, 0.55) | 0.05 (–0.35, 0.46) | 1.47 (0.04, 58.16) |
| Total cholesterol | 1.33 (0.82, 2.15) | 0.30 (–0.15, 0.75) | 0.25 (–0.13, 0.63) | NA^a^ |
| Serum creatinine | 1.06 (0.61, 1.86) | 0.12 (–0.54, 0.77) | 0.08 (–0.36, 0.51) | 1.30 (0.24, 7.09) |
| Uric acid | 0.62 (0.35, 1.10) | –0.65 (–1.70, 0.40) | –0.39 (–1.02, 0.25) | 0.52 (0.21, 1.24) |
| Aspartate aminotransferase | 1.59 (0.81, 3.12) | 0.37 (–0.15, 0.90) | 0.30 (–0.10, 0.71) | NA^a^ |
| Alanine aminotransferasee | 1.10 (0.33, 3.71) | 0.04 (–1.01, 1.09) | 0.03 (–0.88, 0.94) | 1.34 (0.01, 12.66) |

**Notes:** RERI=0, AP=0, and SI=1 indicate the absence of interactive effects between main explanatory variables and covariates. Conversely, when RERI>0, AP>0, and SI>1, this signifies that the combined effects between main explanatory variables and covariates exceed the sum of their individual effects, suggesting synergistic effects. Conversely, if RERI<0, AP<0, and SI<1, it indicates that the combined effects are smaller than the sum of the individual effects of main explanatory variables and covariates. ^a^, NA indicated the SI of part of covariates in addictive interaction analysis were null because of meaningless statistical value.Interaction analyses were adjusted for sex, age, ethnic, marital status, educational attainment, occupation, annual income, cerebrovascular disease, rheumatism, osteoarthropathy, smoking status, alcohol consumption, hand grip strength, anemia, total cholesterol, low density lipoprotein cholesterol, aspartate aminotransferase, alanine aminotransferase, serum creatinine, and uric acid.

**Abbreviations:** ADL= activities of daily living, IADL=instrumental activities of daily living, PR=prevalence ratio, CI=confidence interval, RERI=relative excess risk due to interaction, AP=attribuSupplementary Table proportion due to interaction, SI=synergy index.

**Supplementary Table S19.** Interaction effects of covariates and elevated fasting plasma glucose on functional disability

| Functional disability/Covariates | Multiplicative interaction [PR (95%CI)] | Additive interaction [PR (95%CI)] | | |
| --- | --- | --- | --- | --- |
|  |  | RERI | AP | SI |
| **ADL disability** |  |  |  |  |
| Gender | 0.86 (0.53, 1.38) | –0.20 (–0.77, 0.37) | –0.18 (–0.72, 0.36) | 0.34 (0.01, 12.47) |
| Age group | 1.32 (0.75, 2.33) | 0.83 (–0.16, 1.82) | 0.24 (–0.01, 0.48) | 1.50 (0.91, 2.45) |
| Ethnic | 0.91 (0.53, 1.59) | –0.17 (–0.83, 0.49) | –0.19 (–0.92, 0.55) | NA^a^ |
| Marital status | 1.02 (0.63, 1.63) | 0.19 (–0.59, 0.97) | 0.09 (–0.27, 0.45) | 1.22 (0.54, 2.71) |
| Educational attainment (primary school ) | 1.17 (0.70, 1.97) | 0.17 (–0.43, 0.76) | 0.14 (–0.32, 0.59) | 3.31 (0.01, 30.51) |
| Educational attainment (high school and above) | 1.21 (0.62, 2.38) | 0.10 (–0.53, 0.73) | 0.11 (–0.54, 0.77) | 0.46 (0.01, 12.12) |
| Occupation | 0.73 (0.30, 1.79) | –0.28 (–1.74, 1.17) | –0.13 (–0.78, 0.52) | 0.81 (0.31, 2.10) |
| Annual income (10000–29999 renminbi) | 0.89 (0.48, 1.67) | –0.20 (–0.79, 0.39) | –0.25 (–1.06, 0.56) | 39.59 (0.01, 93.16) |
| Annual income ( ≥ 30000 renminbi) | 0.86 (0.49, 1.49) | –0.24 (–0.78, 0.31) | –0.30 (–1.06, 0.46) | NA^a^ |
| Cerebrovascular disease | 0.68 (0.17, 2.75) | –0.43 (–1.92, 1.06) | –0.41 (–2.33, 1.51) | 0.10 (0.01, 3.43) |
| Rheumatism | 0.98 (0.43, 2.20) | –0.03 (–1.00, 0.93) | –0.03 (–0.83, 0.78) | 0.87 (0.01, 5.74) |
| Osteoarthropathy | 1.68 (0.85, 3.35) | 0.75 (–0.40, 1.90) | 0.40 (0.01, 0.80) | 6.80 (0.19, 23.95) |
| Smoking status | 1.31 (0.68, 2.50) | 0.26 (–0.50, 1.02) | 0.20 (–0.31, 0.71) | 11.90 (0.01, 129.39) |
| Alcohol consumption | 1.26 (0.72, 2.21) | 0.14 (–0.42, 0.71) | 0.14 (–0.36, 0.63) | NA^a^ |
| Low handgrip strength | 1.05 (0.60, 1.86) | 0.34 (–0.54, 1.23) | 0.13 (–0.19, 0.44) | 1.25 (0.68, 2.31) |
| Anemia | 1.19 (0.75, 1.91) | 0.44 (–0.33, 1.21) | 0.21 (–0.11, 0.52) | 1.65 (0.68, 4.01) |
| Low density lipoprotein cholesterol | **0.50 (0.29, 0.84)** | –0.81 (–1.42, –0.20) | –0.99 (–1.98, 0.00) | NA^a^ |
| Total cholesterol | 0.78 (0.49, 1.25) | –0.31 (–0.90, 0.27) | –0.29 (–0.87, 0.29) | 0.20 (0.01, 18.61) |
| Serum creatinine | 1.62 (0.96, 2.75) | 0.70 (–0.08, 1.47) | 0.38 (0.07, 0.70) | 6.83 (0.16, 30.10) |
| Uric acid | 1.31 (0.79, 2.19) | 0.45 (–0.31, 1.22) | 0.25 (–0.10, 0.61) | 2.34 (0.50, 10.96) |
| Aspartate aminotransferase | 1.12 (0.64, 1.96) | 0.11 (–0.55, 0.78) | 0.09 (–0.41, 0.59) | 1.75 (0.05, 57.46) |
| Alanine aminotransferase | 0.39 (0.12, 1.28) | –0.87 (–1.73, –0.01) | –1.53 (–4.46, 1.40) | NA^a^ |
| **IADL disability** |  |  |  |  |
| Gender | 1.09 (0.84, 1.42) | 0.26 (–0.12, 0.63) | 0.11 (–0.04, 0.26) | 1.24 (0.89, 1.71) |
| Age group | 1.18 (0.91, 1.53) | 0.23 (–0.10, 0.56) | 0.11 (–0.04, 0.27) | 1.28 (0.89, 1.85) |
| Ethnic | 0.99 (0.75, 1.33) | –0.01 (–0.29, 0.28) | –0.01 (–0.33, 0.31) | 1.08 (0.07, 15.74) |
| Marital status | 1.08 (0.86, 1.35) | 0.14 (–0.18, 0.46) | 0.08 (–0.09, 0.26) | 1.23 (0.76, 1.99) |
| Educational attainment (primary school ) | 0.96 (0.74, 1.24) | –0.07 (–0.27, 0.14) | –0.10 (–0.42, 0.22) | 1.24 (0.62, 2.50) |
| Educational attainment (high school and above) | 1.11 (0.71, 1.73) | –0.04 (–0.24, 0.16) | –0.12 (–0.75, 0.51) | 1.07 (0.78, 1.46) |
| Occupation | 0.85 (0.45, 1.61) | –0.09 (–0.93, 0.74) | –0.03 (–0.30, 0.24) | 0.96 (0.65, 1.40) |
| Annual income (10000–29999 renminbi) | 0.91 (0.68, 1.22) | –0.10 (–0.35, 0.15) | –0.13 (–0.48, 0.22) | 1.69 (0.38, 7.55) |
| Annual income ( ≥ 30000 renminbi) | 0.86 (0.64, 1.15) | –0.13 (–0.35, 0.08) | –0.24 (–0.65, 0.18) | 1.43 (0.75, 2.71) |
| Cerebrovascular disease | 0.89 (0.44, 1.83) | –0.11 (–0.75, 0.53) | –0.12 (–0.93, 0.68) | 8.92 (0.01, 26.77.) |
| Rheumatism | 1.12 (0.76, 1.64) | 0.12 (–0.30, 0.54) | 0.11 (–0.24, 0.45) | 15.90 (0.01, 39.57) |
| Osteoarthropathy | 1.29 (0.88, 1.88) | 0.25 (–0.16, 0.66) | 0.22 (–0.08, 0.52) | NA^a^ |
| Smoking status | 1.28 (0.86, 1.91) | 0.13 (–0.14, 0.39) | 0.19 (–0.17, 0.55) | 0.73 (0.36, 1.49) |
| Alcohol consumption | 0.83 (0.59, 1.15) | –0.17 (–0.39, 0.04) | –0.33 (–0.81, 0.15) | 1.58 (0.84, 2.98) |
| Low handgrip strength | 1.09 (0.83, 1.42) | 0.12 (–0.20, 0.44) | 0.07 (–0.11, 0.25) | 1.18 (0.73, 1.92) |
| Anemia | 1.07 (0.85, 1.35) | 0.11 (–0.18, 0.40) | 0.08 (–0.11, 0.26) | 1.30 (0.65, 2.62) |
| Low density lipoprotein cholesterol | 1.05 (0.83, 1.34) | 0.05 (–0.19, 0.28) | 0.05 (–0.19, 0.29) | 0.45 (0.01, 51.73) |
| Total cholesterol | 1.09 (0.87, 1.38) | 0.09 (–0.15, 0.32) | 0.08 (–0.14, 0.30) | NA^a^ |
| Serum creatinine | 1.23 (0.96, 1.58) | 0.26 (–0.05, 0.57) | 0.19 (–0.01, 0.39) | 3.28 (0.51, 20.94) |
| Uric acid | 1.21 (0.92, 1.58) | 0.19 (–0.09, 0.48) | 0.17 (–0.06, 0.40) | NA^a^ |
| Aspartate aminotransferase | 0.88 (0.66, 1.18) | –0.13 (–0.41, 0.16) | –0.14 (–0.47, 0.19) | NA^a^ |
| Alanine aminotransferase | 0.92 (0.54, 1.56) | –0.08 (–0.50, 0.34) | –0.11 (–0.69, 0.48) | 1.52 (0.18, 12.56) |
| **Comorbid ADL­IADL disability** |  |  |  |  |
| Gender | 0.98 (0.59, 1.62) | –0.03 (–0.64, 0.58) | –0.02 (–0.52, 0.47) | 0.89 (0.08, 9.98) |
| Age group | 1.22 (0.67, 2.24) | 0.76 (–0.37, 1.88) | 0.20 (–0.06, 0.47) | 1.38 (0.85, 2.23) |
| Ethnic | 0.91 (0.51, 1.63) | –0.17 (–0.87, 0.53) | –0.20 (–0.99, 0.59) | NA^a^ |
| Marital status | 1.12 (0.68, 1.84) | 0.38 (–0.48, 1.24) | 0.17 (–0.17, 0.51) | 1.42 (0.64, 3.17) |
| Educational attainment (primary school ) | 1.05 (0.61, 1.81) | 0.02 (–0.59, 0.64) | 0.02 (–0.51, 0.56) | 1.22 (0.01, 19.85) |
| Educational attainment (high school and above) | 1.07 (0.49, 2.31) | –0.07 (–0.70, 0.55) | –0.11 (–1.05, 0.83) | 1.32 (0.11, 15.53) |
| Occupation | 0.73 (0.28, 1.93) | –0.26 (–1.83, 1.31) | –0.11 (–0.79, 0.56) | 0.83 (0.31, 2.24) |
| Annual income (10000–29999 renminbi) | 0.93 (0.48, 1.78) | –0.18 (–0.80, 0.44) | –0.22 (–1.05, 0.61) | NA^a^ |
| Annual income ( ≥ 30000 renminbi) | 0.77 (0.42, 1.40) | –0.33 (–0.89, 0.24) | –0.48 (–1.41, 0.46) | NA^a^ |
| Cerebrovascular disease | 0.74 (0.18, 2.98) | –0.35 (–1.99, 1.30) | –0.30 (–2.07, 1.47) | 0.32 (0.01, 3.35) |
| Rheumatism | 1.06 (0.47, 2.41) | 0.08 (–0.98, 1.15) | 0.06 (–0.68, 0.80) | 1.30 (0.06, 30.07) |
| Osteoarthropathy | 1.69 (0.82, 3.49) | 0.76 (–0.45, 1.97) | 0.40 (–0.02, 0.83) | 7.02 (0.15, 33.08) |
| Smoking status | 1.43 (0.72, 2.83) | 0.34 (–0.45, 1.14) | 0.26 (–0.24, 0.76) | NA^a^ |
| Alcohol consumption | 0.95 (0.50, 1.78) | –0.15 (–0.73, 0.42) | –0.18 (–0.94, 0.57) | 10.63 (0.01, 30.64) |
| Low handgrip strength | 0.99 (0.54, 1.81) | 0.26 (–0.73, 1.25) | 0.09 (–0.25, 0.43) | 1.17 (0.64, 2.14) |
| Anemia | 0.99 (0.61, 1.65) | 0.18 (–0.67, 1.02) | 0.08 (–0.30, 0.47) | 1.19 (0.52, 2.74) |
| Low density lipoprotein cholesterol | **0.54 (0.31, 0.94)** | –0.72 (–1.37, –0.08) | –0.84 (–1.79, 0.12) | NA^a^ |
| Total cholesterol | 0.73 (0.45, 1.21) | –0.39 (–1.02, 0.23) | –0.37 (–1.03, 0.28) | 0.11 (0.01, 2.40) |
| Serum creatinine | 1.48 (0.85, 2.60) | 0.59 (–0.23, 1.42) | 0.33 (–0.03, 0.69) | 3.95 (0.32, 48.15) |
| Uric acid | 1.25 (0.73, 2.14) | 0.39 (–0.42, 1.21) | 0.22 (–0.17, 0.61) | 1.97 (0.46, 8.44) |
| Aspartate aminotransferase | 1.003 (0.54, 1.85) | –0.02 (–0.71, 0.68) | –0.02 (–0.62, 0.59) | 0.90 (0.01, 55.99) |
| Alanine aminotransferasee | 0.47 (0.14, 1.59) | –0.70 (–1.61, 0.21) | –1.12 (–3.60, 1.37) | NA^a^ |

**Notes:** RERI=0, AP=0, and SI=1 indicate the absence of interactive effects between main explanatory variables and covariates. Conversely, when RERI>0, AP>0, and SI>1, this signifies that the combined effects between main explanatory variables and covariates exceed the sum of their individual effects, suggesting synergistic effects. Conversely, if RERI<0, AP<0, and SI<1, it indicates that the combined effects are smaller than the sum of the individual effects of main explanatory variables and covariates. a, NA indicated the SI of part of covariates in addictive interaction analysis were null because of meaningless statistical value.Interaction analyses were adjusted for sex, age, ethnic, marital status, educational attainment, occupation, annual income, cerebrovascular disease, rheumatism, osteoarthropathy, smoking status, alcohol consumption, hand grip strength, anemia, total cholesterol, low density lipoprotein cholesterol, aspartate aminotransferase, alanine aminotransferase, serum creatinine, and uric acid.

**Abbreviations:** ADL, activities of daily living; IADL, instrumental activities of daily living; PR, prevalence ratio; CI,confidence interval; RERI, relative excess risk due to interaction; AP, attribuSupplementary Table proportion due to interaction; SI, synergy index.

**Supplementary Table S20.** Interaction effects of covariates and elevated triglycerides on functional disability

| Functional disability/Covariates | Multiplicative interaction [PR (95%CI)] | Additive interaction [PR (95%CI)] | | |
| --- | --- | --- | --- | --- |
|  |  | RERI | AP | SI |
| **ADL disability** |  |  |  |  |
| Gender | 1.50 (0.92, 2.47) | 0.36 (–0.04, 0.77) | 0.37 (–0.02, 0.76) | 0.08 (0.01, 2.47) |
| Age group | 1.03 (0.61, 1.72) | 0.16 (–0.78, 1.11) | 0.05 (–0.25, 0.36) | 1.09 (0.67, 1.77) |
| Ethnic | 0.79 (0.46, 1.35) | –0.22 (–0.81, 0.36) | –0.32 (–1.15, 0.50) | 3.75 (0.01, 9.55) |
| Marital status | 1.17 (0.73, 1.88) | 0.21 (–0.45, 0.86) | 0.12 (–0.24, 0.48) | 1.41 (0.47, 4.22) |
| Educational attainment (primary school ) | 0.90 (0.53, 1.52) | –0.11 (–0.65, 0.42) | –0.12 (–0.70, 0.47) | NA^a^ |
| Educational attainment (high school and above) | 0.72 (0.36, 1.44) | –0.25 (–0.78, 0.28) | –0.44 (–1.51, 0.62) | 2.42 (0.17, 34.90) |
| Occupation | 1.03 (0.40, 2.66) | 0.03 (–0.94, 0.99) | 0.02 (–0.59, 0.63) | 1.05 (0.19, 5.91) |
| Annual income (10000–29999 renminbi) | 0.83 (0.44, 1.56) | –0.14 (–0.63, 0.34) | –0.23 (–1.10, 0.63) | 1.56 (0.26, 9.19) |
| Annual income ( ≥ 30000 renminbi) | 0.77 (0.44, 1.36) | –0.20 (–0.66, 0.26) | –0.34 (–1.20, 0.51) | 1.89 (0.27, 13.47) |
| Cerebrovascular disease | **2.67 (1.10, 6.49)** | 1.36 (–0.16, 2.87) | **0.63 (0.28, 0.98)** | NA^a^ |
| Rheumatism | 0.85 (0.36, 1.99) | –0.15 (–0.92, 0.61) | –0.18 (–1.19, 0.83) | NA^a^ |
| Osteoarthropathy | 0.40 (0.15, 1.12) | –0.71 (–1.34, –0.08) | –1.46 (–3.93, 1.02) | NA^a^ |
| Smoking status | 0.51 (0.22, 1.14) | –0.52 (–1.07, 0.04) | –0.98 (–2.59, 0.62) | NA^a^ |
| Alcohol consumption | 0.70 (0.38, 1.31) | –0.27 (–0.74, 0.20) | –0.45 (–1.39, 0.48) | 3.05 (0.15, 60.57) |
| Low handgrip strength | 1.31 (0.76, 2.25) | 0.39 (–0.34, 1.13) | 0.17 (–0.13, 0.46) | 1.42 (0.70, 2.88) |
| Anemia | 1.31 (0.81, 2.12) | 0.39 (–0.32, 1.10) | 0.22 (–0.12, 0.55) | 1.95 (0.56, 6.81) |
| Low density lipoprotein cholesterol | 1.19 (0.72, 1.96) | 0.16 (–0.29, 0.62) | 0.17 (–0.28, 0.63) | 0.23 (0.01, 1.59) |
| Total cholesterol | 1.21 (0.72, 2.03) | 0.17 (–0.29, 0.64) | 0.18 (–0.30, 0.66) | 0.25 (0.01, 23.21) |
| Serum creatinine | 0.77 (0.40, 1.45) | –0.28 (–0.91, 0.35) | –0.30 (–1.11, 0.51) | NA |
| Uric acid | 1.13 (0.68, 1.87) | 0.12 (–0.45, 0.68) | 0.10 (–0.34, 0.53) | 1.99 (0.04, 10.83) |
| Aspartate aminotransferase | 0.84 (0.48, 1.47) | –0.16 (–0.68, 0.36) | –0.19 (–0.86, 0.48) | NA^a^ |
| Alanine aminotransferase | 0.76 (0.31, 1.85) | –0.24 (–1.02, 0.53) | –0.32 (–1.51, 0.86) | NA^a^ |
| **IADL disability** |  |  |  |  |
| Gender | 1.13 (0.85, 1.50) | 0.01 (–0.31, 0.34) | 0.01 (–0.17, 0.18) | 1.02 (0.68, 1.51) |
| Age group | 1.14 (0.89, 1.46) | 0.07 (–0.24, 0.38) | 0.04 (–0.13, 0.20) | 1.09 (0.74, 1.62) |
| Ethnic | 0.86 (0.65, 1.14) | –0.11 (–0.37, 0.15) | –0.15 (–0.52, 0.21) | 1.63 (0.34, 7.87) |
| Marital status | 1.03 (0.82, 1.31) | –0.04 (–0.33, 0.24) | –0.03 (–0.24, 0.18) | 0.90 (0.45, 1.79) |
| Educational attainment (primary school ) | 0.93 (0.71, 1.22) | –0.03 (–0.22, 0.16) | –0.05 (–0.39, 0.29) | 1.07 (0.68, 1.69) |
| Educational attainment (high school and above) | 0.74 (0.46, 1.19) | –0.05 (–0.22, 0.12) | –0.23 (–1.09, 0.63) | 1.07 (0.85, 1.34) |
| Occupation | 0.89 (0.47, 1.68) | –0.36 (–1.10, 0.39) | –0.14 (–0.42, 0.14) | 0.81 (0.56, 1.17) |
| Annual income (10000–29999 renminbi) | 0.89 (0.66, 1.20) | –0.05 (–0.26, 0.17) | –0.08 (–0.45, 0.28) | 1.15 (0.63, 2.08) |
| Annual income ( ≥ 30000 renminbi) | 0.88 (0.65, 1.18) | –0.02 (–0.21, 0.17) | –0.04 (–0.45, 0.37) | 1.04 (0.72, 1.49) |
| Cerebrovascular disease | **1.79 (1.09, 2.93)** | **0.57 (0.02, 1.11)** | **0.46 (0.17, 0.76)** | NA^a^ |
| Rheumatism | 1.20 (0.82, 1.78) | 0.17 (–0.20, 0.54) | 0.18 (–0.16, 0.51) | 0.16 (0.01, 5.77) |
| Osteoarthropathy | 1.17 (0.80, 1.70) | 0.14 (–0.17, 0.46) | 0.17 (–0.16, 0.50) | 0.53 (0.09, 3.18) |
| Smoking status | 0.61 (0.36, 1.01) | –0.16 (–0.37, 0.05) | –0.52 (–1.37, 0.33) | 1.31 (0.91, 1.87) |
| Alcohol consumption | 0.82 (0.57, 1.16) | –0.06 (–0.25, 0.13) | –0.14 (–0.62, 0.34) | 1.11 (0.78, 1.57) |
| Low handgrip strength | 1.08 (0.83, 1.39) | 0.01 (–0.28, 0.29) | 0.01 (–0.18, 0.19) | 1.01 (0.58, 1.77) |
| Anemia | 0.94 (0.72, 1.21) | –0.12 (–0.40, 0.17) | –0.10 (–0.36, 0.16) | 0.57 (0.11, 2.87) |
| Low density lipoprotein cholesterol | 1.26 (0.98, 1.61) | **0.20 (0.01, 0.41)** | **0.24 (0.02, 0.46)** | 0.41 (0.15, 1.16) |
| Total cholesterol | 1.25 (0.95, 1.65) | 0.19 (–0.03, 0.40) | 0.22 (–0.02, 0.46) | 0.43 (0.17, 1.10) |
| Serum creatinine | 1.04 (0.78, 1.39) | 0.01 (–0.28, 0.31) | 0.01 (–0.27, 0.30) | NA^a^ |
| Uric acid | 1.26 (0.97, 1.64) | 0.21 (–0.02, 0.44) | **0.23 (0.01, 0.46)** | 0.29 (0.04, 2.12) |
| Aspartate aminotransferase | 0.88 (0.66, 1.17) | –0.10 (–0.34, 0.13) | –0.13 (–0.46, 0.20) | 1.72 (0.42, 7.10) |
| Alanine aminotransferase | 1.21 (0.76, 1.93) | 0.17 (–0.17, 0.51) | 0.23 (–0.18, 0.64) | 0.61 (0.20, 1.85) |
| **Comorbid ADL­IADL disability** |  |  |  |  |
| Gender | 1.66 (0.97, 2.84) | 0.01 (–0.31, 0.34) | 0.01 (–0.17, 0.18) | 1.02 (0.68, 1.51) |
| Age group | 1.24 (0.70, 2.20) | 0.07 (–0.24, 0.38) | 0.04 (–0.13, 0.20) | 1.09 (0.74, 1.62) |
| Ethnic | 0.93 (0.52, 1.68) | –0.11 (–0.37, 0.15) | –0.15 (–0.52, 0.21) | 1.63 (0.34, 7.87) |
| Marital status | 1.30 (0.79, 2.14) | –0.04 (–0.33, 0.24) | –0.03 (–0.24, 0.18) | 0.90 (0.45, 1.79) |
| Educational attainment (primary school ) | 0.92 (0.53, 1.60) | –0.03 (–0.22, 0.16) | –0.05 (–0.39, 0.29) | 1.07 (0.68, 1.69) |
| Educational attainment (high school and above) | 0.62 (0.27, 1.41) | –0.05 (–0.22, 0.12) | –0.23 (–1.09, 0.63) | 1.07 (0.85, 1.34) |
| Occupation | 0.81 (0.31, 2.17) | –0.36 (–1.10, 0.39) | –0.14 (–0.42, 0.14) | 0.81 (0.56, 1.17) |
| Annual income (10000–29999 renminbi) | 1.0 (0.53, 1.91) | –0.05 (–0.26, 0.17) | –0.08 (–0.45, 0.28) | 1.15 (0.63, 2.08) |
| Annual income ( ≥ 30000 renminbi) | 0.79 (0.43, 1.46) | –0.02 (–0.21, 0.17) | –0.04 (–0.45, 0.37) | 1.04 (0.72, 1.49) |
| Cerebrovascular disease | **2.97 (1.20, 7.36)** | 0.57 (0.02, 1.11) | 0.46 (0.17, 0.76) | NA^a^ |
| Rheumatism | 0.92 (0.39, 2.17) | 0.17 (–0.20, 0.54) | 0.18 (–0.16, 0.51) | 0.16 (0.01, 5.77) |
| Osteoarthropathy | 0.33 (0.10, 1.05) | 0.14 (–0.17, 0.46) | 0.17 (–0.16, 0.50) | 0.53 (0.09, 3.18) |
| Smoking status | **0.30 (0.11, 0.86)** | –0.16 (–0.37, 0.05) | –0.52 (–1.37, 0.33) | 1.31 (0.91, 1.87) |
| Alcohol consumption | 0.56 (0.27, 1.16) | –0.06 (–0.25, 0.13) | –0.14 (–0.62, 0.34) | 1.11 (0.78, 1.57) |
| Low handgrip strength | 1.19 (0.67, 2.11) | 0.01 (–0.28, 0.29) | 0.00 (–0.18, 0.19) | 1.01 (0.58, 1.77) |
| Anemia | 1.40 (0.84, 2.32) | –0.12 (–0.40, 0.17) | –0.10 (–0.36, 0.16) | 0.57 (0.11, 2.87) |
| Low density lipoprotein cholesterol | 1.24 (0.73, 2.09) | **0.20 (0.01, 0.41)** | **0.24 (0.02, 0.46)** | 0.41 (0.15, 1.16) |
| Total cholesterol | 1.31 (0.75, 2.30) | 0.19 (–0.03, 0.40) | 0.22 (–0.02, 0.46) | 0.43 (0.17, 1.10) |
| Serum creatinine | 0.67 (0.33, 1.33) | 0.01 (–0.28, 0.31) | 0.01 (–0.27, 0.30) | NA^a^ |
| Uric acid | 1.14 (0.67, 1.94) | 0.21 (–0.02, 0.44) | **0.23 (0.01, 0.46)** | 0.29 (0.04, 2.12) |
| Aspartate aminotransferase | 0.77 (0.42, 1.41) | –0.10 (–0.34, 0.13) | –0.13 (–0.46, 0.20) | 1.72 (0.42, 7.10) |
| Alanine aminotransferase | 0.76 (0.29, 2.02) | 0.17 (–0.17, 0.51) | 0.23 (–0.18, 0.64) | 0.61 (0.20, 1.85) |

**Notes:** RERI=0, AP=0, and SI=1 indicate the absence of interactive effects between main explanatory variables and covariates. Conversely, when RERI>0, AP>0, and SI>1, this signifies that the combined effects between main explanatory variables and covariates exceed the sum of their individual effects, suggesting synergistic effects. Conversely, if RERI<0, AP<0, and SI<1, it indicates that the combined effects are smaller than the sum of the individual effects of main explanatory variables and covariates. ^a^, NA indicated the SI of part of covariates in addictive interaction analysis were null because of meaningless statistical value.Interaction analyses were adjusted for sex, age, ethnic, marital status, educational attainment, occupation, annual income, cerebrovascular disease, rheumatism, osteoarthropathy, smoking status, alcohol consumption, hand grip strength, anemia, total cholesterol, low density lipoprotein cholesterol, aspartate aminotransferase, alanine aminotransferase, serum creatinine, and uric acid.

**Abbreviations:** ADL, activities of daily living; IADL, instrumental activities of daily living; PR, prevalence ratio; CI, confidence interval; RERI, relative excess risk due to interaction; AP, attribuSupplementary Table proportion due to interaction; SI, synergy index.

**Supplementary Table S21**. Interaction effects of covariates and reduced high density lipoprotein cholesterol on functional disability

| Functional disability/Covariates | Multiplicative interaction [PR (95%CI)] | Additive interaction [PR (95%CI)] | | |
| --- | --- | --- | --- | --- |
|  |  | RERI | AP | SI |
| **ADL disability** |  |  |  |  |
| Gender | 0.97 (0.48, 1.93) | –0.08 (–1.05, 0.89) | –0.06 (–0.81, 0.69) | 0.79 (0.06, 10.47) |
| Age group | **2.18 (1.09, 4.34)** | **1.81 (0.66, 2.95)** | **0.44 (0.25, 0.64)** | **2.41 (1.34, 4.33)** |
| Ethnic | 0.85 (0.47, 1.54) | –0.32 (–1.14, 0.51) | –0.32 (–1.15, 0.52) | NA^a^ |
| Marital status | 1.48 (0.87, 2.49) | 0.80 (–0.03, 1.63) | **0.33 (0.05, 0.62)** | 2.36 (0.85, 6.51) |
| Educational attainment (primary school ) | 0.68 (0.38, 1.24) | –0.50 (–1.23, 0.23) | –0.46 (–1.29, 0.36) | 0.14 (0.01, 108.40) |
| Educational attainment (high school and above) | 0.47 (0.14, 1.51) | –0.78 (–1.58, 0.03) | –1.45 (–4.30, 1.41) | NA^a^ |
| Occupation | 1.25 (0.37, 4.20) | 0.51 (–0.87, 1.89) | 0.23 (–0.40, 0.86) | 1.74 (0.22, 13.72) |
| Annual income (10000–29999 renminbi) | 0.68 (0.34, 1.36) | –0.54 (–1.25, 0.17) | –0.71 (–1.90, 0.48) | NA^a^ |
| Annual income ( ≥ 30000 renminbi) | 0.74 (0.40, 1.36) | –0.48 (–1.16, 0.19) | –0.58 (–1.56, 0.39) | NA^a^ |
| Cerebrovascular disease | 1.87 (0.69, 5.09) | 1.21 (–1.03, 3.45) | 0.47 (–0.04, 0.97) | 4.22 (0.49, 36.31) |
| Rheumatism | **2.51 (1.26, 5.02)** | **1.51 (0.07, 2.95)** | **0.59 (0.31, 0.86)** | 22.54 (0.02, 225.20) |
| Osteoarthropathy | 1.68 (0.80, 3.52) | 0.83 (–0.56, 2.23) | 0.40 (–0.04, 0.83) | 4.13 (0.49, 34.39) |
| Smoking status | 0.97 (0.31, 3.02) | –0.08 (–1.38, 1.21) | –0.07 (–1.27, 1.13) | 0.64 (0.01, 2.80) |
| Alcohol consumption | 1.34 (0.55, 3.25) | 0.22 (–0.84, 1.29) | 0.18 (–0.55, 0.92) | 55.74 (0.01, 263.71) |
| Low handgrip strength | 1.15 (0.62, 2.15) | 0.57 (–0.45, 1.59) | 0.20 (–0.12, 0.52) | 1.43 (0.73, 2.81) |
| Anemia | 1.32 (0.79, 2.20) | 0.66 (–0.20, 1.51) | 0.28 (–0.02, 0.59) | 1.98 (0.77, 5.11) |
| Low density lipoprotein cholesterol | 1.04 (0.60, 1.80) | 0.01 (–0.68, 0.70) | 0.01 (–0.53, 0.55) | 1.04 (0.07, 14.69) |
| Total cholesterol | 1.02 (0.59, 1.78) | 0.01 (–0.69, 0.69) | 0.01 (–0.55, 0.55) | 0.99 (0.07, 14.47) |
| Serum creatinine | 0.46 (0.20, 1.07) | –0.92 (–1.78, –0.06) | –1.03 (–2.68, 0.62) | NA^a^ |
| Uric acid | 1.32 (0.72, 2.40) | 0.59 (–0.54, 1.71) | 0.27 (–0.13, 0.68) | 2.06 (0.60, 7.14) |
| Aspartate aminotransferase | 1.55 (0.84, 2.83) | 0.57 (–0.34, 1.47) | 0.33 (–0.07, 0.73) | 4.97 (0.17, 141.70) |
| Alanine aminotransferase | 1.27 (0.48, 3.33) | 0.26 (–1.03, 1.55) | 0.18 (–0.57, 0.93) | 2.18 (0.06, 73.59) |
| **IADL disability** |  |  |  |  |
| Gender | 0.83 (0.52, 1.34) | –0.28 (–0.85, 0.28) | –0.15 (–0.46, 0.16) | 0.76 (0.45, 1.26) |
| Age group | **1.40 (1.04, 1.88)** | **0.52 (0.15, 0.88)** | **0.23 (0.08, 0.38)** | **1.70 (1.12, 2.58)** |
| Ethnic | 0.88 (0.65, 1.20) | –0.15 (–0.50, 0.20) | –0.16 (–0.54, 0.22) | NA^a^ |
| Marital status | 1.01 (0.78, 1.30) | 0.03 (–0.30, 0.37) | 0.02 (–0.18, 0.22) | 1.05 (0.62, 1.78) |
| Educational attainment (primary school ) | 1.04 (0.76, 1.42) | 0.04 (–0.19, 0.26) | 0.06 (–0.29, 0.40) | 0.91 (0.52, 1.60) |
| Educational attainment (high school and above) | 0.82 (0.37, 1.81) | –0.03 (–0.26, 0.20) | –0.14 (–1.22, 0.94) | 1.04 (0.77, 1.40) |
| Occupation | 1.57 (0.58, 4.22) | 0.57 (–0.20, 1.35) | 0.19 (–0.07, 0.46) | 1.42 (0.79, 2.55) |
| Annual income (10000–29999 renminbi) | 0.71 (0.51, 0.99) | –0.33 (–0.62, –0.05) | –0.49 (–1.00, 0.02) | NA^a^ |
| Annual income ( ≥ 30000 renminbi) | 0.75 (0.54, 1.05) | –0.28 (–0.53, –0.02) | –0.50 (–1.04, 0.04) | 2.66 (0.71, 9.99) |
| Cerebrovascular disease | 1.04 (0.51, 2.09) | 0.03 (–0.70, 0.75) | 0.03 (–0.65, 0.70) | 2.81 (0.01, 39.38) |
| Rheumatism | 1.27 (0.85, 1.88) | 0.27 (–0.22, 0.76) | 0.21 (–0.10, 0.52) | 9.45 (0.01, 89.52) |
| Osteoarthropathy | 1.29 (0.85, 1.95) | 0.26 (–0.21, 0.73) | 0.22 (–0.11, 0.54) | NA^a^ |
| Smoking status | 1.09 (0.52, 2.30) | 0.03 (–0.41, 0.48) | 0.06 (–0.67, 0.78) | 0.93 (0.32, 2.70) |
| Alcohol consumption | 1.19 (0.67, 2.11) | 0.10 (–0.28, 0.47) | 0.16 (–0.36, 0.67) | 0.79 (0.29, 2.14) |
| Low handgrip strength | 0.92 (0.69, 1.23) | –0.06 (–0.44, 0.32) | –0.03 (–0.25, 0.18) | 0.93 (0.58, 1.48) |
| Anemia | 0.96 (0.74, 1.23) | –0.03 (–0.35, 0.30) | –0.02 (–0.25, 0.21) | 0.94 (0.46, 1.92) |
| Low density lipoprotein cholesterol | 1.09 (0.82, 1.44) | 0.08 (–0.21, 0.37) | 0.08 (–0.19, 0.34) | NA^a^ |
| Total cholesterol | 0.97 (0.73, 1.29) | –0.03 (–0.33, 0.27) | –0.03 (–0.33, 0.26) | 0.52 (0.01, 5.41) |
| Serum creatinine | **0.58 (0.39, 0.87)** | –0.57 (–0.95, –0.19) | –0.67 (–1.34, –0.01) | NA^a^ |
| Uric acid | **1.40 (1.02, 1.93)** | 0.39 (–0.01, 0.79) | **0.29 (0.05, 0.52)** | NA^a^ |
| Aspartate aminotransferase | 1.24 (0.90, 1.70) | 0.23 (–0.13, 0.59) | 0.19 (–0.07, 0.45) | NA^a^ |
| Alanine aminotransferase | 1.34 (0.78, 2.30) | 0.25 (–0.29, 0.78) | 0.23 (–0.18, 0.65) | NA^a^ |
| **Comorbid ADL­IADL disability** |  |  |  |  |
| Gender | 0.81 (0.40, 1.64) | –0.32 (–1.45, 0.81) | –0.23 (–1.06, 0.60) | 0.55 (0.10, 3.21) |
| Age group | **2.37 (1.10, 5.11)** | **2.09 (0.77, 3.41)** | **0.46 (0.27, 0.66)** | **2.45 (1.38, 4.35)** |
| Ethnic | 0.83 (0.45, 1.53) | –0.38 (–1.29, 0.52) | –0.37 (–1.27, 0.52) | 0.05 (0.01, 3.83) |
| Marital status | 1.50 (0.86, 2.62) | 0.90 (–0.02, 1.82) | **0.35 (0.06, 0.63)** | 2.31 (0.89, 5.98) |
| Educational attainment (primary school ) | 0.68 (0.36, 1.28) | –0.50 (–1.23, 0.23) | –0.51 (–1.43, 0.42) | NA^a^ |
| Educational attainment (high school and above) | 0.65 (0.20, 2.14) | –0.55 (–1.36, 0.26) | –0.98 (–3.32, 1.37) | NA^a^ |
| Occupation | 1.74 (0.40, 7.56) | 0.89 (–0.42, 2.20) | 0.39 (–0.18, 0.95) | 3.16 (0.10, 97.18) |
| Annual income (10000–29999 renminbi) | 0.77 (0.38, 1.57) | –0.44 (–1.19, 0.31) | –0.53 (–1.61, 0.56) | NA^a^ |
| Annual income ( ≥ 30000 renminbi) | 0.74 (0.39, 1.42) | –0.49 (–1.20, 0.22) | –0.62 (–1.70, 0.46) | NA^a^ |
| Cerebrovascular disease | 1.97 (0.71, 5.46) | 1.45 (–1.06, 3.96) | **0.50 (0.03, 0.98)** | 4.28 (0.58, 31.28) |
| Rheumatism | **2.30 (1.11, 4.76)** | 1.46 (–0.11, 3.02) | **0.55 (0.24, 0.86)** | 9.21 (0.43, 195.41) |
| Osteoarthropathy | 1.85 (0.87, 3.94) | 1.05 (–0.48, 2.58) | **0.45 (0.05, 0.85)** | 4.87 (0.57, 41.37) |
| Smoking status | 1.10 (0.35, 3.44) | 0.05 (–1.37, 1.46) | 0.04 (–1.05, 1.12) | 1.21 (0.01, 29.46) |
| Alcohol consumption | 1.63 (0.66, 4.02) | 0.41 (–0.74, 1.56) | 0.31 (–0.33, 0.94) | NA^a^ |
| Low handgrip strength | 1.34 (0.67, 2.68) | 0.87 (–0.25, 1.98) | 0.27 (–0.03, 0.58) | 1.67 (0.82, 3.40) |
| Anemia | 1.30 (0.76, 2.22) | 0.71 (–0.25, 1.66) | 0.28 (–0.03, 0.60) | 1.88 (0.78, 4.56) |
| Low density lipoprotein cholesterol | 1.07 (0.60, 1.91) | 0.06 (–0.69, 0.81) | 0.04 (–0.50, 0.59) | 1.19 (0.11, 12.56) |
| Total cholesterol | 1.08 (0.60, 1.92) | 0.06 (–0.68, 0.80) | 0.05 (–0.50, 0.59) | 1.23 (0.10, 15.48) |
| Serum creatinine | **0.39 (0.16, 0.99)** | –1.12 (–2.04, –0.19) | –1.32 (–3.40, 0.75) | NA^a^ |
| Uric acid | 1.46 (0.79, 2.69) | 0.85 (–0.42, 2.11) | 0.35 (–0.02, 0.72) | 2.46 (0.74, 8.20) |
| Aspartate aminotransferase | 1.80 (0.97, 3.35) | 0.80 (–0.20, 1.79) | **0.42 (0.06, 0.78)** | 9.00 (0.06, 124.90) |
| Alanine aminotransferase | 1.55 (0.57, 4.21) | 0.49 (–0.93, 1.92) | 0.30 (–0.35, 0.95) | 4.18 (0.06, 30.50) |

**Notes:** RERI=0, AP=0, and SI=1 indicate the absence of interactive effects between main explanatory variables and covariates. Conversely, when RERI>0, AP>0, and SI>1, this signifies that the combined effects between main explanatory variables and covariates exceed the sum of their individual effects, suggesting synergistic effects. Conversely, if RERI<0, AP<0, and SI<1, it indicates that the combined effects are smaller than the sum of the individual effects of main explanatory variables and covariates. ^a^, NA indicated the SI of part of covariates in addictive interaction analysis were null because of meaningless statistical value.Interaction analyses were adjusted for sex, age, ethnic, marital status, educational attainment, occupation, annual income, cerebrovascular disease, rheumatism, osteoarthropathy, smoking status, alcohol consumption, hand grip strength, anemia, total cholesterol, low density lipoprotein cholesterol, aspartate aminotransferase, alanine aminotransferase, serum creatinine, and uric acid.

**Abbreviations:** ADL, activities of daily living; IADL, instrumental activities of daily living; PR, prevalence ratio; CI, confidence interval; RERI, relative excess risk due to interaction; AP, attribuSupplementary Table proportion due to interaction; SI, synergy index.

**Supplementary Table S22.** Interaction effects of covariates and elevated waist circumference on functional disability

| Functional disability/Covariates | Multiplicative interaction [PR (95%CI)] | Additive interaction [PR (95%CI)] | | |
| --- | --- | --- | --- | --- |
|  |  | RERI | AP | SI |
| **ADL disability** |  |  |  |  |
| Gender | 0.90 (0.60, 1.36) | –0.15 (–0.67, 0.37) | –0.12 (–0.55, 0.30) | 0.61 (0.14, 2.71) |
| Age group | 0.97 (0.63, 1.51) | 0.54 (–0.34, 1.42) | 0.14 (–0.08, 0.37) | 1.25 (0.86, 1.81) |
| Ethnic | 1.58 (0.97, 2.57) | 0.35 (–0.04, 0.74) | 0.42 (–0.09, 0.92) | 0.31 (0.09, 1.13) |
| Marital status | 1.08 (0.73, 1.60) | 0.31 (–0.31, 0.94) | 0.14 (–0.13, 0.42) | 1.37 (0.70, 2.68) |
| Educational attainment (primary school ) | 0.98 (0.64, 1.51) | –0.04 (–0.53, 0.45) | –0.03 (–0.45, 0.39) | 0.83 (0.08, 8.78) |
| Educational attainment (high school and above) | 1.09 (0.61, 1.97) | –0.02 (–0.56, 0.52) | –0.02 (–0.61, 0.57) | 1.34 (0.01, 5.44) |
| Occupation | 0.66 (0.28, 1.54) | –0.37 (–1.81, 1.08) | –0.14 (–0.65, 0.37) | 0.82 (0.43, 1.55) |
| Annual income (10000–29999 renminbi) | 1.25 (0.76, 2.07) | 0.09 (–0.38, 0.56) | 0.09 (–0.39, 0.57) | 0.35 (0.01, 3.56) |
| Annual income ( ≥ 30000 renminbi) | 1.08 (0.68, 1.70) | –0.04 (–0.48, 0.41) | –0.04 (–0.55, 0.46) | 1.45 (0.01, 271.78) |
| Cerebrovascular disease | 1.01 (0.40, 2.53) | 0.06 (–1.23, 1.34) | 0.04 (–0.78, 0.86) | 1.12 (0.09, 14.47) |
| Rheumatism | 1.09 (0.57, 2.08) | 0.10 (–0.70, 0.90) | 0.07 (–0.48, 0.63) | 1.37 (0.12, 15.44) |
| Osteoarthropathy | 1.03 (0.59, 1.82) | 0.05 (–0.66, 0.75) | 0.03 (–0.47, 0.54) | 1.15 (0.15, 8.65) |
| Smoking status | 1.13 (0.61, 2.08) | 0.09 (–0.60, 0.77) | 0.07 (–0.47, 0.61) | 1.72 (0.02, 128.32) |
| Alcohol consumption | 0.99 (0.58, 1.72) | –0.08 (–0.59, 0.43) | –0.08 (–0.66, 0.49) | NA^a^ |
| Low handgrip strength | 1.13 (0.71, 1.78) | 0.66 (–0.07, 1.40) | 0.22 (–0.01, 0.44) | 1.48 (0.89, 2.44) |
| Anemia | 1.23 (0.83, 1.82) | 0.50 (–0.10, 1.10) | 0.23 (–0.02, 0.48) | 1.77 (0.81, 3.87) |
| Low density lipoprotein cholesterol | 0.84 (0.56, 1.26) | –0.24 (–0.73, 0.26) | –0.20 (–0.63, 0.24) | 0.44 (0.08, 2.49) |
| Total cholesterol | 0.93 (0.63, 1.38) | –0.11 (–0.59, 0.37) | –0.09 (–0.49, 0.31) | 0.66 (0.13, 3.31) |
| Serum creatinine | 0.93 (0.58, 1.50) | –0.06 (–0.67, 0.56) | –0.04 (–0.48, 0.40) | 0.88 (0.22, 3.57) |
| Uric acid | 1.27 (0.81, 2.0) | 0.41 (–0.21, 1.03) | 0.23 (–0.08, 0.54) | 2.13 (0.53, 8.50) |
| Aspartate aminotransferase | 1.26 (0.78, 2.05) | 0.24 (–0.31, 0.80) | 0.18 (–0.19, 0.55) | 3.37 (0.06, 195.19) |
| Alanine aminotransferase | 2.32 (0.91, 5.94) | 0.77 (–0.10, 1.65) | 0.49 (0.10, 0.88) | NA^a^ |
| **IADL disability** |  |  |  |  |
| Gender | 1.08 (0.84, 1.38) | 0.09 (–0.20, 0.38) | 0.04 (–0.10, 0.19) | 1.09 (0.80, 1.49) |
| Age group | **1.24 (1.01, 1.51)** | **0.40 (0.14, 0.66)** | **0.18 (0.07, 0.29)** | **1.49 (1.10, 2.03)** |
| Ethnic | 1.18 (0.93, 1.49) | 0.14 (–0.07, 0.36) | 0.16 (–0.09, 0.40) | 0.38 (0.11, 1.26) |
| Marital status | 1.07 (0.89, 1.28) | 0.15 (–0.10, 0.41) | 0.09 (–0.05, 0.22) | 1.24 (0.85, 1.80) |
| Educational attainment (primary school ) | 1.02 (0.83, 1.26) | 0.00 (–0.15, 0.16) | 0.01 (–0.24, 0.25) | 0.99 (0.64, 1.53) |
| Educational attainment (high school and above) | 1.16 (0.78, 1.72) | 0.03 (–0.13, 0.18) | 0.08 (–0.40, 0.57) | 0.96 (0.77, 1.20) |
| Occupation | 1.06 (0.60, 1.86) | 0.31 (–0.28, 0.90) | 0.10 (–0.10, 0.30) | 1.17 (0.81, 1.69) |
| Annual income (10000–29999 renminbi) | 0.90 (0.72, 1.13) | –0.13 (–0.34, 0.08) | –0.16 (–0.42, 0.10) | 5.21 (0.01, 30.11) |
| Annual income ( ≥ 30000 renminbi) | 0.96 (0.77, 1.21) | –0.11 (–0.30, 0.08) | –0.16 (–0.44, 0.13) | 1.50 (0.64, 3.47) |
| Cerebrovascular disease | 1.19 (0.74, 1.91) | 0.17 (–0.33, 0.68) | 0.15 (–0.24, 0.54) | NA^a^ |
| Rheumatism | 0.97 (0.71, 1.33) | –0.03 (–0.37, 0.30) | –0.03 (–0.35, 0.28) | 0.71 (0.02, 25.54) |
| Osteoarthropathy | 1.09 (0.82, 1.44) | 0.07 (–0.21, 0.35) | 0.07 (–0.19, 0.32) | NA^a^ |
| Smoking status | 1.03 (0.69, 1.52) | –0.02 (–0.26, 0.21) | –0.04 (–0.46, 0.38) | 1.05 (0.60, 1.84) |
| Alcohol consumption | 0.92 (0.67, 1.27) | –0.08 (–0.27, 0.12) | –0.14 (–0.52, 0.24) | 1.20 (0.75, 1.92) |
| Low handgrip strength | 1.14 (0.93, 1.40) | **0.28 (0.02, 0.54)** | **0.14 (0.01, 0.27)** | 1.41 (0.97, 2.04) |
| Anemia | 1.10 (0.92, 1.33) | 0.17 (–0.06, 0.41) | 0.11 (–0.03, 0.25) | 1.44 (0.84, 2.46) |
| Low density lipoprotein cholesterol | 1.02 (0.84, 1.23) | 0.01 (–0.19, 0.20) | 0.01 (–0.18, 0.20) | 1.71 (0.01, 23.00) |
| Total cholesterol | 1.05 (0.87, 1.26) | 0.04 (–0.15, 0.24) | 0.04 (–0.14, 0.22) | 1.87 (0.04, 87.49) |
| Serum creatinine | 0.90 (0.73, 1.12) | –0.10 (–0.37, 0.16) | –0.08 (–0.30, 0.14) | 0.69 (0.27, 1.80) |
| Uric acid | 1.13 (0.90, 1.43) | 0.13 (–0.12, 0.37) | 0.11 (–0.09, 0.31) | 7.11 (0.01, 11.26) |
| Aspartate aminotransferase | 1.09 (0.87, 1.37) | 0.08 (–0.15, 0.32) | 0.08 (–0.13, 0.28) | 4.79 (0.01, 20.04) |
| Alanine aminotransferase | 1.30 (0.83, 2.04) | 0.19 (–0.18, 0.56) | 0.19 (–0.15, 0.54) | 0.14 (0.01, 17.53) |
| **Comorbid ADL­IADL disability** |  |  |  |  |
| Gender | 0.94 (0.60, 1.46) | –0.08 (–0.63, 0.46) | –0.07 (–0.50, 0.37) | 0.76 (0.15, 3.94) |
| Age group | 1.26 (0.77, 2.04) | **0.91 (0.01, 1.82)** | **0.24 (0.02, 0.45)** | 1.48 (0.97, 2.26) |
| Ethnic | **1.79 (1.06, 3.02)** | **0.44 (0.07, 0.82)** | **0.56 (0.03, 1.09)** | 0.32 (0.12, 0.84) |
| Marital status | 1.24 (0.82, 1.88) | 0.52 (–0.14, 1.17) | 0.23 (–0.03, 0.49) | 1.70 (0.79, 3.66) |
| Educational attainment (primary school ) | 1.10 (0.70, 1.74) | 0.08 (–0.39, 0.54) | 0.07 (–0.35, 0.49) | 10.62 (0.01, 16.58) |
| Educational attainment (high school and above) | 1.20 (0.61, 2.35) | 0.03 (–0.46, 0.52) | 0.05 (–0.65, 0.74) | 0.91 (0.20, 4.14) |
| Occupation | 0.53 (0.21, 1.35) | –0.77 (–2.69, 1.15) | –0.26 (–0.81, 0.29) | 0.72 (0.41, 1.27) |
| Annual income (10000–29999 renminbi) | 1.19 (0.70, 2.03) | 0.05 (–0.43, 0.54) | 0.06 (–0.48, 0.60) | 0.68 (0.03, 17.76) |
| Annual income ( ≥ 30000 renminbi) | 1.11 (0.68, 1.81) | –0.01 (–0.47, 0.44) | –0.02 (–0.56, 0.53) | 1.09 (0.06, 20.09) |
| Cerebrovascular disease | 1.14 (0.45, 2.90) | 0.24 (–1.14, 1.62) | 0.15 (–0.60, 0.89) | 1.58 (0.13, 18.90) |
| Rheumatism | 0.95 (0.48, 1.89) | –0.05 (–0.89, 0.79) | –0.04 (–0.70, 0.63) | 0.86 (0.06, 12.92) |
| Osteoarthropathy | 1.05 (0.58, 1.92) | 0.07 (–0.66, 0.80) | 0.05 (–0.48, 0.58) | 1.25 (0.11, 13.78) |
| Smoking status | 1.15 (0.59, 2.23) | 0.10 (–0.60, 0.79) | 0.09 (–0.49, 0.66) | 3.88 (0.01, 19.61) |
| Alcohol consumption | 0.88 (0.47, 1.62) | –0.18 (–0.68, 0.32) | –0.24 (–1.00, 0.51) | 3.53 (0.01, 9.59) |
| Low handgrip strength | 1.27 (0.77, 2.07) | **0.80 (0.04, 1.57)** | **0.26 (0.03, 0.49)** | 1.64 (0.93, 2.88) |
| Anemia | 1.33 (0.88, 2.01) | 0.61 (–0.03, 1.25) | **0.28 (0.02, 0.53)** | 2.00 (0.84, 4.73) |
| Low density lipoprotein cholesterol | 0.91 (0.59, 1.39) | –0.14 (–0.64, 0.36) | –0.12 (–0.56, 0.33) | 0.53 (0.06, 4.92) |
| Total cholesterol | 1.03 (0.68, 1.58) | 0.02 (–0.46, 0.49) | 0.01 (–0.39, 0.42) | 1.09 (0.06, 18.92) |
| Serum creatinine | 0.87 (0.52, 1.43) | –0.15 (–0.80, 0.51) | –0.11 (–0.61, 0.40) | 0.71 (0.15, 3.33) |
| Uric acid | 1.33 (0.82, 2.14) | 0.46 (–0.19, 1.12) | 0.26 (–0.06, 0.58) | 2.45 (0.48, 12.50) |
| Aspartate aminotransferase | 1.16 (0.70, 1.95) | 0.15 (–0.43, 0.72) | 0.12 (–0.31, 0.55) | 2.51 (0.02, 26.39) |
| Alanine aminotransferase | 1.90 (0.72, 5.03) | 0.55 (–0.33, 1.44) | 0.41 (–0.09, 0.90) | NA^a^ |

**Notes:** RERI=0, AP=0, and SI=1 indicate the absence of interactive effects between main explanatory variables and covariates. Conversely, when RERI>0, AP>0, and SI>1, this signifies that the combined effects between main explanatory variables and covariates exceed the sum of their individual effects, suggesting synergistic effects. Conversely, if RERI<0, AP<0, and SI<1, it indicates that the combined effects are smaller than the sum of the individual effects of main explanatory variables and covariates. ^a^, NA indicated the SI of part of covariates in addictive interaction analysis were null because of meaningless statistical value.Interaction analyses were adjusted for sex, age, ethnic, marital status, educational attainment, occupation, annual income, cerebrovascular disease, rheumatism, osteoarthropathy, smoking status, alcohol consumption, hand grip strength, anemia, total cholesterol, low density lipoprotein cholesterol, aspartate aminotransferase, alanine aminotransferase, serum creatinine, and uric acid.

**Abbreviations:** ADL, activities of daily living; IADL, instrumental activities of daily living; PR, prevalence ratio; CI, confidence interval; RERI, relative excess risk due to interaction; AP, attribuSupplementary Table proportion due to interaction; SI, synergy index.

**Supplementary Table S23.** Associations between MetS and functional disability calculate by binary logistic regression analysis

| Models | ADL disability [PR (95% CI)] | | IADL disability [PR (95% CI)] | | Comorbid ADL­IADL disability [PR (95% CI)] | |
| --- | --- | --- | --- | --- | --- | --- |
|  | Without MetS | With MetS | Without MetS | With MetS | Without MetS | With MetS |
| Model 1 | 1.00 (Ref) | **1.71 (1.34,2.19)***** | 1.00 (Ref) | 1.03 (0.87,1.22) | 1.00 (Ref) | **1.64 (1.26,2.13)***** |
| Model 2 | 1.00 (Ref) | **1.77 (1.35,2.31)***** | 1.00 (Ref) | 0.93 (0.77,1.13) | 1.00 (Ref) | **1.68 (1.26,2.23)***** |
| Model 3 | 1.00 (Ref) | **1.78 (1.33,2.40)***** | 1.00 (Ref) | 0.88 (0.71,1.08) | 1.00 (Ref) | **1.67 (1.22,2.28)**** |

**Abbreviations:** MetS, metabolic syndrome; ADL, activities of daily living; IADL, instrumental activities of daily living; PR, prevalence ratio; CI, confidence interval.

**Notes:** **, P<0.01; ***, P<0.001.Model 1 was unadjusted model;Model 2 adjusted for sex, age, ethnic, marital status, educational attainment, occupation, annual income, cerebrovascular disease, rheumatism, osteoarthropathy, smoking status, and alcohol consumption;Model 3 further adjusted hand grip strength, anemia, total cholesterol, low density lipoprotein cholesterol, aspartate aminotransferase, alanine aminotransferase, serum creatinine, and uric acid.

**Supplementary Table S24.** Associations between number of MetS components and functional disability analyzed using binary logistic regression analysis

| Functional disability/Models | Number of MetS components [PR (95% CI)] | | | | | PR (95% CI) for trend |
| --- | --- | --- | --- | --- | --- | --- |
|  | 0 | 1 | 2 | 3 | ≥4 |  |
| **ADL disability** |  |  |  |  |  |  |
| Model 1 | 1.00 (Ref) | 1.29 (0.91,1.82) | **1.70 (1.19,2.43)**** | **2.38 (1.61,3.51)***** | **2.12 (1.25,3.59)**** | **1.27 (1.16,1.40)***** |
| Model 2 | 1.00 (Ref) | 1.05 (0.73,1.52) | 1.45 (1.00,2.12) | **2.22 (1.47,3.36)***** | 1.70 (0.96,2.99) | **1.26 (1.14,1.40)***** |
| Model 3 | 1.00 (Ref) | 1.13 (0.78,1.65) | **1.63 (1.10,2.41)*** | **2.47 (1.59,3.85)***** | 1.79 (0.94,3.38) | **1.29 (1.15,1.45)***** |
| **IADL disability** |  |  |  |  |  |  |
| Model 1 | 1.00 (Ref) | **1.21 (1.00,1.45)*** | **1.36 (1.12,1.66)**** | 1.26 (1.00,1.61) | 1.18 (0.84,1.66) | **1.07 (1.00,1.13)*** |
| Model 2 | 1.00 (Ref) | 1.08 (0.88,1.32) | 1.18 (0.95,1.48) | 1.09 (0.84,1.43) | 0.84 (0.57,1.23) | 1.01 (0.94,1.08) |
| Model 3 | 1.00 (Ref) | 1.09 (0.89,1.35) | 1.20 (0.95,1.51) | 1.07 (0.81,1.43) | 0.73 (0.48,1.11) | 0.99 (0.92,1.07) |
| **Comorbid ADL­IADL disability** |  |  |  |  |  |  |
| Model 1 | 1.00 (Ref) | 1.39 (0.97,2.01) | **1.76 (1.20,2.57)**** | **2.40 (1.59,3.63)***** | **2.10 (1.20,3.68)**** | **1.26 (1.14,1.39)***** |
| Model 2 | 1.00 (Ref) | 1.14 (0.77,1.67) | 1.48 (0.99,2.21) | **2.22 (1.43,3.45)***** | 1.64 (0.89,3.00) | **1.24 (1.11,1.38)***** |
| Model 3 | 1.00 (Ref) | 1.24 (0.83,1.84) | **1.65 (1.09,2.51)*** | **2.48 (1.55,3.98)***** | 1.59 (0.79,3.18) | **1.26 (1.11,1.42)***** |

**Notes:** *, *P*<0.05, **, *P*<0.01; ***, *P*<0.001. Model 1 was unadjusted model; Model 2 adjusted for sex, age, ethnic, marital status, educational attainment, occupation, annual income, cerebrovascular disease, rheumatism, osteoarthropathy, smoking status, and alcohol consumption; Model 3 further adjusted hand grip strength, anemia, total cholesterol, low density lipoprotein cholesterol, aspartate aminotransferase, alanine aminotransferase, serum creatinine, and uric acid.

**Abbreviations:** MetS, metabolic syndrome; ADL, activities of daily living; IADL, instrumental activities of daily living; PR, prevalence ratio; CI, confidence interval.

**Supplementary Table S25**. Associations between individual MetS components and functional disability analyzed using binary logistic regression analysis

| Functional disability/Models | PR (95% CI) | | | | |
| --- | --- | --- | --- | --- | --- |
|  | Elevated blood pressure ^a^ | Elevated fasting glucose ^b^ | Elevated triglycerides ^c^ | Reduced HDL cholesterol ^d^ | Abdominal obesity ^e^ |
| ADL disability |  |  |  |  |  |
| Model 1 | **1.37 (1.07,1.76)*** | 1.30 (0.99,1.69) | 0.98 (0.74,1.28) | **1.44 (1.08,1.93)*** | **1.36 (1.09,1.71)**** |
| Model 2 | 1.16 (0.89,1.50) | 1.16 (0.87,1.55) | 1.07 (0.80,1.44) | **1.37 (1.00,1.89)*** | **1.50 (1.18,1.92)**** |
| Model 3 | 1.22 (0.93,1.60) | 1.15 (0.85,1.54) | 1.06 (0.74,1.51) | 1.36 (0.94,1.97) | **1.60 (1.23,2.07)***** |
| IADL disability |  |  |  |  |  |
| Model 1 | **1.18 (1.02,1.36)*** | 1.07 (0.90,1.27) | **0.79 (0.66,0.94)**** | 1.15 (0.94,1.39) | **1.18 (1.02,1.36)*** |
| Model 2 | 1.10 (0.94,1.29) | 1.07 (0.88,1.30) | 0.92 (0.76,1.12) | **0.77 (0.62,0.95)*** | 1.07 (0.91,1.25) |
| Model 3 | 1.12 (0.95,1.32) | 1.05 (0.85,1.29) | 0.88 (0.69,1.12) | **0.76 (0.60,0.98)*** | 1.06 (0.90,1.26) |
| Comorbid ADL­IADL disability |  |  |  |  |  |
| Model 1 | **1.36 (1.05,1.76)*** | 1.28 (0.97,1.70) | 0.97 (0.73,1.30) | **1.48 (1.10,2.00)*** | **1.30 (1.03,1.65)*** |
| Model 2 | 1.14 (0.87,1.50) | 1.16 (0.86,1.56) | 1.09 (0.80,1.48) | 1.36 (0.98,1.90) | **1.42 (1.09,1.83)**** |
| Model 3 | 1.22 (0.92,1.62) | 1.14 (0.84,1.56) | 1.10 (0.75,1.60) | 1.31 (0.89,1.93) | **1.45 (1.10,1.90)**** |

**Notes:** ^a^, the reference group was non–elevated blood pressure; ^b^, the reference group was non–elevated fasting glucose; ^c^, the reference group was non–elevated triglycerides; ^d^, the reference group was non–reduced HDL cholesterol; ^e^, the reference group was non–abdominal obesity. *, *P*<0.05; **, *P*<0.01; ***, *P*<0.001. Model 1 was unadjusted model; Model 2 adjusted for sex, age, ethnic, marital status, educational attainment, occupation, annual income, cerebrovascular disease, rheumatism, osteoarthropathy, smoking status, and alcohol consumption; Model 3 further adjusted hand grip strength, anemia, total cholesterol, low density lipoprotein cholesterol, aspartate aminotransferase, alanine aminotransferase, serum creatinine, and uric acid.

**Abbreviations:** MetS, metabolic syndrome; ADL, activities of daily living; IADL, instrumental activities of daily living; PR, prevalence ratio; CI, confidence interval.

**Supplementary Table S26.** Association between various combinations of MetS components and functional disability analyzed using binary logistic regression analysis

| Components/combinations | ADL disability [PR (95% CI)] | | | IADL disability [PR (95% CI)] | | | Comorbid ADL­IADL disability [PR (95% CI)] | | |
| --- | --- | --- | --- | --- | --- | --- | --- | --- | --- |
|  | Model 1 | Model 2 | Model 3 | Model 1 | Model 2 | Model 3 | Model 1 | Model 2 | Model 3 |
| **One MetS Component** |  |  |  |  |  |  |  |  |  |
| None | 1.00 (Ref) | 1.00 (Ref) | 1.00 (Ref) | 1.00 (Ref) | 1.00 (Ref) | 1.00 (Ref) | 1.00 (Ref) | 1.00 (Ref) | 1.00 (Ref) |
| Elevated blood pressure | 1.31 (0.92,1.88) | 1.01 (0.69,1.48) | 1.03 (0.70,1.51) | **1.23 (1.02,1.50)*** | 1.12 (0.90,1.38) | 1.13 (0.91,1.41) | 1.43 (0.98,2.09) | 1.11 (0.75,1.66) | 1.12 (0.75,1.68) |
| Elevated fasting glucose | 1.15 (0.48,2.76) | 1.07 (0.43,2.62) | 1.10 (0.45,2.72) | 1.04 (0.64,1.69) | 1.10 (0.65,1.85) | 1.09 (0.64,1.84) | 1.09 (0.42,2.85) | 1.02 (0.38,2.70) | 1.05 (0.39,2.79) |
| Elevated triglycerides | 1.02 (0.36,2.92) | 0.81 (0.23,2.86) | 0.80 (0.22,2.87) | 0.85 (0.47,1.52) | 1.03 (0.53,2.00) | 1.04 (0.54,2.01) | 1.18 (0.41,3.41) | 0.98 (0.27,3.48) | 0.98 (0.27,3.52) |
| Reduced HDL cholesterol | 1.29 (0.57,2.95) | 1.02 (0.43,2.46) | 1.04 (0.43,2.50) | 0.78 (0.47,1.29) | **0.43 (0.25,0.76)**** | **0.44 (0.25,0.76)**** | 1.50 (0.65,3.44) | 1.19 (0.49,2.90) | 1.21 (0.50,2.93) |
| Abdominal obesity | 1.23 (0.64,2.38) | 1.26 (0.63,2.52) | 1.24 (0.62,2.49) | 1.54 (1.08,2.19)* | 1.34 (0.92,1.97) | 1.26 (0.86,1.86) | 1.30 (0.65,2.60) | 1.33 (0.65,2.74) | 1.32 (0.64,2.73) |
| **Two MetS Components** |  |  |  |  |  |  |  |  |  |
| None | 1.00 (Ref) | 1.00 (Ref) | 1.00 (Ref) | 1.00 (Ref) | 1.00 (Ref) | 1.00 (Ref) | 1.00 (Ref) | 1.00 (Ref) | 1.00 (Ref) |
| Elevated blood pressure + Reduced HDL cholesterol | **2.34 (1.24,4.42)**** | 1.84 (0.92,3.67) | 1.82 (0.91,3.63) | **1.87 (1.24,2.82)**** | 1.24 (0.78,1.99) | 1.26 (0.79,2.02) | **2.71 (1.42,5.17)**** | **2.05 (1.01,4.15)*** | **2.03 (1.00,4.12)** |
| Elevated blood pressure + Elevated triglycerides | 1.16 (0.62,2.15) | 0.99 (0.52,1.89) | 1.05 (0.55,2.02) | 1.15 (0.82,1.60) | 1.28 (0.88,1.86) | 1.29 (0.88,1.88) | 1.14 (0.59,2.21) | 0.98 (0.49,1.97) | 1.03 (0.51,2.07) |
| Elevated blood pressure + Elevated fasting glucose | 1.70 (0.98,2.94) | 1.26 (0.71,2.24) | 1.30 (0.72,2.34) | 1.19 (0.85,1.66) | 1.14 (0.78,1.66) | 1.17 (0.80,1.72) | 1.75 (0.98,3.13) | 1.29 (0.70,2.38) | 1.33 (0.71,2.48) |
| Abdominal obesity  + Elevated blood pressure | **1.82 (1.19,2.76)**** | **1.60 (1.02,2.48)*** | 1.56 (1.00,2.44) | **1.40 (1.10,1.80)**** | 1.13 (0.86,1.48) | 1.13 (0.86,1.48) | **1.78 (1.13,2.78)*** | 1.51 (0.94,2.43) | 1.47 (0.91,2.37) |
| Other combinations | 1.67 (0.88,3.18) | 1.37 (0.68,2.76) | 1.39 (0.69,2.82) | **1.49 (1.01,2.18)*** | 1.16 (0.75,1.78) | 1.16 (0.75,1.79) | **1.93 (1.01,3.72)*** | 1.53 (0.75,3.13) | 1.56 (0.76,3.20) |
| **Three MetS Components** |  |  |  |  |  |  |  |  |  |
| None | 1.00 (Ref) | 1.00 (Ref) | 1.00 (Ref) | 1.00 (Ref) | 1.00 (Ref) | 1.00 (Ref) | 1.00 (Ref) | 1.00 (Ref) | 1.00 (Ref) |
| Elevated blood pressure + Elevated triglycerides + Elevated fasting glucose | 1.85 (0.76,4.54) | 1.82 (0.72,4.56) | 2.12 (0.83,5.43) | 0.78 (0.41,1.47) | 0.88 (0.43,1.77) | 0.93 (0.45,1.91) | 1.38 (0.48,3.99) | 1.40 (0.47,4.17) | 1.73 (0.57,5.25) |
| Abdominal obesity  + Elevated blood pressure + Reduced HDL cholesterol | 2.42 (1.20,4.87)* | 2.02 (0.95,4.29) | 2.06 (0.97,4.41) | 1.58 (0.99,2.53) | 1.19 (0.70,2.04) | 1.17 (0.68,2.02) | 1.96 (0.88,4.34) | 1.55 (0.66,3.65) | 1.62 (0.69,3.84) |
| Abdominal obesity  + Elevated blood pressure + Elevated fasting glucose | **3.11 (1.73,5.58)***** | **2.39 (1.29,4.43)**** | **2.56 (1.36,4.81)**** | **2.47 (1.65,3.69)***** | **2.15 (1.36,3.41)**** | **2.23 (1.39,3.58)**** | **3.36 (1.83,6.17)***** | **2.51 (1.32,4.77)**** | **2.76 (1.42,5.34)**** |
| Abdominal obesity  + Elevated blood pressure + Elevated triglycerides | **1.89 (1.10,3.25)*** | **1.83 (1.03,3.23)*** | **1.94 (1.08,3.49)*** | 0.77 (0.53,1.11) | 0.74 (0.49,1.12) | 0.75 (0.49,1.15) | **1.96 (1.11,3.47)*** | **1.92 (1.05,3.50)*** | **2.11 (1.13,3.92)*** |
| Other combinations | **2.96 (1.55,5.62)**** | **2.17 (1.09,4.31)*** | **2.31 (1.14,4.65)*** | 1.49 (0.94,2.36) | 0.92 (0.54,1.56) | 0.92 (0.54,1.57) | **3.43 (1.78,6.58)***** | **2.50 (1.24,5.03)*** | **2.75 (1.34,5.64)**** |
| **Four MetS Components** |  |  |  |  |  |  |  |  |  |
| None | 1.00 (Ref) | 1.00 (Ref) | 1.00 (Ref) | 1.00 (Ref) | 1.00 (Ref) | 1.00 (Ref) | 1.00 (Ref) | 1.00 (Ref) | 1.00 (Ref) |
| Abdominal obesity  + Elevated blood pressure + Elevated triglycerides + Reduced HDL cholesterol | **2.55 (1.09,5.98)*** | 1.88 (0.74,4.76) | 1.73 (0.67,4.46) | 1.35 (0.74,2.47) | 0.77 (0.39,1.50) | 0.73 (0.37,1.43) | 2.48 (1.00,6.16) | 1.68 (0.62,4.58) | 1.60 (0.58,4.42) |
| Abdominal obesity  + Elevated blood pressure + Elevated triglycerides + Elevated fasting glucose | 1.93 (0.94,3.97) | 1.20 (0.53,2.74) | 1.18 (0.51,2.73) | 1.10 (0.69,1.77) | 1.07 (0.61,1.88) | 1.03 (0.58,1.83) | 1.99 (0.93,4.25) | 1.23 (0.52,2.94) | 1.25 (0.52,3.02) |
| Other combinations | 2.01 (0.68,5.91) | 1.72 (0.55,5.42) | 1.54 (0.48,4.91) | 1.04 (0.49,2.19) | 0.78 (0.34,1.78) | 0.74 (0.32,1.70) | 1.69 (0.50,5.75) | 1.43 (0.39,5.21) | 1.32 (0.36,4.88) |

**Notes:** *, *P*<0.05, **, *P*<0.01; ***, *P*<0.001. Model 1 was unadjusted model; Model 2 adjusted for sex, age, ethnic, marital status, educational attainment, occupation, annual income; Model 3 further adjusted cerebrovascular disease, rheumatism, osteoarthropathy, smoking status, and alcohol consumption.

**Abbeviations:** MetS, metabolic syndrome; ADL, activities of daily living; IADL, instrumental activities of daily living; PR, prevalence ratio; CI, confidence interval.

**Supplementary Table S27**. Associations between various combinations of MetS components and functional disability among Zhuang ethnic population

| Components/combinations | ADL disability [PR (95% CI)] | | | IADL disability [PR (95% CI)] | | | Comorbid ADL­IADL disability [PR (95% CI)] | | |
| --- | --- | --- | --- | --- | --- | --- | --- | --- | --- |
|  | Model 1 | Model 2 | Model 3 | Model 1 | Model 2 | Model 3 | Model 1 | Model 2 | Model 3 |
| **One MetS Component** |  |  |  |  |  |  |  |  |  |
| None | 1.00 (Ref) | 1.00 (Ref) | 1.00 (Ref) | 1.00 (Ref) | 1.00 (Ref) | 1.00 (Ref) | 1.00 (Ref) | 1.00 (Ref) | 1.00 (Ref) |
| Elevated blood pressure | **1.02 (1.00,1.05)*** | 1.01 (0.99,1.03) | 1.01 (0.99,1.03) | **1.04 (1.01,1.08)*** | 1.02 (0.99,1.05) | 1.02 (0.99,1.06) | **1.64 (1.09,2.46)*** | 1.29 (0.86,1.94) | 1.33 (0.89,2.01) |
| Elevated fasting glucose | 1.01 (0.96,1.07) | 1.00 (0.95,1.06) | 1.00 (0.95,1.06) | 1.02 (0.94,1.11) | 1.02 (0.94,1.11) | 1.02 (0.94,1.10) | 1.19 (0.43,3.29) | 1.05 (0.39,2.85) | 1.09 (0.41,2.95) |
| Elevated triglycerides | 1.00 (0.95,1.06) | 0.99 (0.94,1.04) | 0.99 (0.94,1.04) | 0.99 (0.90,1.09) | 1.02 (0.93,1.10) | 1.01 (0.93,1.10) | 1.21 (0.38,3.83) | 0.80 (0.25,2.63) | 0.78 (0.24,2.49) |
| Reduced HDL cholesterol | 1.01 (0.96,1.06) | 1.00 (0.95,1.04) | 1.00 (0.95,1.04) | **0.95 (0.88,1.03)** | **0.89 (0.83,0.95**** | **0.89 (0.83,0.95)**** | 1.41 (0.56,3.54) | 1.10 (0.50,2.44) | 1.13 (0.51,2.51) |
| Abdominal obesity | 1.02 (0.97,1.06) | 1.02 (0.97,1.06) | 1.02 (0.97,1.06) | 1.04 (0.98,1.11) | 1.02 (0.95,1.08) | 1.00 (0.94,1.07) | 1.40 (0.66,2.98) | 1.45 (0.70,3.02) | 1.43 (0.69,2.99) |
| **Two MetS Components** |  |  |  |  |  |  |  |  |  |
| None | 1.00 (Ref) | 1.00 (Ref) | 1.00 (Ref) | 1.00 (Ref) | 1.00 (Ref) | 1.00 (Ref) | 1.00 (Ref) | 1.00 (Ref) | 1.00 (Ref) |
| Elevated blood pressure + Reduced HDL cholesterol | 1.05 (0.99,1.11) | 1.03 (0.97,1.09) | 1.03 (0.97,1.09) | **1.11 (1.03,1.20)**** | 1.03 (0.96,1.10) | 1.03 (0.96,1.10) | **2.39 (1.21,4.72)*** | 1.64 (0.86,3.13) | 1.62 (0.84,3.12) |
| Elevated blood pressure + Elevated triglycerides | 1.01 (0.97,1.05) | 1.00 (0.96,1.04) | 1.01 (0.97,1.04) | 1.01 (0.95,1.07) | 1.02 (0.97,1.08) | 1.02 (0.96,1.08) | 1.30 (0.65,2.61) | 1.12 (0.58,2.14) | 1.17 (0.62,2.22) |
| Elevated blood pressure + Elevated fasting glucose | 1.02 (0.98,1.06) | 1.00 (0.96,1.04) | 1.00 (0.96,1.04) | 1.01 (0.96,1.07) | 1.00 (0.95,1.06) | 1.00 (0.95,1.06) | 1.50 (0.78,2.87) | 1.09 (0.59,2.00) | 1.12 (0.61,2.09) |
| Abdominal obesity  + Elevated blood pressure | **1.05 (1.02,1.08)**** | **1.04 (1.00,1.07)*** | **1.04 (1.00,1.07)*** | **1.08 (1.04,1.13)***** | 1.04 (1.00,1.08) | 1.04 (1.00,1.08) | **2.10 (1.32,3.34)**** | **1.79 (1.11,2.90)*** | **1.82 (1.12,2.93)*** |
| Other combinations | 1.03 (0.98,1.08) | 1.03 (0.98,1.07) | 1.03 (0.98,1.07) | 1.07 (1.00,1.15) | 1.02 (0.96,1.09) | 1.02 (0.96,1.09) | 1.91 (0.93,3.91) | 1.40 (0.78,2.53) | 1.44 (0.79,2.61) |
| **Three MetS Components** |  |  |  |  |  |  |  |  |  |
| None | 1.00 (Ref) | 1.00 (Ref) | 1.00 (Ref) | 1.00 (Ref) | 1.00 (Ref) | 1.00 (Ref) | 1.00 (Ref) | 1.00 (Ref) | 1.00 (Ref) |
| Elevated blood pressure + Elevated triglycerides + Elevated fasting glucose | 1.01 (0.95,1.08) | 1.01 (0.94,1.08) | 1.01 (0.94,1.09) | 0.96 (0.87,1.05) | 0.98 (0.90,1.07) | 0.98 (0.89,1.07) | 0.94 (0.23,3.80) | 0.96 (0.23,4.00) | 1.13 (0.26,4.94) |
| Abdominal obesity  + Elevated blood pressure + Reduced HDL cholesterol | **1.08 (1.01,1.16)*** | 1.06 (0.98,1.14) | 1.06 (0.98,1.14) | 1.08 (0.99,1.17) | 1.02 (0.95,1.10) | 1.02 (0.94,1.10) | 2.20 (1.00,4.82) | 1.65 (0.73,3.72) | 1.68 (0.74,3.81) |
| Abdominal obesity  + Elevated blood pressure + Elevated fasting glucose | **1.10 (1.03,1.18)**** | **1.08 (1.01,1.15)*** | **1.08 (1.01,1.15)*** | **1.18 (1.09,1.28)***** | **1.13 (1.05,1.21)**** | **1.13 (1.05,1.22)**** | **3.63 (1.97,6.68)***** | **2.65 (1.43,4.90)**** | **2.76 (1.47,5.18)**** |
| Abdominal obesity  + Elevated blood pressure + Elevated triglycerides | 1.04 (0.99,1.09) | 1.03 (0.99,1.08) | 1.03 (0.99,1.08) | 0.97 (0.91,1.02) | 0.97 (0.92,1.02) | 0.97 (0.92,1.03) | **1.98 (1.07,3.65)*** | 1.69 (0.94,3.03) | 1.80 (0.98,3.29) |
| Other combinations | 1.07 (1.00,1.15) | 1.03 (0.97,1.10) | 1.03 (0.97,1.10) | 1.04 (0.95,1.13) | 0.96 (0.89,1.03) | 0.95 (0.89,1.03) | **2.86 (1.42,5.79)**** | 1.79 (0.94,3.40) | 1.86 (0.96,3.62) |
| **Four MetS Components** |  |  |  |  |  |  |  |  |  |
| None | 1.00 (Ref) | 1.00 (Ref) | 1.00 (Ref) | 1.00 (Ref) | 1.00 (Ref) | 1.00 (Ref) | 1.00 (Ref) | 1.00 (Ref) | 1.00 (Ref) |
| Abdominal obesity  + Elevated blood pressure + Elevated triglycerides + Reduced HDL cholesterol | 1.05 (0.96,1.15) | 1.03 (0.96,1.11) | 1.03 (0.95,1.11) | 1.02 (0.91,1.15) | 0.95 (0.86,1.06) | 0.95 (0.85,1.06) | 2.41 (0.90,6.49) | 1.74 (0.76,3.97) | 1.68 (0.72,3.93) |
| Abdominal obesity  + Elevated blood pressure + Elevated triglycerides + Elevated fasting glucose | 1.07 (1.00,1.14) | 1.05 (0.98,1.12) | 1.05 (0.98,1.12) | 1.03 (0.95,1.13) | 1.03 (0.95,1.11) | 1.02 (0.94,1.11) | **2.51 (1.20,5.27)*** | 1.67 (0.83,3.36) | 1.65 (0.81,3.36) |
| Other combinations | 1.05 (0.95,1.16) | 1.03 (0.94,1.13) | 1.02 (0.93,1.13) | 1.01 (0.89,1.15) | 0.97 (0.86,1.10) | 0.97 (0.86,1.09) | 1.58 (0.40,6.29) | 1.20 (0.29,4.97) | 1.10 (0.26,4.72) |

**Notes:** *, *P*<0.05, **, *P*<0.01; ***, *P*<0.001. Model 1 was unadjusted model; Model 2 adjusted for sex, age, ethnic, marital status, educational attainment, occupation, annual income; Model 3 further adjusted cerebrovascular disease, rheumatism, osteoarthropathy, smoking status, and alcohol consumption.

**Abbreviations:** MetS, metabolic syndrome; ADL, activities of daily living; IADL, instrumental activities of daily living; PR, prevalence ratio; CI, confidence interval.

**Supplementary Table S28**. Associations between various combinations of MetS components and functional disability among farmers

| Components/combinations | ADL disability [PR (95% CI)] | | | IADL disability [PR (95% CI)] | | | Comorbid ADL­IADL disability [PR (95% CI)] | | |
| --- | --- | --- | --- | --- | --- | --- | --- | --- | --- |
|  | Model 1 | Model 2 | Model 3 | Model 1 | Model 2 | Model 3 | Model 1 | Model 2 | Model 3 |
| **One MetS Component** |  |  |  |  |  |  |  |  |  |
| None | 1.00 (Ref) | 1.00 (Ref) | 1.00 (Ref) | 1.00 (Ref) | 1.00 (Ref) | 1.00 (Ref) | 1.00 (Ref) | 1.00 (Ref) | 1.00 (Ref) |
| Elevated blood pressure | **1.02 (1.00,1.04)*** | 1.00 (0.98,1.02) | 1.00 (0.98,1.03) | **1.04 (1.00,1.07)*** | 1.01 (0.98,1.05) | 1.02 (0.99,1.05) | **1.50 (1.04,2.17)*** | 1.15 (0.80,1.67) | 1.16 (0.80,1.69) |
| Elevated fasting glucose | 1.01 (0.96,1.07) | 1.01 (0.96,1.06) | 1.01 (0.96,1.06) | 1.01 (0.93,1.09) | 1.02 (0.94,1.10) | 1.02 (0.94,1.10) | 1.16 (0.47,2.88) | 1.11 (0.46,2.67) | 1.13 (0.47,2.73) |
| Elevated triglycerides | 1.00 (0.94,1.06) | 0.99 (0.94,1.04) | 0.99 (0.94,1.04) | 0.96 (0.88,1.06) | 0.98 (0.90,1.06) | 0.98 (0.90,1.06) | 1.08 (0.34,3.39) | 0.71 (0.22,2.30) | 0.70 (0.23,2.12) |
| Reduced HDL cholesterol | 1.02 (0.97,1.08) | 1.01 (0.96,1.06) | 1.01 (0.96,1.06) | 0.96 (0.89,1.04) | **0.89 (0.83,0.95)**** | **0.89 (0.84,0.96)**** | 1.56 (0.72,3.39) | 1.22 (0.64,2.34) | 1.24 (0.64,2.41) |
| Abdominal obesity | 1.02 (0.98,1.06) | 1.02 (0.98,1.06) | 1.02 (0.98,1.06) | **1.08 (1.02,1.15)*** | 1.05 (0.99,1.11) | 1.04 (0.98,1.10) | 1.39 (0.72,2.66) | 1.35 (0.73,2.52) | 1.33 (0.71,2.48) |
| **Two MetS Components** |  |  |  |  |  |  |  |  |  |
| None | 1.00 (Ref) | 1.00 (Ref) | 1.00 (Ref) | 1.00 (Ref) | 1.00 (Ref) | 1.00 (Ref) | 1.00 (Ref) | 1.00 (Ref) | 1.00 (Ref) |
| Elevated blood pressure + Reduced HDL cholesterol | **1.08 (1.02,1.16)*** | 1.05 (0.99,1.12) | 1.05 (0.99,1.12) | **1.14 (1.06,1.22)**** | 1.04 (0.97,1.11) | 1.04 (0.97,1.11) | **2.89 (1.62,5.14)***** | **1.90 (1.09,3.31)*** | **1.87 (1.07,3.26)*** |
| Elevated blood pressure + Elevated triglycerides | 1.02 (0.98,1.06) | 1.00 (0.97,1.04) | 1.01 (0.97,1.05) | 1.03 (0.97,1.08) | 1.04 (0.98,1.09) | 1.04 (0.98,1.10) | 1.25 (0.67,2.35) | 1.08 (0.61,1.94) | 1.12 (0.63,2.01) |
| Elevated blood pressure + Elevated fasting glucose | 1.03 (0.99,1.07) | 1.01 (0.97,1.05) | 1.01 (0.97,1.05) | 1.02 (0.97,1.08) | 1.01 (0.96,1.06) | 1.01 (0.96,1.06) | 1.68 (0.96,2.96) | 1.21 (0.71,2.06) | 1.22 (0.71,2.09) |
| Abdominal obesity  + Elevated blood pressure | **1.05 (1.02,1.08)**** | **1.04 (1.00,1.07)*** | **1.03 (1.00,1.07)*** | **1.06 (1.02,1.11)**** | 1.02 (0.98,1.06) | 1.02 (0.98,1.06) | **1.84 (1.20,2.83)**** | 1.51 (0.97,2.35) | 1.47 (0.94,2.30) |
| Other combinations | 1.02 (0.97,1.06) | 1.01 (0.97,1.05) | 1.01 (0.97,1.05) | 1.06 (0.99,1.14) | 1.00 (0.94,1.06) | 1.00 (0.94,1.06) | 1.47 (0.73,2.97) | 1.08 (0.59,1.99) | 1.09 (0.59,2.02) |
| **Three MetS Components** |  |  |  |  |  |  |  |  |  |
| None | 1.00 (Ref) | 1.00 (Ref) | 1.00 (Ref) | 1.00 (Ref) | 1.00 (Ref) | 1.00 (Ref) | 1.00 (Ref) | 1.00 (Ref) | 1.00 (Ref) |
| Elevated blood pressure + Elevated triglycerides + Elevated fasting glucose | 1.06 (0.98,1.15) | 1.05 (0.96,1.14) | 1.06 (0.98,1.15) | 0.98 (0.89,1.08) | 0.98 (0.89,1.08) | 0.99 (0.90,1.09) | 1.58 (0.59,4.26) | 1.50 (0.56,4.07) | 1.81 (0.65,4.99) |
| Abdominal obesity  + Elevated blood pressure + Reduced HDL cholesterol | **1.08 (1.00,1.16)*** | 1.05 (0.98,1.13) | 1.05 (0.98,1.14) | **1.11 (1.02,1.21)*** | 1.03 (0.96,1.12) | 1.03 (0.96,1.11) | **2.17 (1.05,4.48)*** | 1.57 (0.72,3.44) | 1.63 (0.73,3.61) |
| Abdominal obesity  + Elevated blood pressure + Elevated fasting glucose | **1.10 (1.03,1.17)**** | **1.07 (1.00,1.14)*** | **1.07 (1.01,1.14)*** | **1.18 (1.10,1.26)***** | **1.11 (1.04,1.19)**** | **1.12 (1.04,1.19)**** | **3.03 (1.73,5.31)***** | **2.12 (1.19,3.78)*** | **2.28 (1.25,4.17)**** |
| Abdominal obesity  + Elevated blood pressure + Elevated triglycerides | **1.05 (1.00,1.10)*** | 1.04 (1.00,1.09) | **1.05 (1.00,1.09)*** | 0.97 (0.91,1.03) | 0.95 (0.90,1.01) | 0.96 (0.90,1.01) | **1.88 (1.07,3.31)*** | 1.65 (0.96,2.84) | 1.80 (1.01,3.19)* |
| Other combinations | **1.12 (1.04,1.20)**** | **1.08 (1.01,1.15)*** | **1.08 (1.01,1.16)*** | 1.08 (0.99,1.17) | 0.98 (0.91,1.06) | 0.98 (0.91,1.06) | **3.55 (2.01,6.27)***** | **2.38 (1.38,4.09)**** | **2.52 (1.41,4.50)**** |
| **Four MetS Components** |  |  |  |  |  |  |  |  |  |
| None | 1.00 (Ref) | 1.00 (Ref) | 1.00 (Ref) | 1.00 (Ref) | 1.00 (Ref) | 1.00 (Ref) | 1.00 (Ref) | 1.00 (Ref) | 1.00 (Ref) |
| Abdominal obesity  + Elevated blood pressure + Elevated triglycerides + Reduced HDL cholesterol | 1.08 (0.99,1.18) | 1.05 (0.97,1.14) | 1.05 (0.97,1.13) | 1.05 (0.95,1.16) | 0.97 (0.88,1.06) | 0.96 (0.87,1.05) | **2.42 (1.08,5.46)*** | 1.53 (0.72,3.28) | 1.45 (0.67,3.15) |
| Abdominal obesity  + Elevated blood pressure + Elevated triglycerides + Elevated fasting glucose | 1.05 (0.99,1.12) | 1.02 (0.96,1.08) | 1.02 (0.96,1.08) | 1.03 (0.95,1.12) | 1.00 (0.93,1.08) | 1.00 (0.92,1.08) | 1.98 (0.96,4.10) | 1.13 (0.54,2.38) | 1.20 (0.57,2.49) |
| Other combinations | 1.07 (0.96,1.19) | 1.05 (0.95,1.16) | 1.05 (0.95,1.15) | 1.03 (0.91,1.17) | 0.97 (0.86,1.10) | 0.97 (0.85,1.09) | 1.92 (0.62,5.87) | 1.48 (0.48,4.60) | 1.39 (0.44,4.41) |
|  |  |  |  |  |  |  |  |  |  |

**Notes:** *, *P*<0.05, **, *P*<0.01; ***, *P*<0.001. Model 1 was unadjusted model; Model 2 adjusted for sex, age, ethnic, marital status, educational attainment, occupation, annual income;

Model 3 further adjusted cerebrovascular disease, rheumatism, osteoarthropathy, smoking status, and alcohol consumption.

**Abbreviations:** MetS, metabolic syndrome; ADL, activities of daily living; IADL, instrumental activities of daily living; PR, prevalence ratio; CI, confidence interval.

**Supplementary Table S29.** Associations between combinations of MetS components and functional disability among non–drinking population

| Components/combinations | ADL disability [PR (95% CI)] | | | IADL disability [PR (95% CI)] | | | Comorbid ADL­IADL disability [PR (95% CI)] | | |
| --- | --- | --- | --- | --- | --- | --- | --- | --- | --- |
|  | Model 1 | Model 2 | Model 3 | Model 1 | Model 2 | Model 3 | Model 1 | Model 2 | Model 3 |
| **One MetS Component** |  |  |  |  |  |  |  |  |  |
| None | 1.00 (Ref) | 1.00 (Ref) | 1.00 (Ref) | 1.00 (Ref) | 1.00 (Ref) | 1.00 (Ref) | 1.00 (Ref) | 1.00 (Ref) | 1.00 (Ref) |
| Elevated blood pressure | 1.02 (0.99,1.04) | 1.00 (0.97,1.02) | 0.99 (0.97,1.02) | **1.05 (1.02,1.09)**** | 1.02 (0.99,1.06) | 1.02 (0.98,1.05) | 1.39 (0.94,2.05) | 1.06 (0.71,1.57) | 1.04 (0.70,1.55) |
| Elevated fasting glucose | 0.99 (0.93,1.05) | 0.98 (0.92,1.04) | 0.98 (0.92,1.04) | 1.07 (0.97,1.17) | 1.05 (0.95,1.15) | 1.04 (0.95,1.14) | 0.96 (0.31,3.04) | 0.88 (0.29,2.71) | 0.87 (0.28,2.69) |
| Elevated triglycerides | 1.02 (0.95,1.09) | 1.01 (0.95,1.07) | 1.00 (0.95,1.06) | 0.99 (0.90,1.10) | 1.03 (0.93,1.13) | 1.02 (0.93,1.12) | 1.44 (0.53,3.89) | 1.09 (0.41,2.88) | 0.96 (0.36,2.56) |
| Reduced HDL cholesterol | 1.02 (0.96,1.08) | 1.01 (0.96,1.06) | 1.00 (0.96,1.06) | 0.96 (0.89,1.04) | **0.90 (0.84,0.96)**** | **0.90 (0.84,0.96)**** | 1.50 (0.69,3.27) | 1.21 (0.63,2.32) | 1.17 (0.60,2.30) |
| Abdominal obesity | 1.00 (0.96,1.05) | 1.01 (0.96,1.05) | 1.00 (0.96,1.05) | **1.07 (1.01,1.14)*** | 1.05 (0.99,1.11) | 1.04 (0.98,1.10) | 1.10 (0.54,2.24) | 1.11 (0.57,2.19) | 1.09 (0.55,2.15) |
| **Two MetS Components** |  |  |  |  |  |  |  |  |  |
| None | 1.00 (Ref) | 1.00 (Ref) | 1.00 (Ref) | 1.00 (Ref) | 1.00 (Ref) | 1.00 (Ref) | 1.00 (Ref) | 1.00 (Ref) | 1.00 (Ref) |
| Elevated blood pressure + Reduced HDL cholesterol | 1.06 (1.00,1.13) | 1.04 (0.99,1.11) | 1.04 (0.99,1.11) | **1.11 (1.03,1.20)**** | 1.04 (0.97,1.12) | 1.04 (0.97,1.12) | **2.32 (1.27,4.23)**** | **1.79 (1.00,3.19)*** | **1.86 (1.03,3.38)*** |
| Elevated blood pressure + Elevated triglycerides | 1.02 (0.97,1.07) | 1.01 (0.96,1.06) | 1.01 (0.96,1.06) | 1.06 (0.99,1.14) | 1.04 (0.98,1.12) | 1.04 (0.97,1.11) | 1.24 (0.59,2.61) | 1.10 (0.53,2.26) | 1.06 (0.53,2.15) |
| Elevated blood pressure + Elevated fasting glucose | 1.03 (0.98,1.08) | 1.00 (0.95,1.05) | 0.99 (0.95,1.04) | 1.06 (0.99,1.13) | 1.02 (0.96,1.08) | 1.02 (0.96,1.08) | 1.56 (0.81,2.99) | 1.17 (0.64,2.16) | 1.15 (0.61,2.15) |
| Abdominal obesity  + Elevated blood pressure | **1.04 (1.00,1.07)*** | 1.02 (0.99,1.06) | 1.02 (0.99,1.06) | **1.06 (1.01,1.11)*** | 1.01 (0.97,1.06) | 1.01 (0.97,1.06) | **1.60 (1.02,2.52)*** | 1.35 (0.85,2.15) | 1.35 (0.84,2.16) |
| Other combinations | 1.02 (0.97,1.07) | 1.02 (0.97,1.06) | 1.02 (0.97,1.06) | 1.07 (1.00,1.14) | 1.03 (0.97,1.09) | 1.03 (0.97,1.09) | 1.50 (0.76,2.95) | 1.24 (0.69,2.24) | 1.28 (0.71,2.31) |
| **Three MetS Components** |  |  |  |  |  |  |  |  |  |
| None | 1.00 (Ref) | 1.00 (Ref) | 1.00 (Ref) | 1.00 (Ref) | 1.00 (Ref) | 1.00 (Ref) | 1.00 (Ref) | 1.00 (Ref) | 1.00 (Ref) |
| Elevated blood pressure + Elevated triglycerides + Elevated fasting glucose | 1.04 (0.94,1.15) | 1.02 (0.92,1.14) | 1.02 (0.92,1.14) | 1.04 (0.91,1.19) | 1.03 (0.91,1.16) | 1.02 (0.91,1.16) | 1.86 (0.61,5.70) | 1.71 (0.55,5.34) | 1.76 (0.56,5.51) |
| Abdominal obesity  + Elevated blood pressure + Reduced HDL cholesterol | 1.07 (1.00,1.15) | 1.05 (0.98,1.13) | 1.06 (0.98,1.14) | 1.07 (0.99,1.16) | 1.03 (0.96,1.11) | 1.02 (0.95,1.10) | 1.82 (0.88,3.79) | 1.60 (0.74,3.47) | 1.67 (0.77,3.62) |
| Abdominal obesity  + Elevated blood pressure + Elevated fasting glucose | **1.13 (1.05,1.22)**** | **1.10 (1.02,1.18)*** | **1.10 (1.02,1.19)*** | **1.21 (1.12,1.30)***** | **1.14 (1.07,1.23)***** | **1.14 (1.06,1.22)***** | **3.64 (2.14,6.21)***** | **2.78 (1.59,4.87)***** | **2.90 (1.62,5.17)***** |
| Abdominal obesity  + Elevated blood pressure + Elevated triglycerides | **1.05 (1.00,1.11)*** | 1.04 (0.99,1.10) | 1.05 (1.00,1.10) | 0.98 (0.92,1.04) | 0.97 (0.91,1.03) | 0.96 (0.91,1.02) | **2.04 (1.18,3.54)*** | **1.84 (1.07,3.19)*** | **1.94 (1.10,3.42)*** |
| Other combinations | **1.09 (1.01,1.17)*** | 1.06 (0.99,1.13) | 1.06 (0.99,1.13) | 1.05 (0.97,1.14) | 0.96 (0.90,1.04) | 0.96 (0.89,1.03) | **2.84 (1.54,5.24)**** | **2.14 (1.21,3.79)**** | **2.28 (1.27,4.10)**** |
| **Four MetS Components** |  |  |  |  |  |  |  |  |  |
| None | 1.00 (Ref) | 1.00 (Ref) | 1.00 (Ref) | 1.00 (Ref) | 1.00 (Ref) | 1.00 (Ref) | 1.00 (Ref) | 1.00 (Ref) | 1.00 (Ref) |
| Abdominal obesity  + Elevated blood pressure + Elevated triglycerides + Reduced HDL cholesterol | 1.08 (0.99,1.18) | 1.06 (0.98,1.15) | 1.06 (0.97,1.15) | 1.06 (0.95,1.17) | 0.99 (0.90,1.09) | 0.98 (0.89,1.08) | **2.30 (1.02,5.19)*** | 1.78 (0.82,3.86) | 1.80 (0.81,4.00) |
| Abdominal obesity  + Elevated blood pressure + Elevated triglycerides + Elevated fasting glucose | 1.07 (0.99,1.16) | 1.03 (0.95,1.11) | 1.02 (0.94,1.10) | 1.07 (0.97,1.18) | 1.03 (0.94,1.13) | 1.03 (0.93,1.13) | **2.48 (1.21,5.10)*** | 1.52 (0.70,3.28) | 1.40 (0.66,2.97) |
| Other combinations | 1.03 (0.94,1.12) | 1.02 (0.93,1.11) | 1.01 (0.93,1.10) | 0.99 (0.87,1.12) | 0.97 (0.87,1.09) | 0.96 (0.86,1.08) | 1.09 (0.27,4.34) | 1.03 (0.26,4.09) | 0.97 (0.24,3.90) |

**Abbreviations:** MetS, metabolic syndrome; ADL, activities of daily living; IADL, instrumental activities of daily living; PR, prevalence ratio; CI, confidence interval.

**Notes:** *, *P*<0.05, **, *P*<0.01; ***, *P*<0.001. Model 1 was unadjusted model; Model 2 adjusted for sex, age, ethnic, marital status, educational attainment, occupation, annual income; Model 3 further adjusted cerebrovascular disease, rheumatism, osteoarthropathy, smoking status, and alcohol consumption.
